# Bromide-mediated enhancement of far-UVC/peracetic acid process for selective micropollutant abatement

**DOI:** 10.1038/s41467-026-78410-2

**Published:** 2026-10-08

**Authors:** Genying Zeng, Guoyu Li, Hou Wang, Chen Zhang, Qiang Chen, Qinyuan Tang, Tangshan Wu, Li Li, Xinjiang Hu, Ran Yin, Xiaofei Tan

**Affiliations:** 1 College of Environmental Science and Engineering and Key Laboratory of Environmental Biology and Pollution Control (Ministry of Education), Hunan University, Changsha, China; 2 Shenzhen Research Institute of Hunan University, Shenzhen, China; 3 School of Plant Protection and Environment, Henan Institute of Science and Technology, Xinxiang, Henan China; 4 School of Ecology and Environment, Central South University of Forestry and Technology, Changsha, China; 5 State Key Laboratory of Water Pollution Control and Green Resource Reuse, School of the Environment, Nanjing University, Nanjing, China; 6 Institute for the Environment and Health, Nanjing University Suzhou Campus, Suzhou, China

**Keywords:** Environmental sciences, Environmental chemistry, Pollution remediation

## Abstract

Far‑ultraviolet 222 nm (UV_222_) effectively activates oxidants to degrade organic micropollutants (OMPs) in advanced oxidation processes (AOPs), but its synergy and ecotoxicity with bromide (Br^−^) are unclear. This study investigates the synergy of peracetic acid (PAA) and Br^−^ by UV_222_ (UV_222_-PB) for OMPs degradation. The system’s fluence-based rate constant for model pollutants (0.0159 cm^2^·mJ^−1^) is 3.01 and 15.74 times that of UV_254_-PB and UV_365_-PB. Radical and theoretical analyses show that far‑ultraviolet strengthens the interaction between PAA and Br^−^, prolongs radical chains, and boosts organic radicals and reactive bromine species, dominating electron‑rich OMPs oxidation. Correlations of ln(*k*_obs_) with vertical ionization potential and highest occupied molecular orbital energy for 10 OMPs confirm selective oxidation. UV_222_-PB performs robustly in various water matrices and shows low ecotoxicity in assays such as seed germination and zebrafish embryo. Life cycle assessment confirms its sustainability. These findings offer insights and guidance for UV_222_-based AOPs in brominated water treatment.

## Abstract (web summary)

The study confirms far-UVC/peracetic acid boosts reactive species in bromide-rich water. It degrades micropollutants efficiently while reducing brominated byproducts and ecotoxicity, offering a cleaner solution for brominated wastewater treatment.

## Introduction

Ultraviolet-based advanced oxidation processes (UV-AOPs) are effective for eliminating recalcitrant organic micropollutants (OMPs) from water sources, leveraging high degradation efficiency and minimal secondary pollution^1^. Oxidizing agents like hydrogen peroxide (H_2_O_2_), peracetic acid (PAA), and peroxymonosulfate (PMS) can be activated by UV radiation to generate highly reactive species with strong oxidizing properties (e.g., •OH, •SO_4_^−^), enabling rapid degradation of OMPs^2,3^. Among them, PAA, an oxidation disinfectant with high oxidation potential (1.06–1.96 V), low toxicity, and high efficiency, has emerged as the preferred method for wastewater treatment^4,5^. Moreover, compared to H_2_O_2_ (213 kJ·mol^−1^) and PMS (317 kJ·mol^−1^), the O–O bond energy in PAA (159 kJ·mol^−1^) is more readily activated to generate radicals^6^. Research shows that UV_185_ and UV_254_ irradiation can effectively activate PAA, generating multiple reactive species, including hydroxyl radicals (•OH) and organic radicals (•RO)^7,8^. At present, the ultraviolet radiation sources commonly used in UV-AOP are 254 nm low-pressure ultraviolet (LPUV) lamps and 200–400 nm medium-pressure ultraviolet (MPUV) lamps^9^. However, conventional mercury lamps generally suffer from low photonic quantum efficiency and insufficient utilization of oxidizing agents, making it difficult to efficiently degrade large amounts of OMPs at 254 nm and longer wavelengths^10^. Moreover, mercury lamps pose risks such as mercury leakage and mercury contamination during production and use, causing harm to soil and water environments^11^. Therefore, the utilization of environmentally friendly light sources with high photochemical utilization has become a trend in the development of UV-AOPs.

Currently, krypton chloride (KrCl*) excimer lamp has emerged as a highly promising candidate for UV-AOPs systems. Compared with conventional low-pressure mercury lamps, the UV_222_ emerging UV light source offers advantages such as being mercury-free, having higher photon energy, and being safer for human health^12^. Specifically, the photon energy of UV_222_ (539 kJ·mol^−1^) is higher than that of UV_254_ (471 kJ·mol^−1^), enabling it to break stronger chemical bonds and activate various oxidants more efficiently^13^. In multiple studies, UV_222_ outperformed UV_254_ in the direct photolysis of OMPs and the generation of reactive species, requiring a lower UV dose and thereby significantly reducing treatment energy consumption and associated economic costs^10,14^. Hu et al. reported that at the same UV dose, the UV_222_/PAA process achieved a removal efficiency of 90.8% for carbamazepine (CBZ), compared to only 15.1% for UV_254_/PAA, while also exhibiting better electrical energy per order (EE/O) performance and lower energy consumption^15^. Moreover, multiple oxidizing agents, including H_2_O_2_, persulfate (PDS), and PMS, exhibit higher light absorption intensity at 222 nm than at 254 nm^16^. According to reports, the O − O bond in PAA absorbs significantly below 240 nm, with higher molar absorptivity (*ε*, M^−1^·cm^−1)^ and quantum yield (Φ, M·Einstein^−1^) at 222 nm^17^. Yin et al. found that switching from UV_254_ to UV_222_ improved the steady-state concentration of •OH produced by UV/PAA process by 4.1-fold, while •CH_3_C(O)O increased dramatically from undetectable levels^18^. Multiple studies further confirm that, owing to the higher photon energy at 222 nm and enhanced PAA photolysis efficiency, the UV_222_-PAA system could more efficiently generate reactive radicals^3^. Consequently, it exhibited significantly superior degradation kinetics compared to the conventional UV_254_-PAA system for various micropollutants^19,20^. However, the current single UV_222_/PAA process may still have restrictions such as low energy conversion efficiency and yield of reactive species, with poor selectivity, limiting its practical applicability in water treatment.

It has been reported that under far-UVC irradiation, reactive species formed from inorganic anions in natural water matrices play a significant role in driving pollutant degradation^10^. Yin et al. discovered that UV_222_ irradiation boosted the breakdown rates of four pesticides in surface water by 37.1 − 144.75 times compared to UV_254_, remarkably enhancing the generation of •OH and reactive nitrogen species^21^. Furthermore, bromide (Br^−^), a common ion in natural water and wastewater, exhibits significant light absorption properties under far-UVC radiation (*ε*_222_ = 77.6 M^−1^·cm^−1^)^10^. It could be directly photoactivated under UV_222_ via a water cluster-mediated charge transfer mechanism, generating bromine radicals (•Br)^10,22^. Moreover, Br^−^ has also garnered attention in AOP for its ability to react with oxidants to form hypobromous acid (HOBr), a strong secondary oxidant that rapidly reacts with organic compounds such as phenolic compounds (*k* ≈ 10^3^−10^5 ^M^−1^·s^−1^) and amines (*k* ≈ 10^5^ − 10^6 ^M^−1^·s^−1^)^23,24^. Shao et al. discovered that adding Br^−^ at different pH levels enhances BPA breakdown in the UV_254_/PAA process by generating active bromine species^25^. Seawater and water supply systems affected by seawater intrusion, as well as industrial wastewater, contain elevated levels of Br^−^^26^. Among these, as a typical high-brine water body, seawater can reach Br^−^ concentrations of up to 0.5 mM. However, while bromine-rich water bodies provide more active bromine species to promote pollutant degradation, they also introduce the bromate dilemma. Excessive oxidants and radicals can further oxidize reactive bromine into carcinogens such as bromate^22,27^. To date, the formation patterns of disinfection byproducts and associated risks in far-UVC field resulting from the combined use of halide ions and emerging oxidants require further investigation. More importantly, adding Br^−^ to the UV_222_-PAA process is expected to extend the radical generation chain and increase radical concentration. Under the same UV doses and oxidant equivalents, this approach enables more efficient OMPs degradation kinetics in a shorter timeframe, with its synergistic pathways pending systematic elucidation.

In this work, we compared the degradation efficiency and economic feasibility of different processes under irradiation with multiple wavelength light sources. Subsequently, various analytical techniques, theoretical calculations, and kinetic models were employed to deeply elucidate the generation of reactive species, synergistic mechanisms, and selective degradation mechanisms in the UV_222_-PB process. The transformation pathways of OMPs in the UV_222_-PB process are revealed in conjunction with theoretical calculations, and the ecotoxicological effects and the formation patterns of disinfection byproducts (DBPs) are evaluated. Finally, the environmental stability, practical application potential, and environmental-economic impacts of the UV_222_-PB process are comprehensively evaluated. Collectively, this research reveals the interaction between oxidants and natural water matrices at 222 nm and its environmental implications, providing insightful guidance for practical water treatment applications.

## Results

### Effect of ultraviolet wavelength on the performance of the UV-PAA/Br process

**Fig. 1:**
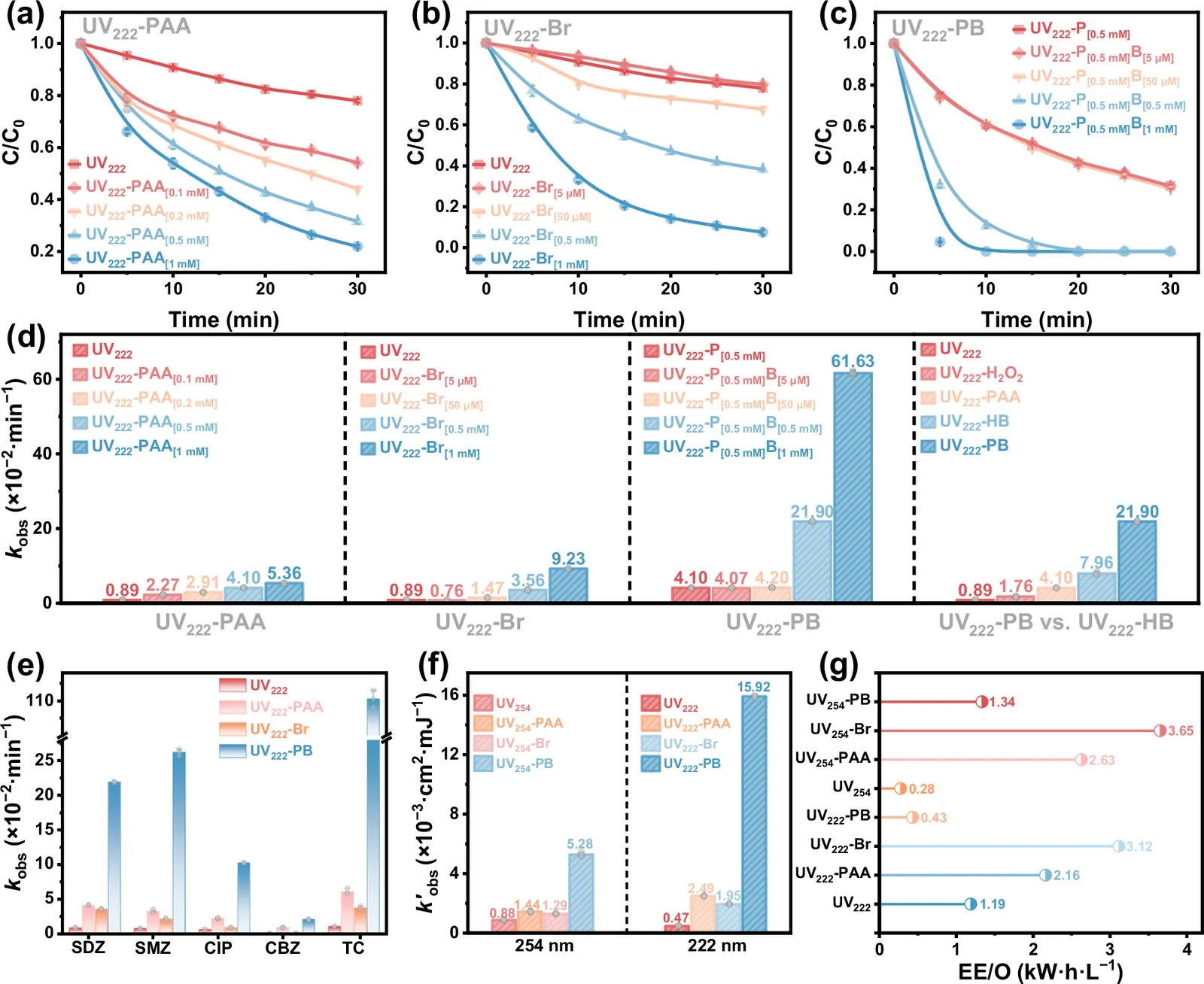
Efficient sulfadiazine (SDZ) degradation and characteristic evaluation under UV-based processes. **a**−**c** SDZ degradation curves under various UV_222_ processes, and (**d**) time-based pseudo-first-order rate constant (*k*_obs_) values. (**e**) *k*_obs_ values for the degradation of various organic micropollutants (OMPs) under the UV_222_ process. The selected OMPs include SDZ, sulfamethazine (SMZ), ciprofloxacin (CIP), carbamazepine (CBZ), tetracycline hydrochloride (TC). **f** Fluence-based pseudo-first-order rate constant (*k*′_obs_) values for various UV_222_ and UV_254_ processes. **g** Electrical energy per order (EE/O) values during SDZ degradation using various processes. Conditions: [SDZ]_0_ = 20 μM, pH = 7.0, [H_2_O_2_]_0_ = 200 μM, [PAA]_0_ = 0.1 mM–1 mM, [Br]_0_ = 5 μM–1 mM (unless otherwise mentioned, [PAA]_0_ = [Br]_0_ = 0.5 mM). Error bars represent SD of three replicate experiments, and the central value is the mean of the data. Data are presented as mean values ± SD. Source data are provided as a Source Data file.

The removal performance of SDZ under UV_222_ irradiation was evaluated for various processes. The results showed that under dark conditions, the degradation of SDZ by PAA alone or Br^−^ alone was almost negligible (Supplementary Fig. 1a). When exposed to UV_222_ radiation, SDZ was deteriorated by only 22.05% via direct photolysis (Fig. 1a). Notably, as the PAA concentration increased, the SDZ degradation efficiency was improved, with its time-based pseudo-first-order rate constant (*k*_obs_) rising from 0.0227 min^−1^ to 0.0536 min^−1^. Similarly, in the UV_222_-Br system, the degradation efficiency of SDZ also increased from 20.17% to 92.44% (Fig. 1b). This is because under 222 nm light excitation, Br^−^ underwent photoactivation mediated by hydrated clusters to generate •Br. This radical could directly attack the aniline group in SDZ and subsequently react with Br^−^ to form a highly reactive dibromine radical anion (•Br_2_^−^), which synergistically degraded SDZ^22^. Thus, the results indicated that UV_222_ can efficiently activate PAA and Br^−^ to produce active substances for SDZ degradation. In particular, the degradation efficiency of SDZ was greatly increased by the coupled system of PAA and Br^−^, reaching an elimination rate of more than 99.99% in just 20 minutes (Fig. 1c)^15^. In UV_222_-PAA-Br (UV_222_-PB) system, the *k*_obs_ value of SDZ (0.2190 min^−1^) was 2.86 times the sum of *k*_obs_ values for UV_222_-PAA (0.0410 min^−1^) and UV_222_-Br (0.0356 min^−1^), indicating a pronounced synergistic interaction between PAA and Br^−^ rather than a mere additive effect, which significantly enhanced the degradation of SDZ (Fig. 1d and Supplementary Fig. 2a−c). Notably, the Dark-PB system under dark conditions achieved 53.96% removal of SDZ within 30 minutes (Supplementary Fig. 1), which can be attributed to the reaction between PAA and Br^−^ to generate HOBr (*k* = 0.24 M^−1^·s^−1^), leading to the oxidative degradation of SDZ^24^. However, the *k*_obs_ value of Dark-PB (0.0251 min^−1^) was only 0.11 times that of UV_222_-PB, indicating that under far-UVC irradiation, the interaction between PAA and Br^−^ may trigger extended radical chain reactions, thereby increasing the amount of reactive species in the system and enhancing the degradation efficiency (see synergistic interaction mechanism for more information). Furthermore, the results of relative contribution quantification analysis indicated that oxidation degradation mediated by reactive species accounted for as much as 95.94% in the UV_222_-PB system, which was 23.63 times higher than direct photolysis (Supplementary Fig. 2d). This significant disparity suggested that the degradation of SDZ in this system primarily relied on the oxidative action of photoreactive species rather than direct photolysis.

Given the inevitable presence of H_2_O_2_ in PAA solutions, this study evaluated the potential impact of H_2_O_2_ on the UV_222_-PB system. Based on previous reports and experimental measurements, this study selected a concentration of 200 μM H_2_O_2_ for irradiation over the same time range (Supplementary Fig. 3a−b)^8^. The relative effect of H_2_O_2_ can be assessed by the ratio of rate constants. The *k*_obs_ values for SDZ degradation in the UV_222_-PB and UV_222_-HB systems were 0.2190 min^−1^ and 0.0796 min^−1^, respectively, with a ratio of 2.75, indicating that SDZ degradation is primarily attributable to PAA rather than H_2_O_2_^15^. To further investigate the role of H_2_O_2_, exogenous H_2_O_2_ at different concentrations was introduced into the UV_222_-PB system (Supplementary Fig. 3c−e). As the concentration of added H_2_O_2_ increased, the degradation rate of SDZ showed a gradual decrease. When the total H_2_O_2_ concentration reached four times the PAA concentration, the *k*_obs_ value of SDZ was only 0.0640 min^−1^. This inhibitory effect can be attributed to the light-shielding effect caused by high H_2_O_2_ concentrations, which weakens the effective photoactivation of PAA and Br^−^ in the solution^28^. At the same time, excess H_2_O_2_ scavenges reactive species such as HOBr from the system, hindering the oxidative degradation of pollutants^29,30^. On the other hand, 20 units·mL^−1^ of catalase (CAT) was added to remove the coexisting H_2_O_2_ from the solution (Supplementary Fig. 3f−h). Unexpectedly, compared to the Dark-PB system (0.0251 min^−1^), the Dark-PB with CAT system (0.1077 min^−1^) exhibited a 3.29-fold increase in the rate constant for SDZ degradation. This is because HOBr, generated by the reaction of PAA with Br^−^, can be reduced by coexisting H_2_O_2_, but when CAT is introduced into the reaction solution, H_2_O_2_ is removed, blocking the reduction pathway of HOBr and thereby unleashing the oxidative potential of HOBr. Similarly, CAT alone had almost no effect on promoting the degradation of SDZ under UV_222_ irradiation. However, when CAT was added, the degradation rate of SDZ in the UV_222_-PB system increased by 40.96%. Since CAT can decompose H_2_O_2_ into O_2_ and H_2_O, we measured the changes in dissolved oxygen (DO) in the UV_222_-PB system with and without CAT. Evidently, upon the addition of CAT, the initial DO concentration in the reaction solution rose from 8.24 mg·L^−1^ to 16.50 mg·L^−1^, and as the reaction proceeded, the DO concentration in the solution gradually decreased (Supplementary Fig. 3i). Previous studies have shown that an increase in the DO concentration in the solution can effectively drive the chain reaction initiated by the UV_222_ photolysis of O_2_, promoting the generation of reactive oxygen species such as •OH, which in turn enhances the system’s oxidative degradation of OMPs^11^.

Furthermore, four typical aquatic environmental OMPs with significantly different chemical structures and functional groups, namely sulfamethazine (SMZ), ciprofloxacin (CIP), CBZ, and tetracycline hydrochloride (TC), were also selected to evaluate the synergistic degradation effect of the UV_222_-PB process (Fig. 1e and Supplementary Fig. 4). The results showed that upon the introduction of Br^−^, the *k*_obs_ values of SMZ and TC increased to 0.0210 min^−1^ and 0.0374 min^−1^, respectively, due to the presence of electron-rich fused rings and aniline groups in their structures, which exhibit high reactivity with bromine radicals^31^. Meanwhile, in the UV_222_-PAA system, the degradation efficiencies of SMZ (0.0325 min^−1^), CIP (0.0223 min^−1^), CBZ (0.0095 min^−1^), and TC (0.0609 min^−1^) were all enhanced to varying degrees. Notably, compared with direct photolysis, the degradation of all four OMPs was significantly improved in the UV_222_-PB process. Particularly for TC, its *k*_obs_ value increased from 0.0108 min^−1^ to 1.1028 min^−1^, providing strong evidence that photoreactive species play a dominant role in pollutant degradation in the UV_222_-PB process. Specifically, the *k*_obs_ values of SMZ, CIP, CBZ, and TC in the UV_222_-PB process were 4.90, 3.25, 1.95, and 11.22 times the sum of the *k*_obs_ values of the UV_222_-PAA and UV_222_-Br processes, respectively (Supplementary Table 2). This further confirms the significant synergistic effect between PAA and Br^−^ under UV_222_ irradiation, demonstrating that the UV_222_-PB process can achieve effective degradation of various OMPs.

In addition, this study also examined the removal effectiveness of SDZ under UV_222_ and UV_254_ across various systems. Figure 1f and Supplementary Fig. 5 depict the fluence-based pseudo-first-order rate constant (*k*′_obs_) for SDZ degradation in these systems. Under identical UV light doses, the UV_222_-PB system (*k*′_obs_ = 0.0159 cm^2^·mJ^−1^) exhibited significantly superior degradation efficacy for SDZ compared to the UV_254_-PB system (*k*′_obs_ = 0.0053 cm^2^·mJ^−1^). To elucidate the degradation differences of SDZ under different UV wavelengths during the UV-PB process, the ε of PAA and Br^−^ at pH 7.0 was calculated (Texts S3–S4 and Supplementary Fig. 6). The results indicated that ε_222_ values of PAA and Br^−^ were determined to be 284 M^−1^·cm^−1^ and 78 M^−1^·cm^−1^, respectively, representing 6.45 times and 39 times the value at 254 nm (Supplementary Table 3). The order-of-magnitude difference caused PAA and Br^−^ to generate more reactive species at 222 nm and exhibit significantly higher catalytic activity. On the other hand, the ε and Φ values of SDZ at 222 nm were lower than those at 254 nm, resulting in more significant degradation of SDZ under UV_254_ during direct photolysis (Supplementary Fig. 5h). To further explore the mechanism of Br^−^ at different wavelengths, the study calculated the excited-state properties of Br^−^ and SDZ using time‑dependent density functional theory (TDDFT). The results show that the effective excitation wavelength of Br^−^(S_1_) is 207.07 nm (5.99 eV), while the photon energies at 222 nm (5.58 eV) and 254 nm (4.88 eV) are both lower than this value; therefore, it is difficult to directly photoexcite Br^−^ to generate reactive bromine species (Supplementary Fig. 7a and Supplementary Table 4). In contrast, the calculation results for SDZ indicate that light with a wavelength shorter than 264.92 nm can excite it to an excited state. Thus, both 222 nm and 254 nm can effectively excite SDZ (Supplementary Fig. 7b and Supplementary Table 5). According to previous reports, electron-rich pollutants can act as potential direct electron donors^32^. Therefore, the study further calculated the interaction between SDZ and Br^−^ at different adsorption sites (Supplementary Fig. 7c−e and Supplementary Tables 6–8). Obviously, when the complexation site of Br^−^ with SDZ is located on the benzene ring or pyrimidine ring of SDZ, photoexcitation can induce an electron transition from the highest occupied molecular orbital (HOMO) to the lowest unoccupied molecular orbital (LUMO), accompanied by charge transfer from Br^−^ to SDZ (Supplementary Fig. 7g). Furthermore, free energy calculations indicate that ground‑state SDZ (S_0_) cannot spontaneously react with Br^−^ to form •Br (4.02 eV), whereas the free energy change for the reaction between excited‑state SDZ (S_1_) and Br^−^ is negative (−0.0055 eV), which is thermodynamically favorable (Supplementary Fig. 7f). This suggests that both 222 nm and 254 nm light excitation can drive electron transfer between SDZ and Br^−^, thereby effectively generating •Br. In addition, 365 nm, another important emission wavelength of the mercury lamp, was also included in the discussion (Text S5).

Additionally, the economic feasibility was determined by calculating the EE/O for various systems (Fig. 1g and Text S6)^33^. Under UV irradiation alone, because SDZ exhibits stronger light absorption at 254 nm, the EE/O value at 254 nm was lower than that at 222 nm. However, in other reaction systems, the EE/O values under UV_222_ irradiation are generally lower than those under the corresponding UV_254_ irradiation conditions. Among these, the UV_222_-PB system exhibited the lowest EE/O value at 0.43 kW·h·L^−1^, which was only 0.32 times that of UV_254_-PB (1.34 kW·h·L^−1^). It was mainly due to the enhanced contaminant degradation kinetics and energy conversion efficiency of the UV_222_-PB system. In conclusion, the UV_222_-PB process provides considerable economic benefits and lower energy consumption than competing systems.

### Identification and function of active substances

**Fig. 2:**
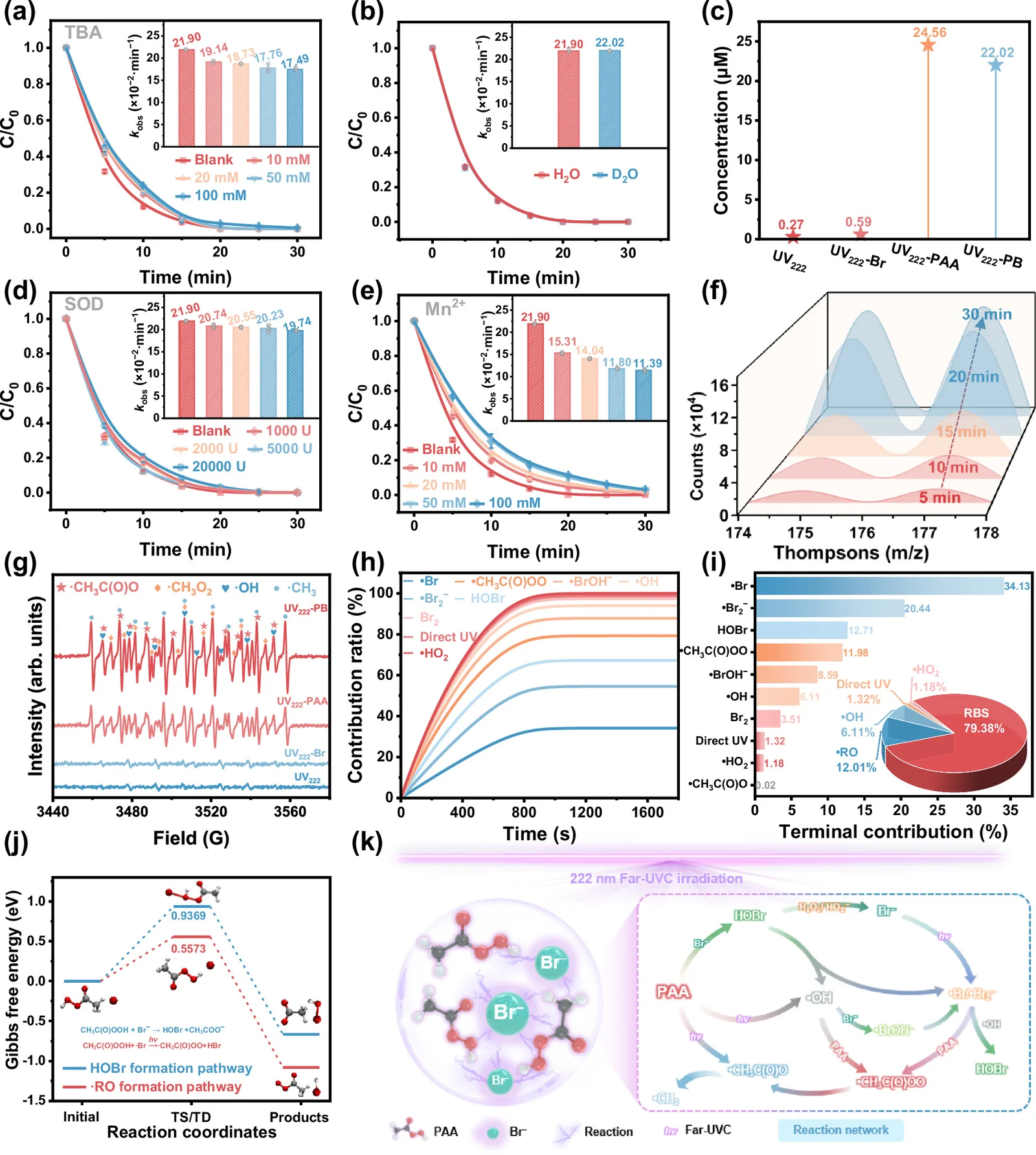
Identification of active species and their interaction mechanisms. Sulfadiazine (SDZ) degradation curves and pseudo-first-order rate constant (*k*_obs_) values in the UV_222_-PB system at different (**a**) tert-butanol (TBA) concentrations and (**b**) solvents. **c** Hydroxyl radical concentrations generated in different UV_222_ treatment procedures. Degradation of SDZ using the UV_222_-PB process at different (**d**) superoxide dismutase (SOD) and (**e**) Mn^2+^ concentrations. The inset shows the rate constant derived from the pseudo-first-order kinetic fitting. **f** Concentration of 4-Bromo-3,5-dimethyl-1H-pyrazole (4-Br-DMPZ) at different time points during the UV_222_-PB process. The arrow indicates that the mass spectrometry peak intensity of 4-Br-DMPZ gradually increases with reaction time. **g** Electron spin resonance (ESR) spectra obtained by using 5-Diisopropoxyphosphoryl-5-methyl-1-pyrroline N-oxide (DIPPMPO) as a spin-trapping agent in different processes. In the UV_222_-PB model, (**h**) the contributions of various active substances to SDZ degradation and **i** the terminal contribution. The inset summarizes the terminal contributions and categorized proportions of each reactive species (RBS: reactive bromine species). **j** Gibbs free energy change diagram for the reaction between HOBr and •CH_3_C(O)OO (TS: transition state, TD: excited state). **k** Radical chain reaction network and bromine cycling mechanism in the UV_222_-PB system. The left panel illustrates the photochemical microenvironment of PAA and Br^−^ under Far-UVC irradiation. The right panel (enclosed by a dashed box) depicts the detailed radical chain reaction network, elucidating the generation and transformation pathways of the reactive species. Conditions: [SDZ]_0_ = 20 μM, pH = 7.0, [PAA]_0_ = [Br]_0_ = 0.5 mM. Error bars represent SD of three replicate experiments, and the central value is the mean of the data. Data are presented as mean values ± SD. Source data are provided as a Source Data file.

To elucidate the primary reactive oxygen species (ROS) generated during the degradation of organic pollutants by the UV_222_-PB system, this study employed quenching experiments using different concentration gradients of quenchers. Preceding the quenching experiments, interference control assessments were conducted for quenchers including tert-butanol (TBA), furfuryl alcohol (FFA), and superoxide dismutase (SOD) (Supplementary Fig. 8a−b). The results indicated that the addition of FFA significantly inhibits the degradation of SDZ. This was attributed to the strong light absorption of FFA at 222 nm, which competed for photons within the system and thereby interfered with the photoactivation process^16^. Consequently, FFA was unsuitable for use as a quencher in this study. In contrast, other quenchers exhibited minimal impact on the experimental outcomes and represented ideal choices for the experiments. Evidently, as a •OH scavenger (*k* = 3.8 × 10^8 ^M^−1^·s^−1^), TBA addition to the UV_222_-PB system only slightly inhibited SDZ removal^34,35^. When TBA concentration was 100 mM, the SDZ elimination rate could still reach 99.45% within 30 minutes, indicating that •OH was not the primary active species in the UV_222_-PB system (Fig. 2a and Supplementary Fig. 8c). To verify the contribution of ^1^O_2_, we replaced the solvent in the reaction system from H_2_O to deuterium oxide (D_2_O). Surprisingly, despite the fact that the lifetime of ^1^O_2_ in H_2_O (3.7 ± 0.4 μs) was only 0.054 times that in D_2_O (68 ± 1 μs)^36,37^, solvent exchange did not improve SDZ removal efficiency. The reaction rate constant of UV_222_-PB system was substantially comparable in D_2_O (*k* = 0.2202 min^−1^) and H_2_O (k = 0.2190 min^−1^), which indicates that the relatively low concentration of ^1^O_2_ in the system is not the primary active species responsible for the oxidative degradation of pollutants (Fig. 2b and Supplementary Fig. 8d). On the other hand, in SOD quenching studies with varied concentration gradients, SDZ degradation remained largely unaffected, demonstrating that •O_2_^−^ contributed minimally to the system (Fig. 2d and Supplementary Fig. 8e).

In addition, the formation of •OH (*k* = 5.9 × 10^9 ^M^−1^·s^−1^) and •O_2_^−^ (5.88 × 10^4 ^M^−1^·s^−1^) was further detected by introducing benzoic acid (BA) and nitrotetrazolium blue chloride (NBT) as probe molecules into the system^30,38^. As indicated, the concentration of BA remained nearly constant in UV_222_ and UV_222_-Br systems, but it reduced by 36.10% and 37.65% in the UV_222_-PAA and UV_222_-PB systems, respectively (Supplementary Fig. 9a). This indicated that far-UVC activation of PAA leading to O−O bond cleavage and H_2_O_2_ decomposition was the primary pathway for •OH generation in the system (Eqs. 1, 2). Notably, although the consumption of BA in the UV_222_-PB system increased compared to the UV_222_-PAA system, the formation of p-hydroxybenzoic acid (p-HBA) decreased. This was due to the competitive consumption of •OH by Br^−^ and •Br_2_^−^ in the solution (Eqs. 4 and 7), which consequently leads to a reduction in the formation of p-HBA (Fig. 2c and Supplementary Fig. 9b). In addition, the blue formazan formed by reduction of NBT by •O_2_^−^ has a significant UV-visible absorption peak at 560 nm^39^. However, when introducing NBT into the three distinct systems, no significant changes were seen at the maximum absorption peak of 560 nm (Supplementary Fig. 10), showing that •O_2_^−^ was almost absent in the aforementioned reaction systems.

1 $${{\rm{CH}}}_{3}{{\rm{C}}}({{\rm{O}}}){{\rm{OOH}}}+{{\rm{hv}}}\to \bullet {{\rm{OH}}}+\bullet {{\rm{CH}}}_{3}{{\rm{C}}}({{\rm{O}}}){{\rm{O}}}$$

2 $${{\rm{H}}}_{2}{{\rm{O}}}_{2}+{{\rm{hv}}}\to 2\bullet {{\rm{OH}}}$$

Moreover, to further investigate the formation of ROS in UV_222_-PB system, the study employed electron spin resonance (ESR) testing. Obviously, after adding DIPPMPO as a spin trapping agent, the ESR spectrum exhibited the characteristic eight-line spectrum of DIPPMPO-•OH (Fig. 2g). Compared to UV_222_ and UV_222_-Br systems, the •OH signals in the UV_222_-PAA and UV_222_-PB systems were substantially more intense, which was consistent with the experimental results of the aforementioned hydroxyl probes. In contrast, no distinct characteristic signal peaks for TEMP-^1^O_2_ and DMPO-•O_2_^−^ were observed in any of the four systems depicted in Supplementary Fig. 11. This finding further confirmed that the amounts of ^1^O_2_ and •O_2_^−^ generated within the reaction systems were negligible.

According to previous research reports, Br^−^ can be transformed into reactive bromine species (RBS, such as free HOBr or bromine radicals •Br, •Br_2_^−^, etc.), through various oxidation processes or far-UVC irradiation (Eqs. 3–8)^22^. Furthermore, PAA can directly react with Br^−^ to produce highly reactive HOBr (${{\mbox{E}}}_{{\mbox{HOBr}}/{\mbox{Br}}}^{0}$ = 1.59 V), effectively eliminating contaminants^23,40^. However, as a highly efficient broad-spectrum oxidant, the strong electrophilicity of HOBr, while enabling effective degradation, also serves as a key precursor for the formation of brominated disinfection byproducts (Br-DBPs) and the potential carcinogen bromate. Therefore, verifying the generation of HOBr was crucial for elucidating bromine transformation pathways and assessing byproduct risks. To confirm the production of free HOBr in the UV_222_-PB system, quenching studies with (NH_4_)_2_SO_4_ were performed (Supplementary Fig. 12). Results indicated that when (NH_4_)_2_SO_4_ was added at concentrations ranging from 10 to 100 mM, the removal rate of SDZ decreased from 92.48% to 84.16%, indicating that the UV_222_-PB system successfully generated HOBr, which contributed to the degradation of SDZ.

Furthermore, this work validated the production of HOBr in the UV_222_-PB system using 3,5-dimethyl-1H-pyrazole (DMPZ) trapping and LC-MS analysis^41^. DMPZ, acting as a probe, may quantitatively (1:1) and rapidly capture HOBr, converting it to 4-Bromo-3,5-dimethyl-1H-pyrazole (4-Br-DMPZ)^42^. Evidently, the mass spectrometry revealed characteristic peaks for 4-Br-DMPZ at mass-to-charge ratios (m/z) of 174.9/176.9 ([M + H]⁺). This indicated that the HOBr generated by the UV_222_-PB system was successfully captured by DMPZ (Fig. 2f and Supplementary Fig. 13). Interestingly, ion chromatography measurements revealed the concentration changes of Br^−^ at different time points. The results indicated that the initial concentration of Br^−^ was nearly identical to its final concentration, showing no significant fluctuations throughout the reaction process and remaining at a relatively stable level (Supplementary Fig. 14). In addition, the study monitored changes in the concentrations of bromate (BrO_3_^−^) and total organic bromine (TOBr) during the reaction (Supplementary Figs. 14c, 23). The results indicate that virtually no BrO_3_^−^ was generated in the UV_222_-PB system, with its concentration remaining below the detection limit. Meanwhile, the concentration of TOBr was also low, accounting for only about 1% of the total bromine, and it began to decrease gradually as the reaction progressed. This phenomenon suggested that Br^−^ in this system was not continuously converted as a consumable reactant but instead participated in a dynamic Br cycle process. During this process, Br^−^ is hardly consumed and is rarely converted into inorganic or organic bromine compounds. Specifically, PAA can react with Br^−^ to form HOBr. Concurrently, H_2_O_2_ present in the system and its derived reactive oxygen species (e.g., HO_2_^−^) can reduce HOBr back to Br^−^ (Eqs. 9–11). Consequently, within the UV_222_-PB system, Br^−^ functioned as a catalytic cycling medium, enabling the continuous regeneration and transformation of bromine species^43^. This drove radical-based chain reactions, thereby achieving highly efficient degradation of organic pollutants.

3 $${{\rm{Br}}}^{-}({{\rm{H}}}_{2}{{\rm{O}}}){{\rm{n}}}+{{\rm{hv}}}\to \bullet {{\rm{Br}}}+{{{\rm{e}}}_{{\rm{g}}}}^{-}+{{\rm{n}}}{{\rm{H}}}_{2}{{\rm{O}}}$$

4 $$\bullet {{\rm{OH}}}+{{\rm{Br}}}^{-}\to \bullet {{\rm{Br}}}{{{\rm{OH}}}}^{-}$$

5 $$\bullet {{\rm{Br}}}{{{\rm{OH}}}}^{-}+{{{\rm{Br}}}}^{-}\to \bullet {{{\rm{Br}}}_{2}}^{-}+{{\rm{OH}}}^{-}$$

6 $${{\bullet }}{{\rm{Br}}}+{{\rm{Br}}}^{-}\to {{\bullet }}{{{\rm{Br}}}_{2}}^{-}$$

7 $$\bullet {{{\rm{Br}}}_{2}}^{-}+\bullet {{\rm{OH}}}\to {{\rm{HOBr}}}+{{\rm{Br}}}^{-}$$

8 $${{\rm{CH}}}_{3}{{\rm{C}}}({{\rm{O}}}){{\rm{OOH}}}+{{\rm{Br}}}^{-}\to {{\rm{HOBr}}}+{{\rm{CH}}}_{3}{{\rm{COO}}}^{-}+{{\rm{H}}}_{2}{{\rm{O}}}$$

9 $${{\rm{H}}}_{2}{{\rm{O}}}_{2}\to {{{\rm{HO}}}_{2}}^{-}+{{\rm{H}}}^{+}$$

10 $${{\rm{HOBr}}}+{{\rm{H}}}_{2}{{\rm{O}}}_{2}\to {{\rm{Br}}}^{-}+{{\rm{O}}}_{2}+{{\rm{H}}}_{2}{{\rm{O}}}$$

11 $${{\rm{HOBr}}}+{{{\rm{HO}}}_{2}}^{-}\to {{\rm{Br}}}^{-}+{{\rm{O}}}_{2}+{{\rm{H}}}_{2}{{\rm{O}}}$$

As widely reported, reactive free radicals including •OH, organic radicals (•RO, such as methyl radical (•CH_3_), acetoxy radical (•CH_3_C(O)O), peroxy radicals (•CH_3_C(O)OO), and methyl peroxy radicals (•CH_3_O_2_)) are produced in PAA-driven advanced oxidation reactions, which can quickly attack contaminants and trigger their oxidative breakdown^44^. Notably, these radicals undergo dynamic interconversion through a series of complex chain reaction mechanisms, thereby collectively sustaining the system’s oxidative capacity. The diene structure present in the commonly used radical scavenger 2,4-HD can effectively quench •OH and •RO. However, due to its strong light absorption at 222 nm, it led to a significant light-shielding effect (Supplementary Fig. 8b). Therefore, we investigated Mn^2+^ (*k* ≈ 0.5 × 10^6^–1.6 × 10^6 ^M^−1^·s^−1^), which exhibits highly selective quenching effects on acylperoxyl radicals^45,46^. Fortunately, when 100 mM Mn^2+^ was separately incubated with SDZ, PAA, and Br^−^ in the dark for 30 min, no significant concentration changes were observed (Supplementary Figs. 15–16), indicating that Mn^2+^ does not interact with the components in the system. UV absorption spectroscopy showed that Mn^2+^ exhibits weak light absorption at 222 nm (Supplementary Fig. 8b). When 100 mM Mn^2+^ solution was irradiated alone at UV_222_ for 30 min, no decrease in Mn^2+^ concentration was detected, suggesting that the impact of Mn^2+^ on the photolysis process is negligible. Finally, the addition of Mn^2+^ to the UV_222_-PB system in the absence of pollutants had almost no effect on the concentration changes of the various species (Supplementary Fig. 15a). Therefore, Mn^2+^ can serve as an effective quencher for acylperoxyl radicals in the UV_222_-PB process. Clearly, the addition of Mn^2+^ exerted a significant inhibitory effect on the degradation of SDZ. When its concentration exceeded 50 mM, the degradation rate constant dropped to 0.1180 min^−1^, indicating that •CH_3_C(O)OO played a dominant role in the degradation of SDZ in the UV_222_-PB system (Fig. 2e and Supplementary Fig. 15g)^47^. Additionally, the spin trapper DIPPMPO was employed to further identify the types of •RO radicals actually present in the system. Complex signals with multiple peaks were observed in the trapping spectrum, which were resolved and attributed to free radicals such as •OH, •CH_3_, •CH_3_C(O)O and •CH_3_O_2_ (Fig. 2g)^48^. Compared with UV_222_-PAA, the typical twelve-line spectrum signals from DIPPMPO-•CH_3_, DIPPMPO-•CH_3_C(O)O and DIPPMPO-•CH_3_O_2_ were significantly enhanced in the UV_222_-PB system. This phenomenon could be attributed to: (1) The •OH radical formed by HOBr breakdown facilitating the conversion of PAA to •CH_3_C(O)OO (Eqs. 12–13). (2) •Br in solution underwent a radical substitution reaction with PAA, yielding •CH_3_C(O)OO, which could be further transformed into other •RO (Eqs. 14–16). (3) HOBr reacted with H_2_O_2_ and HO_2_^−^ to generate O_2_, which could then interact with •CH_3_ to form •CH_3_O_2_ (Eq. 17). The above experimental results showed that PAA could work with Br^−^ to produce a high amount of •RO, which aided in SDZ breakdown.

12 $${{\rm{HOBr}}}+{{\rm{hv}}}\to {{\bullet}}{{\rm{Br}}}+{{\bullet}}{{\rm{OH}}}$$

13 $${{\rm{CH}}}_{3}{{\rm{C}}}({{\rm{O}}}){{\rm{OOH}}}+{{\bullet }}{{\rm{OH}}}\to {{\bullet }}{{\rm{CH}}}_{3}{{\rm{C}}}({{\rm{O}}}){{\rm{OO}}}+{{\rm{H}}}_{2}{{\rm{O}}}$$

14 $${{\rm{CH}}}_{3}{{\rm{C}}}({{\rm{O}}}){{\rm{OOH}}}+{{\bullet }}{{\rm{Br}}}\to {{\bullet }}{{\rm{CH}}}_{3}{{\rm{C}}}({{\rm{O}}}){{\rm{OO}}}+{{\rm{HBr}}}$$

15 $$2{{\bullet }}{{\rm{CH}}}_{3}{{\rm{C}}}({{\rm{O}}}){{\rm{OO}}}\to 2{{\bullet }}{{\rm{CH}}}_{3}{{\rm{C}}}({{\rm{O}}}){{\rm{O}}}+{{\rm{O}}}_{2}$$

16 $${{\bullet }}{{\rm{CH}}}_{3}{{\rm{C}}}({{\rm{O}}}){{\rm{O}}}\to {{\bullet }}{{\rm{CH}}}_{3}+{{\rm{CO}}}_{2}$$

17 $${{\bullet }}{{\rm{CH}}}_{3}+{{\rm{O}}}_{2}\to {{\bullet }}{{\rm{CH}}}_{3}{{\rm{O}}}_{2}$$

### Combined thermodynamic and kinetic analysis of the UV_222_-PB mechanism

Based on the above analysis, it can be concluded that •RO and HOBr are the important active species responsible for the efficient degradation of SDZ in the UV_222_-PB system, with minor contributions from ^1^O_2_ and •O_2_^−^. Furthermore, DFT calculations offered an in-depth analysis of the reaction thermodynamics involved in the interaction of PAA with Br, clarifying the pathways and mechanisms of active species production in the UV_222_-PB process (Fig. 2j). PAA interacted with Br^−^ to produce HOBr via a nucleophilic substitution reaction^49^. As a nucleophile, Br^−^ attacked the O − O bond in PAA, overcoming an energy barrier of 0.9369 eV to form the transition state (TS), eventually converting into CH_3_C(O)O^−^ and HOBr. The occurrence of the aforementioned reaction was further supported by LC-MS data, as the creation of HOBr was substantially higher in the UV_222_-PB process than in the UV_222_-Br process (Fig. 2f and Supplementary Fig. 13). On the other hand, Zhao et al. found that when Br^−^ forms a water cluster complex with four water molecules, it can generate •Br under UV_222_ irradiation^22^. Meanwhile, HOBr can also undergo a decomposition reaction to produce •Br. The •Br in solution initiated radical substitution reactions with PAA via single-electron transfer, resulting in •CH_3_C(O)OO and HBr. Notably, in comparison to the HOBr generation process, organic radical reactions had a lower energy barrier (0.5573 eV) and a higher energy release, indicating that organic radical production was thermodynamically more advantageous.

A kinetic model was used to further quantify the contribution of each active substance to the degradation of SDZ in the UV_222_-PB process. The predicted values from the kinetic model were in good agreement with the experimental observations, demonstrating that the constructed kinetic model provides a relatively accurate description of SDZ degradation (Supplementary Fig. 17a−c). The model predicted the concentration changes of various active species and the instantaneous rates of the major pathways to further evaluate the dominant species in the SDZ transformation process (Supplementary Fig. 17d−f). Specifically, throughout the reaction, •Br was the dominant bromine species, consistently exhibiting the highest instantaneous rate. Terminal contribution analysis revealed that although the steady-state concentration of •Br was approximately four orders of magnitude lower than that of HOBr, its contribution to SDZ transformation was substantially higher (Fig. 2h−i). This is because the reaction rate of HOBr (1.78 × 10^5 ^M^−1^·s^−1^) with SDZ is less than one-hundred-thousandth that of •Br (4.28 × 10^10 ^M^−1^·s^−1^). On the other hand, in the •RO system, although the reaction rate of •CH_3_C(O)O with SDZ is slightly higher than that of •CH_3_C(O)OO, its resulting concentration is approximately four orders of magnitude lower than that of •CH_3_C(O)OO. Consequently, •CH_3_C(O)OO remained the dominant pathway for SDZ degradation. In summary, the major reactive species responsible for SDZ degradation in the UV_222_-PB process were •Br (34.13%), •Br_2_^−^ (20.44%), HOBr (12.71%), and •CH_3_C(O)OO (11.98%), while •OH contributed only 6.11%, and contributions from other reactive oxygen species were relatively minor.

Furthermore, the kinetic model effectively validated the Br^−^ cycling mechanism. To elucidate the primary source of •Br generation in the system, the study conducted a simulated blocking analysis by setting the rate constants for the photolysis of Br^−^ and HOBr, as well as the protonation and dehydration pathway of •BrOH^−^, to zero in the reaction network (Supplementary Fig. 17g). The results showed that when the HOBr photolysis pathway was deactivated, the simulated SDZ degradation curve exhibited only a minor change. In contrast, when the Br^−^ photolysis pathway was blocked, the fitted curve deviated significantly, indicating that the direct photolysis of Br^−^ is the main initial source of •Br. Furthermore, deactivation of the •BrOH^−^ protonation–dehydration pathway also resulted in a noticeable deviation of the degradation curve from the original fit, suggesting that •BrOH^−^ serves as an important regeneration channel for •Br during the chain reaction. To verify the robustness of the model predictions, a local sensitivity analysis was further conducted by perturbing the rate constants of each reaction channel by ±10% relative to the original simulation curve (Text S7, Supplementary Fig. 17h−k). The results clearly showed that the Br^−^ photolysis channel exhibited the highest sensitivity coefficient, being 1.63 and 4.48 times higher than those of the •BrOH^−^ and HOBr channels, respectively. Even minor fluctuations in its rate constant led to significant changes in the degradation curve, further confirming that Br^−^ photolysis is the critical initiation step of the bromine radical chain reaction, while •BrOH^−^ protonation serves as an important synergistic pathway for sustaining the chain cycle. The model fitting results indicated that the Br^−^ concentration remained almost unchanged during the UV_222_-PB process, and the concentrations of HOBr, •Br, and other species gradually approached steady state, suggesting that a dynamic equilibrium was established among the reactive bromine species, and Br^−^ accomplished a closed loop of oxidation, radical chain reactions, and regeneration. In addition, considering the crucial role of H_2_O_2_ in the Br^−^ cycling and regeneration process, the study tracked the concentration changes of PAA and H_2_O_2_ during the reaction using both experimental measurements and model predictions (Supplementary Fig. 17l). Obviously, the experimental values and model predictions agreed well over the entire time scale. At the end of the reaction, the consumption rates of PAA and H_2_O_2_ were approximately 45.19% and 36.90%, respectively. Given that the second-order reaction rate constant between H_2_O_2_ and HOBr is 1.50 × 10^4 ^M^−1^·s^−1^, and the residual concentration of H_2_O_2_ (0.1262 μM) is much higher than the steady-state concentration of HOBr, it is demonstrated that H_2_O_2_ in the system is sufficient to continuously reduce HOBr back to Br^−^, thereby ensuring the normal operation of the bromine catalytic cycle.

In summary, under far-UVC excitation, the UV_222_-PB system establishes an efficient degradation reaction network governed by photolysis initiation, radical proliferation, and a bromine catalytic cycle (Fig. 2k). Specifically, Br^−^ in the system can either be directly photolyzed under UV_222_ irradiation to generate highly reactive •Br via the dissociation of water-cluster complexes (Eq. 3), or act as a nucleophile to react with PAA via a substitution reaction to produce HOBr (Eq. 8). The generated HOBr can further undergo photolysis under continuous irradiation to yield •Br, accompanied by the release of •OH (Eq. 12). Subsequently, the produced •Br can both combine with Br^−^ to form •Br_2_^−^ (Eq. 6) and engage in radical chain substitution reactions with PAA via single-electron transfer, efficiently generating •CH_3_C(O)OO (Eq. 14). Meanwhile, PAA is photolytically activated to directly generate •OH and •CH_3_C(O)O (Eq. 1), while continuously proliferating •RO through radical hydrogen abstraction and chain propagation pathways, thereby ensuring the effective supply of •RO and •OH (Eqs. 13, 15—17). Notably, although the generated •OH can react with a portion of •Br to form •BrOH^−^, this intermediate can rapidly regenerate •Br via the protonation–dehydration pathway (Eq. 4). Furthermore, the coexisting H_2_O_2_ in the PAA solution and the HO_2_^−^ generated within the system efficiently achieve the in situ regeneration and closed-loop catalytic cycling of Br^−^, driven by their fast reaction kinetics with RBS such as HOBr and •Br (Eqs. 9–11). Consequently, the synergistic multi-species degradation and bromine catalytic cycle in the UV_222_-PB process enable both the rapid attack and sustained degradation of OMPs.

### Photodriven targeted oxidative degradation mechanism of the UV_222_-PB system

**Fig. 3:**
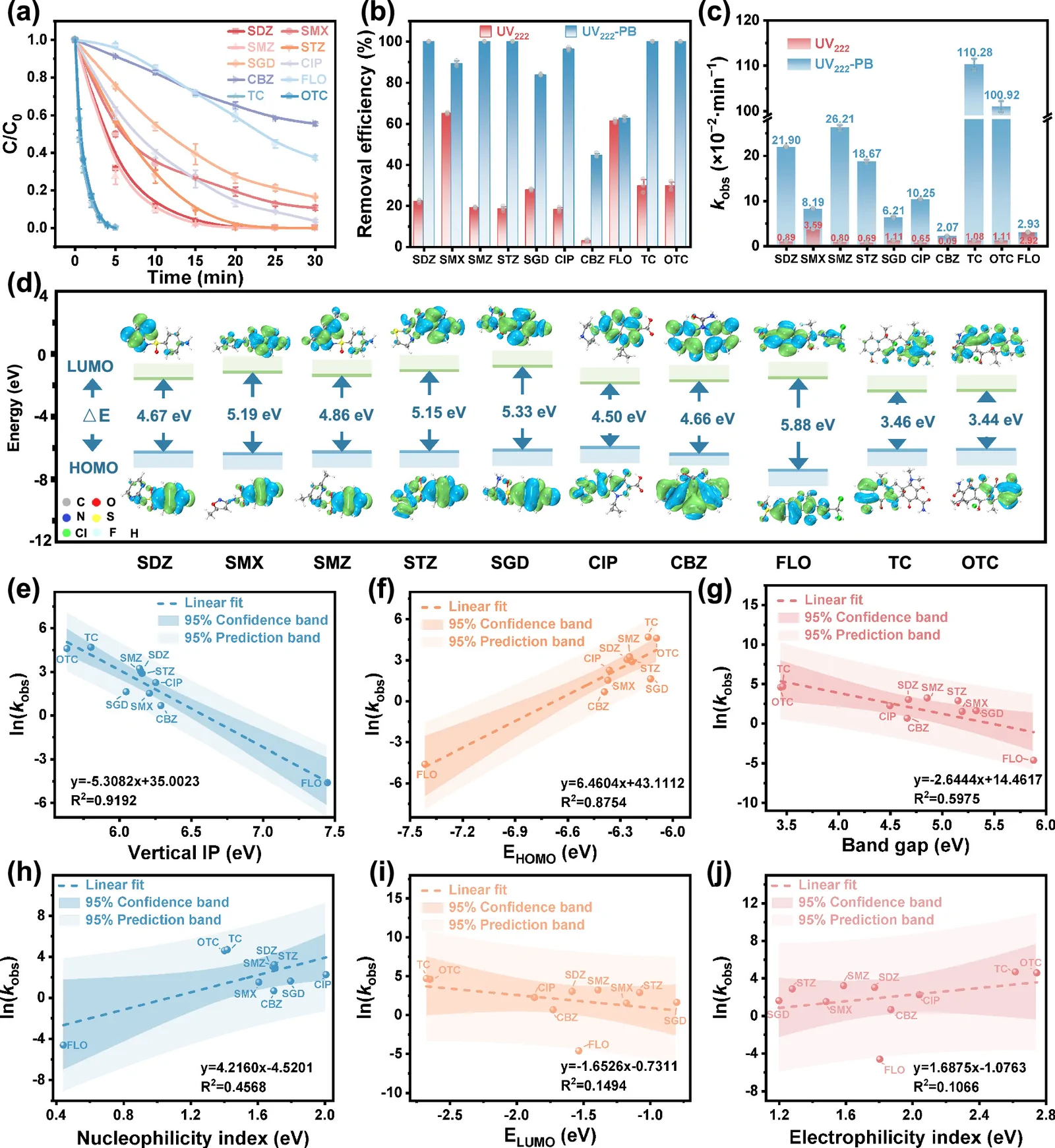
Targeted digestion of organic micropollutants (OMPs) and kinetic coupling of molecular parameters. **a** Degradation curve, (**b**) removal rate, and (**c**) pseudo-first-order rate constant (*k*_obs_) values of different OMPs by the UV_222_-PB process. The selected OMPs include sulfadiazine (SDZ), sulfamethoxazole (SMX), sulfamethazine (SMZ), sulfathiazole (STZ), sulfaguanidine (SGD), ciprofloxacin (CIP), carbamazepine (CBZ), tetracycline hydrochloride (TC), oxytetracycline hydrochloride (OTC), and florfenicol (FLO). **d** Comparison of the energy gaps between the highest occupied molecular orbital (HOMO) and the lowest unoccupied molecular orbital (LUMO) among different OMPs. The blue and green regions represent holes and electrons (isosurface set at 0.0200 a.u.), respectively, and Δ*E* is the energy gap between the HOMO and LUMO. Furthermore, linear regression analyses were performed between ln(*k*_obs_) of the different OMPs and their (**e**) Vertical IP, (**f**) Energy levels of HOMO (*E*_HOMO_), (**g**) Band gap, (**h**) Nucleophilicity indexes, (**i**) Energy levels of LUMO (*E*_LUMO_), and (**j**) Electrophilicity indexes. The dashed lines represent the linear fits based on the regression equations provided in the lower-left corner of each panel. The *R*^2^ values indicate the degree of correlation between ln(*k*_obs_) and the respective molecular descriptors. The dark shaded regions and light shaded regions represent the 95% Confidence bands and 95% Prediction bands of the linear fitting, respectively. Conditions: [OMPs]_0_ = 20 μM, pH = 7.0, [PAA]_0_ = [Br]_0_ = 0.5 mM. Error bars represent SD of three replicate experiments, and the central value is the mean of the data. Data are presented as mean values ± SD. Source data are provided as a Source Data file.

Based on the above analysis, it can be concluded that the reactive species produced by the UV_222_-PB system were mostly electrophilic (e.g., •RO, RBS). Therefore, sulfamethoxazole (SMX), SDZ, SMZ, sulfathiazole (STZ), sulfaguanidine (SGD), TC, oxytetracycline hydrochloride (OTC), florfenicol (FLO), CBZ, and CIP were selected as model pollutants. Simultaneously, theoretical calculations were employed to determine comprehensive electronic descriptors, including the HOMO and LUMO values, band gap (*E*_HOMO_-*E*_LUMO_), vertical ionization potential (vertical IP), Fukui functions, nucleophilic index (NI), electrophilic index (EI), and Mulliken electronegativity, to investigate potential relationships between model pollutant properties and degradation in the UV_222_-PB system (Supplementary Table 9). It can be observed that the UV_222_-PB system exhibited significant differences in the degradation of various pollutants. The degradation rate of electron-rich pollutants such as TC, OTC, and SDZ reached 100% in 30 minutes, with TC and OTC degrading almost completely within 5 minutes. But the degradation rate of electron-deficient pollutants FLO (62.61%) and CBZ (44.51%) fell dramatically (Fig. 3a−c and Supplementary Fig. 18a−c).

Since photolysis is essentially based on the interaction of photon energy and compound structure, the direct photolysis rate of pollutants is unaffected by electron-rich or electron-poor properties^50^. To elucidate the selective degradation mechanism of the UV_222_-PB system in detail, this study separated the *k*_obs_ value for the photolysis of UV_222_. Notably, when fitting the ln(*k*_obs_) values of ten separated model pollutants against different descriptors, the most significant correlations were observed for IP (*R*^2^ = 0.9192) and E_HOMO_ (*R*^2^ = 0.8754) (Fig. 3d−j). IP value is an important parameter to measure the redox ability of compounds. Specifically, pollutants with lower IP values have a larger electron cloud density, are more likely to lose electrons as reducing agents, and are more vulnerable to electrophilic active species^51^. Due to the presence of numerous electron-donating groups (−NH_2_ and −OH) in TC and OTC, coupled with the influence of their molecular conjugated systems, both exhibit low IP values and significantly higher degradation rates^52^. Similarly, the E_HOMO_ value is a molecular orbital descriptor used to measure the electron-donating ability of a compound; the higher the E_HOMO_ value, the greater the electron-donating capacity of molecule^49^. Contaminants with higher E_HOMO_ values (like TC, OTC, and SMZ) were more easily damaged by reactive species produced by the UV_222_-PB system, whereas FLO, with a lower E_HOMO_ value, experienced little oxidative degradation. However, the band gap (*R*^2^ = 0.5945) and NI (*R*^2^ = 0.4568) had little correlation with the value of ln(*k*_obs_), while the indices such as E_LUMO_ value, EI, and Mulliken electronegativity had little correlation with the value of ln(*k*_obs_) (Supplementary Fig. 18d, e). In general, the electrophilic active species produced by the UV_222_-PB system, such as RBS and •RO, had excellent selectivity for electron-rich contaminants, allowing for rapid oxidative degradation.

### Analysis of pollutant degradation pathways and systemic toxicity assessment

**Fig. 4:**
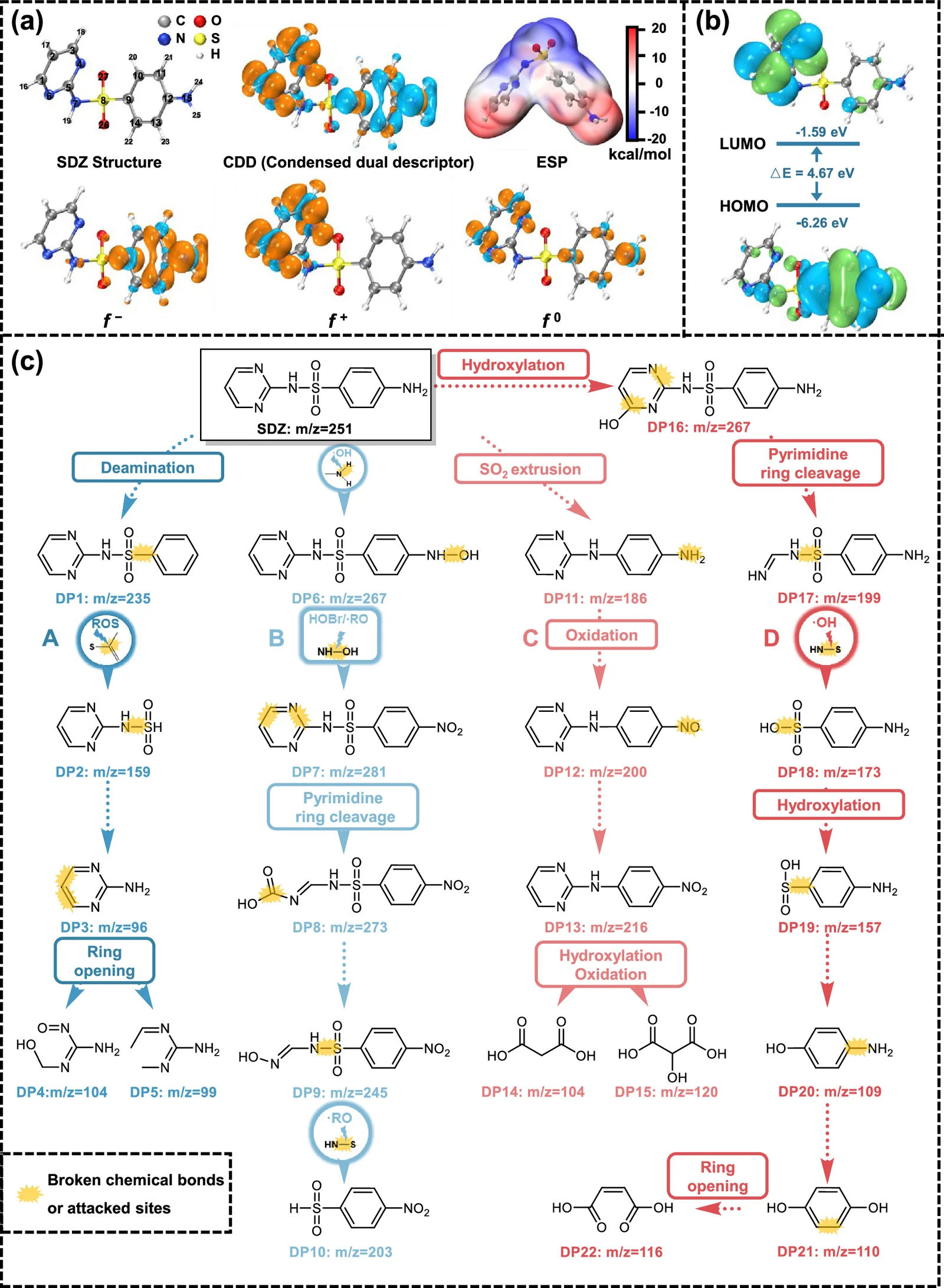
Quantitative analysis of degradation pathways. **a** Optimized molecular structure of sulfadiazine (SDZ), isosurface maps of the condensed dual descriptor (CDD) and electrostatic potential (ESP), as well as the Fukui reactivity functions (*f* ^*–*^*, f* ^*+*^*, f* ^*0*^). In general, *f* ^*–*^*, f* ^*+*^, and *f* ^*0*^ describe the susceptibility of SDZ to electrophilic, nucleophilic, and radical attacks, respectively. **b** Spatial distribution of the highest occupied molecular orbital (HOMO) and the lowest unoccupied molecular orbital (LUMO) in the SDZ. The blue and green regions represent holes and electrons (isosurface set at 0.0200 a.u.), respectively, and Δ *E* is the energy gap between the HOMO and LUMO. **c** Four degradation pathways of SDZ in the UV_222_-PB system (ROS: reactive oxygen species). The yellow markers indicate the predicted broken chemical bonds or attacked sites.

Furthermore, DFT simulations were employed to calculate key electronic structure parameters of SDZ, including the Fukui indices, HOMO, LUMO, electrostatic potential (ESP), bond order (BO), and bond length (BL), aiming to identify the potential reactive sites on the SDZ molecule. Combined with LC-MS analysis of the post-reaction solution, a more detailed investigation of the SDZ degradation pathways was conducted (Supplementary Tables 10, 11). Figure 4a illustrates the optimal molecular structure, Fukui indices, and electron density isosurface map of SDZ. Generally, *f* ^*–*^, *f* ^*+*^, and *f* ^*0*^ describe the probabilities of SDZ being attacked by electrophiles, nucleophiles, and radicals, respectively^53^. Reaction sites with higher Fukui indices show more reactivity^54^. Furthermore, the condensed dual descriptor (CDD) was introduced as an effective complementary tool to quantitatively assess the local electrophilic/nucleophilic sensitivity of SDZ. The CDD results indicated that regions with strongly negative values exhibit higher sensitivity to electrophilic attacks^55^. According to the HOMO and LUMO analyses, the benzene ring and amino group serve as the primary electron-donating sites, while the pyrimidine ring is the most prominent electron-accepting site (Fig. 4b). In addition, the bond strength of SDZ is positively correlated with the BO and negatively correlated with the BL. Consequently, chemical bonds such as N7–S8, S8–C9, and C9–C10 in the SDZ molecule are more susceptible to attack and cleavage by reactive species.

As shown in Fig. 4c and Supplementary Fig. 19, in Pathway A, the N15 site exhibits a high *f* ^*–*^ (0.1888) and a low CDD (-0.1856), indicating that the amino group at this position is prone to electrophilic attack. Concurrently, the reactive species in the system preferentially attack the C12–N15 bond due to its relatively low BO (0.733), leading to its cleavage^56^. This results in the deamination of the amino group on the SDZ benzene ring to generate DP1 (m/z = 235). Subsequently, ROS continuously attack the relatively weak S8–C9 (BO = 0.718, BL = 1.754 Å) and N7–S8 (BO = 0.563, BL = 1.682 Å) bonds, leading to the cleavage of the sulfonamide bridge to form DP2 (m/z = 159) and DP3 (m/z = 96). Finally, C1 (0.1693) and C3 (0.1682) on the pyrimidine ring, which possess higher CDD values, are attacked by nucleophiles to undergo ring-opening reactions, yielding small-molecule products DP4 (m/z = 104) and DP5 (m/z = 99). On the other hand, in Pathway B, owing to the strong electron-donating effect of the amino group on the benzene ring, it undergoes monohydroxylation under the attack of •OH to generate DP6 (m/z = 267). As DP6 is chemically unstable, it is susceptible to further deep oxidation by HOBr and •RO, transforming the amino group into -NO_2_ to form DP7 (m/z = 281). Subsequently, the pyrimidine ring undergoes cleavage to form DP8 (m/z = 273). The ester group in DP8 is then destroyed, leading to the generation of DP9 (m/z = 245). Further, under the action of highly reactive •RO, the N7–S8 bond in DP9 is cleaved to form DP10 (m/z = 203)^51^. In addition, because the pyrimidine ring region is affected by the electron-withdrawing effects of heteroatoms such as N and O, SDZ is also susceptible to electrophilic attack, causing SO_2_ to be expelled and the molecular structure to rearrange, thereby generating DP11 (m/z = 186) via pathway C. The benzene ring of this intermediate still retains an easily oxidizable amino group, providing active sites for subsequent deep oxidation. Therefore, under the action of reactive species such as HOBr and •RO, the -NH_2_ group on the benzene ring of DP11 undergoes a two-step consecutive oxidation. It is oxidized to the relatively unstable -NO group (DP12, m/z = 200), and then further oxidized to -NO_2_ (DP13, m/z = 216). Finally, DP13 undergoes hydroxylation and ring-opening reactions to yield smaller molecular weight products DP14 (m/z = 104) and DP15 (m/z = 120). In Pathway D, the higher *f* ^*0*^ values of C1 and C3 confer them greater radical reactivity, making them more prone to hydroxylation to form DP16 (m/z = 267)^57^. Furthermore, the presence of N4 and N6 leads to an uneven distribution of electron density on the pyrimidine ring. Under the combined effects of N4 and N6, C5 becomes more susceptible to attack, leading to the cleavage of the pyrimidine ring and the formation of DP17 (m/z = 199). Subsequently, the sulfonamide group undergoes hydrolysis under the attack of •OH to generate DP18 (m/z = 173). With further side-chain hydroxylation and deep ring-opening reactions, the DP18 structure progressively decomposes to form DP19 (m/z = 157), DP20 (m/z = 109), DP21 (m/z = 110), and DP22 (m/z = 116).

**Fig. 5:**
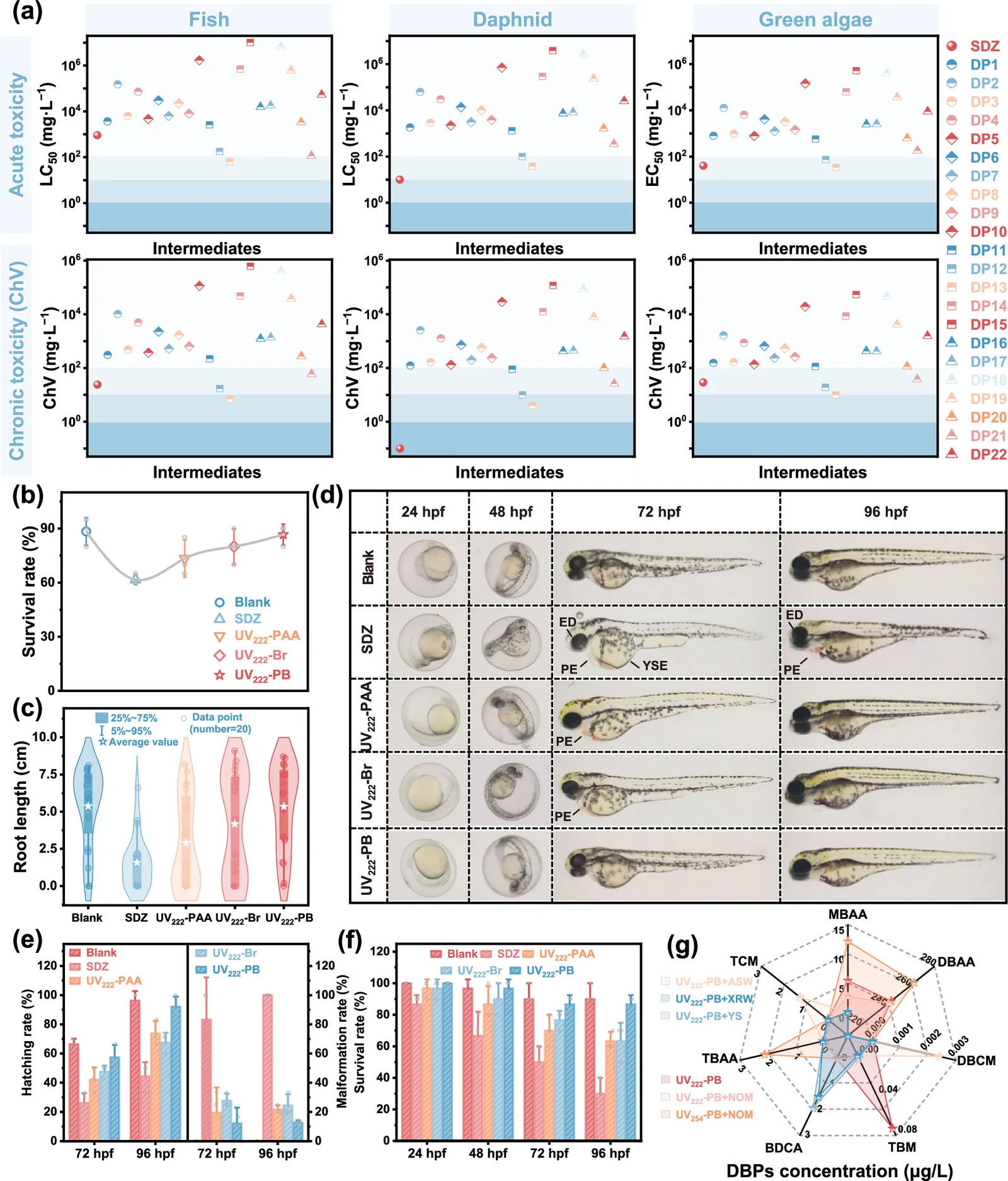
Multi-model toxicological effect assessment. **a** Acute and chronic toxicity of sulfadiazine (SDZ) and its intermediates. In different treatment conditions: (**b**) wheat germination rates, (**c**) violin diagram of wheat bud length distribution and zebrafish embryo (**d**) development (PE: pericardial edema, ED: eye deformation, YSE: yolk sac edema), (**e**) hatching rates, (**f**) survival rates. **g** The amount of disinfection byproducts (DBPs) produced in different processes (MBAA: monobrominated acetic acid, DBAA: dibrominated acetic acid, DBCM: dibromochloromethane, TBM: tribromomethane, BDCA: bromodichloroacetic acid, TBAA: tribromoacetic acid, TCM: trichloromethane). Error bars represent the SD of three replicate experiments, and the central value is the mean of the data. Data are presented as mean values ± SD. Source data are provided as a Source Data file.

It is critical to recognize that variations in the toxicity of SDZ and its degradation products cannot be ignored during the pollutant removal process. Thus, this study used the ECOSAR model, which is based on ecological structure-activity relationships, to predict the acute and chronic toxicity of SDZ and its breakdown products to fish, Daphnia, and green algae. As shown in Fig. 5a and Supplementary Table 12, SDZ exhibited varying degrees of toxicity toward different aquatic organisms, with the most pronounced chronic toxicity observed in Daphnia. Most intermediates had lower acute and chronic toxicity than the parent pollutant SDZ, indicating that UV_222_-PB system effectively reduced the ecological toxicity of pollutants. Surprisingly, DP13 in pathway C exhibited enhanced ecotoxicity toward fish and green algae, likely due to oxidative stress and cellular damage caused by the introduction of nitro groups^58^. Fortunately, through hydroxylation and ring-opening reactions, DP13 was ultimately converted into non-toxic small-molecule substances.

In addition, this research conducted further multidimensional toxicity assessments of the degradation products using plant and animal models, including wheat and amaranth seed germination experiments and zebrafish embryo cultures. The results of the zebrafish embryo culture experiment showed that, except for the blank control group, all treatment groups exhibited varying degrees of morphological abnormalities. Among them, the SDZ treatment group showed the highest malformation rate, reaching 100% at 96 h post-fertilization (hpf). Zebrafish exposed to the original SDZ solution began to show morphological malformations such as pericardial edema (PE), eye deformation (ED), and yolk sac edema (YSE) at 72 hpf (Fig. 5d), indicating that the SDZ solution has high animal toxicity. Zebrafish cultured in solutions treated with the UV_222_-PAA and UV_222_-Br processes also exhibited PE developmental abnormalities at 72 hpf. Compared with the original SDZ group, both the survival rate and hatching rate of zebrafish at 96 hpf were improved after treatment with the various UV_222_-based processes (Fig. 5e−f). Among them, the UV_222_-PB process exhibited the most significant detoxification effect, with its hatching rate at 96 hpf being only 4.17 % lower than that of the blank control group. Additionally, in wheat seed germination experiments, after cultivation in five different water samples-blank group, SDZ solution, UV_222_-PAA, UV_222_-Br, and UV_222_-PB the survival rates of wheat seeds were 88.33%, 61.67%, 73.33%, 80%, and 86.67%, respectively (Fig. 5b−c and Supplementary Fig. 20). Obviously, the growth of wheat seeds in SDZ solution (1.58 cm) was obviously inhibited, and the average bud length at 120 h was only 0.29 times that of the blank group (5.37 cm), while the average bud length of wheat treated with UV_222_-PB system (5.34 cm) was almost the same as that of the blank group. Similarly, in the germination experiment of amaranth seeds, although all five systems maintained a high survival rate, only UV_222_-PB showed the same rhizome length as the control group (Supplementary Figs. 21, 22). Therefore, from the aspects of model prediction and comprehensive evaluation of animals and plants, it was fully confirmed that the water samples treated by UV_222_-PB system were low in toxicity and harmless and had excellent practical application potential.

In the presence of oxidizing agents, the active compound HOBr can be further converted to bromate, resulting in the formation of Br-DBPs. Therefore, this study evaluated the formation of bromate in different systems. The results showed that no bromate was detected in any of the four systems, namely UV_254_-Br, UV_254_-PB, UV_222_-Br, and UV_222_-PB (Supplementary Fig. 23). This is because the generation concentration of •OH in these systems is limited, insufficient to further oxidize reactive bromine species to bromate^59^. At the same time, coexisting substances such as H_2_O_2_ reduce HOBr and other intermediates back to Br^−^, thereby blocking the bromate formation pathway. In addition, studies indicate that natural organic matter (NOM), as an important precursor of DBPs, may react with secondary free radicals with lower oxidation ability, such as •Br and •Cl to generate DBPs, which are toxic and harmful to the environment^60^. Therefore, this study examined the formation of DBPs under different UV radiation conditions, with or without Suwannee River III NOM (SRNOM), and in various natural water bodies. It was found that in the presence of 2 mg_C_·L^−1^ SRNOM, only the formation of monobrominated acetic acid (MBAA), dibrominated acetic acid (DBAA), and tribromomethane (TBM) was identified by GC-ECD (Fig. 5g and Supplementary Table 13). Notably, compared to UV_254_, the formation concentrations of MBAA (2.69 μg·L^−1^) and DBAA (242.89 μg·L^−1^) both decreased under UV_222_ irradiation. This was due to the significantly higher degradation rates of various DBPs under UV_222_ compared to UV_254_^61^. Besides, it was observed that the concentrations of the four Br-DBP products uniformly increased slightly in the absence of SRNOM. This is due to electron-rich sites such as the phenolic group in SRNOM that can effectively quench HOBr generated in the system, thereby reducing the yield of Br-DBPs. It is noteworthy that the UV_222_-PB process exhibited low concentrations of DBPs in real water matrices such as Xiangjiang River water (XRW), Yellow Sea (YS), and Aquaculture Seawater Wastewater (ASW). Specifically, Cl^−^ present in real water environments can react with PAA to generate HOCl, which is also an important precursor of DBPs^62^. However, compared with HOBr, HOCl has weaker electrophilicity and thus a lower potential to form DBPs^63^. Consequently, only low concentrations of chlorinated DBPs (Cl-DBPs), such as trichloromethane (TCM), dibromochloromethane (DBCM), and bromodichloroacetic acid (BDCA), were detected in real water matrices. On the other hand, components such as NOM, HCO_3_^−^, and nitrite in real water can competitively consume bromine species^64,65^. Furthermore, DOM in the solution can convert •Br and •Br_2_^−^ back to Br^−^ via single electron transfer^66^, thereby hindering the formation of RBS and subsequently suppressing the formation of Br-DBPs.

### Comprehensive evaluation of environmental stability of the UV_222_-PB system

**Fig. 6:**
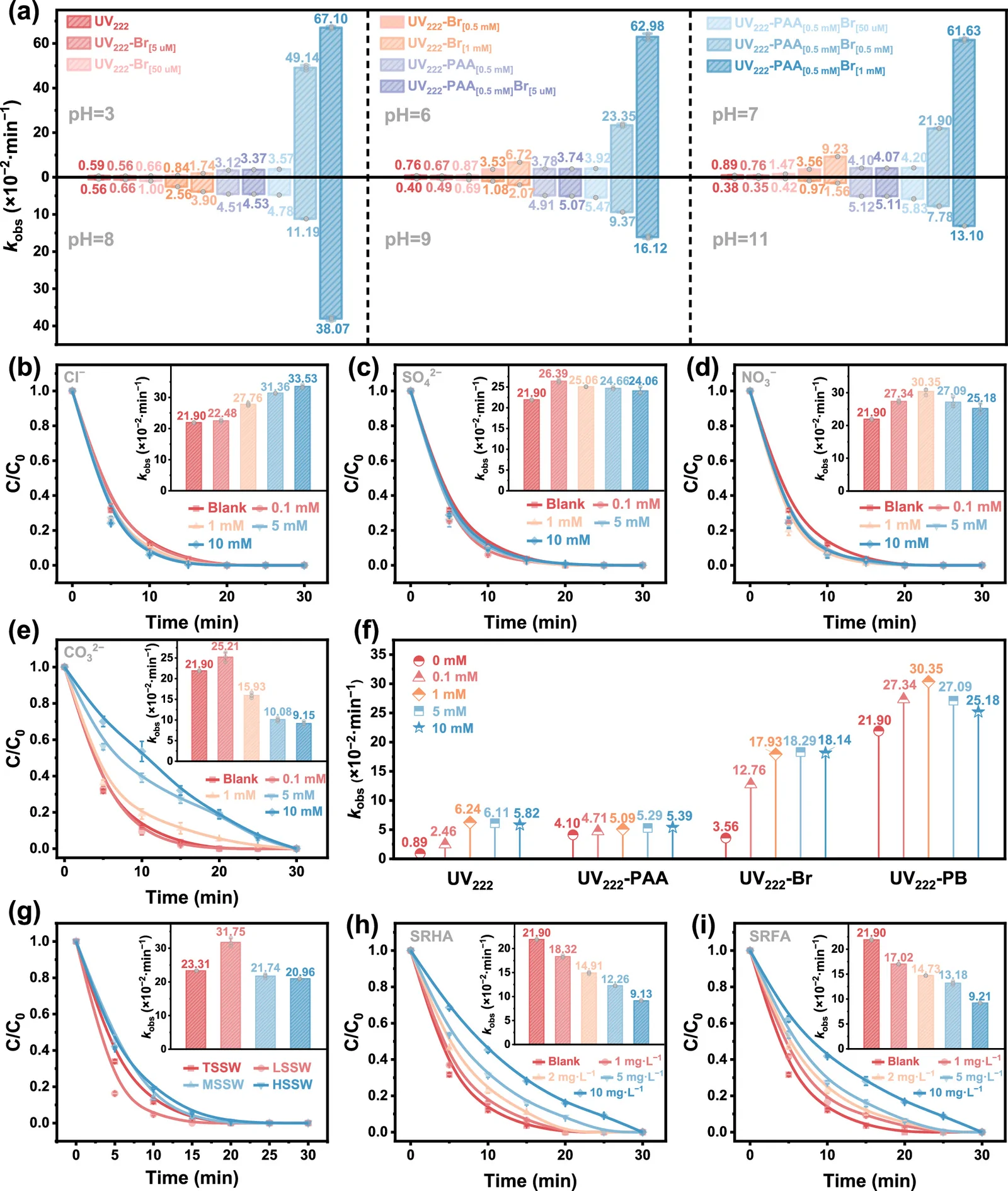
The regulatory effects and mechanisms of water quality parameters on SDZ degradation. **a** Pseudo-first-order rate constant (*k*_obs_) values of sulfadiazine (SDZ) in different processes at multiple pH levels. Degradation curves and *k*_obs_ values of SDZ degraded by UV_222_-PB in the presence of (**b**) Cl^−^, (**c**) SO_4_^2−^, (**d**) NO_3_^−^, (**e**) CO_3_^2−^. **f** The effect of coexisting NO_3_^−^ on the degradation of SDZ under UV_222_ using different processes. **g** The performance of UV_222_-PB in simulated water bodies with different salinities (TSSW: trace‑salinity simulated water, LSSW: low‑salinity simulated water, MSSW: medium‑salinity simulated water, HSSW: high‑salinity simulated water). Degradation curves and *k*_obs_ values of SDZ degraded by UV_222_-PB in the presence of (**h**) Suwannee River III HA (SRHA) and (**i**) Suwannee River III FA (SRFA). The inset shows the rate constant derived from the pseudo-first-order kinetic fitting. Conditions: [SDZ]_0_ = 20 μM, [PAA]_0_ = [Br]_0_ = 0.5 mM. Error bars represent SD of three replicate experiments, and the central value is the mean of the data. Data are presented as mean values ± SD. Source data are provided as a Source Data file.

The pH of solution is an important parameter that influences pollutant degradation and the production of reactive chemicals. Therefore, this study investigated the effect of the UV_222_-PB system on the degradation of SDZ under different pH conditions. It is evident that, in the UV_222_ system, SDZ exhibits the best degradation performance under neutral conditions (Supplementary Fig. 24). This is because SDZ, as an amphiprotic compound (pK_a1_ = 1.57, pK_a2_ = 6.5), exists in different forms depending on the pH of the solution. At pH 3, SDZ predominantly exists in its neutral form (SDZ^0^). As the pH increases further, the deprotonated form (SDZ^−^) gradually replaces SDZ^0^ as the dominant species (Supplementary Fig. 25). DFT calculations indicate that compared to SDZ^+^ (5.29 eV) and SDZ^−^ (4.82 eV), SDZ^0^ (4.67 eV) has a smaller HOMO-LUMO energy gap, thus requiring lower energy for excitation, which results in a faster reaction rate (Supplementary Fig. 26)^9^. On the other hand, in the UV_222_-Br system, increasing the Br^−^ concentration from 0 to 1 mM gradually enhances the degradation efficiency of SDZ. Interestingly, the UV_222_-Br process shows better degradation performance at pH 7. This phenomenon may be attributed to the reduced generation of •OH under acidic conditions (Supplementary Fig. 27), while alkaline conditions are unfavorable for the formation of HOBr^25^. Moreover, in the UV_222_-PB system, when the Br^−^ concentration was within 50 μM, the removal efficiency of PB for SDZ correlated positively with pH. When the Br^−^ concentration surpassed the threshold, an inverse pH-dependent relationship appeared (Fig. 6a and Supplementary Fig. 28). Under different pH values, the differences in SDZ degradation by the UV_222_-PB system are attributed to: (1) low Br^−^ concentration. PAA oxidation of Br^−^ results in limited HOBr, and SDZ elimination relies on the reactive species produced by PAA. However, the morphology of PAA (pK_a_ = 8.2) is influenced by pH. As the solution pH rises, deprotonated PAA (PAA^−^) increasingly dominates. The reaction rate constant of PAA^−^ (*k* = 3.25 × 10^−5^ s^−1^) activated by far-UVC in alkaline conditions was higher than that of PAA (*k* = 2.98 × 10^−5^ s^−1^) in acidic conditions, thus demonstrating superior removal efficiency of SDZ in alkaline environments^18^. (2) High Br^−^ concentration. The pH of the solution affects the species distribution of SDZ and HOBr (pK_a_ = 8.8)^67^. Since SDZ^−^ becomes the predominant species in alkaline conditions, electrostatic repulsion causes a decrease in the degradation rate. Furthermore, the oxidation potential of PAA^−^ (1.01 V vs. SHE) was weaker than that of PAA (1.75 V vs. SHE)^34^. Therefore, as the pH rose, the amount of HOBr formed by oxidation in the system gradually decreased. Meanwhile, under alkaline conditions, HOBr underwent self-decomposition to form BrO^−^, which exhibited weaker electrophilic and thus was less efficient in attacking the sulfonamide group of SDZ and the amino groups in its aniline ring^23^. Therefore, compared to acidic environments, the UV_222_-PB system with higher Br^−^ concentrations in alkaline circumstances exhibited significantly reduced degradation performance toward SDZ.

The presence of diverse inorganic anions in aquatic environments has an unavoidable impact on the UV_222_-PB system’s ability to oxidatively degrade contaminants. This work added inorganic anions (Cl^−^, NO_3_^−^, SO_4_^2−^ and CO_3_^2−^) at varying concentrations (0.1−10 mM) to the UV_222_-PB system to better simulate actual wastewater conditions. In short, the existence of Cl^−^, SO_4_^2−^, and NO_3_^−^ enhanced the performance of the UV_222_-PB system, whereas the introduction of higher concentrations of CO_3_^2−^ obviously inhibited the degradation of SDZ (Fig. 6b−e and Supplementary Fig. 29a−d). In particular, when 0.1−10 mM Cl^−^ was added to the system, the degradation of *k*_obs_ by SDZ was increased by 1.03−1.53 times compared with the blank group. The promotion of Cl^−^ can be attributed to: (1) Higher Cl^−^ concentrations enhancing Cl^−^ exposure surrounding PAA in the reaction system and generating a highly oxidizing weak acid, HOCl (Eq. 18), which can promote organic pollutant breakdown. HOCl rapidly attacks SDZ through targeted electrophilic reactions and synergistic interactions with other reactive species, causing it to rapidly oxidize and cleavage^34,68^. (2) Cl^−^ in the system combines with •OH to generate •Cl, which then converts into •Cl_2_^−^ (with lifetimes of tens of microseconds or even milliseconds) and other reactive chlorine species (RCS) (Eqs. 19−23)^53^. (3) The addition of Cl^−^ enables reaction with Br^−^ to create halogen species with higher reactivity (BrCl and BrOCl, Eqs. 24−25), thereby improving the conversion of SDZ^30^. Additionally, SO_4_^2−^ promotes the system by successfully consuming •OH radicals, resulting in •SO_4_^−^ radicals with increased oxidative capability and longer half-lives (Eq. 26)^53^. On the other hand, NO_3_^−^ exhibits strong absorption at 222 nm (*ε* = 2747 M^−1^·cm^−1^), and its photolysis generates •OH and reactive nitrogen species (RNS), which significantly affect the performance of UV_222_-based processes^69^. To comprehensively evaluate the role of NO_3_^−^, this study investigated the degradation of SDZ under different UV_222_-based processes in the presence of NO_3_^−^ (0.1−10 mM) (Fig. 6f and Supplementary Fig. 29e−j). Specifically, at low concentrations, RNS generated by NO_3_^−^ photolysis under UV_222_ can attack the sulfonamide group and the aromatic N atoms of the heterocyclic ring of SDZ, resulting in a slight promoting effect. However, as the NO_3_^−^ concentration increases, its light-shielding effect gradually reduces the degradation rate of the pollutant^70^. Notably, in the UV_222_-PAA system, the addition of NO_3_^−^ exhibited a significant inhibitory effect, which is consistent with previous reports^20^. This phenomenon is attributed to the strong photon competition by NO_3_^−^, which suppresses the activation of PAA and H_2_O_2_ in the solution, thereby preventing effective SDZ degradation^71^. In contrast, the *k*_obs_ value for the UV_222_-Br process increased by 3.58 to 5.14 times after the addition of NO_3_^−^. This is because •OH generated from NO_3_^−^ photolysis reacts rapidly with Br^−^ in solution to form •Br_2_^−^, which exhibits high reactivity toward SDZ (*k* ≈ 17.2 × 10^7 ^M^−1^·s^−1^)^31^. Based on these observations, in the UV_222_-PB process, the enhancement effect of NO_3_^−^ is optimal at a concentration of 1 mM, where the reaction rate constant is 1.39 times that of the system without NO_3_^−^. However, when the NO_3_^−^ concentration increases further, the removal rate of SDZ decreases. This is because low concentrations of NO_3_^−^ promote pollutant degradation by accelerating the generation of reactive bromine species, whereas the light-shielding effect at high NO_3_^−^ concentrations limits the formation of radicals in the system. Similarly, CO_3_^2−^ also exhibits absorption at 222 nm (*ε* = 42.3 M^−1^·cm^−1^). When introduced at low concentrations, CO_3_^2−^ reacts with •OH to form the longer-lived •CO_3_^−^ (Eqs. 27–29), which then attacks electron−rich sites in SDZ (5.5 × 10^8 ^M^−1^·s^−1^), including amino groups, sulfonamide groups, and the pyrimidine ring, leading to rapid oxidative degradation of SDZ^10,72^. Meanwhile, •CO_3_^−^ can also react with PAA to form •CH_3_C(O)OO, promoting the generation of organic radicals in the system (Eq. 30)^73^. However, the addition of a higher concentration of CO_3_^2−^ will cause a light shielding phenomenon and change the pH of the UV_222_-PB system (Supplementary Table 14). Under alkaline conditions, SDZ degradation is hindered, which is consistent with the results of previous pH experiments.

18 $${{\rm{CH}}}_{3}{{\rm{C}}}({{\rm{O}}}){{\rm{OOH}}}+{{\rm{Cl}}}^{-}\to {{\rm{HOCl}}}+{{\rm{CH}}}_{3}{{\rm{C}}}{{\rm{OO}}}^{-}+{{\rm{H}}}_{2}{{\rm{O}}}$$

19 $${{\bullet }}{{\rm{OH}}}+{{\rm{Cl}}}^{-}\to {{\bullet }}{{\rm{Cl}}}{{\rm{OH}}}^{-}$$

20 $${{\bullet }}{{\rm{Cl}}}{{\rm{OH}}}^{-}\to {{\bullet }}{{\rm{OH}}}+{{\rm{Cl}}}^{-}$$

21 $$\bullet {{\rm{Cl}}}+{{\rm{Cl}}}^{-}\to \bullet {{{\rm{C}}}{{\rm{l}}}_{2}}^{-}$$

22 $${{\bullet }}{{\rm{Cl}}}+{{\bullet }}{{\rm{Cl}}}\to {{\rm{Cl}}}_{2}$$

23 $${{\rm{Cl}}}_{2}+{{\rm{H}}}_{2}{{\rm{O}}}\to {{\rm{Cl}}}^{-}+{{\rm{HOCl}}}\,+\,{{\rm{H}}}^{+}$$

24 $${{\rm{Cl}}}^{-}+{{\rm{HOBr}}}+\,{{\rm{H}}}^{+}\to {{\rm{BrCl}}}+{{\rm{H}}}_{2}{{\rm{O}}}$$

25 $${{\rm{HOBr}}}+{{\rm{HOCl}}}\to {{\rm{BrOCl}}}+{{\rm{H}}}_{2}{{\rm{O}}}$$

26 $${{{\rm{SO}}}_{4}}^{2-}+{{\bullet }}{{\rm{OH}}}\to {{\bullet }}{{{\rm{SO}}}_{4}}^{-}-+{{\rm{OH}}}^{-}$$

27 $${{\rm{CO}}_{3}}^{2-}+{{\bullet }}{{\rm{OH}}}\to {{\bullet}}{{\rm{C}}}{{\rm{O}}_{3}}^{-}+{{\rm{OH}}}^{-}$$

28 $${{\rm{CO}}_{3}}^{2-}+{{\rm{H}}}_{2}{{\rm{O}}}+{{\rm{CO}}}_{2}\to 2{{\rm{H}}}{{{\rm{CO}}}_{3}}^{-}$$

29 $${{\rm{HC}}}{{\rm{O}}_{3}}^{-}+{{\bullet }}{{\rm{OH}}}\to {{\bullet }}{{\rm{C}}}{{\rm{O}}_{3}}^{-}+{{\rm{H}}}_{2}{{\rm{O}}}$$

30 $${{\rm{CH}}}_{3}{{\rm{C}}}({{\rm{O}}}){{\rm{OOH}}}+{{\bullet}}{{{\rm{CO}}}_{3}}^{-}\to {{\rm{CH}}}_{3}{{\rm{C}}}({{\rm{O}}}){{\rm{OO}}}+{{\rm{HC}}}{{\rm{O}}_{3}}^{-}$$

Real water matrices often consist of multiple complex ions. Therefore, this study configured trace‑salinity simulated water (TSSW), low‑salinity simulated water (LSSW), medium‑salinity simulated water (MSSW), and high‑salinity simulated water (HSSW) to better evaluate the performance of the UV_222_-PB process in water bodies with different salinity ranges. It can be observed that the UV_222_-PB process exhibited good degradation performance in all four simulated water matrices, with the pollutant being almost completely degraded within 20 minutes (Fig. 6g and Supplementary Fig. 30a). Moreover, the performance of the UV_222_-PB process was optimal in LSSW. Compared with TSSW, the appropriate concentrations of NO_3_^−^, SO_4_^2−^, and CO_3_^2−^ in LSSW promoted the generation of more reactive species in the system. However, as the salinity further increased, the degradation rate of SDZ gradually decreased, with a *k*_obs_ value of 0.2096 min^−1^ in HSSW. This may be attributed to the increase in pH caused by the addition of a higher concentration of CO_3_^2−^ as well as the light‑shielding effect of NO_3_^−^ (Supplementary Table 15). Overall, the UV_222_-PB process can operate stably across a wide salinity range from freshwater to seawater, providing strong support for its application in practical high‑salinity water bodies.

Natural organic matter is widely present in various water bodies and is one of the key factors influencing pollutant removal. Therefore, the representative Suwannee River III HA (SRHA) and Suwannee River III FA (SRFA) were selected for discussion in this study to further evaluate the stability of the UV_222_-PB system. The results show that although SRHA (*ε* = 0.0576 mg_C_^−1^·L·cm^−1^) and SRFA (*ε* = 0.0614 mg_C_^−1^·L·cm^−1^) have absorption at 222 nm, their addition has a negative effect on the UV_222_-PB system^10^. Specifically, as SRHA concentrations increased from 0 to 10 mg·L^−1^, the system’s *k*_obs_ value declined from 0.2190 min^−1^ to 0.0913 min^−1^ (Fig. 6h and Supplementary Fig. 30b). The negative effects of SRHA originate from its ability to quench •OH radicals (*k* = 2.50 × 10^4 ^mg^−1^·s^−1^) and •RO radicals (*k* = 5.76 × 10^4 ^mg^−1^·s^−1^) in solution, with •RO being the primary reactive species responsible for SDZ degradation^3^. Moreover, SRHA competes with the oxidant PAA for photons and causes a light-shielding effect, resulting in a drop in radical concentration^15^. Similarly, SRFA acts as a photo-competitor and has photo-shielding properties. When added at concentrations exceeding 1 mg·L^−1^, the reaction rate constant drops from 0.78 (*k*_obs_ = 0.1702 min^−1^) to 0.42 (*k*_obs_ = 0.0921 min^−1^) times that of the UV_222_-PB system (*k*_obs_ = 0.2190 min^−1^) (Fig. 6i and Supplementary Fig. 30c). Overall, while the addition of NOM decreased the degradation rate of pollutants, the degradation rate of SDZ still reached 100% within 30 minutes. It demonstrates that the UV_222_-PB system exhibits excellent stability and resistance to interference, indicating high potential for application in industrial water treatment.

### Practical applicability and long-term stability of the UV_222_-PB system

**Fig. 7:**
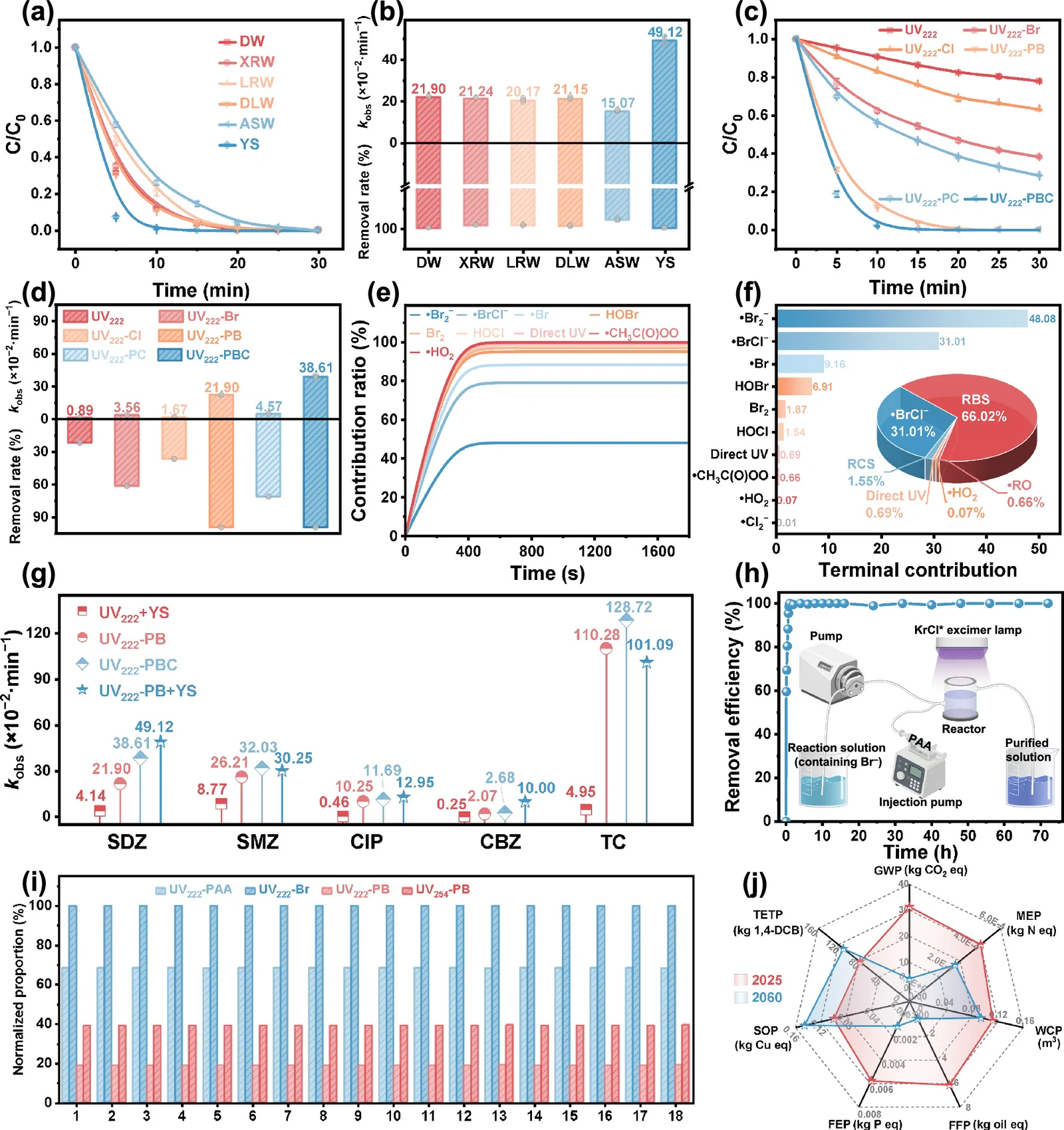
Sustainability and practical application assessment. **a** Degradation curves, (**b**) removal rates (20 min) and pseudo-first-order rate constant (*k*_obs_) of sulfadiazine (SDZ) under UV_222_-PB treatment in different real water conditions (DW: Deionized Water, XRW: Xiangjiang River water, LRW: Liuyang River water, DLW: Dongting Lake water, YS: Yellow Sea, ASW: Aquaculture Seawater Wastewater). **c** Degradation curves, (**d**) removal rates and *k*_obs_ of SDZ under different UV_222_ treatment conditions. In the UV_222_-PBC model, (**e**) the contributions of various active substances to SDZ degradation and (**f**) the terminal contribution. The inset summarizes the terminal contributions and categorized proportions of each reactive species (RBS: reactive bromine species, RCS: reactive chlorine species). **g** *k*_obs_ values for the degradation of SDZ, sulfamethazine (SMZ), ciprofloxacin (CIP), carbamazepine (CBZ), and tetracycline hydrochloride (TC) under the different UV_222_ process. **h** Removal efficiency of SDZ in continuous-flow experiments. The inset shows a schematic diagram of the continuous-flow photocatalytic reactor. **i** Comparative analysis of 18 environmentally relevant descriptors for the treatment of 50 mL of SDZ (20 µM) across different processes, including: 1: TAP, Terrestrial acidification potential; 2: GWP, Global warming potential; 3: FETP, Freshwater ecotoxicity potential; 4: METP, Marine ecotoxicity potential; 5: TETP, Terrestrial ecotoxicity potential; 6: FFP, Fossil fuel potential; 7: FEP, Freshwater eutrophication potential; 8: MEP, Marine eutrophication potential:; 9: HTPc, Human toxicity potential carcinogenic:; 10: HTPnc, Human toxicity potential non-carcinogenic:; 11: IRP, Ionizing radiation potential; 12: LOP, Agricultural land occupation; 13: SOP, Surplus ore potential; 14: ODP, Ozone depletion potential; 15: PMFP, Particulate matter formation potential; 16: HOFP, Photochemical oxidant formation potential humans:; 17: EOFP, Photochemical oxidant formation potential ecosystems:; and 18: WCP, Water consumption potential. **j** Comparison of the environmental impacts of UV_222_-PB (CF) in life cycle assessments for 2025 and 2060. Conditions: [OMPs]_0_ = 20 μM, pH = 7.0, [PAA]_0_ = [Br]_0_ = 0.5 mM, [Cl]_0_ = 0.5 M. Error bars represent SD of three replicate experiments, and the central value is the mean of the data. Data are presented as mean values ± SD. Source data are provided as a Source Data file.

As is generally known, actual water bodies frequently have more complicated influencing factors that interfere with the degradation process of pollutants. This study further evaluated the UV_222_-PB system in Deionized Water (DW), XRW, Liuyang River water (LRW), Dongting Lake water (DLW), YS, and ASW. This evaluation aimed to better assess the practical applicability of the UV_222_-PB system. Detailed water quality parameters are provided in Supplementary Table 16. Obviously, the UV_222_-PB system maintained stable performance in XRW (*k*_obs_ = 0.2124 min^−1^), LRW (*k*_obs_ = 0.2017 min^−1^), and DLW (*k*_obs_ = 0.2115 min^−1^) (Fig. 7a−b and Supplementary Fig. 31a). The *k*_obs_ value of SDZ only slightly decreased. It may be due to the high concentration of HCO_3_^−^ in the system, which led to the inhibition effect brought by alkaline solution and the positive effect brought by Cl^−^ counterbalance each other. However, in ASW (*k*_obs_ = 0.1507 min^−1^) water sample, the degradation of SDZ was still prevented due to the light shielding and competition effect generated by high organic matter content, even at high Cl^−^ concentrations (2862 mg·L^−1^). Specifically, the UV_222_-PB system performed substantially better in YS water (*k*_obs_ = 0.4912 min^−1^), attaining a 99.19% SDZ degradation rate in 10 minutes. This strong promotion effect was attributed to the high concentration of Cl^−^ (25367 mg·L^−1^), resulting in a considerable promotion effect, leading to the conversion of more active species.

However, while high concentrations of Cl^−^ promote degradation, they may also compete with Br^−^ for reactive species, thereby interfering with the formation of bromine species^74^. To better clarify the dominant reactive species responsible for pollutant degradation in the UV_222_-PB process in real seawater, we conducted a series of supplementary experiments under high Cl^−^ concentration (Fig. 7c−d and Supplementary Fig. 31b). Specifically, in the UV_222_-Cl system containing only 0.5 M Cl^−^, the degradation of SDZ was 36.92%, and its *k*_obs_ value was only 0.47 times that of the UV_222_-Br system. Upon the addition of PAA, although the *k*_obs_ value of SDZ degradation in the UV_222_-PAA-Cl (UV_222_-PC) system increased to 0.0457 min^−1^, it remained much lower than that in the UV_222_-PB process. Moreover, compared with Dark-PB, the degradation rate of SDZ in Dark-PC decreased by approximately 60.16% (Supplementary Fig. 31c–e), a difference that may be attributed to the fact that the rate constant for the reaction of PAA with Cl^−^ to form HOCl is about four orders of magnitude lower than that for the reaction of PAA with Br^−^ to form HOBr^30^. Notably, in the UV_222_-PAA-Br-Cl (UV_222_-PBC) system containing 0.5 M Cl^−^, SDZ was already degraded by 98.13% within 10 minutes, and its *k*_obs_ value was 1.76 and 8.45 times higher than those in the UV_222_-PB and UV_222_-PC systems, respectively. In summary, the above experimental results demonstrate that chlorine‑derived species alone cannot achieve efficient pollutant degradation, and the presence of Br^−^ is an indispensable part of performance enhancement.

Furthermore, to further elucidate the attribution and interaction of dominant reactive species in the UV_222_-PB process under high chloride concentration, we introduced 0.5 M Cl^−^ into the original kinetic model and constructed the UV_222_-PBC model to better simulate seawater and seawater‑affected high‑salinity water bodies. Remarkably, the terminal contribution analysis of the UV_222_-PBC model revealed that RBS (66.02%) contributed far more to SDZ degradation than RCS (1.55%), with •Br_2_^−^ becoming the dominant reactive species, contributing as much as 48.08% (Fig. 7e−f and Supplementary Fig. 32a–e). This phenomenon is attributed to the rapid conversion of RCS to RBS under high Cl^−^ concentration. Specifically, •Cl and •Cl_2_^−^ generated in the system can rapidly react with Br^−^ to form •BrCl^−^, resulting in low steady-state concentrations of •Cl and •Cl_2_^−^ at 5.57 × 10^−15^ M and 3.83 × 10^−14^ M, respectively. Moreover, although HOCl is present in high abundance, its second-order rate constant with SDZ is relatively low. Consequently, the contribution of RCS to SDZ degradation is rather limited. Model predictions indicate that •Br_2_^−^ and •BrCl^−^ are the two major contributing bromine-related species, with steady-state concentrations of 5.19 × 10^−11^ M and 2.97 × 10^−11^ M, respectively. This pattern can be explained by the fact that RCS promotes the generation of •BrCl^−^, while •BrCl^−^ can rapidly react with Br^−^ to form •Br_2_^−^ (k = 8.00 × 10^9 ^M^−1^·s^−1^), leading to an approximately 181% increase in the steady-state concentration of •Br_2_^−^ in the UV_222_-PBC model compared with the UV_222_-PB model. Although the steady-state concentration of •BrCl^−^ (1.78 × 10^8 ^M^−1^·s^−1^) is only 0.59 times that of •Br_2_^−^, its second-order rate constant with SDZ is slightly higher than that of •Br_2_^−^ (1.53 × 10^8 ^M^−1^·s^−1^), making •BrCl^−^ an important contributor to SDZ degradation. Meanwhile, the generation of •Br is significantly inhibited. The relatively low H⁺ concentration in the neutral environment limits the conversion of •BrOH^−^ to •Br, and the high concentration of Cl^−^ also consumes •Br, jointly leading to an approximately 5.6-fold decrease in the steady-state concentration of •Br. Therefore, multiple factors collectively result in •Br_2_^−^ far exceeding •Br in its contribution to SDZ degradation, making it the dominant reactive bromine species in the UV_222_-PBC system.

On the other hand, the rapid interconversion among halogen reactive species drives the dynamic evolution of the compositional structure of reactive species in the system. Accordingly, the model further quantified the steady‑state concentrations and reaction contributions of mixed bromine‑chlorine species (e.g., •BrCl^−^, BrCl, BrOCl). Due to the relatively low reaction rate between HOBr and HOCl, the formation concentration of BrOCl is very low, rendering its contribution to SDZ degradation negligible. Compared with the UV_222_-PB model, the steady‑state concentrations of •Br and •BrOH^−^ in the UV_222_-PBC model decrease significantly, a phenomenon attributed to the rapid reaction of these species with high‑concentration Cl^−^, leading to the formation of mixed halogen species such as •BrCl^−^. Furthermore, although the steady‑state concentrations of BrCl and •BrCl^−^ are comparable, their reaction rates with SDZ differ by approximately five orders of magnitude, resulting in a marked difference in their contributions to SDZ degradation. Notably, the model predictions show that compared with UV_222_-PB, PAA consumption decreases in the UV_222_-PBC model, while H_2_O_2_ consumption increases (Supplementary Fig. 32f–g). This is because the steady‑state concentration of •OH in the UV_222_-PBC system decreases to 3.13×10^−17^ M, which hinders the reaction of PAA with •OH, leading to an 89% decrease in the steady‑state concentration of •CH_3_C(O)OO. This is the main reason for the significant reduction in the contribution of •CH_3_C(O)OO in the UV_222_-PBC model. Meanwhile, H_2_O_2_ in the UV_222_-PBC system is required not only to reduce HOBr but also to react with HOCl, resulting in increased H_2_O_2_ consumption. Nevertheless, the Br^−^ concentration remains largely unchanged during the reaction, confirming the robust operation of the bromine cycle under high‑chloride conditions. In summary, although Cl^−^ concentration is overwhelmingly dominant, Br^−^ still plays an indispensable role in the UV_222_-PBC system, allowing bromine and chlorine species to jointly dominate the oxidative degradation of pollutants.

Real seawater contains various complex components. To further evaluate the universality of the UV_222_-PB process for pollutant degradation in real seawater, this study selected typical aquatic OMPs such as SMZ, CIP, CBZ, and TC, and systematically investigated their degradation behavior in YS water matrix (Fig. 7g and Supplementary Fig. 33). Notably, compared with the UV_222_-PB system, the addition of high‑concentration chloride ions increased the degradation rates of all pollutants. The *k*_obs_ values of SMZ, CIP, CBZ, and TC in the UV_222_-PBC system increased to 0.3203 min^−1^, 0.1169 min^−1^, 0.0268 min^−1^, and 1.2872 min^−1^, respectively. This enhancement is attributed to the increase in reactive species such as •Br_2_^−^ and HOCl, as well as the synergistic oxidation arising from the rapid interconversion among halogen reactive species. However, in real YS water, the UV_222_-PB process exhibited differentiated degradation trends for different pollutants. Specifically, SMZ (3.18 × 10^8 ^M^−1^·s^−1^) and TC (6.26 × 10^8 ^M^−1^·s^−1^) show high reactivity toward •Br_2_^−31^. In real YS water, the coexisting CO_3_^2−^ and HCO_3_^−^ effectively quench •OH in the system, thereby inhibiting the generation of •Br and •Br_2_^−^. Since TC exhibits a higher reaction rate with •Br_2_^−^ than SMZ, its degradation is more significantly inhibited. In contrast, CIP and CBZ show relatively lower reactivity toward bromine species and display a promoting effect in real seawater^31,75^. This is because SO_4_^2−^ present in real water matrices can be converted into secondary radicals such as •SO_4_^−^, which rapidly attack and degrade the pollutants. Among them, CBZ shows a more pronounced degradation promotion, which may be attributed to the high reaction rate of •SO_4_^−^ with CBZ (1.92 × 10^9 ^M^−1^·s^−1^) and its effective disruption of the central heterocyclic olefinic double bond in CBZ^76^. Although the degradation efficiency of UV_222_-PB for different OMPs in real seawater varies, the overall removal performance remains stable and efficient. This confirms that the UV_222_-PB process possesses broad‑spectrum and high‑efficiency removal capabilities for OMPs, demonstrating good application potential in high‑salinity water treatment.

Based on the aforementioned water quality parameters and the excellent results from in-situ water body experiments, this study further investigated the long-term stability of the UV_222_-PB system in a custom-built continuous operation setup to provide a reference for its industrial-scale upscaling. The results showed that after 72 h of continuous operation, the degradation efficiency of SDZ by the UV_222_-PB system remained above 98%, with a cumulative treated wastewater volume of 16.8 L. Moreover, considering that the theoretical hydraulic retention time of the pollutant solution in the reactor was approximately 1 h, we monitored the degradation of SDZ during the initial 1 h of reactor operation. The results indicated that the *k*_obs_ value for SDZ degradation reached 5.32 h^−1^, and SDZ was almost completely degraded in the reactor effluent after 1 h of operation (Fig. 7h and Supplementary Figs. 34−35). This occurrence further demonstrated the robust stability and significant potential for effective pollutant removal of UV_222_-PB system, providing a valuable reference for practical water treatment applications.

Additionally, the sustainability and environmental impacts of different processes were comprehensively and systematically quantified through life cycle assessment (LCA) (Supplementary Table 17)^77^. The results showed that, compared with processes such as UV_254_-PB, the UV_222_-PB process demonstrated superior environmental performance across multiple environmental impact indicators, confirming its favorable environmental friendliness and sustainability (Fig. 7i and Supplementary Figs. 36–37). Moreover, in the 16.8 L continuous-flow experiment, the UV_222_-PB (CF) process maintained high pollutant removal efficiency while sustaining a low carbon emission level. This further verified a good balance between its environmental friendliness and treatment efficiency, providing solid data support for its future engineering application. To further clarify the positioning and advantages of the UV_222_-PB (CF) process among water treatment technologies, this study compared it with other typical water treatment processes, targeting the treatment of 1 m^3^ of water^33,78^. To strengthen the fairness and consistency of the comparison, we extracted the life cycle inventory data of each process and recalculated them using the same LCA methodology as in this study. It can be seen that the UV_222_-PB (CF) process outperformed the compared processes such as O_3_, CaO_2_/O_3_, and TiO_2_ photocatalysis in most environmental impact categories (Supplementary Fig. 38). Among them, the CO_2_ emissions of UV_222_-PB were 31.34 kg CO_2_ eq·m^−3^, which is only 36.99% of that of the CN-KI/PI/Sunlight process (84.72 kg CO_2_ eq·m^−3^). Notably, since the UV_222_-PB process does not involve catalyst preparation, electricity input has become the main factor affecting its environmental pressure. According to Zhu et al.‘s projection of China’s electricity mix in 2060, under the policy-driven scenario, the share of thermal power will drop to approximately 2.07%, while wind power and photovoltaic power will account for 36.5% and 36.9%, respectively^79^. Based on this, this study further assessed the life cycle environmental impacts of the UV_222_-PB (CF) process under the different electricity mixes in 2025 and 2060 (Fig. 7j and Supplementary Fig. 39). The results indicate that when the electricity mix changes, indicators such as surplus ore potential (SOP) and terrestrial ecotoxicity potential (TETP) show an upward trend due to the upstream metal mining and refining activities driven by the large-scale deployment of wind and solar power^80^. However, with the continuous clean transition of the electricity mix, environmental impact indicators such as global warming potential (GWP) and fossil fuel potential (FFP) of the UV_222_-PB process decrease significantly, with CO_2_ emissions dropping from 31.34 kg CO_2_ eq·m^−3^ to 3.73 kg CO_2_ eq·m^−3^. Overall, against the backdrop of the deepening global energy transition, grid decarbonization will further amplify the environmental advantages of the UV_222_-PB process. For future development, it is recommended to increase the operating load rate of electrical equipment to as close to saturation as possible, thereby helping to reduce the environmental burden.

## Discussion

Previous studies have demonstrated that 222 nm-based water treatment technology holds significant application prospects. Its energy-efficient, high-performance, and environmentally friendly characteristics align well with current sustainable development goals, making it crucial for achieving clean drinking water and sanitation. In this work, PAA and Br^−^ exhibited distinct light absorption characteristics in the 222 nm far-UVC region, with their ε values increasing by 6.45 and 39 times, respectively, compared to those at 254 nm. Consequently, UV_222_-PB achieved effective degradation performance and lower energy consumption costs. The enhanced degradation performance is attributed to the effective activation and interaction of PAA and Br^−^ under UV_222_ irradiation, generating more RBS and •RO that accelerate the degradation rate of SDZ. This is supported by quenching experiments, probe tests, ESR analysis, and kinetic modeling. Due to the predominantly electrophilic nature of the reactive species in the system, the ln(*k*_obs_) values of ten OMPs with different structures showed a significant correlation with their IP and E_HOMO_ values, confirming the high selectivity of the UV_222_-PB process toward electron-rich pollutants. Furthermore, the environmental applicability and eco-friendliness of the process were evaluated. The degradation rate of SDZ in the UV_222_-PB system exhibited a strong dependence on solution pH, with acidic and neutral conditions being more favorable for pollutant degradation. The process also demonstrated relatively stable performance in the presence of inorganic anions and NOM, across mixed-anion systems covering a wide salinity range, and in various real water matrices.

Regarding the potential risks of DBPs formation, the study monitored bromate formation in different processes and conducted toxicity assessments from multiple perspectives, including the ECOSAR model, wheat and amaranth seed germination tests, and zebrafish embryo culture. The results showed that the ecotoxicity of the treated water was significantly reduced. LCA further confirmed the environmental friendliness and sustainability of the UV_222_-PB process. However, since the UV_222_-PB process does not involve catalyst preparation, electricity input remains the main factor affecting the environmental pressure of the process. In future development, it is recommended to operate electrical equipment under saturated load conditions, which would contribute to reducing the environmental burden. In summary, this work highlights the feasibility of coupling oxidants with natural water matrix components at 222 nm, suggesting a potential pathway for the degradation of OMPs under far-ultraviolet light.

## Methods

### Chemicals and reagents

All reagents used in this study were of analytical grade or higher. Detailed chemical information is listed in Text S1 of Supporting Information.

### Experimental procedures

In pollutant degradation experiments, the UV irradiation apparatus (Supplementary Fig. 40) employed either a KrCl* excimer lamp (UV_222_, FIRST B2-15W, Guangdong Excimer Optoelectronic Technology Co., Ltd., China), an LPUV lamp (UV_254_, 10 W, Guangzhou Xingchuang Electronics Co., Ltd., China), or an MPUV lamp (UV_365_, 10 W, Guangzhou Xingchuang Electronics Co., Ltd., China) as the ultraviolet radiation source, coupled with a bandpass filter. The emission spectra of the lamps are shown in Supplementary Fig. 41. Before the experiment began, the KrCl* excimer lamp required 5 min warm-up period (LPUV and MPUV lamps required 30 min) to ensure stable light intensity. For detailed calculations of UV measurements, refer to Text S2.

In the UV_222_-PB degradation experiment, the initial pH of the sulfadiazine (SDZ) (20 μM) reaction solution with phosphate-buffered solution (10 mM) was adjusted to neutral. To activate the reaction, 0.5 mM of Br^−^ and PAA were added to the solution, respectively, and exposed to UV_222_ irradiation while swirling at 300 rpm. At predetermined time intervals, 0.7 mL of reaction solution was collected, filtered through 0.22 μm membrane filter, and transferred to a brown sample vial. An excess of quenching solution was immediately added to terminate the reaction (each sample represents an independent irradiation event). The treated samples were then quantitatively analyzed using high-performance liquid chromatography (HPLC). For other systems, such as the sole UV_222_ system, no PAA or Br^−^ was added, and the reaction was initiated directly by UV_222_ irradiation. All degradation experiments were conducted independently and repeated at least three times. The reported data include the mean and standard deviation. For detailed information regarding other experiments, please refer to Supplementary Text S8.

### Analytical methods

Detailed analytical methods for organic pollutant concentrations, disinfection byproducts, and SDZ degradation intermediates are provided in Texts S9–S11 and Supplementary Table 1.

### Toxicity assessment of SDZ and its intermediates based on the ECOSAR model

To evaluate the potential applications of UV_222_-PB system, the ecotoxicity of SDZ and its intermediates was predicted using the ECOSAR model (Version 2.2). Based on the activity relationship of ecological structure, the model can predict the acute and chronic toxicity for fish, Daphnia, and green algae. Among them, the semi-lethal concentration (LC_50_) and the semi-effective concentration (EC_50_) are used to reflect acute toxicity, while the chronic value (ChV) represents chronic toxicity.

### Wheat seed culture experiment

The phytotoxicity of water samples treated under different systems was investigated through wheat seed germination experiments. Specifically, the wheat seeds were extensively cleaned with deionized water to eliminate any remaining H_2_O_2_ after being steeped in a 3% H_2_O_2_ solution for 20 minutes to disinfect them. Then, select 20 plump and uniformly sized wheat seeds. Distribute them evenly across the bottom of a petri dish lined with filter paper. Add 7 mL of solution treated with different systems to each seed (using deionized water and untreated SDZ solution as controls) to support seed germination and growth. And later, the culture dishes were placed in a constant-temperature incubator at 25 °C with a 12/12 h light-dark cycle. Photographs were taken every 24 h to document wheat growth, and the solution in the Petri dishes was replenished regularly to compensate for evaporation losses. After 120 h, the germination rate of wheat seeds stabilized, and seedling stem length was measured to evaluate wheat growth performance under different treatment conditions. To ensure the accuracy of experimental results, three parallel samples were established for each treatment group to cultivate wheat seeds.

### Germination experiment of amaranth seeds

In the amaranth seed germination experiment, amaranth seeds were washed three times with deionized water to remove surface impurities. Subsequently, 20 uniformly sized amaranth seeds were selected and evenly distributed in a Petri dish lined with filter paper. Each dish was then added 5 mL of solution treated with different systems (using deionized water and untreated SDZ solution as controls). The dishes were covered and placed in a constant-temperature incubator at 25 °C. After 48 h, the sprout length and germination rate of the amaranth seeds were recorded to assess the toxicity of the aqueous solutions treated with different systems. To ensure the accuracy of the experimental results, three parallel samples were set up for each group to cultivate the amaranth seeds.

### Experiment of zebrafish embryo culture

All zebrafish husbandry and experimental procedures were approved by the Research Ethics Committee of the College of Environmental Science and Engineering, Hunan University (HNU-ESELL-Z501). Using the hatching rate and teratogenic effects of zebrafish embryos as indicators, the developmental toxicity of degradation intermediates from different systems was evaluated. The AB strain zebrafish were provided by the China Zebrafish Resource Center (CZRC). Before the experiment, two healthy sexually mature female zebrafish and one male were placed into a spawning box equipped with a divider. After being kept in darkness overnight, the divider was removed upon exposure to light the next morning. Following spawning, fertilized zebrafish eggs with developmental potential were selected. The selected eggs were then rinsed with water from the culture system to remove any contaminants adhering to the egg surfaces. Water samples treated by different systems were used as exposure solutions, while untreated SDZ and nutrient solution (blank) served as control groups. At 6 hpf, zebrafish embryos were randomly distributed into 24-well plates, with 10 embryos per group and each well containing 1 mL of sample solution, which was replaced every 24 hours. Throughout the experiment, exposure solutions were renewed every 24 hours to ensure stability. Images of zebrafish larvae were captured using a stereomicroscope (Leica, Germany), and all parameter settings were kept consistent across groups. All animal experiments were conducted in strict accordance with the Guide for the Care and Use of Laboratory Animals published by the National Research Council of the United States^81^.

### Preprocessing procedures and parameters for DBPs analysis

In order to evaluate the formation potential of DBPs, the formation concentrations of tribromomethane (TBM), bromodichloromethane (BDCM), dibromochloromethane (DBCM), trichloromethane (TCM), monochloroacetic acid (MCAA), Monobromoacetic acid (MBAA), dichloroacetic acid (DCAA), trichloroacetic acid (TCAA), bromochloroacetic acid (BCAA), dibromoacetic acid (DBAA), bromodichloroacetic acid (BDCA), chloro-dibromoacetic acid (CDBA) and tribromoacetic acid (TBAA) in trihalomethane (THMs) and haloacetic acid (HAA) were monitored. Determination of THMs concentration according to USEPA method 551.1: First, add 10 mL of the post-reaction water sample to the sample vial, then add 2 g of anhydrous sodium sulfate and 3 mL of MTBE, stir for 2 min, let it stand for 20 min, and then collect the upper organic phase for further analysis. The concentration of HAA was determined by using USEPA method 552.3: firstly, add 25 mL of the post-reaction water sample to the sample vial, then 2 mL of concentrated sulfuric acid, 10 g of anhydrous sodium sulfate, and 4 mL of MTBE were added in turn, stirred for 2 min and left standing for 10 min. The collected supernatant was mixed with the currently prepared sulfuric acid-methanol solution (1: 9, v: v) and reacted for 2 h in a water bath at 50°C. Saturated sodium bicarbonate is added to the cooled solution for reaction to consume excessive acid, and finally, the upper organic phase can be collected for further analysis.

### Theoretical calculations

All DFT calculations were performed using the Gaussian 16 software package. Within the framework of density functional theory (DFT), the geometries of the molecules were optimized using the M06-2X functional and the 6-31G(d) basis set. The DFT-D3 dispersion correction method and the IEFPCM implicit solvent model were employed to describe van der Waals interactions and the solvation effects of water. Single-point calculations were performed on the optimized structures using the B3PW91 functional and the larger 6-311G(d) basis set to determine the energy levels of the frontier orbitals. To calculate the vertical ionization potential (IP) and electron affinity (EA), the M06-2X functional and the 6-311G(d) basis set were also used to compute the electron density and energy of these optimized structures in their neutral, cationic, and anionic states. Using the Multiwfn program, the Fukui functions (*f* ^*+*^, *f* ^−^, *f* ^*0*^) and dual descriptors were calculated via finite differences of the electron density in the neutral, anionic, and cationic states. Conceptual density functional theory (CDFT) calculations were performed to determine the Mulliken electronegativity, electrophilicity index (EI), and nucleophilicity index (NI), which are key electronic descriptors for analyzing electronic structure and reactivity. Visualizations of frontier molecular orbitals and Fukui functions were generated using the Visual Molecular Dynamics (VMD) program.

On the other hand, for Gibbs free energy calculations, the structures were optimized using the M06-2X functional and the def2-TZVP basis set. The transition state was optimized by the rational function optimization (RFO) method. Vibrational frequencies were calculated at the same level to ensure no imaginary frequencies at the minima and saddle points, and the Gibbs free energy was calculated at 298.15 K and 1 atm. Excited-state calculations employed the time-dependent density functional theory (TDDFT) method. Based on the universal solvation model proposed by Truhlar et al., which uses the solute electron density, water was treated as an implicit solvent in all calculations. Additionally, high‑precision single‑point energy calculations were carried out using the def2‑TZVP basis set together with Grimme’s DFT‑D3 empirical dispersion correction. Ground‑state geometry optimizations were also performed using the M06‑2X functional and the 6-311G(d) basis set, and excited states were calculated with TDDFT at the optimized ground‑state geometries. The electron distributions at the HOMO and LUMO of the models were visualized using VMD 1.9.3 combined with Multiwfn 3.8. The polarizable continuum model (PCM) implicit solvation model was chosen to account for the solvation effect of water, which is widely accepted for approximating solvent influences on energy‑related parameters and helps to minimize deviations that might arise from neglecting solvation effects. Supplementary Data 1 provides the optimized geometric coordinates of all molecules involved in this study.

### Kinetic model

The kinetic equations were numerically solved using Python’s scipy.integrate.solve_ivp function, with the solver configured to use the backward differentiation formula (BDF) method, which is suitable for stiff reaction systems. This approach effectively handles the disparate time scales arising from fast radical reactions, the transformation of reactive halogen species, and multi‑component coupled reactions. The relative and absolute error tolerances are set to rtol equal to 5 × 10^−5^ and atol equal to 1 × 10^−12^, respectively, to improve the numerical stability of low-concentration reactive intermediates such as, •Br, •Br_2_^−^, and •Cl_2_^−^ while ensuring computational efficiency. During the parameter optimization process, each set of candidate parameters is converted into corresponding reaction rate constants and substituted into the complete reaction network; the fully coupled system of ordinary differential equations (ODEs) is then reintegrated to obtain a series of relevant fit data for SDZ. The goodness of fit between simulated and experimental values was evaluated using MSE and R^2^ to assess parameter adequacy. Detailed information on the construction of the ODE model and the reaction network is provided in Text S12 and Supplementary Table 18.

### Life cycle assessment

LCA was conducted using Simapro software with the Ecoinvent (3.10) database to quantitatively analyze 18 environmental impact indicators under the ReCiPe 2016 v1.03, midpoint (H) methodology and investigate the environmental impacts of different study systems. Based on actual experimental data, the functional unit defined for this study is 50 mL (1 m^3^) SDZ (20 μM). The 18 impact categories are as follows: (1) TAP, Terrestrial acidification potential (kg SO_2_-Eq); (2) GWP, Global warming potential (kg CO_2_-Eq); (3) FETP, Freshwater ecotoxicity potential (kg 1,4-DCB-Eq); (4) METP, Marine ecotoxicity potential (kg 1,4-DCB-Eq); (5) TETP, Terrestrial ecotoxicity potential (kg 1,3-DCB-Eq); (6) FFP, Fossil fuel potential (kg oil-Eq); (7) FEP, Freshwater eutrophication potential (kg P-Eq); (8) MEP, Marine eutrophication potential (kg N-Eq); (9) HTPc, Human toxicity potential (carcinogenic) (kg 1,4-DCB-Eq); (10) HTPnc, Human toxicity potential (non-carcinogenic) (kg 1,4-DCB-Eq); (11) IRP, Ionizing radiation potential (kBq Co-60-Eq); (12) LOP, Agricultural land occupation (m^2^·a crop-Eq); (13) SOP, Surplus ore potential (kg Cu-Eq); (14) ODP, Ozone depletion potential (kg CFC-11-Eq); (15) PMFP, Particulate matter formation potential (kg PM_2.5_-Eq); (16) HOFP, Photochemical oxidant formation potential (humans) (kg NO_x_-Eq); (17) EOFP, Photochemical oxidant formation potential (ecosystems) (kg NO_x_-Eq); and (18) WCP, Water consumption potential (m^3^).

### Reporting summary

Further information on research design is available in the Nature Portfolio Reporting Summary linked to this article.

## Supplementary information

Reporting Summary

Supplementary Information

Description of Additional Supplementary Files

Supplementary Data 1

Transparent Peer Review file

## Source data

Source Data

## Data Availability

The data supporting the findings of the study are included in the main text and Supplementary Information files. The data that support the findings of this study are openly available in Figshare at https://doi.org/10.6084/m9.figshare.33302178. Source data are provided with this paper^82^ .
