# Supplementary material for: Bromide-mediated enhancement of far-UVC/peracetic acid process for selective micropollutant abatement
Source: Nat Commun. 2026 Oct 8;17:11295. doi: 10.1038/s41467-026-78410-2 (PMC13722704; doi:10.1038/s41467-026-78410-2)
Supplement: Supplementary file 1 [file 41467_2026_78410_MOESM1_ESM.pdf]

nature portfolio

Corresponding author(s): Xiaofei Tan

Last updated by author(s): Sep 7, 2026

Reporting Summary

Nature Portfolio wishes to improve the reproducibility of the work that we publish. This form provides structure for consistency and transparency in reporting. For further information on Nature Portfolio policies, see our Editorial Policies and the Editorial Policy Checklist.

Statistics

For all statistical analyses, confirm that the following items are present in the figure legend, table legend, main text, or Methods section.

n/a	Confirmed	
☐	☒	The exact sample size (*n*) for each experimental group/condition, given as a discrete number and unit of measurement
☐	☒	A statement on whether measurements were taken from distinct samples or whether the same sample was measured repeatedly
☒	☐	The statistical test(s) used AND whether they are one- or two-sided<br>*Only common tests should be described solely by name; describe more complex techniques in the Methods section.*
☒	☐	A description of all covariates tested
☒	☐	A description of any assumptions or corrections, such as tests of normality and adjustment for multiple comparisons
☐	☒	A full description of the statistical parameters including central tendency (e.g. means) or other basic estimates (e.g. regression coefficient) AND variation (e.g. standard deviation) or associated estimates of uncertainty (e.g. confidence intervals)
☒	☐	For null hypothesis testing, the test statistic (e.g. *F*, *t*, *r*) with confidence intervals, effect sizes, degrees of freedom and *P* value noted<br>*Give P values as exact values whenever suitable.*
☒	☐	For Bayesian analysis, information on the choice of priors and Markov chain Monte Carlo settings
☒	☐	For hierarchical and complex designs, identification of the appropriate level for tests and full reporting of outcomes
☒	☐	Estimates of effect sizes (e.g. Cohen's *d*, Pearson's *r*), indicating how they were calculated

Our web collection on statistics for biologists contains articles on many of the points above.

Software and code

Policy information about availability of computer code

Data collection	The concentrations of organic pollutants were determined by high-performance liquid chromatography (HPLC, Agilent 1100 Infinity II) equipped with a UV detector and a C18 column. Dissolved oxygen (DO) was measured using a DO meter (HQ2200). The concentrations of bromine and bromates were analyzed by ion chromatography (IC, AQUION). Degradation intermediates were identified by ultra-performance liquid chromatography coupled with quadrupole time-of-flight mass spectrometry (UPLC-Q-Tof 6550). Radical species were confirmed by electron spin resonance (ESR, Bruker A300). Density functional theory (DFT) calculations were performed using Gaussian 16 Rev. C.01. Numerical solving of kinetic equations was implemented using Python version 3.10.
Data analysis	All degradation kinetics of pollutants were processed using Origin 2021 software. Molecular structures were drawn using ChemDraw 2022 software. Three-dimensional molecular models were constructed using Cinema 4D 2023. Schematic diagrams were drawn using Adobe Illustrator 2023. The toxicity of SDZ and its degradation intermediates was predicted using the Ecological Structure-Activity Relationships (ECOSAR, Version 2.2) model. Life cycle assessment was conducted using Simapro (version 10.1) software in conjunction with the Ecoinvent (3.10) database.

For manuscripts utilizing custom algorithms or software that are central to the research but not yet described in published literature, software must be made available to editors and reviewers. We strongly encourage code deposition in a community repository (e.g. GitHub). See the Nature Portfolio guidelines for submitting code & software for further information.

Data

Policy information about availability of data

All manuscripts must include a data availability statement. This statement should provide the following information, where applicable:

- Accession codes, unique identifiers, or web links for publicly available datasets
- A description of any restrictions on data availability
- For clinical datasets or third party data, please ensure that the statement adheres to our policy

The data supporting the findings of the study are included in the main text and Supplementary information files. The data that support the findings of this study are openly available in Figshare at https://doi.org/10.6084/m9.figshare.33302178. Source data are provided in this paper.

Research involving human participants, their data, or biological material

Policy information about studies with human participants or human data. See also policy information about sex, gender (identity/presentation), and sexual orientation and race, ethnicity and racism.

Reporting on sex and gender	The manuscript does not mention this information.
Reporting on race, ethnicity, or other socially relevant groupings	The manuscript does not mention this information.
Population characteristics	The manuscript does not mention this information.
Recruitment	The manuscript does not mention this information.
Ethics oversight	The manuscript does not mention this information.

Note that full information on the approval of the study protocol must also be provided in the manuscript.

Field-specific reporting

Please select the one below that is the best fit for your research. If you are not sure, read the appropriate sections before making your selection.

☐ Life sciences ☐ Behavioural & social sciences ☒ Ecological, evolutionary & environmental sciences

For a reference copy of the document with all sections, see nature.com/documents/nr-reporting-summary-flat.pdf

Ecological, evolutionary & environmental sciences study design

All studies must disclose on these points even when the disclosure is negative.

Study description	In this study, we explored the reaction mechanisms and toxicological implications of a far-UVC-based peracetic acid process that enhances the production of reactive species in bromine-rich water. The process not only achieves efficient degradation of organic micropollutants but also suppresses the formation of disinfection byproducts, offering a more sustainable and cleaner alternative for the treatment of high bromine wastewater.
Research sample	This study employed both simulated wastewater and real natural water samples as the research matrix. The simulated wastewater, which was prepared in the laboratory with varying compositions (e.g., different Br^- concentrations, pH values, or natural organic matter content), served to systematically elucidate the reaction mechanisms and key influencing factors under well-controlled conditions. In contrast, the actual natural water bodies were utilized to evaluate the environmental feasibility and robustness of the process in complex aqueous matrices.
Sampling strategy	The degradation experiments were conducted in a 50 mL petri dish containing an SDZ solution. The reaction was initiated by adding peracetic acid or bromide ions to the solution while simultaneously exposing it to UV222 irradiation, and samples were then collected and analyzed at predetermined intervals. All degradation experiments were conducted independently and repeated at least three times. The reported data include the mean and standard deviation. Data are presented as mean values +/- SD.
Data collection	At predetermined time intervals, 0.7 mL of the reaction solution was collected, filtered through a 0.22 μm membrane, transferred to an amber sample vial, and immediately quenched with an excess of termination solution to stop the reaction for subsequent analysis (each sample represented an independent irradiation). All experiments were conducted independently and repeated at least three times. A high-performance liquid chromatograph (Agilent 1100 Infinity II) equipped with a UV detector and a C18 column (5 μm, 4.6 × 250 mm) was used to quantify the residual contaminant concentration in the solution. A UV-Vis spectrophotometer was used to determine the concentrations of peracetic acid and coexisting hydrogen peroxide in the solution. The concentrations of bromide and bromate in the samples were analyzed by ion chromatography (IC, AQUION). An Agilent 1290 ultra-performance liquid chromatography quadrupole time-of-flight mass spectrometer (UPLC-Q-ToF 6550) was employed to analyze the formation of 4-Br-DMPZ and the intermediate products of SDZ degradation. Using 2,2,6,6-tetramethylpiperidine (TEMP), 5,5-dimethyl-1-pyrroline N-

	oxide (DMPO), and 5-diisopropoxyphosphoryl-5-methyl-1-pyrroline N-oxide (DIPPMPO) as trapping agents, the types of free radicals in the system were identified by electron spin resonance (ESR, Bruker A300). Use the Ecological Structure-Activity Relationship (ECOSAR) model to predict the toxicity of SDZ and its degradation intermediates. Conduct a life cycle assessment using Simapro software.
Timing and spatial scale	This study was conducted from December 2024 to June 2026. Simulated wastewater was prepared and tested in the laboratory. Actual water samples were collected from Dongting Lake, the Xiangjiang River, aquaculture wastewater from Guangxi, and the Yellow Sea. Unless otherwise specified, the sampling frequency for all degradation experiments was once every 5 minutes to monitor the degradation kinetics of the pollutants.
Data exclusions	All collected data has been included in the analysis in this paper, no data has been excluded.
Reproducibility	All experiments were conducted independently and repeated at least three times. The reported data include the mean and standard deviation.
Randomization	Randomization is not applicable to this study because all reaction conditions were fixed and precisely controlled, and remained consistent across all replicates.
Blinding	This study is a chemical treatment experiment in which all experimental conditions (e.g., oxidant dosage, pH, etc.) were controlled by instruments and preset programs. The study did not involve group allocation of experimental subjects or subjective outcome assessment; therefore, blinding was not applicable.

Did the study involve field work? ☐ Yes ☒ No

Reporting for specific materials, systems and methods

We require information from authors about some types of materials, experimental systems and methods used in many studies. Here, indicate whether each material, system or method listed is relevant to your study. If you are not sure if a list item applies to your research, read the appropriate section before selecting a response.

Materials & experimental systems

n/a	Involved in the study
☒	☐ Antibodies
☒	☐ Eukaryotic cell lines
☒	☐ Palaeontology and archaeology
☐	☒ Animals and other organisms
☒	☐ Clinical data
☒	☐ Dual use research of concern
☐	☒ Plants

Methods

n/a	Involved in the study
☒	☐ ChIP-seq
☒	☐ Flow cytometry
☒	☐ MRI-based neuroimaging

Animals and other research organisms

Policy information about studies involving animals; ARRIVE guidelines recommended for reporting animal research, and Sex and Gender in Research

Laboratory animals	This study used the AB zebrafish strain provided by the China Zebrafish Resource Center (CZRC). Prior to breeding, two healthy female fish and one male fish were placed in a spawning box to induce spawning. Embryos at 6 hours post-fertilization (hpf) were used in all exposure experiments.
Wild animals	The manuscript does not mention this information.
Reporting on sex	The manuscript does not mention this information.
Field-collected samples	The manuscript does not mention this information.
Ethics oversight	All zebrafish husbandry and experimental procedures were approved by the Research Ethics Committee of the College of Environmental Science and Engineering, Hunan University (HNU-ESELL-Z501).

Note that full information on the approval of the study protocol must also be provided in the manuscript.

Dual use research of concern

Policy information about dual use research of concern

Hazards

Could the accidental, deliberate or reckless misuse of agents or technologies generated in the work, or the application of information presented in the manuscript, pose a threat to:

No	Yes	
☒	☐	Public health
☒	☐	National security
☒	☐	Crops and/or livestock
☒	☐	Ecosystems
☒	☐	Any other significant area

Experiments of concern

Does the work involve any of these experiments of concern:

No	Yes	
☒	☐	Demonstrate how to render a vaccine ineffective
☒	☐	Confer resistance to therapeutically useful antibiotics or antiviral agents
☒	☐	Enhance the virulence of a pathogen or render a nonpathogen virulent
☒	☐	Increase transmissibility of a pathogen
☒	☐	Alter the host range of a pathogen
☒	☐	Enable evasion of diagnostic/detection modalities
☒	☐	Enable the weaponization of a biological agent or toxin
☒	☐	Any other potentially harmful combination of experiments and agents

Plants

Seed stocks	Wheat seeds and amaranth seeds
Novel plant genotypes	The manuscript does not mention this information.
Authentication	The manuscript does not mention this information.
