# Supplementary material for: Bromide-mediated enhancement of far-UVC/peracetic acid process for selective micropollutant abatement
Source: Nat Commun. 2026 Oct 8;17:11295. doi: 10.1038/s41467-026-78410-2 (PMC13722704; doi:10.1038/s41467-026-78410-2)
Supplement: Supplementary file 2 [file 41467_2026_78410_MOESM3_ESM.pdf]

Description of Additional Supplementary Files

File Name: Supplementary Data 1

Description: This file contains the atomic coordinates of the studied structures.
