# Supplementary material for: Bromide-mediated enhancement of far-UVC/peracetic acid process for selective micropollutant abatement
Source: Nat Commun. 2026 Oct 8;17:11295. doi: 10.1038/s41467-026-78410-2 (PMC13722704; doi:10.1038/s41467-026-78410-2)
Supplement: Supplementary file 3 [file 41467_2026_78410_MOESM5_ESM.pdf]

nature portfolio

Peer Review File

Bromide-Mediated Enhancement of Far-UVC/Peracetic Acid Process for Selective Micropollutant Abatement

Corresponding Author: Professor Xiaofei Tan

Parts of this Peer Review File have been redacted as indicated to maintain the confidentiality of unpublished data.

This file contains all reviewer reports in order by version, followed by all author rebuttals in order by version.

Version 0:

Reviewer comments:

Reviewer #1

(Remarks to the Author)
The manuscript presents an interesting investigation into the synergistic activation of peracetic acid and bromide by 222 nm far-ultraviolet radiation for the degradation of organic micropollutants. The reported performance, including rapid OMP removal within 10 minutes, is promising, and the mechanistic insights are valuable. However, several areas could be strengthened to enhance the novelty, and practical relevance of the work. First, to better highlight the novelty, the authors should more explicitly contextualize their findings against existing literature. The promoting effects of Br^- under UV irradiation (including 254 nm) and NO_3^- in advanced oxidation processes have been previously documented. A more detailed discussion on the unique advantages of UV_{222} over conventional 254 nm UV in Br^--containing systems would help clarify the incremental contribution of this study. Second, the experimental section could be deepened by providing mechanistic insights into the observed promoting effects of NO_3^- and the rate constant difference between UV_{222} and UV_{254}. Additional radical quantification or kinetic modeling would strengthen the conclusions regarding the superiority of UV_{222}. Finally, to improve practical relevance, the authors are encouraged to consider the impact of high Cl^- concentrations, which are common in real water matrices. Characterizing the formation of chlorinated and brominated disinfection byproducts would provide a more comprehensive assessment of the environmental safety and sustainability of the UV_{222}-AOPs system. In summary, supplementary experiments are required to address insufficient novelty and, crucially, to validate the system's applicability to real environments. Only after resolving these two core issues can the manuscript be re-evaluated for publication.

1. In Fig. 1e, why is the degradation rate of SDZ in the UV254–Br^- system higher than that under UV254 alone? Can 254 nm UV directly activate Br^- to generate bromine radicals?
2. What is the bromate yield in the UV222–PAA system? Are the toxicity assessment methods using zebrafish and wheat germ applicable for bromate evaluation?
3. Is the UV222–PB system designed for pollutant removal in seawater? How was the Cl^- concentration selected in Fig. 3? It is far lower than the actual chloride concentration in seawater.
4. This study only considers the effects of trace Br^- and Cl^- in the UV222–PAA system. However, in real seawater, the concentration of Cl^- is three orders of magnitude higher than that of Br^-. Therefore, it is necessary to comprehensively consider the quenching effects of high-concentration Cl^- on bromine radicals and $CH_3C(O)OOH$, as well as the interconversion among different halogen radicals.
5. The formation of chlorinated byproducts in real water matrices, especially seawater, should be investigated.
6. In Fig. 3, NO_3^- promotes the degradation of SDZ. However, in other UV222–PAA studies (Wat. Res. 2025, 124830), nitrate inhibited the degradation of target pollutants. In addition, in most UV222-AOPs, high concentrations of NO_3^- suppress pollutant removal, mainly due to its strong UV absorbance and low radical yield. The authors should further compare the effects of different NO_3^- concentrations on UV222, UV222–PAA, and UV222–PB.
7. What are the residual concentrations of PAA and H_2O_2 after the reaction? Can the remaining H_2O_2 continuously reduce HBrO back to Br^-?
8. In Fig. 7, the SDZ removal rate in Yellow Sea water under UV222–PB is much higher than that in deionized water. It is worth clarifying whether the dominant reactive species in Yellow Sea water is Cl^--derived or Br^--derived. The authors should include additional experiments on SDZ degradation in the UV222–PAA–Cl^- (0.5 M) system.
9. Is the enhanced SDZ removal in Yellow Sea water attributed to the formation of chlorine radicals? Could bromine radicals

be scavenged by Cl^-? Moreover, would the shift in dominant halogen radical species also promote the removal of other pollutants beyond SDZ?

Reviewer #2

(Remarks to the Author)
This work developed a novel far-ultraviolet-based water treatment process, using various analytical techniques to comprehensively reveal the hidden synergistic mechanism and ecotoxicological effects between peracetic acid (PAA) and bromide under far-UV irradiation. Compared with conventional UV254, UV222 more effectively activates PAA and bromide, enabling ultrafast pollutant degradation at relatively safe UV doses. The core mechanism involves the efficient generation of highly reactive organic radicals and HBrO in the UV222-PAA/Br^- system, which drives the oxidative degradation. This system exhibits excellent selective oxidation toward electron-rich pollutants and maintains strong performance across diverse real water matrices. Multiple toxicity assessments confirm that the resulting disinfection byproducts remain within safe limits, highlighting advantages including low energy consumption, high efficiency, and practical application potential. Overall, the work addresses a novel and interesting topic with clear logic, detailed data, and comprehensive analysis, offering insightful strategies for key challenges in water treatment. Well-designed figures and tables also enhance readability. It is recommended that this manuscript can be accepted in Nature Communications after addressing these minor issues.

(1) The manuscript appears to have inconsistent formatting in multiple sections, including text and equation editing. Please carefully review the entire text and make necessary corrections.
(2) The findings reveal an interesting phenomenon, bromide exhibits a catalytic effect in the UV222-PAA system. However, is the bromide concentration selected based on environmental levels? Please clarify the experimental basis for this concentration and the actual water scenario it simulates.
(3) Besides UV222 and UV254, UV365 is also a commonly used UV light source. It is recommended to supplement relevant experiments to further highlight the advantages of UV222.
(4) Line 144: “section 3.2” is confusing. Please correctly indicate where the synergistic mechanism between PAA and bromide is discussed.
(5) Lines 214-219: The hydroxyl radical probe experiment is incomplete. Please supplement the measurement of hydroxybenzoic acid.
(6) The pH values selected in this work (3 and 11) seem to lack environmental relevance. To enhance the environmental applicability of the study, it is suggested to include a pH range that is more practically meaningful.
(7) pH often alters the HOMO-LUMO energy gap of pollutants, thereby affecting their photodegradability. It is recommended that the authors supplement relevant DFT calculations to further clarify this aspect.
(8) In Fig. 3(a), the color contrast is insufficient. Please use colors with higher contrast for better distinction.
(9) Lines 408-409: LC-MS data for SDZ degradation are missing.
(10) The Discussion requires improvement. Please elaborate further on the practical environmental implications of the UV222-PB process and offer forward-looking guidance on the application prospects of far-ultraviolet technology.

Reviewer #3

(Remarks to the Author)
In the manuscript entitled "Bromide-Mediated Enhancement of Far-UVC/Peracetic Acid Process for Selective Micropollutant Abatement",the authors systematically elucidates the synergistic mechanism between bromide ions (Br^-) and peracetic acid (PAA) under far-UVC irradiation at 222 nm, confirming that the UV222-PB process achieves selective oxidative degradation of electron-rich pollutants primarily through the efficient generation of organic radicals (•RO) and hypobromous acid (HOBr). The process demonstrates stable performance across various real water matrices and significantly reduces ecotoxicity in treated water samples. Life cycle assessment further supports its environmental sustainability. This work provides a theoretical foundation and technical support for the application of far-UVC in water treatment. The following comments are offered:

1. In the introduction, when presenting the research background of the UV222 light source, the comparison with traditional mercury lamps only mentions the risk of mercury leakage. It is recommended to also discuss the differences in energy efficiency, light absorption characteristics, and practical application costs between the two light sources to further highlight the application value of UV222 as a green light source.
2. Although the research objectives are listed in the introduction,their logical connections are not clearly articulated, and the link between these objectives and the results presented in later sections is not always explicit.It is suggested to sort out the logical relationship and express it in a more hierarchical manner, such as from basic performance research to mechanism analysis, then to toxicity and practical application evaluation.
3. In the section "Effect of ultraviolet wavelength on the performance of the UV-PAA/Br process",only sulfadiazine (SDZ) was used as a model pollutant to verify the synergistic effect of UV222-PB. What is the degradation efficiency for other typical trace pollutants? It is recommended to include degradation data for one or two additional representative micropollutants to demonstrate the universality of the wavelength effect.
4. When calculating the Electrical Energy per Order (EE/O) value for economic feasibility analysis, the authors provide EE/O values for different systems but do not detail the calculation method or parameter settings.It is suggested to supplement relevant calculation details in the main text or Supplementary materials.
5. The quenching experiments have methodological limitations. For Mn2+ used as a quencher for •RO, it has not been verified whether Mn2+ undergoes side reactions under 222 nm irradiation, reacts non-specifically with PAA or Br - , or

competes for photons.Moreover, only experiments with varying Mn2+ concentrations were conducted; control experiments (e.g., in the dark without UV222) to assess potential direct interactions between Mn2+ and SDZ, PAA, or Br-are missing. Such controls are necessary to confirm the specificity of Mn2+ as a quencher.
6. The authors only specifically measured the Br- concentration at the initial stage and at different time points, but did not systematically quantify intermediate reactive bromine species or transformation products involved in the bromine cycle.Moreover, the proposed dynamic Br- cycling process is inferred rather than quantitatively tracked. Since this cycling is central to explaining why trace amounts of Br- significantly enhance degradation efficiency, the lack of comprehensive bromine speciation and quantitative tracking weakens the evidence supporting this core mechanism.
7. In investigating the effect of pH on the UV222-PB system, only three pH values (3, 7, and 11) were tested. It is suggested to add more gradient pH points in the neutral to alkaline range to more accurately identify the optimal pH range of the system and the critical pH value for performance changes, making the conclusion more precise.
8. When examining the impact of inorganic anions, the authors analyzes the promoting or inhibiting effects of Cl−,NO3−, SO42−and CO32−, but does not consider the simultaneous presence of multiple anions in real water matrices. It is suggested to add experiments with mixed anions to better simulate realistic water conditions and evaluate their combined effects on system performance.
9. In the sectionr "Analysis of pollutant degradation pathways and systemic toxicity assessment", density functional theory (DFT) calculations provide the energy barriers for HOBr generation and organic radical reactions.Were temperature effects considered in these calculations?If not, it is suggested to add relevant discussion or explain why temperature factors were not considered in the experiment.
10. In the toxicity assessment, the zebrafish embryo culture experiment only records hatching status.It does not evaluate teratogenic rate, types of malformations, or post-hatching larval survival. It is suggested to add relevant toxicological indicators to make the toxicity assessment more comprehensive and in-depth.
11. The manuscript contains several grammatical errors and awkward phrasings (e.g., line471:" UV222-PB the survival rates of wheat seeds were 90%, 60%, 70%, 85%, and 90%,"). Additionally, some abbreviations are not defined at their first appearance in the text (e.g., SRHA, SRFA, NOM). A thorough revision of the language and formatting is recommended to ensure clarity and adherence to standard academic writing conventions.

Version 1:

Reviewer comments:

Reviewer #1

(Remarks to the Author)
The authors are appreciated for their efforts in responding to the previous comments and making corresponding revisions. Nevertheless, there remain several important issues, particularly those related to the core reaction mechanisms, that would benefit from further clarification.
1. The authors indicated that •BrCl reacts rapidly with Br^- to generate •Br, so •Br is the main reactive species. However, as shown in Figure 7e, the contribution of •BrCl to SDZ removal is only slightly lower than that of •Br. In Supplementary Table 18, the authors determined the second-order rate constants of SDZ with $•Br_2^-$ and $•BrCl^-$ as 1.53×10^8 $M^{-1}·s^{-1}$ and 1.78×10^8 $M^{-1}·s^{-1}$, respectively. The second-order rate constant for the reaction of $•BrCl^-$ with Br^- to generate $•Br_2^-$ is 8.00×10^9 $M^{-1}·s^{-1}$. Moreover, in the reaction, the concentration of Br^- is 25 times that of SDZ, so $•BrCl^-$ should be rapidly scavenged. Then what is the reason for the high contribution proportion of $•BrCl^-$ in Figure 7e?
2. The degradation rate of SDZ by UV_{222}-PB is much higher than that by UV_{222}-Br^-, and bromine radicals are the main factor for degradation. Therefore, is the main source of bromine radicals the photolysis of HBrO by UV_{222}? What is the steady-state concentration of HBrO in the reaction system? Could you supplement the degradation experiment of SDZ by UV_{222}/HBrO to better understand the reaction mechanism?
3. How long does it take for PAA + Br^- to generate HBrO? Is it necessary to conduct a dark reaction before UV_{222} irradiation?
4. Does the H_2O_2 content in PAA affect the performance of UV_{222}-PB? HBrO can be reduced by H_2O_2, and the photolysis of H_2O_2 contributes little to SDZ degradation.
5. In the control experiments, the degradation of SDZ by the dark reaction of PAA + Br^- should be supplemented to evaluate the oxidizing capacity of HBrO.
6. In the LCA evaluation, is it appropriate to use the degradation data from batch reactions (50 mL SDZ) as input parameters? Moreover, the LCA assessment only compares different combinations of UV_{222}, PAA, and Br^-, which is somewhat one-sided; it should be compared with conventional seawater treatment technologies.
7. What is the degradation rate of SDZ in the continuous-flow experiment? Why were the batch experiments chosen for the LCA assessment instead of the continuous-flow experiment? In the continuous experiment, Br^- should be pre-mixed with SDZ rather than injected separately like PAA, because Br^- is a naturally coexisting inorganic ion in the water to be treated (seawater).
8. The authors seem to have only provided a method for correcting light intensity, but not a method for measuring it. Please supplement the emission spectrum diagram with information on UV_{365}.

Reviewer #2

(Remarks to the Author)
The author's response meets all my concerns, addressing each point with clarity and thoroughness, demonstrating a deep understanding of the subject matter. The revisions made to the manuscript significantly enhance its quality and rigor, making

it a valuable contribution to the field. Given the importance of the research findings and the meticulous attention to detail in the author's revisions, I believe this manuscript is now well-suited for publication in Nature Communications.

Reviewer #3

(Remarks to the Author)
The authors of the manuscript titled "Bromide-Mediated Enhancement of Far-UVC/Peracetic Acid Process for Selective..." have appropriately revised the paper in response to the reviewers' comments, and the revised version now meets the requirements of Nature Communications. I recommend acceptance of this paper.

Version 2:

Reviewer comments:

Reviewer #1

(Remarks to the Author)
The authors have revised and supplemented the manuscript according to the comments.

Open Access This Peer Review File is licensed under a Creative Commons Attribution 4.0 International License, which permits use, sharing, adaptation, distribution and reproduction in any medium or format, as long as you give appropriate credit to the original author(s) and the source, provide a link to the Creative Commons license, and indicate if changes were made.
In cases where reviewers are anonymous, credit should be given to 'Anonymous Referee' and the source.
The images or other third party material in this Peer Review File are included in the article's Creative Commons license, unless indicated otherwise in a credit line to the material. If material is not included in the article's Creative Commons license and your intended use is not permitted by statutory regulation or exceeds the permitted use, you will need to obtain permission directly from the copyright holder.
To view a copy of this license, visit https://creativecommons.org/licenses/by/4.0/

Responses to Reviewers' Comments

Journal: Nature Communications

Manuscript Number: NCOMMS-26-008494A

Title: Bromide-Mediated Enhancement of Far-UVC/Peracetic Acid Process for Selective Micropollutant Abatement

Author(s): Genying Zeng [a b], Guoyu Li [a], Hou Wang [a], Chen Zhang [a], Qiang Chen [a b], Qinyuan Tang [a b], Tangshan Wu [a b], Li Li [c], Xinjiang Hu [d *], Ran Yin [e f *], Xiaofei Tan [a b *]

General note: Comments from reviewers are presented in black *italic* style. Responses to each comment are in blue and regular style. "Revised text as it appears in the manuscript" is indented and in red.

Reviewer #1

General Comments: *The manuscript presents an interesting investigation into the synergistic activation of peracetic acid and bromide by 222 nm far-ultraviolet radiation for the degradation of organic micropollutants. The reported performance, including rapid OMP removal within 10 minutes, is promising, and the mechanistic insights are valuable. However, several areas could be strengthened to enhance the novelty, and practical relevance of the work. First, to better highlight the novelty, the authors should more explicitly contextualize their findings against existing literature. The promoting effects of* Br^- *under UV irradiation (including 254 nm) and* NO_3^- *in advanced oxidation processes have been previously documented. A more detailed discussion on the unique advantages of* UV_{222} *over conventional 254 nm UV in* Br^-*-containing systems would help clarify the incremental contribution of this study. Second, the experimental section could be deepened by providing mechanistic insights into the observed promoting effects of* NO_3^- *and the rate constant difference between* UV_{222} *and* UV_{254}*. Additional radical quantification or kinetic modeling would strengthen the conclusions regarding the superiority of* UV_{222}*. Finally, to improve practical relevance, the authors are encouraged to consider the impact of high* Cl^- *concentrations, which are common in real water matrices. Characterizing the formation of chlorinated and brominated disinfection byproducts would provide a more comprehensive assessment of the environmental safety and sustainability of the* UV_{222}*-AOPs system. In summary, supplementary experiments are required to address insufficient novelty and, crucially, to validate the system's applicability to real environments. Only after resolving these two core issues can the manuscript be re-evaluated for publication.*

Response: We sincerely appreciate your recognition of our research achievements and for your thorough and insightful comments on our manuscript. These comments have helped us identify the shortcomings of our work and have provided valuable guidance for improving its quality. In response to the three core issues you raised, novelty,

mechanistic depth, and practical application relevance, we have supplemented the manuscript with a series of experiments and analyses covering theoretical calculations, process performance, identification of disinfection byproducts, and validation of practical applications. Specifically, we have added a more detailed comparison between UV_{222} and conventional UV_{254} in the Introduction to highlight the unique advantages of UV_{222} (**Lines 57–77**). Moreover, we employed time-dependent density functional theory (TDDFT) to calculate the excited states of Br^-, SDZ, and their combined states (**Lines 201–223**). We determined the formation of bromate (**Lines 548–555**) and disinfection byproducts under different process conditions (**Lines 570–582**). We investigated the stability of the UV_{222}-PB process under high Cl^- conditions and its degradation efficiency for multiple micropollutants in real seawater, and we elucidated the reactive species in the high-Cl^- system through kinetic modeling (**Lines 730–826**). Furthermore, we verified the ecological safety and environmental applicability of the treated water samples through methods such as constructing a mixed anion system covering a wide salinity range (**Lines 675–690**), employing pH experimental conditions that more closely resemble actual environments (**Lines 584–621**), and conducting more detailed zebrafish embryo cultivation experiments (**Lines 521–534**). Detailed revisions are provided in the point-by-point responses below. Your valuable comments are crucial for the improvement of our manuscript, and we sincerely hope that our revisions meet your expectations. Once again, we extend our heartfelt thanks to you. Your professional and profound insights have set an exemplary standard for our future research.

Comment 1: In Fig. 1e, why is the degradation rate of SDZ in the UV_{254}–Br^- system higher than that under UV_{254} alone? Can 254 nm UV directly activate Br^- to generate bromine radicals?

Response: We greatly appreciate your critical question, which has helped us better clarify the role of Br^- in the UV_{254} system. In response to your question, we calculated the excitation wavelengths of Br^-, SDZ, and their combined states using time-dependent density functional theory (TDDFT). The results show that the effective excitation wavelength of $Br^-(S_1)$ is 207 nm (5.99 eV), whereas the photon energy at 254 nm is only 4.88 eV, so direct excitation of Br^- to generate bromine radicals is not possible. However, charge transfer analysis revealed that SDZ could serve as a potential electron acceptor. Specifically, we further calculated the interaction between SDZ and Br^- at different adsorption sites. When the adsorption site is located on the benzene or pyrimidine ring of SDZ, a photoinduced HOMO–LUMO electron transition accompanied by charge transfer from Br^- to SDZ can be observed. Free energy calculations further confirmed that the reaction between excited-state SDZ (S_1) and Br^- to generate •Br can proceed spontaneously. In summary, the enhanced pollutant degradation efficiency in the UV_{254}-Br system is attributed to electron transfer from photoexcited SDZ rather than direct photolysis of Br^-. The relevant description appears on **Lines 201–223** of the manuscript, in **Supplementary Fig. 7**, and in **Supplementary Tables 4–8**. We would like to thank you once again for your thorough and professional review of our manuscript, which has greatly improved its quality.

The related descriptions and images are presented below for your convenience:

Lines 201–223: "To further explore the mechanism of Br^- at different wavelengths, the study calculated the excited-state properties of Br^- and SDZ using time-dependent density functional theory (TDDFT). The results show that the effective excitation wavelength of $Br^-(S_1)$ is 207.07 nm (5.99 eV), while the photon energies at 222 nm (5.59 eV) and 254 nm (4.88 eV) are both lower than this value; therefore, it is difficult to directly photoexcite Br^- to generate reactive bromine species (Supplementary Fig. 7a and Supplementary Table 4). In contrast, the calculation results for SDZ indicate that

light with a wavelength shorter than 264.92 nm can excite it to an excited state; thus, both 222 nm and 254 nm can effectively excite SDZ (Supplementary Fig. 7b and Supplementary Table 5). According to previous reports, electron-rich pollutants can act as potential direct electron donors[29]. Therefore, the study further calculated the interaction between SDZ and Br^- at different adsorption sites (Supplementary Figs. 7c–e and Supplementary Tables 6–8). Obviously, when the complexation site of Br^- with SDZ is located on the benzene ring or pyrimidine ring of SDZ, photoexcitation can induce an electron transition from the highest occupied molecular orbital (HOMO) to the lowest unoccupied molecular orbital (LUMO), accompanied by charge transfer from Br^- to SDZ (Supplementary Fig. 7g). Furthermore, free energy calculations indicate that ground-state SDZ (S_0) cannot spontaneously react with Br^- to form •Br (4.02 eV), whereas the free energy change for the reaction between excited-state SDZ (S_1) and Br^- is negative (-0.0055 eV), which is thermodynamically favorable (Supplementary Fig. 7f). This suggests that both 222 nm and 254 nm light excitation can drive electron transfer between SDZ and Br^-, thereby effectively generating •Br."

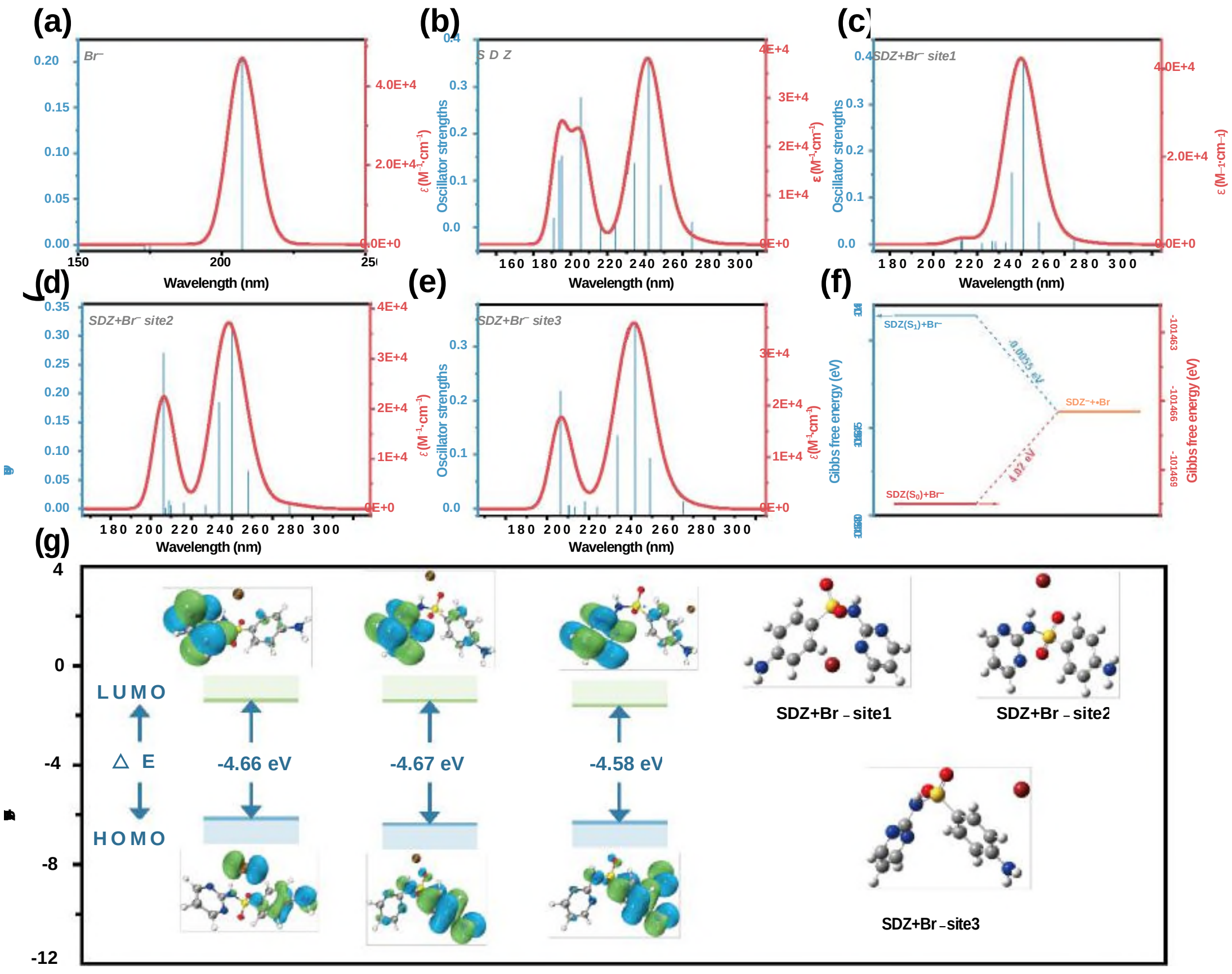

Supplementary Fig. 7 | Oscillator strengths and molar absorption coefficients (ε) for (a) Br^-, (b) SDZ, (c) SDZ+Br^- site1, (d) SDZ+Br^- site2, and (e) SDZ+Br^- site3. (f) Gibbs free energy changes for the reaction of SDZ with Br^- under different electronic states. (g) HOMO and LUMO of SDZ and Br^- at different complexation sites.

Supplementary Table 4 | The vertical excitation energy, excitation wavelength, and oscillator strength of Br^-.

Excited state	Vertical excitation energy	Wavelength	Oscillator strengths
S1	5.9875 eV	207.07 nm	f= 0.2035
S2	5.9875 eV	207.07 nm	f= 0.2035
S3	5.9875 eV	207.07 nm	f= 0.2035
S4	7.0840 eV	175.02 nm	f= 0.0000
S5	7.0840 eV	175.02 nm	f= 0.0000

Supplementary Table 5 | The vertical excitation energy, excitation wavelength, and oscillator strength of SDZ.

Excited state	Vertical excitation energy	Wavelength	Oscillator strengths
S1	4.6800 eV	264.92 nm	f= 0.0122
S2	4.9941 eV	248.26 nm	f= 0.0916
S3	5.1301 eV	241.68 nm	f= 0.3632
S4	5.3016 eV	233.86 nm	f= 0.1365
S5	5.5286 eV	224.26 nm	f= 0.0032
S6	5.7359 eV	216.15 nm	f= 0.0102
S7	6.0301 eV	205.61 nm	f= 0.2767
S8	6.3428 eV	195.47 nm	f= 0.1523
S9	6.3973 eV	193.81 nm	f= 0.1436
S10	6.4902 eV	191.03 nm	f= 0.0198

Supplementary Table 6 | The vertical excitation energy, excitation wavelength, and oscillator strength of SDZ+Br^- site1.

Excited state	Vertical excitation energy	Wavelength	Oscillator strengths
S1	4.6948 eV	264.09 nm	f= 0.0087
S2	4.9994 eV	248.00 nm	f= 0.0472
S3	5.1450 eV	240.98 nm	f= 0.3990
S4	5.2586 eV	235.77 nm	f= 0.1528
S5	5.3239 eV	232.88 nm	f= 0.0031
S6	5.4285 eV	228.39 nm	f= 0.0051
S7	5.4707 eV	226.63 nm	f= 0.0061
S8	5.5791 eV	222.23 nm	f= 0.0018
S9	5.8211 eV	212.99 nm	f= 0.0096
S10	5.8309 eV	212.63 nm	f= 0.0083

Supplementary Table 7 | The vertical excitation energy, excitation wavelength, and oscillator strength of SDZ+Br^{-} site2.

Excited state	Vertical excitation energy	Wavelength	Oscillator strengths
S1	4.6191 eV	268.42 nm	f= 0.0113
S2	4.9972 eV	248.11 nm	f= 0.0656
S3	5.1664 eV	239.98 nm	f= 0.3229
S4	5.3088 eV	233.54 nm	f= 0.1840
S5	5.4659 eV	226.83 nm	f= 0.0053
S6	5.7333 eV	216.25 nm	f= 0.0102
S7	5.9069 eV	209.90 nm	f= 0.0062
S8	5.9369 eV	208.84 nm	f= 0.0142
S9	5.9818 eV	207.27 nm	f= 0.0010
S10	6.0124 eV	206.21 nm	f= 0.2706

Supplementary Table 8 | The vertical excitation energy, excitation wavelength, and oscillator strength of SDZ+Br^{-} site3.

Excited state	Vertical excitation energy	Wavelength	Oscillator strengths
S1	4.6768 eV	265.10 nm	f= 0.0135
S2	4.9749 eV	249.22 nm	f= 0.0941
S3	5.1245 eV	241.94 nm	f= 0.3433
S4	5.3028 eV	233.81 nm	f= 0.1351
S5	5.5339 eV	224.04 nm	f= 0.0033
S6	5.6823 eV	218.19 nm	f= 0.0130
S7	5.8166 eV	213.16 nm	f= 0.0030
S8	5.8878 eV	210.58 nm	f= 0.0065
S9	5.8980 eV	210.21 nm	f= 0.0055
S10	6.0032 eV	206.53 nm	f= 0.2181

Comment 2: What is the bromate yield in the UV_{222}–PAA system? Are the toxicity assessment methods using zebrafish and wheat germ applicable for bromate evaluation?

Response: Thank you very much for your concerns regarding bromate formation and the toxicity assessment methods. The two issues are very important for improving our manuscript. In response to your questions, we determined the bromate concentration in different processes using ion chromatography (IC, AQUION). The results showed that no bromate was detected in processes such as UV_{222}-Br and UV_{222}-PB. This is attributed to the relatively low concentration of •OH in the system, which is insufficient to further oxidize reactive bromine species to bromate. Moreover, coexisting substances such as H_2O_2 reduce HOBr and other intermediates back to Br^-, thereby blocking the bromate formation pathway.

On the other hand, the zebrafish embryo culture and wheat seed germination toxicity tests were employed to evaluate the overall ecotoxicity of the treated water, rather than specifically targeting bromate. These bioassays reflect the combined toxic effects of all residual pollutants, transformation products, and disinfection byproducts (including any bromate that might be formed). Since the bromate concentration in our system is negligible, its contribution to the observed toxicity is minimal. Therefore, the zebrafish and wheat seed tests remain valid and appropriate for assessing the overall environmental safety of the treated water. In addition, we supplemented the toxicity assessment with additional endpoints from zebrafish embryo culture, including malformation rate and hatching rate, to further improve the toxicity evaluation of this study. The bromate measurement data and the zebrafish culture experiments have been added to the revised manuscript (**Lines 548–555**, **Lines 521–534**, **Supplementary Fig. 23** and **Figs. 5d–f**). Once again, we thank you for your careful review and valuable comments on our manuscript, from which we have greatly benefited.

The related descriptions and images are presented below for your convenience:

Lines 548–555: "Therefore, this study evaluated the formation of bromate in different systems. The results showed that no bromate was detected in any of the four systems, namely UV_{254}-Br, UV_{254}-PB, UV_{222}-Br, and UV_{222}-PB (Supplementary Fig.

23). This is because the generation concentration of •OH in these systems is limited, insufficient to further oxidize reactive bromine species to bromate[56]. At the same time, coexisting substances such as H_2O_2 reduce HOBr and other intermediates back to Br^-, thereby blocking the bromate formation pathway."

Lines 521–534: "The results of the zebrafish embryo culture experiment showed that, except for the blank control group, all treatment groups exhibited varying degrees of morphological abnormalities. Among them, the SDZ treatment group showed the highest malformation rate, reaching 100% at 96 hours post-fertilization (hpf). Zebrafish exposed to the original SDZ solution began to show morphological malformations such as pericardial edema (PE), eye deformation (ED), and yolk sac edema (YSE) at 72 hpf (Fig. 5d), indicating that the SDZ solution has high animal toxicity. Zebrafish cultured in solutions treated with the UV_{222}-PAA and UV_{222}-Br processes also exhibited PE developmental abnormalities at 72 hpf. Compared with the original SDZ group, both the survival rate and hatching rate of zebrafish at 96 hpf were improved after treatment with the various UV_{222}-based processes (Figs. 5e–f). Among them, the UV_{222}-PB process exhibited the most significant detoxification effect, with its hatching rate at 96 hpf being only 4.17 % lower than that of the blank control group."

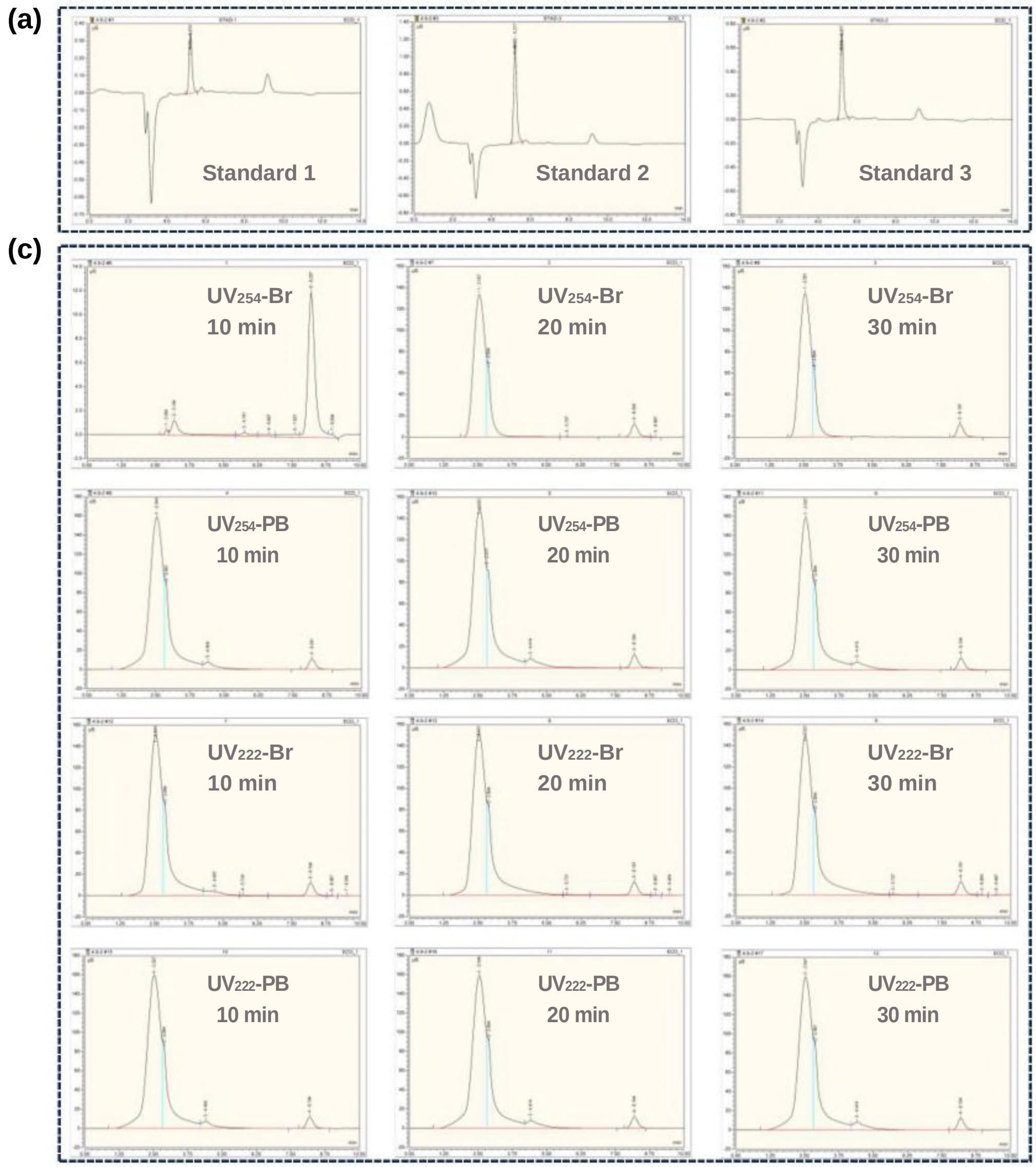

Supplementary Fig. 23 | (a) BrO_3^- standard sample detected by IC. (b) Ion chromatograms of BrO_3^- in the UV_{254}-Br, UV_{254}-PB, UV_{222}-Br, UV_{222}-PB systems at different time points.

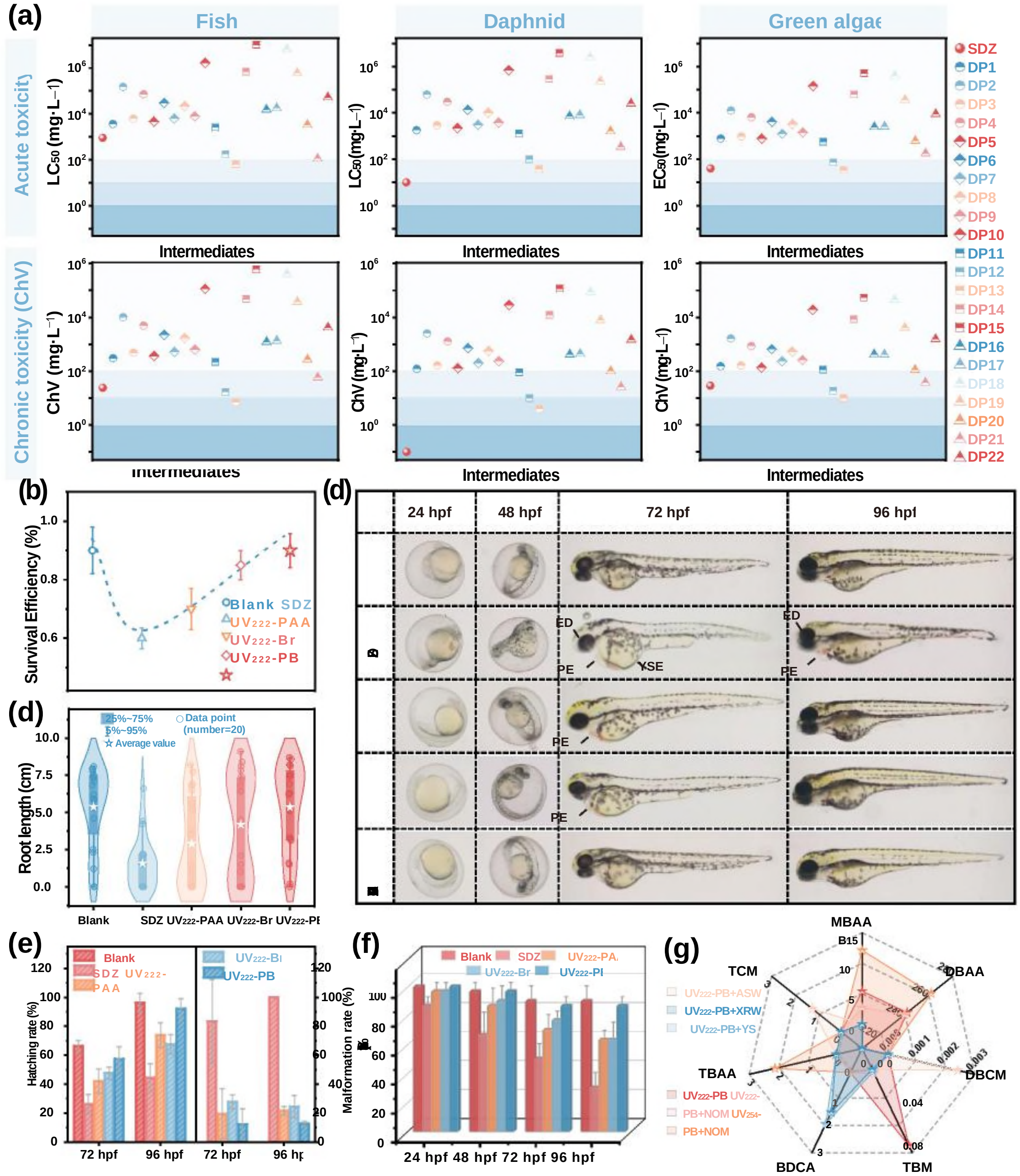

Fig. 5 | Multi-model toxicological effect assessment. (a) Acute and chronic toxicity of SDZ and its intermediates. In different treatment conditions (b) wheat germination rates, (c) violin diagram of wheat bud length distribution and zebrafish embryo (d) development, (e) hatching rates, (f) survival rates. (g) The amount of DBPs produced in different processes.

Comment 3: Is the UV_{222}-PB system designed for pollutant removal in seawater? How was the Cl^- concentration selected in Fig. 3? It is far lower than the actual chloride concentration in seawater.

Response: Thank you very much for your thorough review of our manuscript and for raising this critical question. The UV_{222}-PB process is primarily designed for the removal of micropollutants from high-salinity water matrices, including seawater, brackish water, aquaculture wastewater, and water affected by seawater intrusion and backflow. In this study, the selection of the Br^- concentration was based on the typical environmental level of Br^- in seawater (0.5 mM, approximately 39.95 mg/L). Therefore, the process is clearly targeted at real high-salinity water bodies such as seawater. Furthermore, the choice of the Cl^- concentration gradient in Fig. 3 was based on mechanistic considerations. In general, Cl^- in advanced oxidation processes can either promote pollutant degradation by generating reactive chlorine species or exert an inhibitory effect by quenching radicals, and its influence does not follow a monotonic trend with increasing concentration. Hence, the Cl^- concentration gradient in Fig. 3 was designed to better reveal the mechanistic pattern and possible inflection points of Cl^- action, rather than to directly simulate the chloride level in seawater.

Nevertheless, as you pointed out, the typical concentration of Cl^- in real seawater (approximately 0.5 M) is also critically important for the UV_{222}-PB process. Therefore, following your suggestion, we conducted additional experiments with UV_{222}-PAA-Cl (UV_{222}-PC) and UV_{222}-PAA-Br-Cl (UV_{222}-PBC) to systematically evaluate the impact of high Cl^- concentration on the UV_{222}-PB process. In addition, we prepared a high-salinity simulated water (HSSW, containing 0.5 M Cl^-), and collected real water samples including Yellow Sea (YS), and aquaculture seawater wastewater (ASW). The above results are presented in **Lines 675–690, 722–749**, **Figs. 6g, 7a–d**, **Supplementary Figs. 30a–b, 31b** of the revised manuscript. Once again, we appreciate your valuable comments, which have enabled us to more comprehensively demonstrate the application potential of the UV_{222}-PB process in high-salinity water bodies.

The related descriptions and images are presented below for your convenience:

Lines 675–690: "Real water matrices often consist of multiple complex ions. Therefore, this study configured trace-salinity simulated water (TSSW), low-salinity simulated water (LSSW), medium-salinity simulated water (MSSW), and high-salinity simulated water (HSSW) to better evaluate the performance of the UV_{222}-PB process in water bodies with different salinity ranges. It can be observed that the UV_{222}-PB process exhibited good degradation performance in all four simulated water matrices, with the pollutant being almost completely degraded within 20 minutes (Fig. 6g and Supplementary Figs. 30a–b). Moreover, the performance of the UV_{222}-PB process was optimal in LSSW. Compared with TSSW, the appropriate concentrations of NO_3^-, SO_4^{2-}, and CO_3^{2-} in LSSW promoted the generation of more reactive species in the system. However, as the salinity further increased, the degradation rate of SDZ gradually decreased, with a k_{obs} value of 0.2093 min^{-1} in HSSW. This may be attributed to the increase in pH caused by the addition of a higher concentration of CO_3^{2-} as well as the light-shielding effect of NO_3^- (Supplementary Table 15). Overall, the UV_{222}-PB process can operate stably across a wide salinity range from freshwater to seawater, providing strong support for its application in practical high-salinity water bodies."

Lines 722–749: "However, in ASW (k_{obs} = 0.1507 min^{-1}) water sample, the degradation of SDZ was still prevented due to the light shielding and competition effect generated by high organic matter content, even at high Cl^- concentrations (2862 $mg \cdot L^{-1}$). Specifically, the UV_{222}-PB system performed substantially better in YS water (k_{obs} = 0.4912 min^{-1}), attaining a 99.19% SDZ degradation rate in 10 minutes. This strong promotion effect was attributed to the high concentration of Cl^- (25367 $mg \cdot L^{-1}$), resulting in a considerable promotion effect, leading to the conversion of more active species.

However, while high concentrations of Cl^- promote degradation, they may also compete with Br^- for reactive species, thereby interfering with the formation of bromine species[71]. To better clarify the dominant reactive species responsible for pollutant degradation in the UV_{222}-PB process in real seawater, we conducted a series of supplementary experiments under high Cl^- concentration (Figs. 7c–d and

Supplementary Fig. 31b). Specifically, in the UV_{222}-Cl system containing only 0.5 M Cl^-, the degradation of SDZ was 36.94%, and its k_{obs} value was only 0.47 times that of the UV_{222}-Br system. Upon the addition of PAA, although the k_{obs} value of SDZ degradation in the UV_{222}-PAA-Cl (UV_{222}-PC) system increased to 0.0457 min^{-1}, it remained much lower than that in the UV_{222}-PB process. Moreover, compared with Dark-PB, the degradation rate of SDZ in Dark-PC decreased by approximately 60.32% (Supplementary Figs. 31c–e), a difference that may be attributed to the fact that the rate constant for the reaction of PAA with Cl^- to form HOCl is about four orders of magnitude lower than that for the reaction of PAA with Br^- to form HOBr[34]. Notably, in the UV_{222}-PAA-Br-Cl (UV_{222}-PBC) system containing 0.5 M Cl^-, SDZ was already degraded by 98.12% within 15 min, and its k_{obs} value was 1.76 and 8.45 times higher than those in the UV_{222}-PB and UV_{222}-PC systems, respectively. In summary, the above experimental results demonstrate that chlorine-derived species alone cannot achieve efficient pollutant degradation, and the presence of Br^- is an indispensable part of performance enhancement.

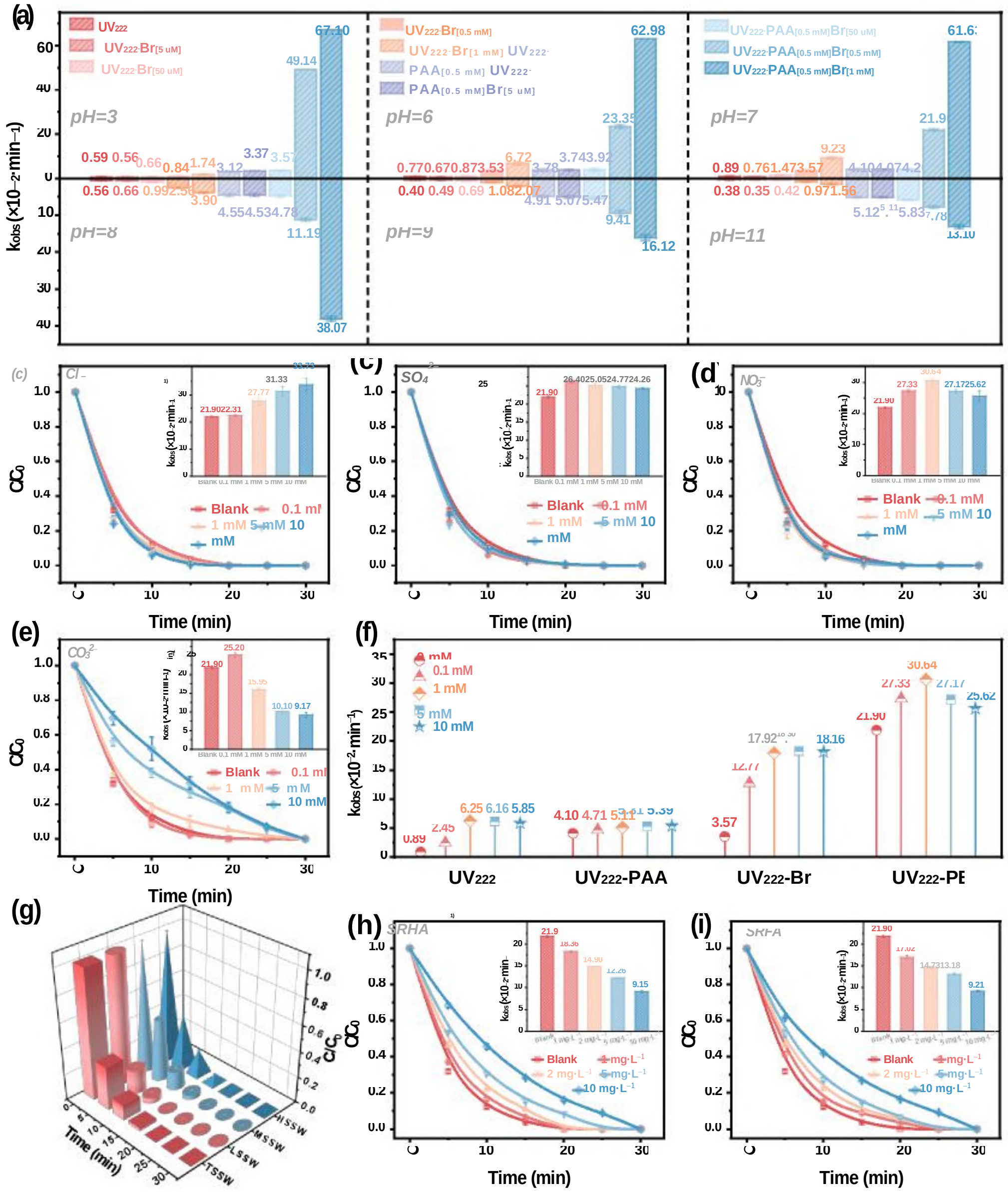

Fig. 6 | The Regulatory Effects and Mechanisms of Water Quality Parameters on SDZ Degradation. (a) k_{obs} values of SDZ in different processes at multiple pH levels. Degradation curves and k_{obs} values of SDZ degraded by UV_{222}-PB in the presence of (b) Cl^-, (c) SO_4^{2-}, (d) NO_3^-, (e) CO_3^{2-}. (f) The effect of coexisting NO_3^- on the degradation of SDZ under UV_{222} using different processes. (g) The performance of UV_{222}-PB in simulated water bodies with different salinities. Degradation curves and k_{obs} values of SDZ degraded by UV_{222}-PB in the presence of (h) SRHA and (i) SRFA.

(**Redaction**)

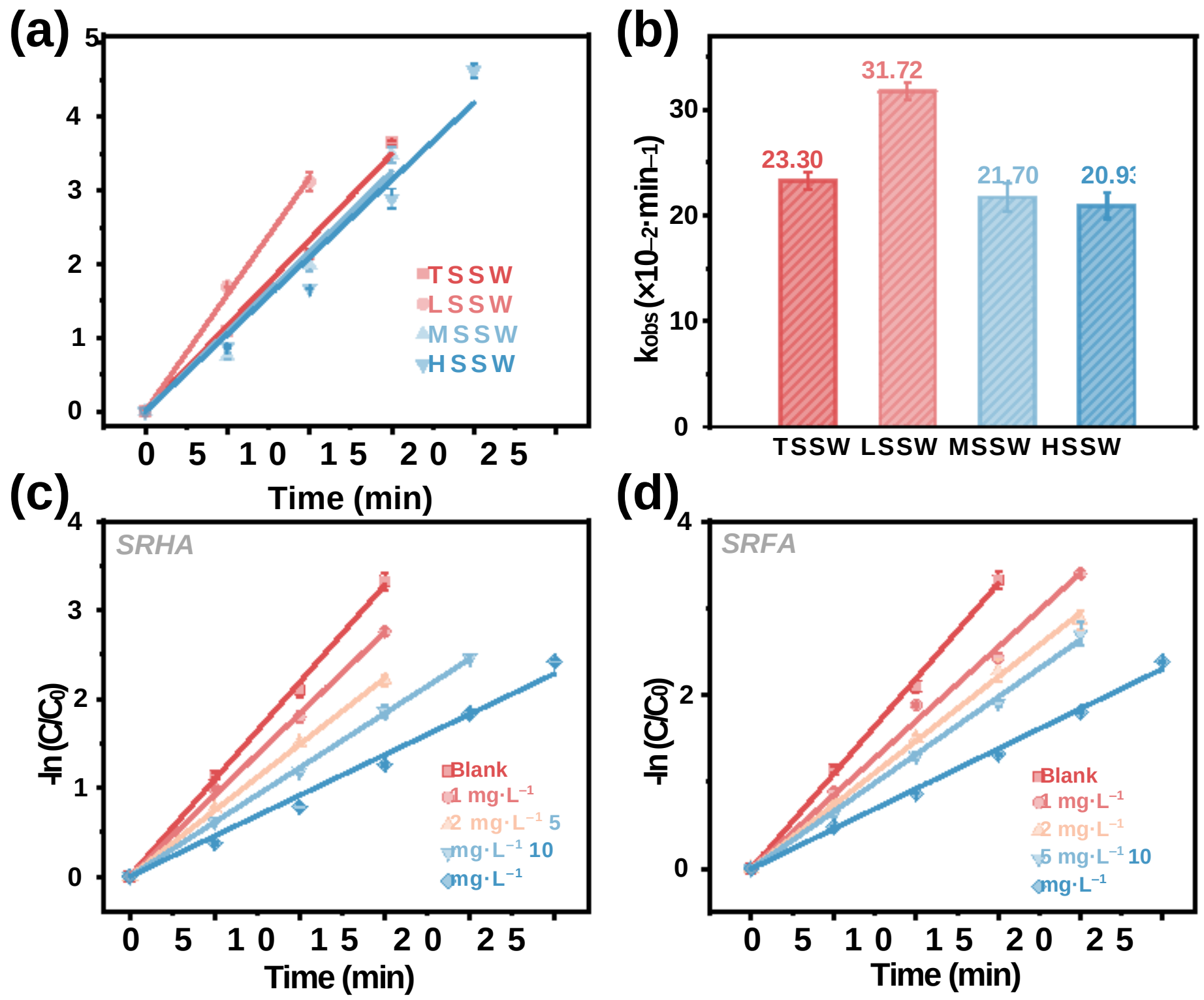

Supplementary Fig. 30 | (a) Pseudo-first-order kinetic fitting curves and (b) k_{obs} values for different simulated salinity water bodies in the UV_{222}-PB system. Pseudo-first-order kinetic fitting curves of SDZ under different concentrations of (c) SRHA and (d) SRFA in the UV_{222}-PB system. Conditions: $[SDZ]_0 = 20$ μM, $[PAA]_0 = [Br]_0 = 0.5$ mM.

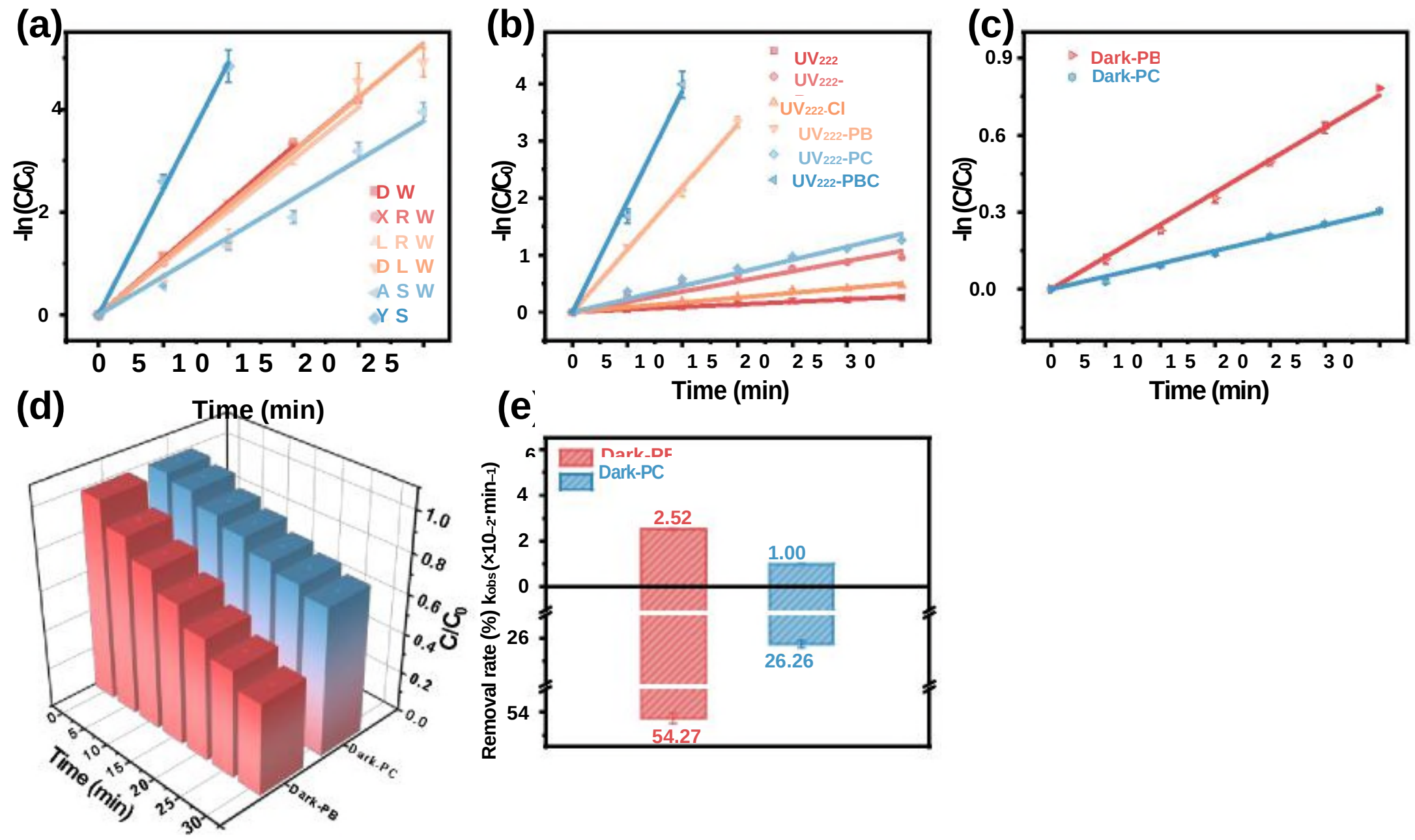

Supplementary Fig. 31 | Pseudo-first-order kinetic curves of SDZ under the UV_{222}-PB process in different real water bodies. (b) Pseudo-first-order kinetic curves of SDZ under different processes with UV_{222}. (c) Pseudo-first-order kinetic curves, (d) degradation curves, and (e) k_{obs} values for SDZ under different processing conditions in the dark. $[SDZ]_0$ = 20 μM, $[PAA]_0$ = $[Br]_0$ = 0.5 mM, $[Cl]_0$= 0.5 M.

Comment 4: This study only considers the effects of trace Br^- and Cl^- in the UV_{222}-PAA system. However, in real seawater, the concentration of Cl^- is three orders of magnitude higher than that of Br^-. Therefore, it is necessary to comprehensively consider the quenching effects of high-concentration Cl^- on bromine radicals and $CH_3C(O)OOH$, as well as the interconversion among different halogen radicals.

Response: Thank you for raising these critical questions, which are essential for evaluating the applicability of the UV_{222}-PB process in seawater. To comprehensively address your concerns, we first conducted a series of supplementary experiments under high Cl^- concentration (0.5 M). The results indicated that the degradation efficiency of SDZ was relatively low in the UV_{222}-Cl and UV_{222}-PC systems, which contain only Cl^-. however, when Br^- is introduced (UV_{222}-PBC), the degradation rate of SDZ increases

significantly. This demonstrates that chlorine-derived species alone cannot achieve efficient degradation of pollutants, and the presence of Br^- is a key factor in enhancing performance. Building on this, we further developed a kinetic model containing a high concentration of 0.5 M Cl^-, incorporating key pathways such as the quenching reactions of •OH and •Br by Cl^-, as well as the interconversion between halogen radicals. The model predictions show that although high-concentration Cl^- quenches a portion of •OH and •Br, rapid interconversion reactions continuously regenerate •Br, allowing •Br to maintain a steady-state concentration of 3.64 × 10^{-14} M. Moreover, due to the rapid reaction between the mixed halogen species $•BrCl^-$ and Br^-, $•Br_2^-$ becomes the dominant reactive species in the UV_{222}-PBC model. In addition, the rate constant for the reaction of PAA with Cl^- to form HOCl is approximately four orders of magnitude lower than that for PAA with Br^- to form HOBr, and the relatively low •OH steady-state concentration (3.13 × 10^{-17} M) in the UV_{222}-PBC system further restricts the reaction of PAA with •OH. Consequently, PAA consumption decreases in the UV_{222}-PBC model.

In summary, this study systematically investigated the quenching effects of high Cl^- on free radicals and the interconversion among halogen radicals through both experimental and kinetic modeling approaches. Detailed data have been added to **Lines 730–800** and **Fig. 7c–f** of the revised manuscript. We thank you again for your thorough review of our manuscript, which has provided valuable guidance for improving our kinetic model.

The related descriptions and images are presented below for your convenience:

Lines 730–800: "However, while high concentrations of Cl^- promote degradation, they may also compete with Br^- for reactive species, thereby interfering with the formation of bromine species[71]. To better clarify the dominant reactive species responsible for pollutant degradation in the UV_{222}-PB process in real seawater, we conducted a series of supplementary experiments under high Cl^- concentration (Figs. 7c–d and Supplementary Fig. 31b). Specifically, in the UV_{222}-Cl system containing only 0.5 M Cl^-, the degradation of SDZ was 36.94%, and its k_{obs} value was only 0.47 times that of the UV_{222}-Br system. Upon the addition of PAA, although the k_{obs} value

of SDZ degradation in the UV_{222}-PAA-Cl (UV_{222}-PC) system increased to 0.0457 min^{-1}, it remained much lower than that in the UV_{222}-PB process. Moreover, compared with Dark-PB, the degradation rate of SDZ in Dark-PC decreased by approximately 60.32% (Supplementary Figs. 31c–e), a difference that may be attributed to the fact that the rate constant for the reaction of PAA with Cl^- to form HOCl is about four orders of magnitude lower than that for the reaction of PAA with Br^- to form HOBr[34]. Notably, in the UV_{222}-PAA-Br-Cl (UV_{222}-PBC) system containing 0.5 M Cl^-, SDZ was already degraded by 98.12% within 15 min, and its k_{obs} value was 1.76 and 8.45 times higher than those in the UV_{222}-PB and UV_{222}-PC systems, respectively. In summary, the above experimental results demonstrate that chlorine-derived species alone cannot achieve efficient pollutant degradation, and the presence of Br^- is an indispensable part of performance enhancement.

Furthermore, to further elucidate the attribution and interaction of dominant reactive species in the UV_{222}-PB process under high chloride concentration, we introduced 0.5 M Cl^- into the original kinetic model and constructed the UV_{222}-PBC model to better simulate seawater and seawater-affected high-salinity water bodies. Remarkably, the terminal contribution analysis of the UV_{222}-PBC model revealed that RBS (66.02%) contributed far more to SDZ degradation than RCS (1.55%), with $•Br_2^-$ becoming the dominant reactive species, contributing as much as 48.08% (Figs. 7e–f and Supplementary Fig. 32a–e). This phenomenon is attributed to the rapid reaction of •Cl and $•Cl_2^-$ with Br^- in the solution to form $•BrCl^-$, resulting in low steady-state concentrations of chlorine radicals. Although the steady-state concentration of $•Cl_2^-$ ($3.83×10^{-14}$ M) is of the same order of magnitude as that of •Br ($3.64×10^{-14}$ M), its reaction rate with SDZ is approximately three orders of magnitude lower. Moreover, the steady-state concentration of •Cl is even lower, making the direct contribution of chlorine radicals to SDZ degradation very weak. Furthermore, although HOCl is present in high abundance, its reactivity toward SDZ is relatively low. Additionally, among the various transformation pathways involving $•BrCl^-$, its further reaction with Br^- to form $•Br_2^-$ ($k = 8×10^9$ $M^{-1}·s^{-1}$) exhibits the highest rate, leading to an

approximately 181% increase in the steady-state concentration of $•Br_2^-$ in the UV_{222}-PBC model compared to the UV_{222}-PB model. Moreover, the relatively low H+ concentration in the neutral environment limits the conversion of $•BrOH^-$ to •Br, and the high concentration of Cl^- in the solution also consumes •Br, resulting in a steady-state concentration of •Br that was approximately 5.6 times lower than that observed with UV_{222}-PB. Consequently, multiple factors together cause $•Br_2^-$ to far exceed •Br in its contribution to SDZ degradation, becoming the dominant reactive bromine species in the UV_{222}-PBC system.

On the other hand, the rapid interconversion among halogen reactive species drives the dynamic evolution of the compositional structure of reactive species in the system. Accordingly, the model further quantified the steady-state concentrations and reaction contributions of mixed bromine-chlorine species (e.g., $•BrCl^-$, BrCl, BrOCl). Due to the relatively low reaction rate between HOBr and HOCl, the formation concentration of BrOCl is extremely low, rendering its contribution to SDZ degradation negligible. Compared with the UV_{222}-PB model, the steady-state concentrations of •Br and $•BrOH^-$ in the UV_{222}-PBC model decrease significantly, a phenomenon attributed to the rapid reaction of these species with high-concentration Cl^-, leading to the formation of mixed halogen species such as $•BrCl^-$. Furthermore, although the steady-state concentrations of BrCl and $•BrCl^-$ are comparable, their reaction rates with SDZ differ by approximately five orders of magnitude, resulting in a marked difference in their contributions to SDZ degradation. Notably, the model predictions show that compared with UV_{222}-PB, PAA consumption decreases in the UV_{222}-PBC model, while H_2O_2 consumption increases (Supplementary Figs. 32f–g). This is because the steady-state concentration of •OH in the UV_{222}-PBC system decreases to 3.13×10^{-17} M, which hinders the reaction of PAA with •OH, leading to an 89% decrease in the steady-state concentration of $•CH_3C(O)OO$. This is the main reason for the significant reduction in the contribution of $•CH_3C(O)OO$ in the UV_{222}-PBC model. Meanwhile, H_2O_2 in the UV_{222}-PBC system is required not only to reduce HOBr but also to react with HOCl, resulting in increased H_2O_2 consumption. Nevertheless, the Br^-

concentration remains largely unchanged during the reaction, confirming the robust operation of the bromine cycle under high-chloride conditions. In summary, although Cl^- concentration is overwhelmingly dominant, Br^- still plays an indispensable role in the UV_{222}-PBC system, allowing bromine and chlorine species to jointly dominate the oxidative degradation of pollutants."

(Redaction)

Fig. 7 | Sustainability and practical application assessment. (a) Degradation curves, (b) removal rates and k_{obs} of SDZ under UV_{222}-PB treatment in different real water conditions. (c) Degradation curves, (d) and removal rates and k_{obs} of SDZ under

different UV_{222} treatment conditions. In the UV_{222}-PBC model, (e) the effect of various active substances on SDZ removal and (f) the terminal contribution. (g) k_{obs} values for the degradation of various OMPs under the UV_{222} process. (h) Removal efficiency and schematic diagram of SDZ in continuous operation experiment. (i) The relative environmental impact of 18 environment-related descriptors with 50 mL SDZ (20 μM) treated by different systems. Conditions: $[OMPs]_0$ = 20 μM, pH = 7.0, $[PAA]_0 = [Br]_0$ = 0.5 mM, $[Cl]_0$= 0.5 M.

Comment 5: The formation of chlorinated byproducts in real water matrices, especially seawater, should be investigated.

Response: Thank you for providing valuable feedback on the completeness and accuracy of our manuscript. Following your important suggestion, we selected three representative water samples covering different salinity gradients, namely Xiangjiang River water (XRW), Yellow Sea seawater (YS), and aquaculture seawater wastewater (ASW). The formation concentrations of chlorinated disinfection byproducts during the UV_{222}-PB process in these real water matrices were determined using GC-ECD. The detailed results have been added to the revised manuscript (**Lines 570–582** and **Fig. 5g**). The results indicate that the UV_{222}-PB process produces low concentrations of DBPs in actual water matrices. These findings further demonstrate the environmental safety and application potential of the UV_{222}-PB process in real water matrices. Once again, we appreciate your guidance, which has significantly enhanced the practical applicability of this work.

The related descriptions and images are presented below for your convenience:

Lines 570–582: "It is noteworthy that the UV_{222}-PB process exhibited low concentrations of DBPs in real water matrices such as Xiangjiang River water (XRW), Yellow Sea (YS), and Aquaculture Seawater Wastewater (ASW). Specifically, Cl^- present in real water environments can react with PAA to generate HOCl, which is also an important precursor of DBPs[59]. However, compared with HOBr, HOCl has weaker electrophilicity and thus a lower potential to form DBPs[60]. Consequently, only low

concentrations of chlorinated DBPs (Cl-DBPs), such as trichloromethane (TCM), dibromochloromethane (DBCM), and bromodichloroacetic acid (BDCA), were detected in real water matrices. On the other hand, components such as NOM, HCO_3^-, and nitrite in real water can competitively consume bromine species[61,62]. Furthermore, DOM in the solution can convert •Br and $•Br_2^-$ back to Br^- via single electron transfer[63], thereby hindering the formation of RBS and subsequently suppressing the formation of Br-DBPs."

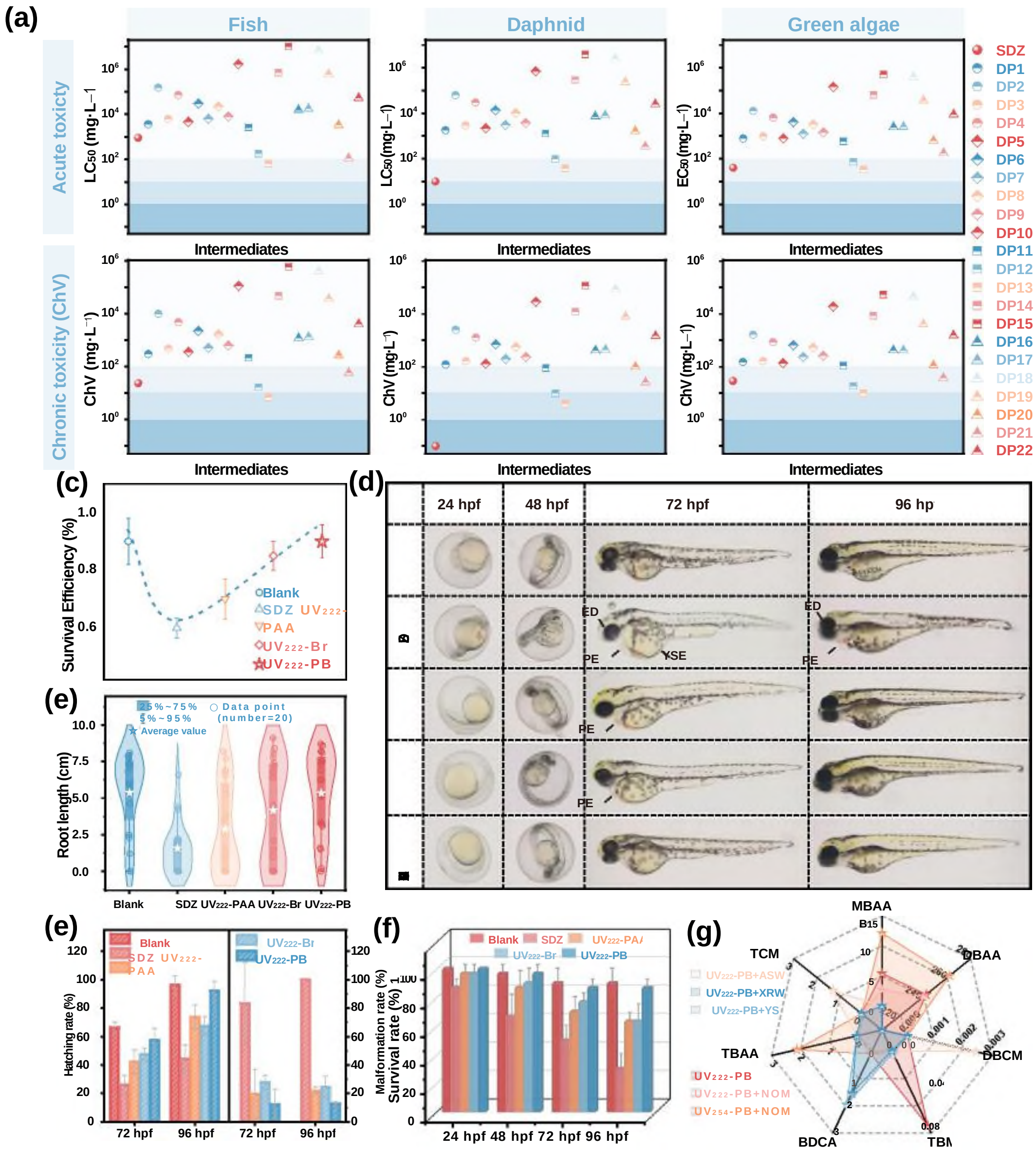

Fig. 5 | Multi-model toxicological effect assessment. (a) Acute and chronic toxicity

of SDZ and its intermediates. In different treatment conditions (b) wheat germination rates, (c) violin diagram of wheat bud length distribution and zebrafish embryo (d) development, (e) hatching rates, (f) survival rates. (g) The amount of DBPs produced in different processes.

Comment 6: In Fig. 3, NO_3^- promotes the degradation of SDZ. However, in other UV_{222}–PAA studies (Wat. Res. 2025, 124830), nitrate inhibited the degradation of target pollutants. In addition, in most UV_{222}-AOPs, high concentrations of NO_3^- suppress pollutant removal, mainly due to its strong UV absorbance and low radical yield. The authors should further compare the effects of different NO_3^- concentrations on UV_{222}, UV_{222}–PAA, and UV_{222}–PB.

Response: We sincerely appreciate your insightful feedback regarding the shortcomings in our discussion of the mechanism of action of NO_3^-. In response to your valuable comments, we investigated the effects of different NO_3^- concentrations (0.1, 1, 5, and 10 mM) on SDZ degradation in four systems: UV_{222} alone, UV_{222}-PAA, UV_{222}-Br, and UV_{222}-PB. The detailed results are presented in **Lines 642–665, Fig. 6f** and **Supplementary Figs. 29e–j** of the revised manuscript. Overall, NO_3^- exhibited a concentration-dependent pattern of low-concentration promotion and high-concentration inhibition in the UV_{222}, UV_{222}-Br, and UV_{222}-PB systems. In contrast, in the UV_{222}-PAA system, NO_3^- consistently inhibited SDZ degradation, which is consistent with the results of the reference you provided. This difference is attributed to the fact that •OH generated from NO_3^- photolysis reacts with Br^- to produce more selective and persistent bromine radicals. Once again, we thank you for your important suggestion, which has significantly enhanced the rigor and completeness of our manuscript

The related descriptions and images are presented below for your convenience:

Lines 642–665: "On the other hand, NO_3^- exhibits strong absorption at 222 nm (ε = 2747 $M^{-1}\cdot cm^{-1}$), and its photolysis generates •OH and reactive nitrogen species (RNS), which significantly affect the performance of UV_{222}-based processes[66]. To

comprehensively evaluate the role of NO_3^-, this study investigated the degradation of SDZ under different UV_{222}-based processes in the presence of NO_3^- (0.1–10 mM) (Fig. 6f and Supplementary Figs. 29e–j). Specifically, at low concentrations, RNS generated by NO_3^- photolysis under UV_{222} can attack the sulfonamide group and the aromatic N atoms of the heterocyclic ring of SDZ, resulting in a slight promoting effect. However, as the NO_3^- concentration increases, its light-shielding effect gradually reduces the degradation rate of the pollutant[67]. Notably, in the UV_{222}-PAA system, the addition of NO_3^- exhibited a significant inhibitory effect, which is consistent with previous reports[20]. This phenomenon is attributed to the strong photon competition by NO_3^-, which suppresses the activation of PAA and H_2O_2 in the solution, thereby preventing effective SDZ degradation[68]. In contrast, the k_{obs} value for the UV_{222}-Br process increased by 3.58 to 5.09 times after the addition of NO_3^-. This is because •OH generated from NO_3^- photolysis reacts rapidly with Br^- in solution to form $•Br_2^-$, which exhibits high reactivity toward SDZ ($k \approx 17.2 \times 10^7$ $M^{-1}·s^{-1}$)[28]. Based on these observations, in the UV_{222}-PB process, the enhancement effect of NO_3^- is optimal at a concentration of 1 mM, where the reaction rate constant is 1.40 times that of the system without NO_3^-. However, when the NO_3^- concentration increases further, the removal rate of SDZ decreases. This is because low concentrations of NO_3^- promote pollutant degradation by accelerating the generation of reactive bromine species, whereas the light-shielding effect at high NO_3^- concentrations limits the formation of radicals in the system."

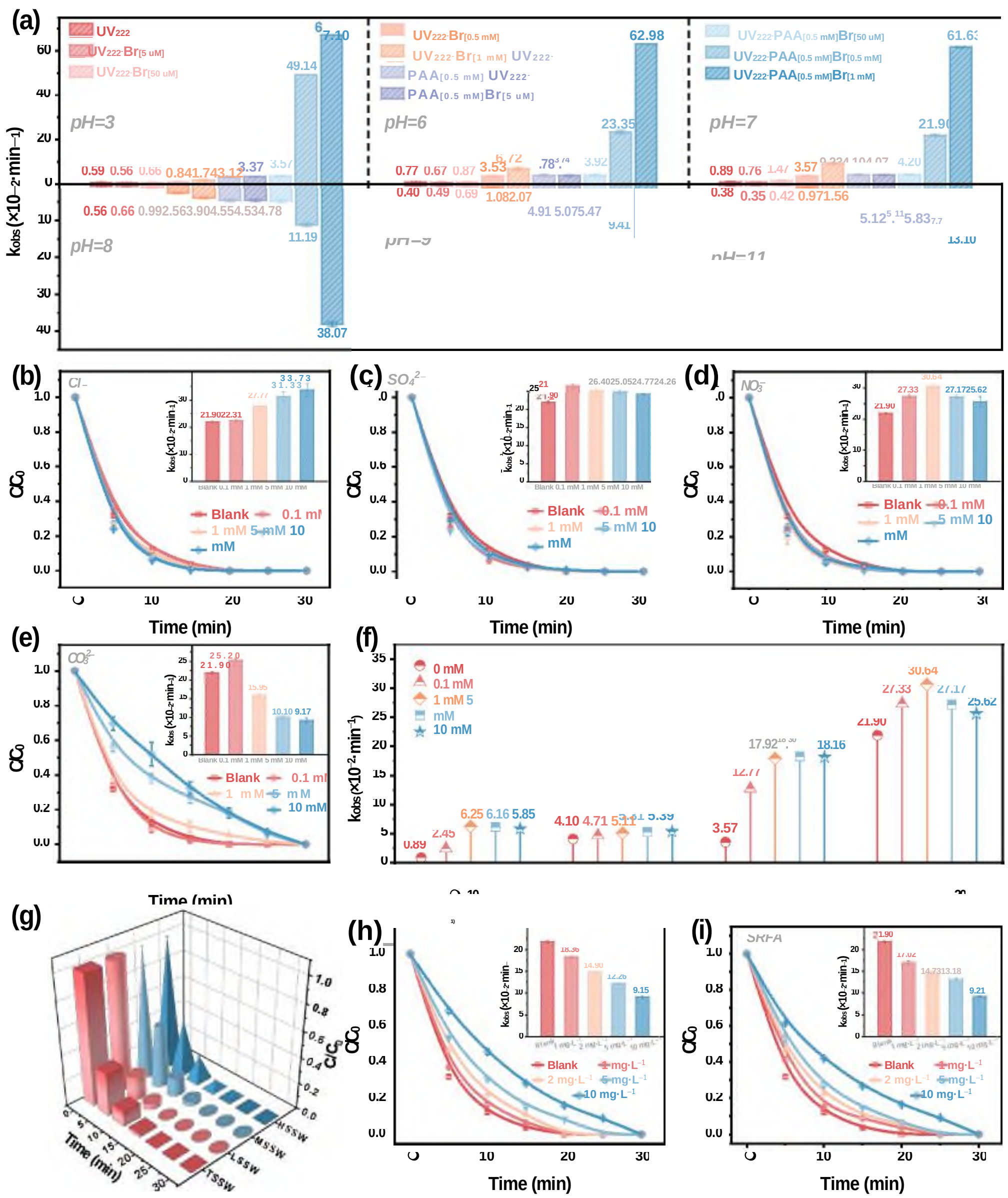

Fig. 6 | The Regulatory Effects and Mechanisms of Water Quality Parameters on SDZ Degradation. (a) k_{obs} values of SDZ in different processes at multiple pH levels. Degradation curves and k_{obs} values of SDZ degraded by UV_{222}-PB in the presence of (b) Cl^-, (c) SO_4^{2-}, (d) NO_3^-, (e) CO_3^{2-}. (f) The effect of coexisting NO_3^- on the degradation of SDZ under UV_{222} using different processes. (g) The performance of UV_{222}-PB in simulated water bodies with different salinities. Degradation curves and k_{obs} values of SDZ degraded by UV_{222}-PB in the presence of (h) SRHA and (i) SRFA.

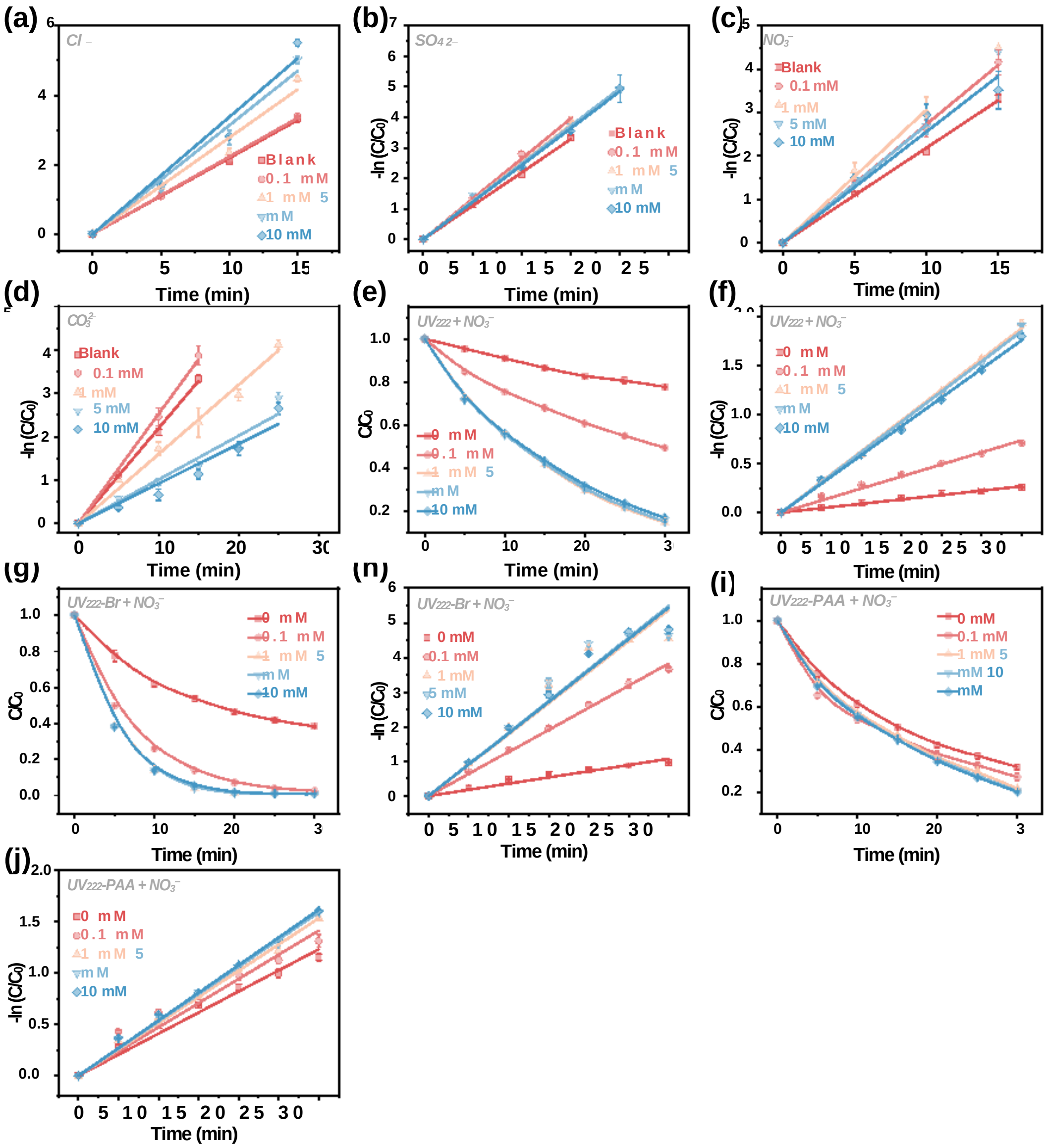

Supplementary Fig. 29 | Pseudo-first-order kinetic fitting curves of SDZ under different concentrations of (a) Cl^-, (b) SO_4^{2-}, (c) NO_3^-, and (d) CO_3^{2-} in the UV_{222}-PB system. Degradation curves and pseudo-first-order kinetic fitting curves of SDZ under different NO_3^- concentrations in the (e–f) UV_{222}, (g–h) UV_{222}-Br, and (i–j) UV_{222}-PAA systems. Conditions: $[SDZ]_0 = 20$ μM, $[PAA]_0 = [Br]_0 = 0.5$ mM, pH = 7.0.

Comment 7: What are the residual concentrations of PAA and H_2O_2 after the reaction? Can the remaining H_2O_2 continuously reduce HBrO back to Br^-?

Response: Thank you very much for your thorough review of our manuscript.

Your question regarding the residual concentrations of PAA and H_2O_2 after the reaction is of great value for elucidating the catalytic cycling mechanism of bromide ions. In response to your suggestion, we experimentally measured and modeled the concentration changes of PAA and H_2O_2 in the UV_{222}-PB process. The results showed that after 30 min of reaction, the residual concentrations of PAA and H_2O_2 were 0.2741 μM and 0.1262 μM, respectively, which are in good agreement with the predictions of our kinetic model. The rate constant for the second-order reaction between H_2O_2 and HOBr was 1.50×10^4 $M^{-1}\cdot s^{-1}$, and the concentration of residual H_2O_2 was significantly higher than the steady-state concentration of HOBr. This indicates that the remaining H_2O_2 in the system is sufficient to continuously reduce HOBr and regenerate Br^-, thereby providing strong support for the sustained operation of the bromine catalytic cycle. Furthermore, we also monitored the change of Br^- concentration at different time points in the UV_{222}-PB process. The results showed that the Br^- concentration after 30 min remained almost unchanged compared to the initial concentration, which indirectly confirms that H_2O_2 can continuously reduce HOBr back to Br^-, thus accomplishing the Br^- catalytic cycle. The relevant results have been added to the revised manuscript (**Lines 307–311, 415–425** and **Supplementary Figs. 14, 17f**). Once again, we would appreciate your valuable comments, which are crucial for improving the description of the bromine cycling mechanism.

The related descriptions and images are presented below for your convenience:

Lines 307–311: "Interestingly, ion chromatography measurements revealed the concentration changes of Br^- at different time points. The results indicated that the initial concentration of Br^- was nearly identical to its final concentration, showing no significant fluctuations throughout the reaction process and remaining at a relatively stable level (Supplementary Fig. 14)."

Lines 415–425: "In addition, considering the crucial role of H_2O_2 in the Br^- cycling and regeneration process, the study tracked the concentration changes of PAA and H_2O_2 during the reaction using both experimental measurements and model predictions (Supplementary Fig. 17f). Obviously, the experimental values and model

predictions agreed well over the entire time scale. At the end of the reaction, the consumption rates of PAA and H_2O_2 were approximately 45.19% and 36.90%, respectively. Given that the second-order reaction rate constant between H_2O_2 and HOBr is 1.50×10^4 $M^{-1} \cdot s^{-1}$, and the residual concentration of H_2O_2 (0.1262 μM) is much higher than the steady-state concentration of HOBr, it is demonstrated that H_2O_2 in the system is sufficient to continuously reduce HOBr back to Br^-, thereby ensuring the normal operation of the bromine catalytic cycle."

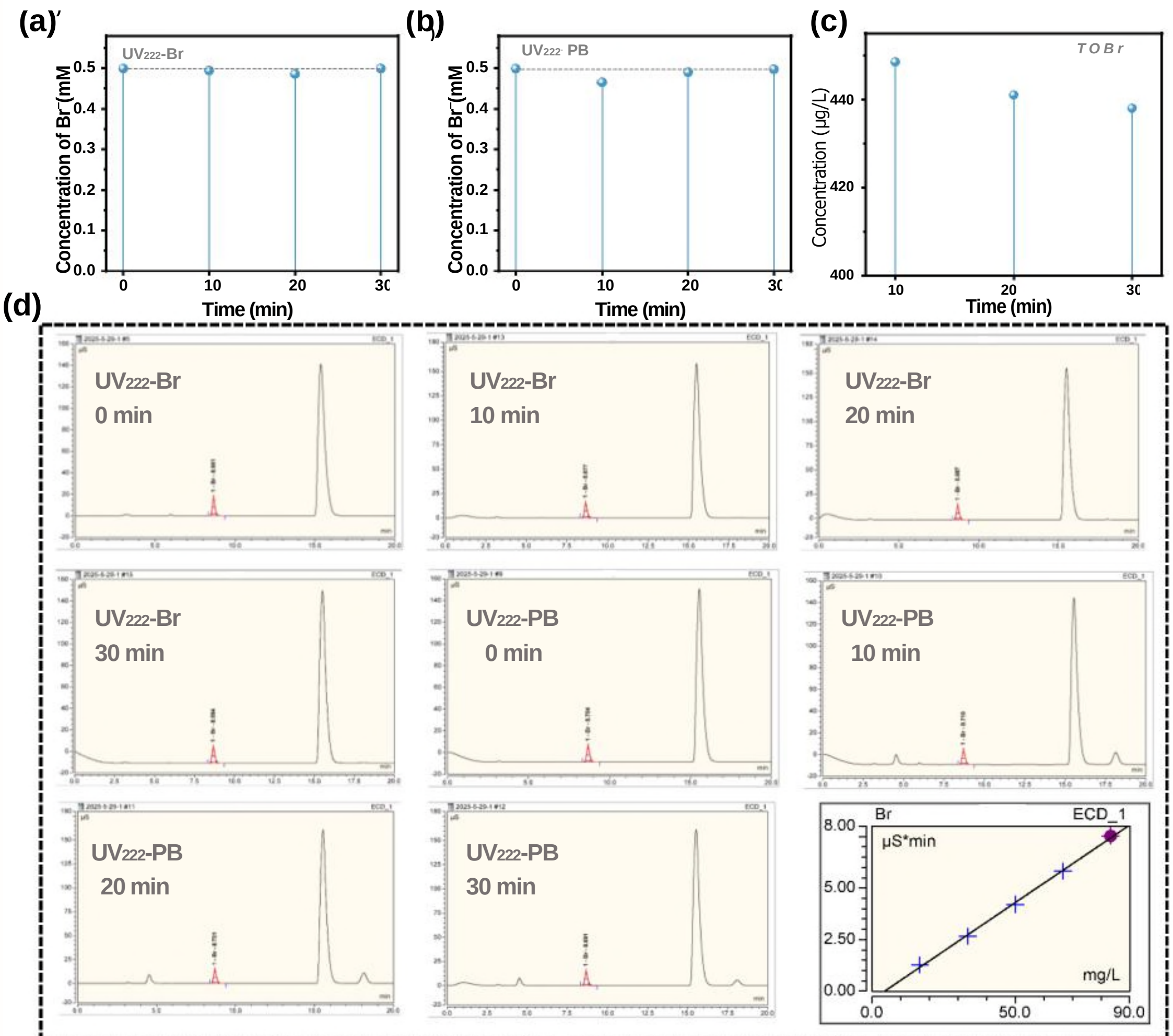

Supplementary Fig. 14 | (a) Concentration changes of Br^- in the UV_{222}-Br and (b) UV_{222}-PB systems. (c) Concentrations of total organic bromine (TOBr) generated during the UV_{222}-PB process at different time points. (d) Ion chromatograms of Br^- in the UV_{222}-Br and UV_{222}-PB systems at different time points, and standard curve of Br^-

under IC measurement.

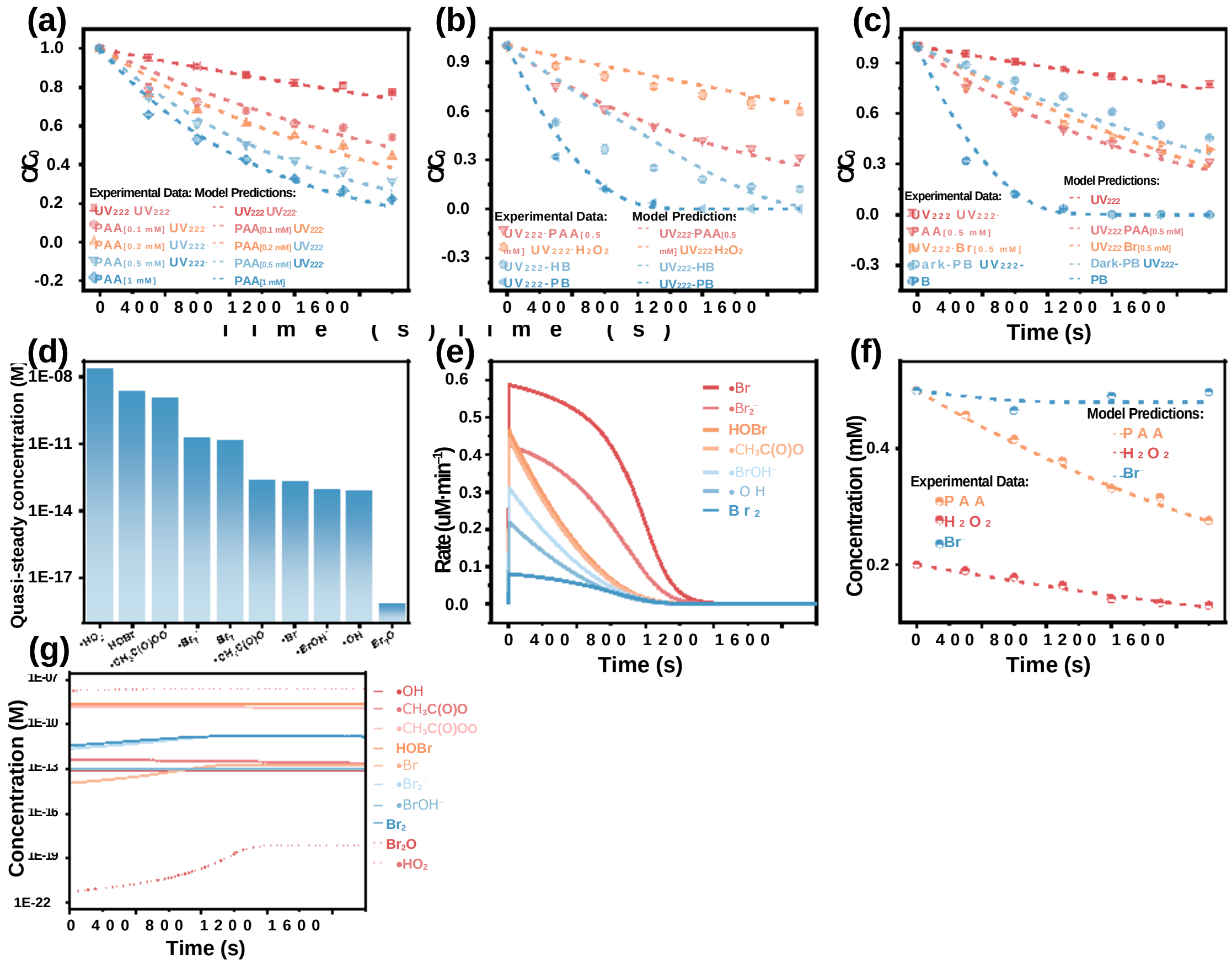

Supplementary Fig. 17 | (a–c) Degradation model curves of the UV222-PB process (symbols represent experimental data, dashed lines represent model predictions). (d) Final steady-state concentrations of different reactive species, (e) reaction rate curves, and (g) species concentration curves in the model. (f) Concentration variations of PAA, H_2O_2, and Br^-. symbols represent experimental data, dashed lines represent model predictions.

Comment 8: In Fig. 7, the SDZ removal rate in Yellow Sea water under UV222–PB is much higher than that in deionized water. It is worth clarifying whether the dominant reactive species in Yellow Sea water is Cl^--derived or Br^--derived. The authors should include additional experiments on SDZ degradation in the UV222–PAA–Cl^- (0.5 M) system.

Response: Thank you very much for raising this key question, which helps to

further clarify the attribution of the dominant reactive species in real seawater systems. In response to your suggestion, we conducted a series of experiments under high chloride concentration (0.5 M Cl^-) and developed a kinetic model under the same high-chloride background to elucidate the dominant reactive species under such conditions. The results showed that in the UV_{222}-Cl system containing only 0.5 M Cl^-, the degradation rate of SDZ was only 46.78% of that in the UV_{222}-Br system. After the addition of PAA, the degradation rate in the UV_{222}-PAA-Cl (UV_{222}-PC) system was still much lower than that in the UV_{222}-PB process. In contrast, in the chlorine-bromine coexisting UV_{222}-PAA-Br-Cl (UV_{222}-PBC) system, the degradation rate of SDZ increased dramatically, being 8.45 and 1.76 times higher than those in the UV_{222}-PC and UV_{222}-PB systems, respectively. These experimental results preliminarily demonstrate that chlorine-derived species alone cannot achieve efficient degradation, and that the presence of Br^- is key to performance enhancement.

Furthermore, terminal contribution analysis of the UV_{222}-PBC kinetic model showed that reactive bromine species (RBS) contributed 66.02% to SDZ degradation, far exceeding the contribution of reactive chlorine species (RCS) at 1.55%. Among these, $Br_2{\bullet}^-$ emerged as the dominant reactive species, contributing as much as 48.08%. Moreover, the mixed halogen species $\bullet BrCl^-$ became the second major contributing pathway, providing effective evidence for halogen cross-reactions. In summary, through the combined validation of experiments and kinetic modeling, it is clarified that in Yellow Sea water, Br^--derived species and Cl^--derived species together dominate SDZ degradation. Detailed experimental results and model descriptions are presented in **Lines 730–800** and **Figs. 7c–f**. We thank you again for your rigorous review, which has deepened our understanding of the dominant mechanisms in high-salinity water systems.

The related descriptions and images are presented below for your convenience:

Lines 730–800: "However, while high concentrations of Cl^- promote degradation, they may also compete with Br^- for reactive species, thereby interfering with the formation of bromine species[71]. To better clarify the dominant reactive species

responsible for pollutant degradation in the UV_{222}-PB process in real seawater, we conducted a series of supplementary experiments under high Cl^- concentration (Figs. 7c–d and Supplementary Fig. 31b). Specifically, in the UV_{222}-Cl system containing only 0.5 M Cl^-, the degradation of SDZ was 36.94%, and its k_{obs} value was only 0.47 times that of the UV_{222}-Br system. Upon the addition of PAA, although the k_{obs} value of SDZ degradation in the UV_{222}-PAA-Cl (UV_{222}-PC) system increased to 0.0457 min^{-1}, it remained much lower than that in the UV_{222}-PB process. Moreover, compared with Dark-PB, the degradation rate of SDZ in Dark-PC decreased by approximately 60.32% (Supplementary Figs. 31c–e), a difference that may be attributed to the fact that the rate constant for the reaction of PAA with Cl^- to form HOCl is about four orders of magnitude lower than that for the reaction of PAA with Br^- to form HOBr[34]. Notably, in the UV_{222}-PAA-Br-Cl (UV_{222}-PBC) system containing 0.5 M Cl^-, SDZ was already degraded by 98.12% within 15 min, and its k_{obs} value was 1.76 and 8.45 times higher than those in the UV_{222}-PB and UV_{222}-PC systems, respectively. In summary, the above experimental results demonstrate that chlorine-derived species alone cannot achieve efficient pollutant degradation, and the presence of Br^- is an indispensable part of performance enhancement.

Furthermore, to further elucidate the attribution and interaction of dominant reactive species in the UV_{222}-PB process under high chloride concentration, we introduced 0.5 M Cl^- into the original kinetic model and constructed the UV_{222}-PBC model to better simulate seawater and seawater-affected high-salinity water bodies. Remarkably, the terminal contribution analysis of the UV_{222}-PBC model revealed that RBS (66.02%) contributed far more to SDZ degradation than RCS (1.55%), with $\bullet Br_2^-$ becoming the dominant reactive species, contributing as much as 48.08% (Figs. 7e–f and Supplementary Fig. 32a–e). This phenomenon is attributed to the rapid reaction of $\bullet$Cl and $\bullet Cl_2^-$ with Br^- in the solution to form $\bullet BrCl^-$, resulting in low steady-state concentrations of chlorine radicals. Although the steady-state concentration of $\bullet Cl_2^-$ (3.83×10^{-14} M) is of the same order of magnitude as that of $\bullet$Br (3.64×10^{-14} M), its reaction rate with SDZ is approximately three orders of magnitude lower. Moreover,

the steady-state concentration of •Cl is even lower, making the direct contribution of chlorine radicals to SDZ degradation very weak. Furthermore, although HOCl is present in high abundance, its reactivity toward SDZ is relatively low. Additionally, among the various transformation pathways involving $•BrCl^{-}$, its further reaction with Br^{-} to form $•Br_2^{-}$ ($k = 8\times10^9$ $M^{-1}\cdot s^{-1}$) exhibits the highest rate, leading to an approximately 181% increase in the steady-state concentration of $•Br_2^{-}$ in the UV_{222}-PBC model compared to the UV_{222}-PB model. Moreover, the relatively low H+ concentration in the neutral environment limits the conversion of $•BrOH^{-}$ to •Br, and the high concentration of Cl^{-} in the solution also consumes •Br, resulting in a steady-state concentration of •Br that was approximately 5.6 times lower than that observed with UV_{222}-PB. Consequently, multiple factors together cause $•Br_2^{-}$ to far exceed •Br in its contribution to SDZ degradation, becoming the dominant reactive bromine species in the UV_{222}-PBC system.

On the other hand, the rapid interconversion among halogen reactive species drives the dynamic evolution of the compositional structure of reactive species in the system. Accordingly, the model further quantified the steady-state concentrations and reaction contributions of mixed bromine-chlorine species (e.g., $•BrCl^{-}$, BrCl, BrOCl). Due to the relatively low reaction rate between HOBr and HOCl, the formation concentration of BrOCl is extremely low, rendering its contribution to SDZ degradation negligible. Compared with the UV_{222}-PB model, the steady-state concentrations of •Br and $•BrOH^{-}$ in the UV_{222}-PBC model decrease significantly, a phenomenon attributed to the rapid reaction of these species with high-concentration Cl^{-}, leading to the formation of mixed halogen species such as $•BrCl^{-}$. Furthermore, although the steady-state concentrations of BrCl and $•BrCl^{-}$ are comparable, their reaction rates with SDZ differ by approximately five orders of magnitude, resulting in a marked difference in their contributions to SDZ degradation. Notably, the model predictions show that compared with UV_{222}-PB, PAA consumption decreases in the UV_{222}-PBC model, while H_2O_2 consumption increases (Supplementary Figs. 32f–g). This is because the steady-state concentration of •OH in the UV_{222}-PBC system decreases to 3.13×10^{-17} M,

which hinders the reaction of PAA with •OH, leading to an 89% decrease in the steady-state concentration of •$CH_3C(O)OO$. This is the main reason for the significant reduction in the contribution of •$CH_3C(O)OO$ in the UV_{222}-PBC model. Meanwhile, H_2O_2 in the UV_{222}-PBC system is required not only to reduce HOBr but also to react with HOCl, resulting in increased H_2O_2 consumption. Nevertheless, the Br^- concentration remains largely unchanged during the reaction, confirming the robust operation of the bromine cycle under high-chloride conditions. In summary, although Cl^- concentration is overwhelmingly dominant, Br^- still plays an indispensable role in the UV_{222}-PBC system, allowing bromine and chlorine species to jointly dominate the oxidative degradation of pollutants."

(Redaction)

Comment 9: Is the enhanced SDZ removal in Yellow Sea water attributed to the formation of chlorine radicals? Could bromine radicals be scavenged by Cl^-? Moreover, would the shift in dominant halogen radical species also promote the removal of other pollutants beyond SDZ?

Response: We sincerely thank your rigorous review of the manuscript and greatly value your key insights regarding the impact of halogen radical species transformation on the degradation process. Regarding the mechanism underlying the enhanced removal rate of SDZ in Yellow Sea seawater, we draw the following conclusions based on experimental and kinetic modeling results: (1) The efficient removal of SDZ in Yellow Sea seawater is not solely attributable to chlorine radicals. Although the steady-state concentration of $•Cl_2^-$ is on the same order of magnitude as that of •Br under high Cl^- concentrations, its reaction rate with SDZ is approximately three orders of magnitude lower. Moreover, given the even lower steady-state concentration of •Cl, the direct contribution of chlorine radicals to SDZ degradation is minimal. The primary reactive species in the system are $•Br_2^-$ and $•BrCl^-$, indicating that the enhanced degradation results from the synergistic effects of chlorine and bromine species. (2) Bromine radicals are not completely scavenged by Cl^-. Model predictions show that upon the introduction of 0.5 M Cl^-, the steady-state concentration of •Br decreases from 2.04×10^{-13} M to 3.64×10^{-14} M. Although high Cl^- concentrations quench a certain amount of •Br, the rapid interconversion reactions among halogen radicals in the system enable the continuous regeneration of •Br, thereby maintaining its steady-state concentration.

Furthermore, by further evaluating the degradation behavior of typical aquatic organic micropollutants with significantly different chemical structures and functional groups in the UV_{222}-PBC system and Yellow Sea seawater, we confirmed that the shift in dominant halogen radical species also promotes the removal of other OMPs. The

experimental results show that, owing to the increase in reactive species such as $•Br_2^-$ and HOCl, as well as the synergistic oxidation resulting from rapid interconversion among halogen reactive species, the degradation efficiencies of the four OMPs in the UV_{222}-PBC system were all enhanced compared to those in the UV_{222}-PB system. However, in Yellow Sea seawater, the degradation efficiencies of SMZ and TC, which have high reaction rates with $•Br_2^-$, decreased, whereas those of CIP and CBZ increased. This is because the presence of CO_3^{2-} and HCO_3^- in Yellow Sea seawater scavenges

•OH in the solution, thereby hindering the generation of ·Br and $•Br_2^-$. Although the degradation efficiencies of different OMPs in real Yellow Sea seawater vary, the overall removal performance remains stable and efficient, confirming the broad-spectrum applicability of the shift in halogen radical species. Relevant descriptions have been added to **Lines 750–826** and **Fig.7e–g** of the revised manuscript. Once again, we would appreciate your insightful comments, which are very important for enhancing the generality of the conclusions of this study.

The related descriptions and images are presented below for your convenience:

Lines 750–826: "Furthermore, to further elucidate the attribution and interaction of dominant reactive species in the UV_{222}-PB process under high chloride concentration, we introduced 0.5 M Cl^- into the original kinetic model and constructed the UV_{222}-PBC model to better simulate seawater and seawater-affected high-salinity water bodies. Remarkably, the terminal contribution analysis of the UV_{222}-PBC model revealed that RBS (66.02%) contributed far more to SDZ degradation than RCS (1.55%), with $•Br_2^-$ becoming the dominant reactive species, contributing as much as 48.08% (Figs. 7e–f and Supplementary Fig. 32a–e). This phenomenon is attributed to the rapid reaction of

•Cl and $•Cl_2^-$ with Br^- in the solution to form $•BrCl^-$, resulting in low steady-state concentrations of chlorine radicals. Although the steady-state concentration of $•Cl_2^-$ (3.83×10^{-14} M) is of the same order of magnitude as that of •Br (3.64×10^{-14} M), its reaction rate with SDZ is approximately three orders of magnitude lower. Moreover, the steady-state concentration of •Cl is even lower, making the direct contribution of chlorine radicals to SDZ degradation very weak. Furthermore, although HOCl is

present in high abundance, its reactivity toward SDZ is relatively low. Additionally, among the various transformation pathways involving $\bullet BrCl^-$, its further reaction with Br^- to form $\bullet Br_2^-$ (k = 8×10^9 $M^{-1}\cdot s^{-1}$) exhibits the highest rate, leading to an approximately 181% increase in the steady-state concentration of $\bullet Br_2^-$ in the UV_{222}-PBC model compared to the UV_{222}-PB model. Moreover, the relatively low H+ concentration in the neutral environment limits the conversion of $\bullet BrOH^-$ to •Br, and the high concentration of Cl^- in the solution also consumes •Br, resulting in a steady-state concentration of •Br that was approximately 5.6 times lower than that observed with UV_{222}-PB. Consequently, multiple factors together cause $\bullet Br_2^-$ to far exceed •Br in its contribution to SDZ degradation, becoming the dominant reactive bromine species in the UV_{222}-PBC system.

On the other hand, the rapid interconversion among halogen reactive species drives the dynamic evolution of the compositional structure of reactive species in the system. Accordingly, the model further quantified the steady-state concentrations and reaction contributions of mixed bromine-chlorine species (e.g., $\bullet BrCl^-$, BrCl, BrOCl). Due to the relatively low reaction rate between HOBr and HOCl, the formation concentration of BrOCl is extremely low, rendering its contribution to SDZ degradation negligible. Compared with the UV_{222}-PB model, the steady-state concentrations of •Br and $\bullet BrOH^-$ in the UV_{222}-PBC model decrease significantly, a phenomenon attributed to the rapid reaction of these species with high-concentration Cl^-, leading to the formation of mixed halogen species such as $\bullet BrCl^-$. Furthermore, although the steady-state concentrations of BrCl and $\bullet BrCl^-$ are comparable, their reaction rates with SDZ differ by approximately five orders of magnitude, resulting in a marked difference in their contributions to SDZ degradation. Notably, the model predictions show that compared with UV_{222}-PB, PAA consumption decreases in the UV_{222}-PBC model, while H_2O_2 consumption increases (Supplementary Figs. 32f–g). This is because the steady-state concentration of •OH in the UV_{222}-PBC system decreases to 3.13×10^{-17} M, which hinders the reaction of PAA with •OH, leading to an 89% decrease in the steady-state concentration of $\bullet CH_3C(O)OO$. This is the main reason for the significant

reduction in the contribution of $\bullet CH_3C(O)OO$ in the UV_{222}-PBC model. Meanwhile, H_2O_2 in the UV_{222}-PBC system is required not only to reduce HOBr but also to react with HOCl, resulting in increased H_2O_2 consumption. Nevertheless, the Br^- concentration remains largely unchanged during the reaction, confirming the robust operation of the bromine cycle under high-chloride conditions. In summary, although Cl^- concentration is overwhelmingly dominant, Br^- still plays an indispensable role in the UV_{222}-PBC system, allowing bromine and chlorine species to jointly dominate the oxidative degradation of pollutants.

Real seawater contains various complex components. To further evaluate the universality of the UV_{222}-PB process for pollutant degradation in real seawater, this study selected typical aquatic OMPs such as SMZ, CIP, CBZ, and TC, and systematically investigated their degradation behavior in YS water matrix (Fig. 7g and Supplementary Fig. 33). Notably, compared with the UV_{222}-PB system, the addition of high-concentration chloride ions increased the degradation rates of all pollutants. The k_{obs} values of SMZ, CIP, CBZ, and TC in the UV_{222}-PBC system increased to 0.3203 min^{-1}, 0.1169 min^{-1}, 0.0268 min^{-1}, and 1.2872 min^{-1}, respectively. This enhancement is attributed to the increase in reactive species such as $\bullet Br_2^-$ and HOCl, as well as the synergistic oxidation arising from the rapid interconversion among halogen reactive species. However, in real YS water, the UV_{222}-PB process exhibited differentiated degradation trends for different pollutants. Specifically, SMZ (3.18×10^8 $M^{-1}\cdot s^{-1}$) and TC (6.26×10^8 $M^{-1}\cdot s^{-1}$) show high reactivity toward $\bullet Br_2^-$[28]. In real Ys water, the coexisting CO_3^{2-} and HCO_3^- effectively quench •OH in the system, thereby inhibiting the generation of •Br and $\bullet Br_2^-$. Since TC exhibits a higher reaction rate with $\bullet Br_2^-$ than SMZ, its degradation is more significantly inhibited. In contrast, CIP and CBZ show relatively lower reactivity toward bromine species and display a promoting effect in real seawater[28,72]. This is because SO_4^{2-} present in real water matrices can be converted into secondary radicals such as $\bullet SO_4^-$, which rapidly attack and degrade the pollutants. Among them, CBZ shows a more pronounced degradation promotion, which may be attributed to the high reaction rate of $\bullet SO_4^-$ with CBZ (1.92×10^9 $M^{-1}\cdot s^{-1}$) and its

effective disruption of the central heterocyclic olefinic double bond in CBZ[73]. Although the degradation efficiency of UV_{222}-PB for different OMPs in real seawater varies, the overall removal performance remains stable and efficient. This confirms that the UV_{222}-PB process possesses broad-spectrum and high-efficiency removal capabilities for OMPs, demonstrating good application potential in high-salinity water treatment."

(Redaction)

Reviewer #2

General comments: *This work developed a novel far-ultraviolet-based water treatment process, using various analytical techniques to comprehensively reveal the hidden synergistic mechanism and ecotoxicological effects between peracetic acid (PAA) and bromide under far-UV irradiation. Compared with conventional* UV_{254}, UV_{222} *more effectively activates PAA and bromide, enabling ultrafast pollutant degradation at relatively safe UV doses. The core mechanism involves the efficient generation of highly reactive organic radicals and HOBr in the* UV_{222}*-PAA/*Br^{-} *system, which drives the oxidative degradation. This system exhibits excellent selective oxidation toward electron-rich pollutants and maintains strong performance across diverse real water matrices. Multiple toxicity assessments confirm that the resulting disinfection byproducts remain within safe limits, highlighting advantages including low energy consumption, high efficiency, and practical application potential. Overall, the work addresses a novel and interesting topic with clear logic, detailed data, and comprehensive analysis, offering insightful strategies for key challenges in water treatment. Well-designed figures and tables also enhance readability. It is recommended that this manuscript can be accepted in Nature Communications after addressing these minor issues.*

Response: We sincerely appreciate your recognition and encouragement of our manuscript, as well as for your valuable and constructive comments. Your positive affirmation of the novelty, logic, data comprehensiveness, figure and table design of our work has greatly inspired us. Meanwhile, your thoughtful and pertinent suggestions have provided important guidance for our revision. We have carefully revised and thoroughly improved the manuscript in response to each of your comments, striving to make the arguments more rigorous and the content more complete. Specific modifications are presented in the point-by-point response. Once again, we appreciate your comprehensive review and professional insights, which have been highly beneficial to us. We hope that our response and revisions will meet your satisfaction.

Comment 1: The manuscript appears to have inconsistent formatting in multiple sections, including text and equation editing. Please carefully review the entire text and make necessary corrections.

Response: Thank you very much for your rigorous review of the manuscript. Your meticulousness and strict requirements have greatly instructed us. We have followed your comments and systematically reviewed and revised the entire manuscript item by item. All modifications have been marked in red in the revised manuscript.

Comment 2: The findings reveal an interesting phenomenon, bromide exhibits a catalytic effect in the UV_{222}-PAA system. However, is the bromide concentration selected based on environmental levels? Please clarify the experimental basis for this concentration and the actual water scenario it simulates.

Response: Thank you very much for raising this key question. The selection of the 0.5 mM bromide concentration in this study is primarily based on the environmental levels of seawater and other high-salinity water bodies. The details are as follows: (1) According to previous literature, the concentration of Br^- in seawater, marine aquaculture wastewater, and areas affected by seawater intrusion and backflow is relatively high, with typical concentrations reaching 0.5 mM (approximately 39.95 mg/L). Therefore, we selected 0.5 mM Br^- for our experiments to simulate the typical Br^- concentration in seawater and seawater-impacted high-salinity water bodies (e.g., desalination plant influent, coastal groundwater, and intruded freshwater systems). (2) The study investigated the effects of different Br^- concentrations (5 μM, 50 μM, 0.5 mM, and 1 mM) on the efficiency of micropollutant degradation in the UV_{222}-PAA system. The results showed that when the Br^- concentration was below 50 μM, the time-based pseudo-first-order rate constant (k_{obs}) for SDZ degradation showed almost no improvement. However, when the Br^- concentration reached 0.5 mM or higher, the k_{obs} value for SDZ degradation increased from 0.0410 min^{-1} to 0.2190-0.6163 min^{-1}, representing an enhancement of 5.34-15.03-fold (Fig. 1c). This finding indicates that 0.5 mM is an important threshold concentration for influencing micropollutant

degradation in the UV_{222}-PAA system. (3) Furthermore, in **Lines 722–729** of the revised manuscript, we conducted real-water experiments using Yellow Sea (YS) and aquaculture seawater wastewater (ASW) from Guangxi to further validate the practical relevance of this concentration choice. The results showed that in YS, the degradation efficiency of the UV_{222}-PB process was significantly enhanced, with a k_{obs} value of 0.4912 min^{-1}, representing a 2.24-fold increase. On the other hand, although the high organic matter content in the marine aquaculture wastewater exerted certain light-shielding and competitive effects, leading to a reduction in the kobs value for SDZ degradation in the UV_{222}-PB process, the system still maintained a relatively high degradation efficiency (Figs. 7a–b). Taken together, these results demonstrate that at a bromide concentration of 0.5 mM, the UV_{222}-PAA process exhibits high micropollutant degradation efficiency and holds potential for practical application.

The related descriptions and images are presented below for your convenience:

Lines 104–107: "Seawater and water supply systems affected by seawater intrusion, as well as industrial wastewater, contain elevated levels of Br^{-}[26]. Among these, as a typical high-brine water body, seawater can reach Br^{-} concentrations of up to 0.5 mM."

Lines 139–144: "Similarly, in UV_{222}-Br system, the degradation efficiency of SDZ also increased from 19.74% to 92.15% (Fig. 1b). This is because under 222 nm light excitation, Br^{-} underwent photoactivation mediated by hydrated clusters to generate •Br. This radical could directly attack the aniline group in SDZ and subsequently reacted with Br^{-} to form highly reactive dibromine radical anion ($•Br_2^{-}$), which synergistically degraded SDZ[22]."

Lines 722–729: "However, in ASW (k_{obs} = 0.1507 min^{-1}) water sample, the degradation of SDZ was still prevented due to the light shielding and competition effect generated by high organic matter content, even at high Cl^{-} concentrations (2862 $mg·L^{-1}$). Specifically, the UV_{222}-PB system performed substantially better in YS water (k_{obs} = 0.4912 min^{-1}), attaining a 99.19% SDZ degradation rate in 10 minutes. This strong promotion effect was attributed to the high concentration of Cl^{-} (25367 $mg·L^{-1}$),

resulting in a considerable promotion effect, leading to the conversion of more active species."

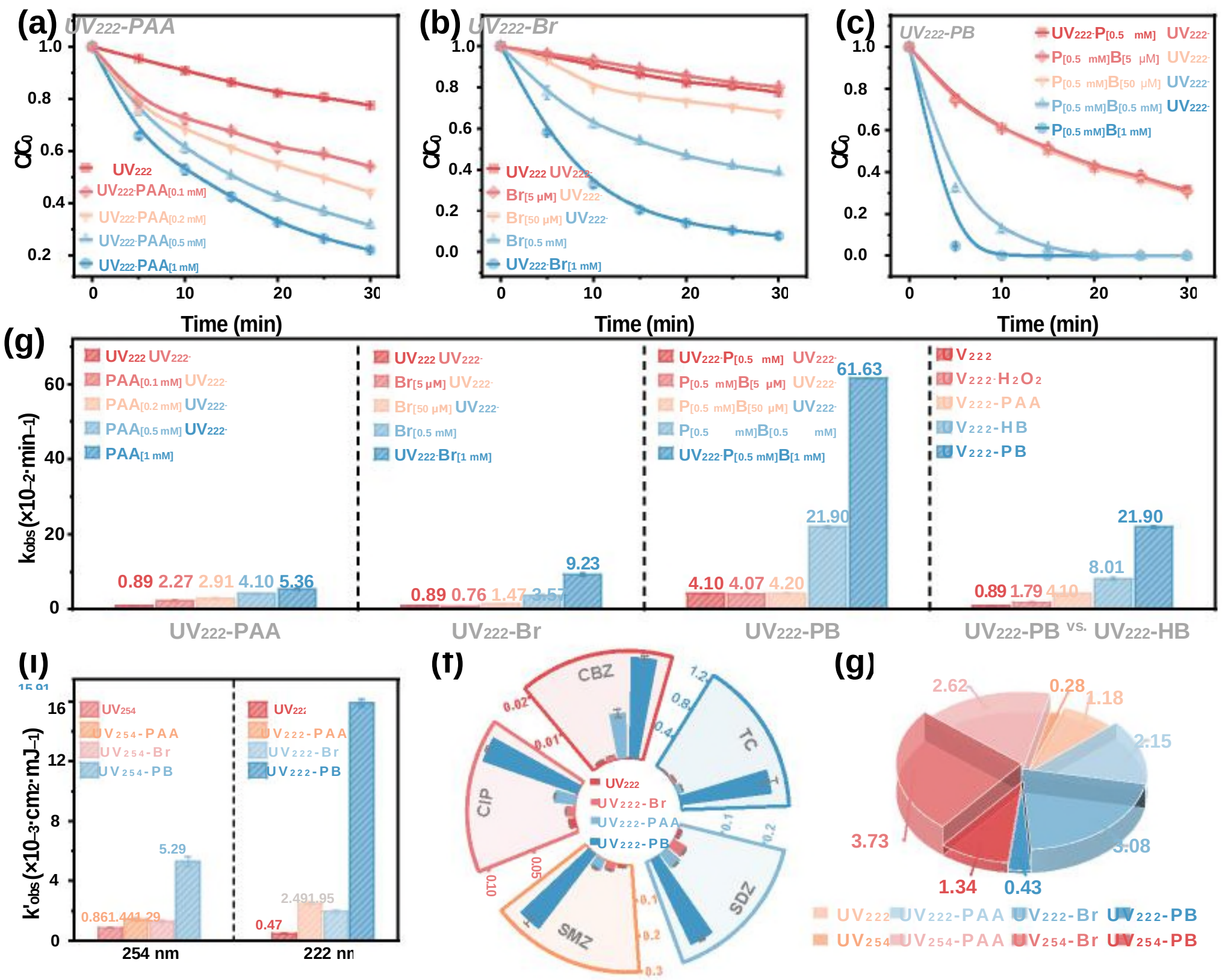

Fig. 1 | Efficient SDZ degradation and characteristic evaluation under UV-based processes. (a–c) SDZ degradation curves under various UV_{222} processes, and (d) k_{obs} values. (e) k'_{obs} values for various UV_{222} and UV_{254} processes. (f) k_{obs} values for the degradation of various OMPs under the UV_{222} process. (g) EE/O values during SDZ degradation using various processes. Conditions: $[SDZ]_0 = 20$ μM, pH = 7.0, $[H_2O_2]_0 = 200$ μM, $[PAA]_0 = 0.1$ mM-1 mM, $[Br]_0 = 5$ μM-1 mM (unless otherwise mentioned, $[PAA]_0 = [Br]_0 = 0.5$ mM).

(Redaction)

(Redaction)

Comment 3: Besides UV_{222} and UV_{254}, UV_{365} is also a commonly used UV light source. It is recommended to supplement relevant experiments to further highlight the advantages of UV_{222}.

Response: Thank your constructive suggestion, which is crucial for highlighting the advantages of UV_{222}. In response to your comment, we conducted additional experiments at 365 nm. The results showed that the fluence based degradation rate constant (k'_{obs}) of the UV_{365}-PB process is less than one tenth of that of the UV_{222}-PB process. These results clearly demonstrate the superior performance of UV_{222} in synergistically activating PAA and Br^{-}. The relevant explanation has been added to Supplementary Information Text S5 (**Lines 87–100**).

The related descriptions and images are presented below for your convenience:

Text S5. Evaluation of the performance of the UV-PAA/Br process under 365 nm UV wavelength.

Since SDZ and Br^{-} exhibit almost no light absorption at 365 nm, the degradation of SDZ in the UV_{365} direct photolysis system and the UV_{365}-Br system is negligible (Supplementary Fig. 5a–b). According to previous studies, although PAA cannot generate organic radicals under 365 nm irradiation, it can still produce a small amount of •OH, which explains the slight enhancement of SDZ degradation in the UV_{365}-PAA system[2]. Notably, the fluence-based degradation rate constant (k'_{obs}) of SDZ in the UV_{365}-PB system is 505 times and 2.77 times higher than that in the UV_{365}-Br and UV_{365}-PAA systems, respectively. This is because PAA and Br^{-} can react to form HOBr, thereby promoting SDZ degradation. However, compared with the UV_{365}-PB process (k'_{obs} = 0.00101 $cm^2 \cdot mJ^{-1}$), the degradation rate of the UV_{222}-PB process (k'_{obs} = 0.0159 $cm^2 \cdot mJ^{-1}$) is 15.74 times higher, which further fully demonstrates the unique advantage of UV_{222} in synergistically activating PAA and Br^{-}.

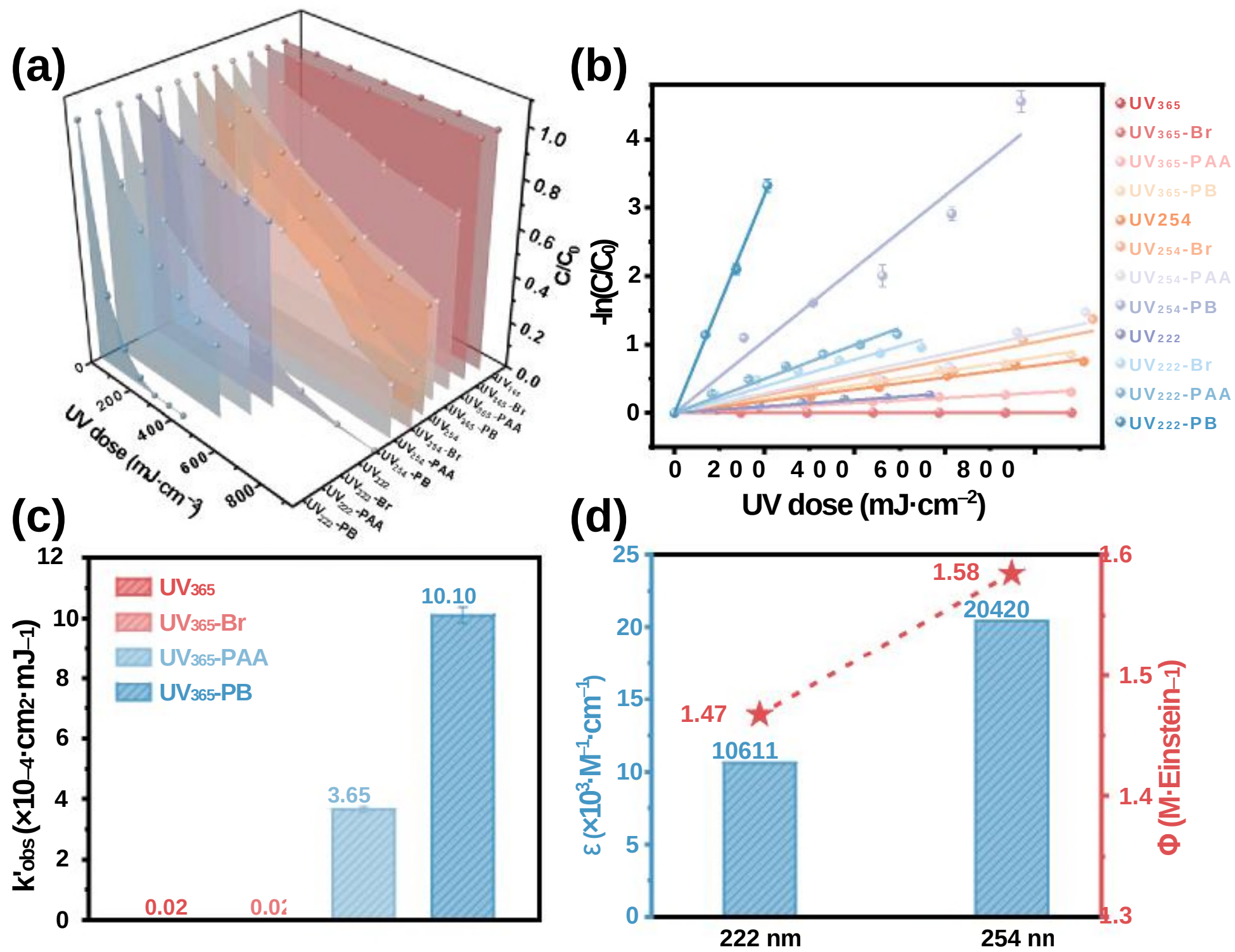

Supplementary Fig. 5 | (a) Degradation curves of SDZ under irradiation at different wavelengths and (b) pseudo-first-order kinetic fitting curves. (c) k_{obs} values for different processes under UV_{365}. (d) ε and Φ of SDZ at 222 and 254 nm. Conditions: $[SDZ]_0$=20 uM, pH=7.0, $[PAA]_0$=$[Br]_0$=0.5 mM.

Comment 4: Line 144: “section 3.2” is confusing. Please correctly indicate where the synergistic mechanism between PAA and bromide is discussed.

Response: We sincerely thank you for raising this important point, which helps improve the quality and rigor of our manuscript. In **Line 154**, to avoid ambiguity, we have revised the text to read “**see synergistic interaction mechanism for more information**” to more accurately direct readers to the relevant discussion of the synergistic mechanism between PAA and Br-. Thank you again for your careful review.

Comment 5: Lines 214-219: The hydroxyl radical probe experiment is incomplete. Please supplement the measurement of hydroxybenzoic acid.

Response: We sincerely appreciate your valuable comments on the completeness

and rigor of the manuscript. In accordance with your important suggestion, we have supplemented the images of hydroxybenzoic acid measurements in **Supplementary Figs. 9, 27** of the revised manuscript, and added the corresponding descriptions in **Lines 267–271, 595–600**. The results showed that almost no hydroxybenzoic acid was generated in the UV_{222} and UV_{222}-Br systems. Moreover, compared with the UV_{222}-PAA system, the concentration of hydroxybenzoic acid generated in the UV_{222}-PB process was lower. This phenomenon is attributed to the competitive consumption of •OH by Br^- and $•Br_2^-$ in the solution, which consequently suppressed the formation of hydroxybenzoic acid.

The related descriptions and images are presented below for your convenience:

Lines 267–271: "Notably, although the consumption of BA in the UV_{222}-PB system increased compared to the UV_{222}-PAA system, the formation of p-hydroxybenzoic acid (p-HBA) decreased. This is due to the competitive consumption of •OH by Br^- and $•Br_2^-$ in the solution (Equations 4 and 7), which consequently leads to a reduction in the formation of p-HBA (Supplementary Fig. 9)."

Lines 595–600: "On the other hand, in the UV_{222}-Br system, increasing the Br^- concentration from 0 to 1 mM gradually enhances the degradation efficiency of SDZ. Interestingly, the UV_{222}-Br process shows better degradation performance at pH 7. This phenomenon may be attributed to the reduced generation of •OH under acidic conditions (Supplementary Fig. 27), while alkaline conditions are unfavorable for the formation of HOBr[25]."

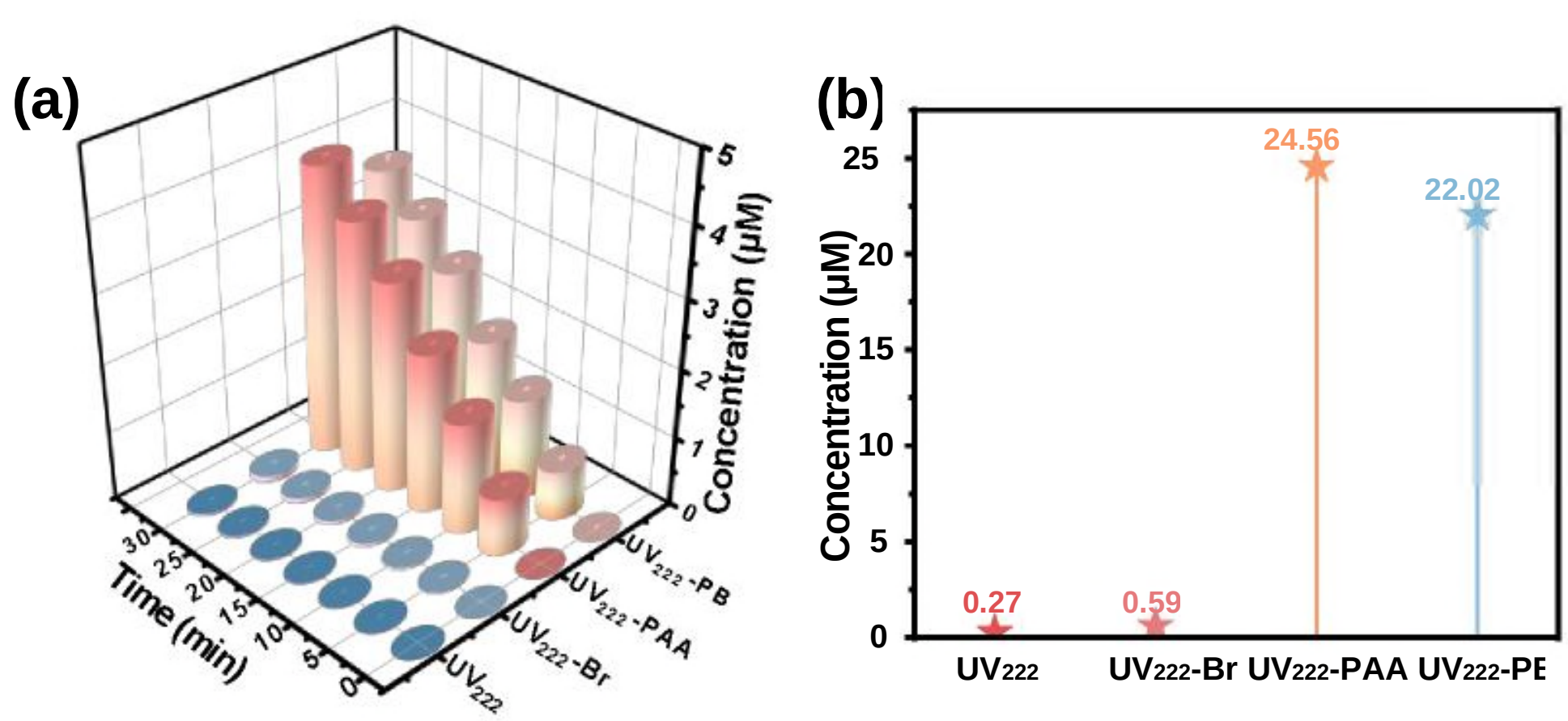

Supplementary Fig. 9 | Under UV_{222} irradiation in different processes (a) the generated concentration of p-HBA and (b) the cumulative concentration of •OH. Conditions: $[SDZ]_0$=20 uM, pH=7.0, $[PAA]_0$=$[Br]_0$=0.5 mM.

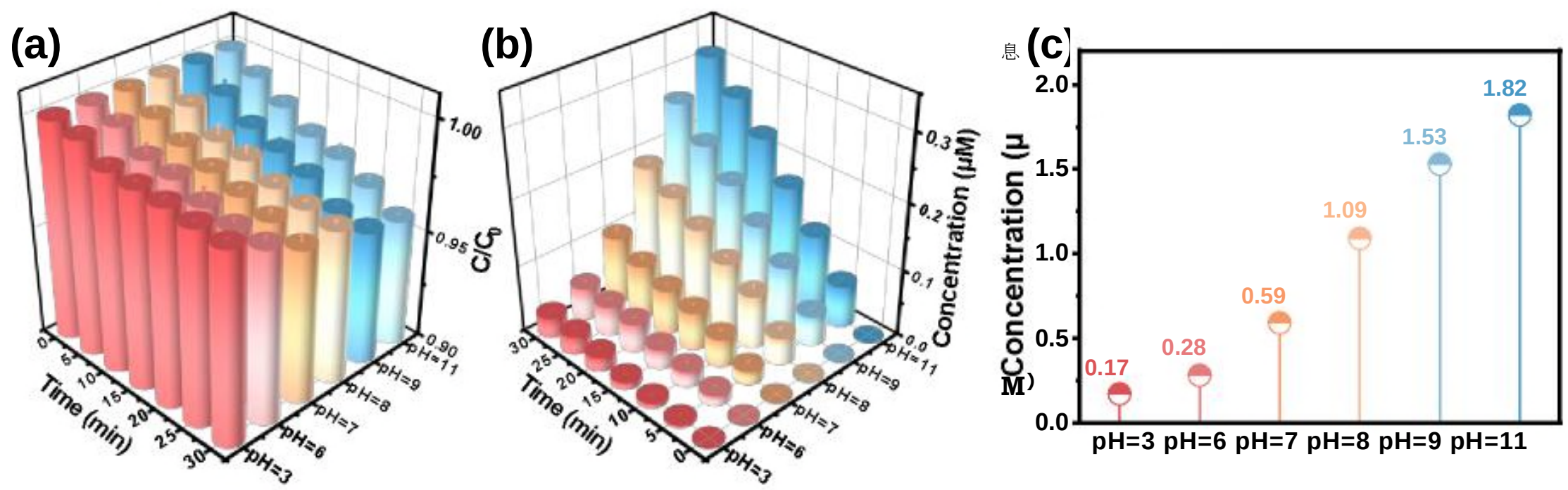

Supplementary Fig. 27 | Under different pH values in the UV_{222}-Br process, (a) degradation of BA, (b) the generated concentration of p-HBA, and (b) the cumulative concentration of •OH. Conditions: $[SDZ]_0$=20 uM, $[PAA]_0$=$[Br]_0$=0.5 mM, $[BA]_0$=0.2 mM, pH = 7.0.

Comment 6: The pH values selected in this work (3 and 11) seem to lack environmental relevance. To enhance the environmental applicability of the study, it is suggested to include a pH range that is more practically meaningful.

Response: We sincerely appreciate your insightful comment regarding the environmental relevance of the selected pH values, which helped us recognize this important issue and further improve the quality of the manuscript. Given that the pH of natural water bodies typically ranges from 6.0 to 9.0, with most waters being near neutral or weakly alkaline, we have therefore supplemented our study with additional experiments at pH 6, 8, and 9 to enhance the environmental relevance of this work. The detailed results are described in **Lines 584–621** and **Fig. 6a** of the revised manuscript.

The related descriptions and images are presented below for your convenience:

Lines 584–621: "The pH of solution is an important parameter that influences pollutant degradation and the production of reactive chemicals. Therefore, this study investigated the effect of the UV_{222}-PB system on the degradation of SDZ under

different pH conditions. It is evident that, in the UV_{222} system, SDZ exhibits the best degradation performance under neutral conditions (Supplementary Fig. 24). This is because SDZ, as an amphiprotic compound (pK_{a1} = 1.57, pK_{a2} = 6.5), exists in different forms depending on the pH of the solution. At pH 3, SDZ predominantly exists in its neutral form (SDZ^0). As the pH increases further, the deprotonated form ($SDZ^–$) gradually replaces SDZ^0 as the dominant species (Supplementary Fig. 25). DFT calculations indicate that compared to SDZ^+ (5.29 eV) and $SDZ^–$ (4.82 eV), SDZ^0 has a smaller HOMO-LUMO energy gap (4.67 eV), thus requiring lower energy for excitation, which results in a faster reaction rate (Supplementary Fig. 26)[9]. On the other hand, in the UV_{222}-Br system, increasing the $Br^–$ concentration from 0 to 1 mM gradually enhances the degradation efficiency of SDZ. Interestingly, the UV_{222}-Br process shows better degradation performance at pH 7. This phenomenon may be attributed to the reduced generation of •OH under acidic conditions (Supplementary Fig. 27), while alkaline conditions are unfavorable for the formation of HOBr[25]. Moreover, in UV_{222}-PB system, when the $Br^–$ concentration was within 50 μM, the removal efficiency of PB for SDZ correlated positively with pH. When the $Br^–$ concentration surpassed the threshold, an inverse pH–dependent relationship appeared (Fig. 6a and Supplementary Fig. 28). Under different pH values, the differences in SDZ degradation by UV_{222}-PB system are attributed to: (1) low $Br^–$ concentration. PAA oxidation of $Br^–$ results in limited HOBr, and SDZ elimination relies on the reactive species produced by PAA. However, the morphology of PAA (pK_a = 8.2) is influenced by pH. As the solution pH rises, deprotonated PAA ($PAA^–$) increasingly dominates. The reaction rate constant of $PAA^–$ ($k = 3.25 \times 10^{-5}\ s^{-1}$) activated by far-UVC in alkaline condition was higher than that of PAA ($k = 2.98 \times 10^{-5}\ s^{-1}$) in acidic condition, thus demonstrating superior removal efficiency of SDZ in alkaline environments[18]. (2) High $Br^–$ concentration. The pH of the solution affects the species distribution of SDZ and HOBr (pK_a = 8.8)[64]. Since $SDZ^–$ becomes the predominant species in alkaline conditions, electrostatic repulsion causes a decrease in the degradation rate. Furthermore, the oxidation potential of $PAA^–$ (1.01 V vs. SHE) was weaker than that of

PAA (1.75 V vs. SHE)[31]. Therefore, as the pH rose, the amount of HOBr formed by oxidation in system gradually decreased. Meanwhile, under alkaline conditions, HOBr underwent self-decomposition to form BrO^{-}, which exhibited weaker electrophilic and thus less efficient in attacking the sulfonamide group of SDZ and the amino groups in its aniline ring[23]. Therefore, compared to acidic environments, the UV_{222}-PB system with higher Br^{-} concentrations in alkaline circumstances exhibited significantly reduced degradation performance toward SDZ."

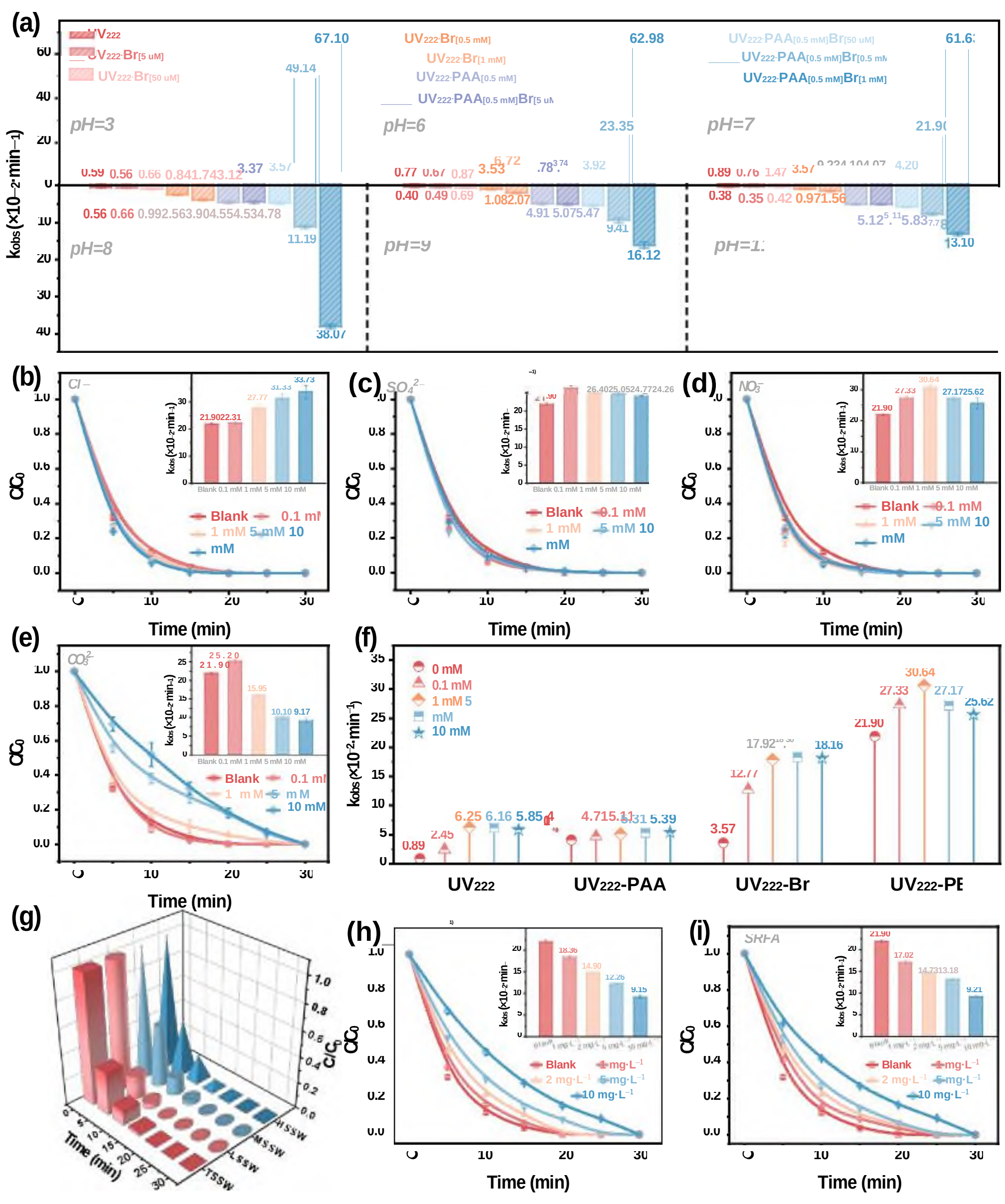

Fig. 6 | The Regulatory Effects and Mechanisms of Water Quality Parameters on

SDZ Degradation. (a) k_{obs} values of SDZ in different processes at multiple pH levels. Degradation curves and k_{obs} values of SDZ degraded by UV_{222}-PB in the presence of (b) Cl^-, (c) SO_4^{2-}, (d) NO_3^-, (e) CO_3^{2-}. (f) The effect of coexisting NO_3^- on the degradation of SDZ under UV_{222} using different processes. (g) The performance of UV_{222}-PB in simulated water bodies with different salinities. Degradation curves and k_{obs} values of SDZ degraded by UV_{222}-PB in the presence of (h) SRHA and (i) SRFA.

Comment 7: pH often alters the HOMO-LUMO energy gap of pollutants, thereby affecting their photodegradability. It is recommended that the authors supplement relevant DFT calculations to further clarify this aspect.

Response: Thanks for your careful review of the manuscript, which helped us recognize this important issue and further deepen the mechanistic understanding of our work. In response to your constructive suggestion, we performed DFT calculations to obtain the HOMO and LUMO energies of sulfadiazine (SDZ) in its protonated (SDZ+) and deprotonated (SDZ^-) forms, in order to better understand the pH-dependent performance differences of the UV_{222}-PB process. The relevant results have been added to **Lines 586–595** and **Supplementary Fig. 26** of the revised manuscript.

The related descriptions and images are presented below for your convenience:

Lines 586–595: "It is evident that, in the UV_{222} system, SDZ exhibits the best degradation performance under neutral conditions (Supplementary Fig. 24). This is because SDZ, as an amphiprotic compound ($pK_{a1} = 1.57$, $pK_{a2} = 6.5$), exists in different forms depending on the pH of the solution. At pH 3, SDZ predominantly exists in its neutral form (SDZ^0). As the pH increases further, the deprotonated form (SDZ^-) gradually replaces SDZ^0 as the dominant species (Supplementary Fig. 25). DFT calculations indicate that compared to SDZ^+ (5.29 eV) and SDZ^- (4.82 eV), SDZ^0 has a smaller HOMO-LUMO energy gap (4.67 eV), thus requiring lower energy for excitation, which results in a faster reaction rate (Supplementary Fig. 26)[9]."

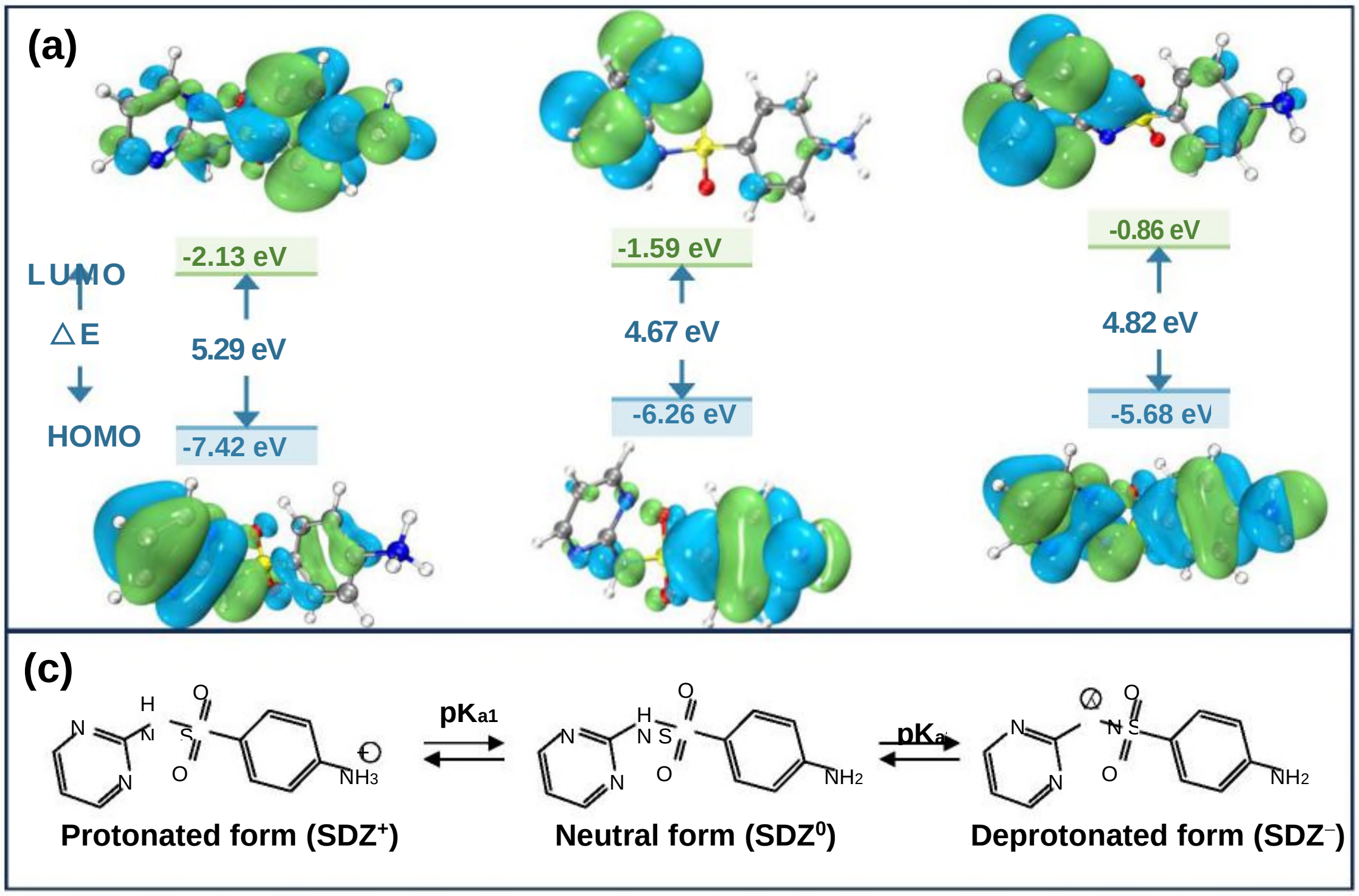

Supplementary Fig. 26 | (a) HOMO and LUMO, (b) molecular structures of SDZ under different existing forms.

Comment 8: In Fig. 3(a), the color contrast is insufficient. Please use colors with higher contrast for better distinction.

Response: We sincerely appreciate your valuable suggestion. In the revised manuscript, we have replaced the colors using a higher-contrast color scheme to improve visual clarity. Furthermore, **Fig. 3(a) in the original manuscript has been changed to Fig. 6(a)** in the revised version. We once again appreciate your attention to the quality of figure presentation, which has helped further enhance the quality of our manuscript.

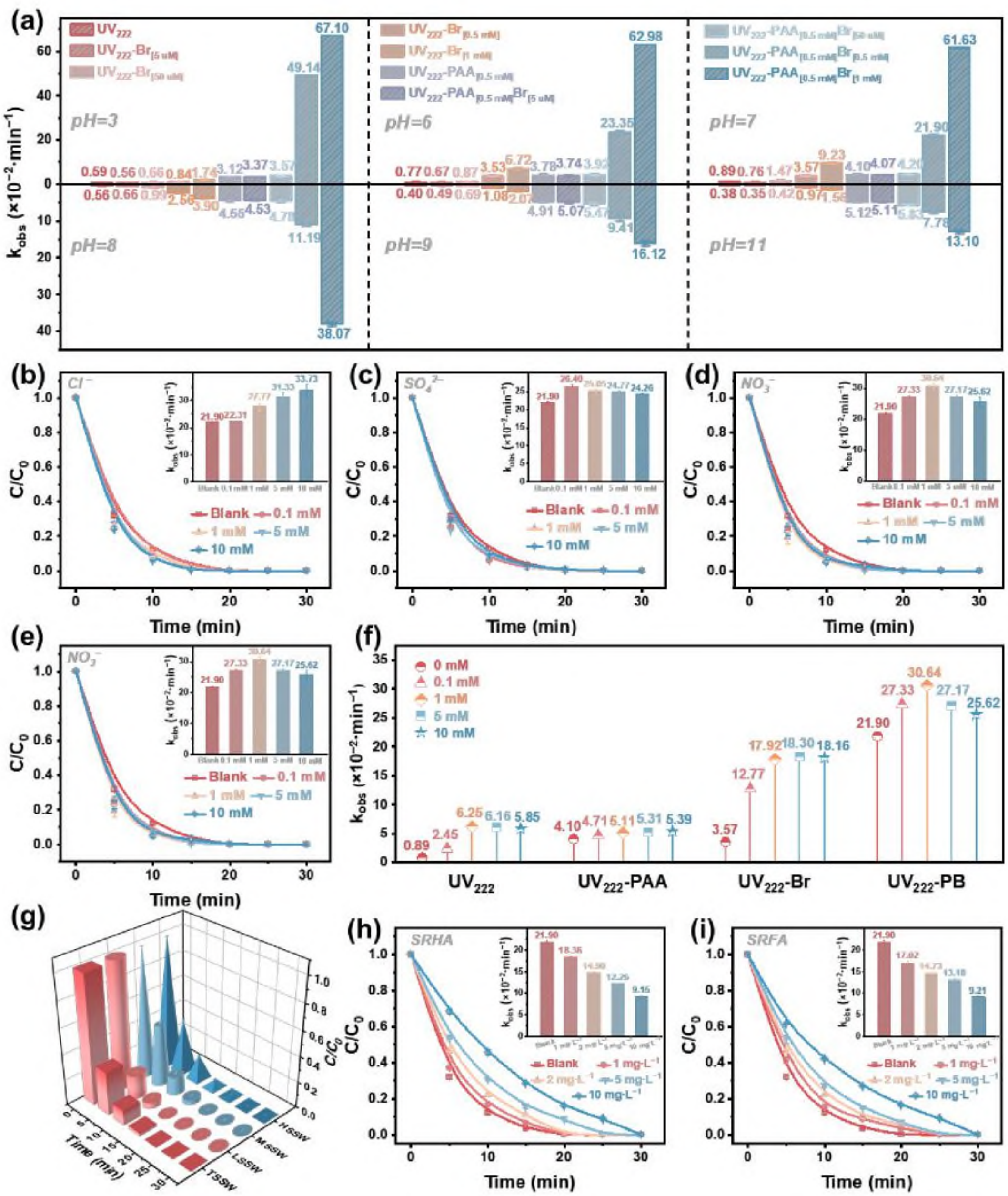

Fig. 6 | The Regulatory Effects and Mechanisms of Water Quality Parameters on SDZ Degradation. (a) k_{obs} values of SDZ in different processes at multiple pH levels. Degradation curves and k_{obs} values of SDZ degraded by UV_{222}-PB in the presence of (b) Cl^-, (c) SO_4^{2-}, (d) NO_3^-, (f) CO_3^{2-}, (h) SRHA, and (i) SRFA. (e) The effect of coexisting NO_3^- on the degradation of SDZ under UV_{222} using different processes. (g) The performance of UV_{222}-PB in simulated water bodies with different salinities.

Comment 9: Lines 408-409: LC-MS data for SDZ degradation are missing.

Response: Thank you for pointing out this omission in the manuscript. In response to your comment, we have supplemented the LC-MS analysis data of SDZ degradation intermediates in **Supplementary Fig. 19** of the Supplementary Information. Once again, we appreciate your careful review, which has helped improve the completeness of our study.

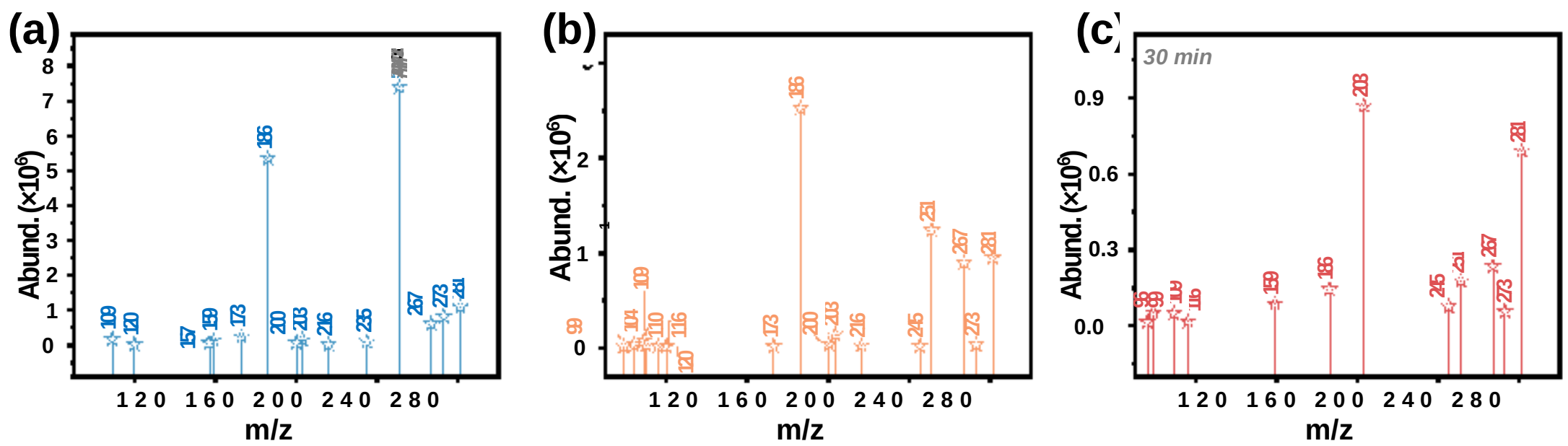

Supplementary Fig. 19 | LC-MS analysis of SDZ in the UV_{222}-PB system at (a) 10 min, (b) 20 min, and (c) 30 min.

Comment 10: The Discussion requires improvement. Please elaborate further on the practical environmental implications of the UV_{222}-PB process and offer forward-looking guidance on the application prospects of far-ultraviolet technology.

Response: We greatly appreciate your constructive suggestions on our manuscript. In response to your comments, we have further expanded the Discussion section to better clarify the practical environmental implications of the UV_{222}-PB process and to provide forward-looking guidance on far-UVC technology. The detailed content has been added to the Discussion section of the revised manuscript (**Lines 849–882**). We thank you again for your guidance, which has strengthened the practical applicability and technological implications of this work.

The related descriptions and images are presented below for your convenience:

Lines 849–882: "Previous studies have demonstrated that 222 nm-based water treatment technology holds significant application prospects. Its energy-efficient, high-performance, and environmentally friendly characteristics align well with current

sustainable development goals, making it crucial for achieving clean drinking water and sanitation. In this work, PAA and Br^- exhibited distinct light absorption characteristics in the 222 nm far-UVC region, with their ε values increasing by 6.45 and 39 times, respectively, compared to those at 254 nm. Consequently, UV_{222}-PB achieved superior degradation performance and lower energy consumption costs. The enhanced degradation performance is attributed to the effective activation and interaction of PAA and Br^- under UV_{222} irradiation, generating more RBS and •RO that accelerate the degradation rate of SDZ. This is supported by quenching experiments, probe tests, ESR analysis, and kinetic modeling. Due to the predominantly electrophilic nature of the reactive species in the system, the $\ln(k_{obs})$ values of ten OMPs with different structures showed a significant correlation with their IP and E_{HOMO} values, confirming the high selectivity of the UV_{222}-PB process toward electron-rich pollutants. Furthermore, the environmental applicability and eco-friendliness of the process were further evaluated. The degradation rate of SDZ in the UV_{222}-PB system exhibited a strong dependence on solution pH, with acidic and neutral conditions being more favorable for pollutant degradation. The process also demonstrated relatively stable performance in the presence of inorganic anions and NOM, across mixed-anion systems covering a wide salinity range, and in various real water matrices.

Regarding the potential risks of DBPs formation, the study monitored bromate formation in different processes and conducted toxicity assessments from multiple perspectives, including the ECOSAR model, wheat and amaranth seed germination tests, and zebrafish embryo culture. The results showed that the ecotoxicity of the treated water was significantly reduced. LCA further confirmed the environmental friendliness and sustainability of the UV_{222}-PB process. However, since the UV_{222}-PB process does not involve catalyst preparation, electricity input remains the main factor affecting the environmental pressure of the process. In future development, it is recommended to operate electrical equipment under saturated load conditions, which would contribute to reducing the environmental burden. In summary, this work highlights the feasibility of coupling oxidants with natural water matrix components at

222 nm, offering a new approach for the degradation of OMPs under far-ultraviolet light."

Reviewer #3

General comments: *In the manuscript entitled "Bromide-Mediated Enhancement of Far-UVC/Peracetic Acid Process for Selective Micropollutant Abatement", the authors systematically elucidates the synergistic mechanism between bromide ions (Br^-) and peracetic acid (PAA) under far-UVC irradiation at 222 nm, confirming that the UV_{222}-PB process achieves selective oxidative degradation of electron-rich pollutants primarily through the efficient generation of organic radicals (•RO) and hypobromous acid (HOBr). The process demonstrates stable performance across various real water matrices and significantly reduces ecotoxicity in treated water samples. Life cycle assessment further supports its environmental sustainability. This work provides a theoretical foundation and technical support for the application of far-UVC in water treatment. The following comments are offered:*

Response: We sincerely thank you for your affirmation of our work, and your recognition has greatly encouraged us. In response to your valuable and constructive comments, we have revised the manuscript accordingly to make it more rigorous and complete. The specific revisions have been detailed in the point-by-point response. Once again, we gratefully acknowledge your thorough review and professional insights, which have been highly beneficial to us. We sincerely hope that our responses and revisions meet your satisfaction.

Comment 1: In the introduction, when presenting the research background of the UV_{222} light source, the comparison with traditional mercury lamps only mentions the risk of mercury leakage. It is recommended to also discuss the differences in energy efficiency, light absorption characteristics, and practical application costs between the two light sources to further highlight the application value of UV_{222} as a green light source.

Response: I appreciate your valuable comments on the introduction section, which have helped us more comprehensively highlight the application value of the emerging UV_{222} light source. In response to your suggestions, we have supplemented the introduction with a discussion of the advantages of UV_{222} over conventional mercury lamps in terms of photon energy, energy efficiency and economic feasibility, light absorption characteristics, oxidant activation efficiency, and pollutant degradation performance. The relevant content can be found in **Lines 55–77** of the revised manuscript. Once again, we appreciate your thorough review and constructive suggestions, which have made the application background of this work more comprehensive and rigorous.

The related descriptions are presented below for your convenience:

Lines 55–77: "At present, the ultraviolet radiation sources commonly used in UV-AOP are 254 nm low-pressure ultraviolet (LPUV) lamps and 200–400 nm medium-pressure ultraviolet (MPUV) lamps[9]. However, conventional mercury lamps generally suffer from low photonic quantum efficiency and insufficient utilization of oxidizing agents, making it difficult to efficiently degrade large amounts of OMPs at 254 nm and longer wavelengths[10]. Moreover, mercury lamps pose risks such as mercury leakage and mercury contamination during production and use, causing harm to soil and water environments[11]. Therefore, the utilization of environmentally friendly light sources with high photochemical utilization has become a trend in the development of UV-AOPs.

Currently, krypton chloride (KrCl*) excimer lamp has emerged as an outstanding candidate for UV-AOPs systems. Compared with conventional low-pressure mercury lamps, the UV_{222} emerging UV light source offers advantages such as being mercury-

free, having higher photon energy, and being safer for human health[12]. Specifically, the photon energy of UV_{222} (539 kJ·mol^{-1}) is higher than that of UV_{254} (471 kJ·mol^{-1}), enabling it to break stronger chemical bonds and activate various oxidants more efficiently[13]. In multiple studies, UV_{222} outperformed UV_{254} in the direct photolysis of OMPs and the generation of reactive species, requiring a lower UV dose and thereby significantly reducing treatment energy consumption and associated economic costs[10,14]. Hu et al. reported that at the same UV dose, the UV_{222}/PAA process achieved a removal efficiency of 90.8% for carbamazepine (CBZ), compared to only 15.1% for UV_{254}/PAA, while also exhibiting better electrical energy per order (EE/O) performance and lower energy consumption[15]."

Comment 2: Although the research objectives are listed in the introduction, their logical connections are not clearly articulated, and the link between these objectives and the results presented in later sections is not always explicit. It is suggested to sort out the logical relationship and express it in a more hierarchical manner, such as from basic performance research to mechanism analysis, then to toxicity and practical application evaluation.

Response: Our sincere thanks for your careful review and professional comments on the introduction section. In response to your valuable suggestions, we have restructured the research objectives presented in **Lines 117–129** of the revised manuscript. **Accordingly, we have also adjusted the order of the research sections in the manuscript to achieve a tighter logical connection among the study contents.** Once again, we appreciate your guidance, which has been very helpful in improving the logical clarity of our research.

The related descriptions are presented below for your convenience:

Lines 117–129: "This research was based on the following explicit objectives: (1) First, to compare the degradation efficiency and economic feasibility of different processes under irradiation with multiple wavelength light sources. (2) Subsequently, to employ various analytical techniques, theoretical calculations, and kinetic models to

deeply elucidate the generation of reactive species, synergistic mechanisms, and selective degradation mechanisms in the UV_{222}-PB process. (3) To reveal the transformation pathways of OMPs in the UV_{222}-PB process in conjunction with theoretical calculations, and to evaluate the ecotoxicological effects and the formation patterns of disinfection byproducts (DBPs). (4) Finally, to comprehensively evaluate the environmental stability, practical application potential, and environmental-economic impacts of the UV_{222}-PB process. Collectively, this research reveals the interaction between oxidants and natural water matrices at 222 nm and its environmental implications, providing insightful guidance for practical water treatment applications."

Comment 3: In the section "Effect of ultraviolet wavelength on the performance of the UV-PAA/Br process", only sulfadiazine (SDZ) was used as a model pollutant to verify the synergistic effect of UV_{222}-PB. What is the degradation efficiency for other typical trace pollutants? It is recommended to include degradation data for one or two additional representative micropollutants to demonstrate the universality of the wavelength effect.

Response: Thank you for reminding us to focus on the universality of the wavelength effect, which is crucial to the conclusions of this study. In response to your valuable and constructive comments, we selected four typical aquatic environmental OMPs belonging to different classes (sulfonamides, fluoroquinolones, psychotropic drugs, and tetracyclines), namely sulfamethazine (SMZ), ciprofloxacin (CIP), carbamazepine (CBZ), and tetracycline hydrochloride (TC). These pollutants exhibit significant differences in chemical structures and functional groups, allowing for a thorough evaluation of the applicability of the UV_{222}-PB process to various types of OMPs. The relevant descriptions have been added in **Lines 167–186**, **Fig. 1f**, **Supplementary Fig. 4** and **Supplementary Table2**. The results demonstrate that the UV_{222}-PB process achieves significant degradation efficiency and synergistic effects for all five OMPs. These experimental findings further support the broad applicability

of the UV_{222}-PB process. Once again, we appreciate your meticulous guidance, which is highly important for enhancing the generalizability of the conclusions of this study. The related descriptions and charts are presented below for your convenience:

Lines 167–186: "Furthermore, four typical aquatic environmental OMPs with significantly different chemical structures and functional groups, namely sulfamethazine (SMZ), ciprofloxacin (CIP), CBZ, and tetracycline hydrochloride (TC), were also selected to evaluate the synergistic degradation effect of the UV_{222}-PB process (Fig. 1f and Supplementary Fig. 4). The results showed that upon the introduction of Br^-, the k_{obs} values of SMZ and TC increased to 0.0221 min^{-1} and 0.0376 min^{-1}, respectively, due to the presence of electron-rich fused rings and aniline groups in their structures, which exhibit high reactivity with bromine radicals[28]. Meanwhile, in the UV_{222}-PAA system, the degradation efficiencies of SMZ (0.0335 min^{-1}), CIP (0.0219 min^{-1}), CBZ (0.0094 min^{-1}), and TC (0.0623 min^{-1}) were all enhanced to varying degrees. Notably, compared with direct photolysis, the degradation of all four OMPs was significantly improved in the UV_{222}-PB process. Particularly for TC, its k_{obs} value increased from 0.0108 min^{-1} to 1.1028 min^{-1}, providing new and strong evidence that photoreactive species play a dominant role in pollutant degradation in the UV_{222}-PB process. Specifically, the k_{obs} values of SMZ, CIP, CBZ, and TC in the UV_{222}-PB process were 4.71, 3.31, 1.97, and 11.04 times the sum of the k_{obs} values of the UV_{222}-PAA and UV_{222}-Br processes, respectively (Supplementary Table 2). This further confirms the significant synergistic effect between PAA and Br^- under UV_{222} irradiation, demonstrating that the UV_{222}-PB process can achieve effective degradation of various OMPs."

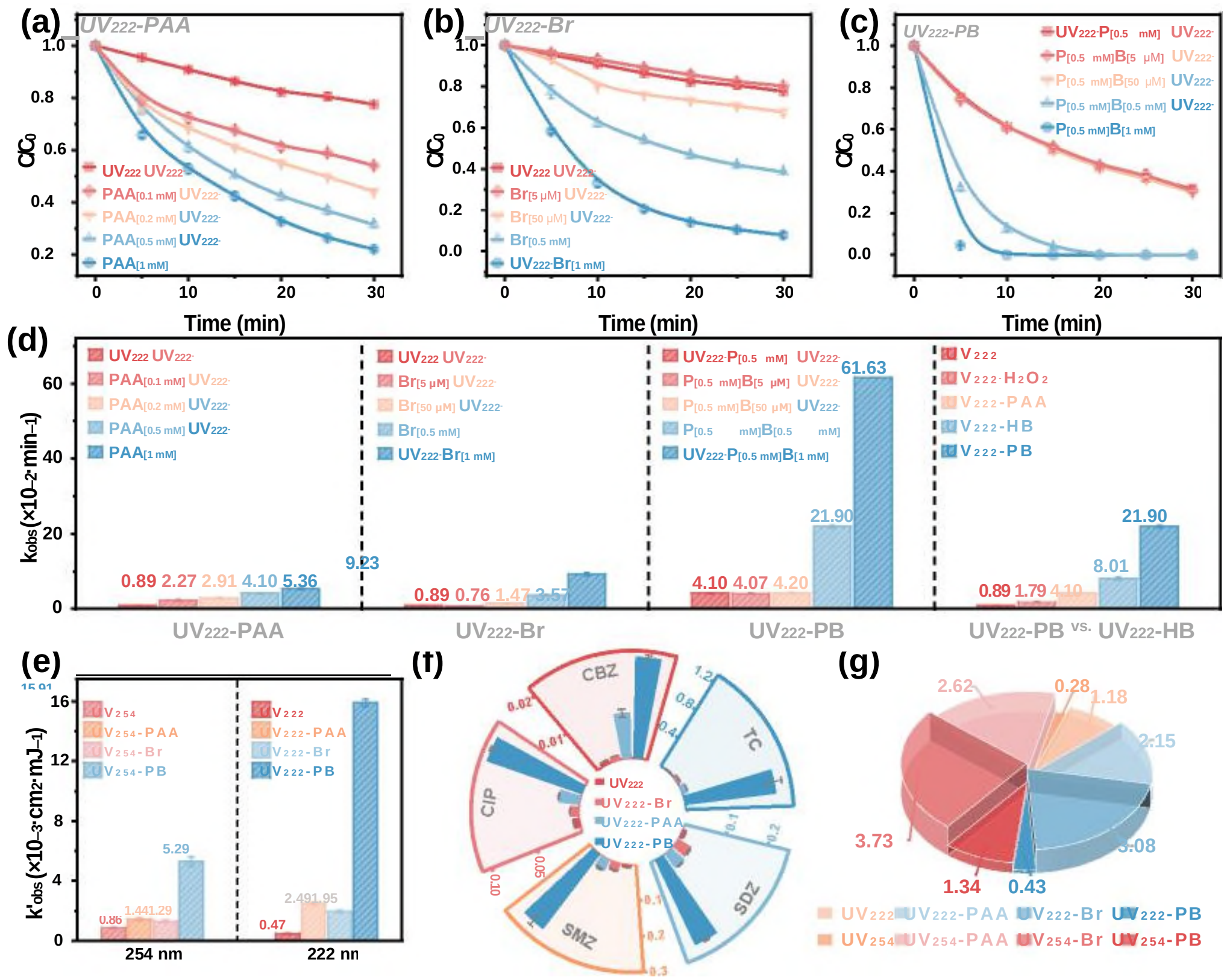

Fig. 1 | Efficient SDZ degradation and characteristic evaluation under UV-based processes. (a–c) SDZ degradation curves under various UV_{222} processes, and (d) k_{obs} values. (e) k'_{obs} values for various UV_{222} and UV_{254} processes. (f) k_{obs} values for the degradation of various OMPs under the UV_{222} process. (g) EE/O values during SDZ degradation using various processes. Conditions: $[SDZ]_0 = 20$ μM, pH = 7.0, $[H_2O_2]_0 = 200$ μM, $[PAA]_0 = 0.1$ mM-1 mM, $[Br]_0 = 5$ μM-1 mM (unless otherwise mentioned, $[PAA]_0 = [Br]_0 = 0.5$ mM).

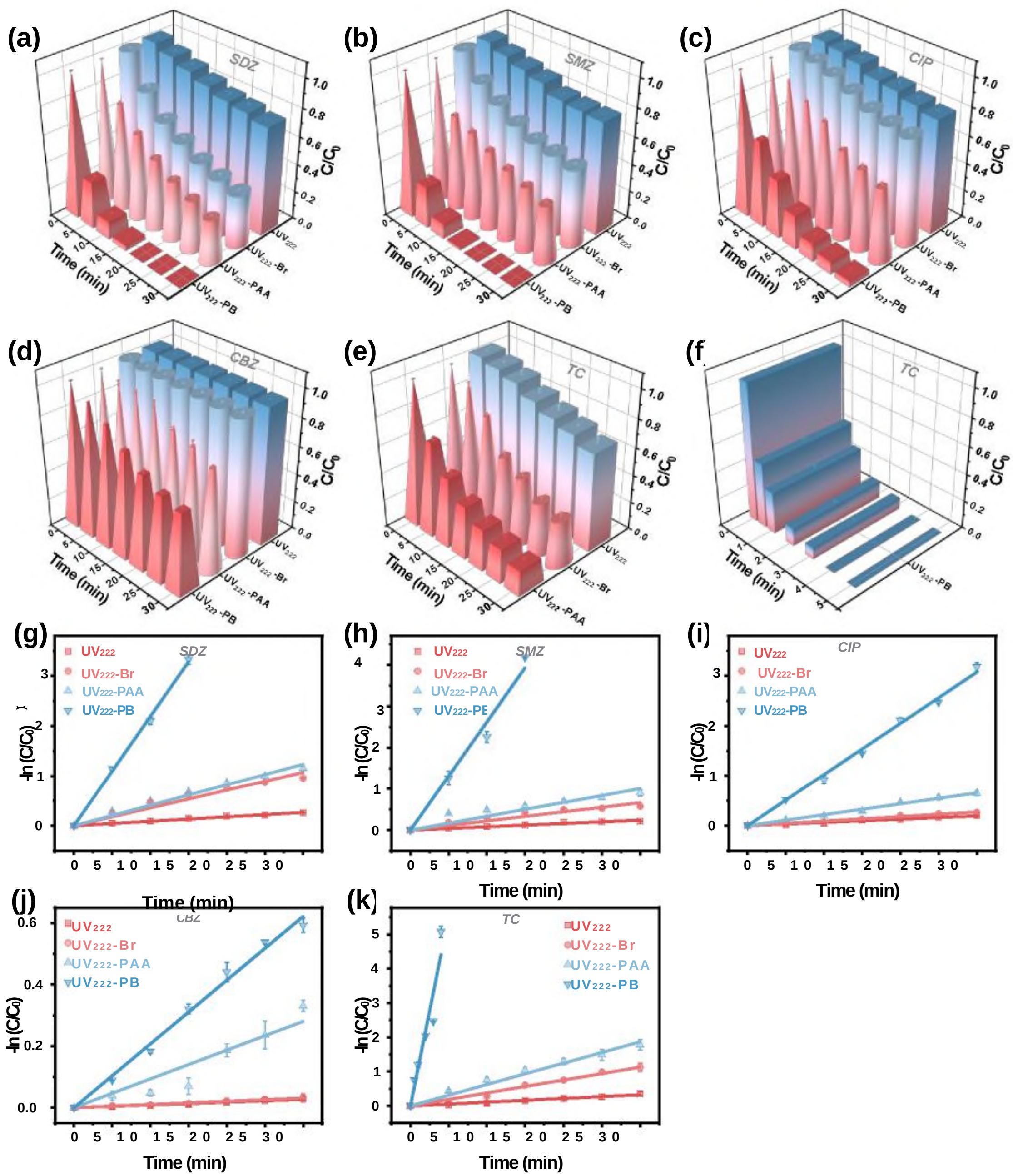

Supplementary Fig. 4 | (a, g) SDZ, (b, h) SMZ, (c, i) CIP, (d, j) CBZ, and (e–f, k) TC degradation curves and pseudo-first-order kinetic fitting curves under different UV_{222} processes. Conditions: $[OMPs]_0$=20 uM, pH=7.0, $[PAA]_0$=$[Br]_0$=0.5 mM.

Supplementary Table 2 | k_{obs} values of OMPs under UV_{222} irradiation for different processes.

OMPs	UV_{222}	UV_{222}-Br	UV_{222}-PAA	UV_{222}-PB
SDZ	0.0089 min^{-1}	0.0357 min^{-1}	0.0410 min^{-1}	0.2190 min^{-1}
SMZ	0.0080 min^{-1}	0.0221 min^{-1}	0.0335 min^{-1}	0.2620 min^{-1}
CIP	0.0065 min^{-1}	0.0091 min^{-1}	0.0219 min^{-1}	0.1025 min^{-1}
CBZ	0.0009 min^{-1}	0.0011 min^{-1}	0.0094 min^{-1}	0.0207 min^{-1}
TC	0.0108 min^{-1}	0.0376 min^{-1}	0.0623 min^{-1}	1.1028 min^{-1}

Comment 4: When calculating the Electrical Energy per Order (EE/O) value for economic feasibility analysis, the authors provide EE/O values for different systems but do not detail the calculation method or parameter settings. It is suggested to supplement relevant calculation details in the main text or Supplementary materials.

Response: We sincerely appreciate your careful review and guidance on the manuscript, which has helped improve its completeness. Following your suggestion, we have added a detailed explanation of Electrical Energy per Order (EE/O) in **Lines 101–119** of the supplementary information.

The related descriptions are presented below for your convenience:

Text S6. The calculation of EE/O and electricity cost.

Electrical Energy per Order (EE/O) can be used to evaluate energy consumption efficiency. An economic assessment of the practical application potential of systems such as UV_{222}-PB was conducted using EE/O. The total EE/O_T of the system includes the electrical energy consumption ($EE/O_{UV222,\ UV254}$) for UV_{254} or UV_{222} irradiation, the equivalent electrical energy consumption (EE/O_{PAA}) for PAA, and the equivalent electrical energy consumption (EE/O_{Br}) for Br^{-}, which can be calculated using equations S11–S14:

$$EE/O_T = EE/O_{UV222,\ UV254} + EE/O_{PAA} + EE/O_{Br} \qquad \text{(S11)}$$

$$EE/O_{UV222,UV254} = \frac{P \times t}{V \times \log\left(\frac{C_0}{C_t}\right)} \qquad \text{(S12)}$$

$$EE/O_A = \alpha_A \times \frac{[A]\times 1000}{\log(\frac{C_0}{C_t})} \quad \text{(S13)}$$

$$\alpha_A = \frac{Price \times M_A}{10^6 \times Purity \times AEC} \quad \text{(S14)}$$

Where P denotes the total electrical power input to the system (kW), t represents the reaction time (h), V denotes the reaction solution volume (L, V = 0.05 L), C_0 and C_t represent the SDZ concentrations in the solution at 0 and t min, respectively (mol·L^{-1}), α_A is the energy consumed per mole of substance produced by equivalent electricity generation (kW·h·mol^{-1})[3], [A] denotes the concentration of Br^- or PAA (mol·L^{-1}), Price represents the average price of Br^- (RMB·ton^{-1}), M_A is the molar mass (g·mol^{-1}), Purity indicates the purity (%), and AEC denotes the average electricity cost (0.65 RMB·kW^{-1}·h^{-1})[4]. The average price of KBr (99% by weight) on Alibaba.com was 10000 RMB·ton^{-1} (2025). Therefore, α_{PAA} and α_{Br} are 1.41 kW·h·mol^{-1} and 1.85 kW·h·mol^{-1}, respectively. The calculated EE/O values for different process systems are shown in Fig. 1g.

Comment 5: The quenching experiments have methodological limitations. For Mn^{2+} used as a quencher for •RO, it has not been verified whether Mn^{2+} undergoes side reactions under 222 nm irradiation, reacts non-specifically with PAA or Br^-, or competes for photons. Moreover, only experiments with varying Mn^{2+} concentrations were conducted; control experiments (e.g., in the dark without UV_{222}) to assess potential direct interactions between Mn^{2+} and SDZ, PAA, or Br-are missing. Such controls are necessary to confirm the specificity of Mn^{2+} as a quencher.

Response: We sincerely appreciate your comprehensive review of the manuscript and for your expert comments. In response to your valuable suggestions, we have performed the following additional experiments: (1) The 100 mM Mn^{2+} solution was irradiated alone at UV_{222} for 30 min, and no significant change in Mn^{2+} concentration was detected. (2) Compared with the UV_{222}-PB system without Mn^{2+} (in the absence of SDZ), the addition of 100 mM Mn^{2+} had almost no effect on the concentration changes

of the various components in the system. (3) UV absorption spectroscopy analysis showed that 100 mM Mn^{2+} exhibits no significant light absorption at 222 nm, and its impact on the photolysis process is minor. (4) In the dark, 100 mM Mn^{2+} was separately incubated with SDZ, PAA, and Br^{-} for 30 min. The results showed no significant concentration changes of Mn^{2+} or the respective reactants, indicating that Mn^{2+} does not directly react with these components in the absence of UV_{222} irradiation. Collectively, these results confirm that Mn^{2+} can serve as an effective quencher for acylperoxyl radicals. The detailed results are presented in **Supplementary Figs. 8, 15– 16**, and the corresponding descriptions have been added in **Lines 338–347** of the revised manuscript. Once again, we appreciate your rigorous review, which has significantly enhanced the scientific rigor of this study.

The related descriptions and images are presented below for your convenience:

Lines 338–347: "Fortunately, when 100 mM Mn^{2+} was separately incubated with SDZ, PAA, and Br^{-} in the dark for 30 min, no significant concentration changes were observed (Supplementary Figs. 15–16), indicating that Mn^{2+} does not interact with the components in the system. UV absorption spectroscopy showed that Mn^{2+} exhibits weak light absorption at 222 nm (Supplementary Fig. 8b). When 100 mM Mn^{2+} solution was irradiated alone at UV_{222} for 30 min, no decrease in Mn^{2+} concentration was detected, suggesting that the impact of Mn^{2+} on the photolysis process is negligible. Finally, the addition of Mn^{2+} to the UV_{222}-PB system in the absence of pollutants had almost no effect on the concentration changes of the various species. Therefore, Mn^{2+} can serve as an effective quencher for acylperoxyl radicals in the UV_{222}-PB process."

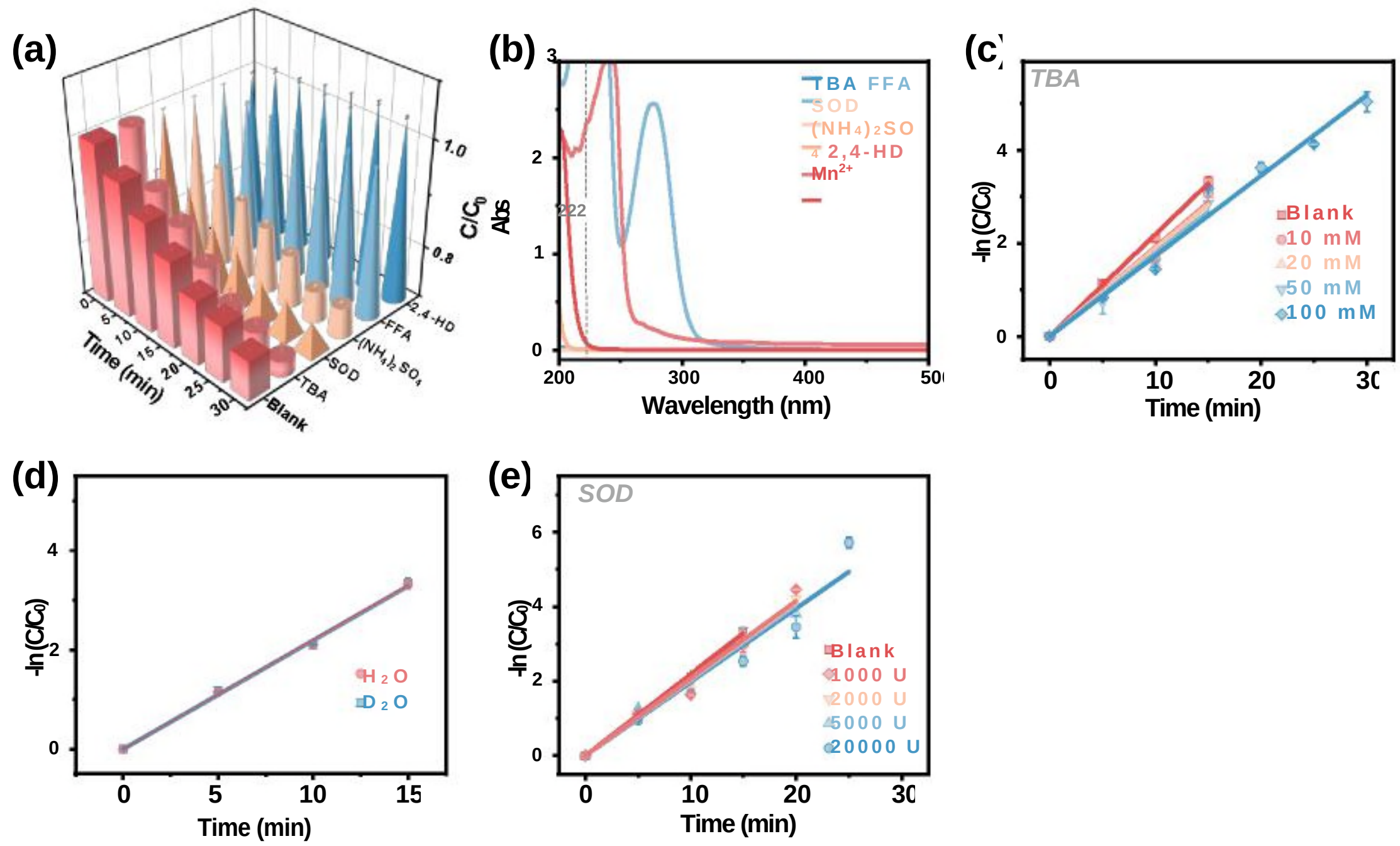

Supplementary Fig. 8 | (a) Evaluation of interference experiments and (b) absorption spectra for different quenchers at a concentration of 100 mM. Pseudo-first-order kinetic fitting curves of SDZ degradation in the UV_{222}-PB system at different (c) TBA, (d) solvents, (e) SOD concentrations. Conditions: $[SDZ]_0$=20 uM, pH=7.0, $[PAA]_0$=$[Br]_0$=0.5 mM.

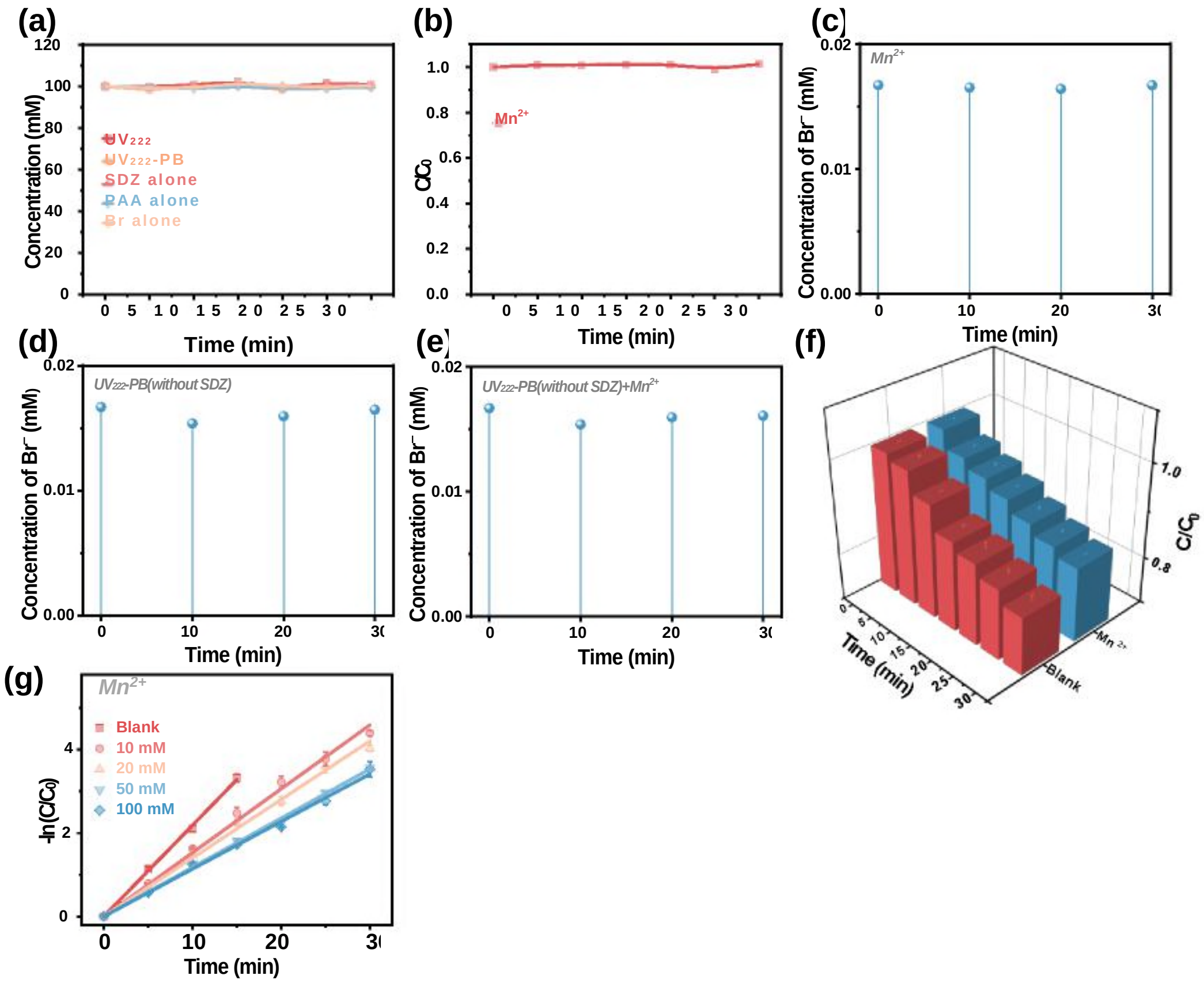

Supplementary Fig. 15 | (a) Concentration changes of 100 mM Mn^{2+} under different processes. (b) Degradation of SDZ by 100 mM Mn^{2+}. Concentration changes of Br^- in the system of (c) 100 mM Mn^{2+}, (d) UV_{222}-PB (without SDZ), and (e) UV_{222}-PB (without SDZ) + 100 mM Mn^{2+} (Br^- concentration diluted 30 times). (f) The effect of 100 mM Mn^{2+} on the degradation of SDZ in the UV_{222} system. (g) Pseudo first-order kinetic fitting curve of SDZ at different concentrations of Mn^{2+}. Conditions: $[SDZ]_0$=20 uM, pH=7.0, $[PAA]_0$=$[Br]_0$=0.5 mM, $[Mn^{2+}]_0$=10 mM-100 mM.

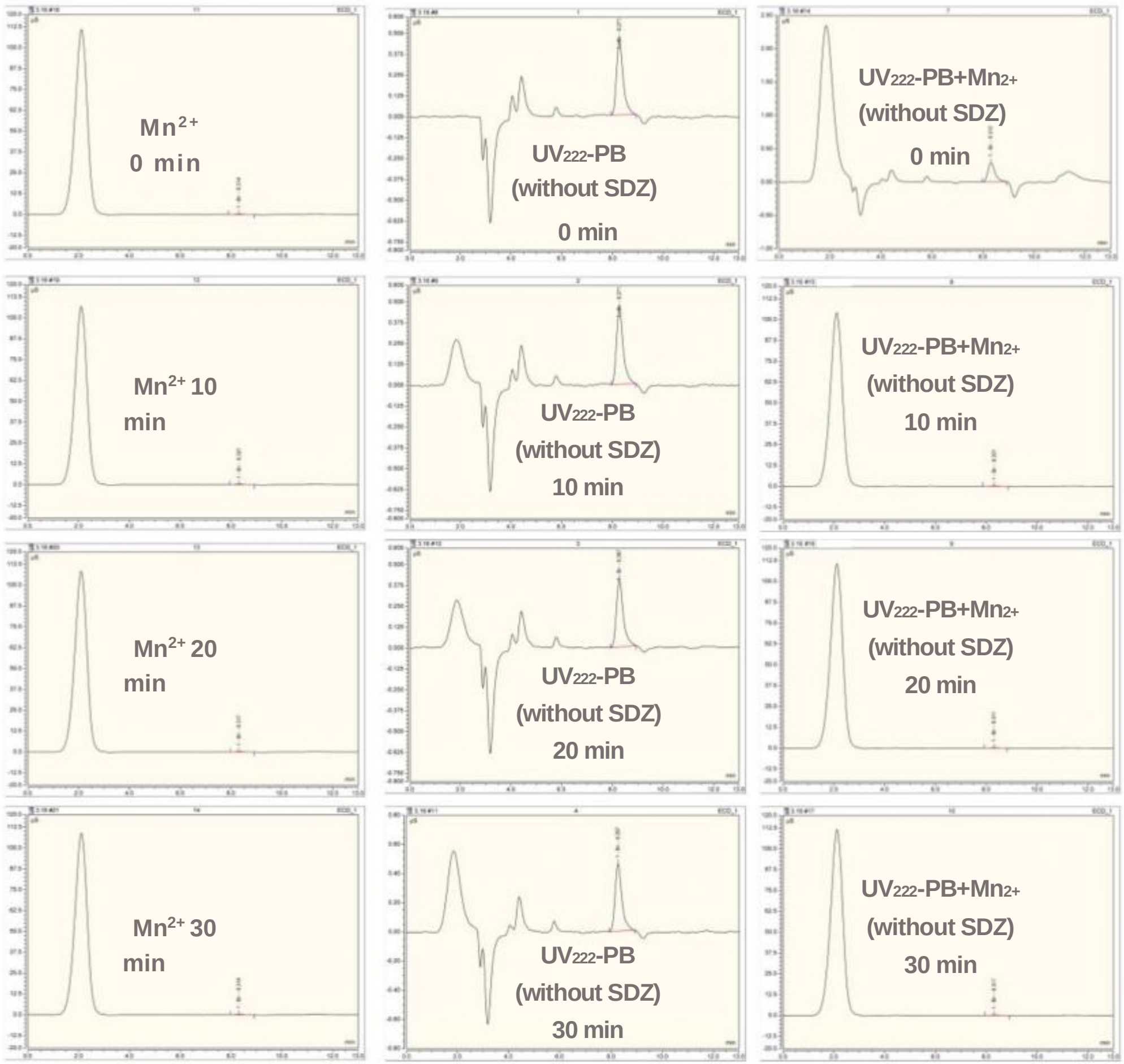

Supplementary Fig. 16 | Ion chromatograms of Br^- at different time points for 100 mM Mn^{2+}, UV_{222}-PB (without SDZ), and UV_{222}-PB (without SDZ) + 100 mM Mn^{2+} (Br^- concentration diluted 30 times).

Comment 6: The authors only specifically measured the Br^- concentration at the initial stage and at different time points, but did not systematically quantify intermediate reactive bromine species or transformation products involved in the bromine cycle. Moreover, the proposed dynamic Br^- cycling process is inferred rather than quantitatively tracked. Since this cycling is central to explaining why trace amounts of Br^- significantly enhance degradation efficiency, the lack of comprehensive bromine speciation and quantitative tracking weakens the evidence supporting this core mechanism.

Response: Thank you very much for your insightful comments on the bromine

cycling mechanism in the UV_{222}-PB process, which are crucial for improving our mechanistic study. To better address your questions, we monitored the formation of bromate (BrO_3^-) and total organic bromine (TOBr) in the UV_{222}-PB process, and constructed a kinetic model covering PAA photolysis, radical chain reactions, interconversion of bromine species, and the terminal degradation pathways of SDZ. This model quantified the contribution of each reactive species to SDZ degradation and further confirmed the Br^- cycling mechanism. The results showed that the BrO_3^- concentration was below the detection limit, and the TOBr formation was also very low, indicating that Br^- in the system mainly participates in the cyclic reaction rather than being converted into other inorganic or organic bromine products. On the other hand, the model results revealed that HOBr and •Br were the two major bromine-related pathways, together contributing 63.05% of SDZ degradation. Meanwhile, the model predicted that the Br^- concentration remained almost unchanged during the process, confirming the catalytic cycling role of bromide ions. Furthermore, both the model and experimental data demonstrated that the residual H_2O_2 after the reaction was still sufficient to continuously reduce HOBr back to Br^-, thereby further ensuring the smooth operation of the bromine cycle. Detailed descriptions of BrO_3^-, TOBr, and the kinetic model are presented in **Lines 311–317, 385–425**, **Fig. 2h–i** and **Supplementary Figs. 14c, 17, 23** of the revised manuscript. Once again, we appreciate your rigorous review, which has helped us strengthen the mechanistic evidence for the bromine cycle.

The related descriptions and images are presented below for your convenience:

Lines 311–317: "In addition, the study monitored changes in the concentrations of bromate (BrO_3^-) and total organic bromine (TOBr) during the reaction (Supplementary Figs. 14c, 23). The results indicate that virtually no BrO_3^- was generated in the UV_{222}-PB system, with its concentration remaining below the detection limit. Meanwhile, the concentration of TOBr was also extremely low, accounting for only about 1% of the total bromine, and it began to decrease gradually as the reaction progressed."

Lines 385–425: "A kinetic model was used to further quantify the contribution of

each active substance to the degradation of SDZ in the UV_{222}-PB process. The predicted values from the kinetic model were in good agreement with the experimental observations, demonstrating that the constructed kinetic model provides a relatively accurate description of SDZ degradation (Supplementary Figs. 17a–c). The model predicted the concentration changes of various active species and the instantaneous rates of the major pathways to further evaluate the dominant species in the SDZ transformation process (Supplementary Figs. 17d–g). Specifically, throughout the reaction, •Br was the dominant bromine species, consistently exhibiting the highest instantaneous rate. Terminal contribution analysis revealed that although the steady-state concentration of •Br was approximately four orders of magnitude lower than that of HOBr, its contribution to SDZ transformation was substantially higher (Figs. 2h–i). This is because the reaction rate of HOBr (1.78×10^5 $M^{-1}\cdot s^{-1}$) with SDZ is less than one-hundred-thousandth that of •Br (4.28×10^{10} $M^{-1}\cdot s^{-1}$). On the other hand, in the •RO system, although the reaction rate of $•CH_3C(O)O$ with SDZ is slightly higher than that of $•CH_3C(O)OO$, its resulting concentration is approximately four orders of magnitude lower than that of $•CH_3C(O)OO$. Consequently, $•CH_3C(O)OO$ remained the dominant pathway for SDZ degradation. In summary, the major reactive species responsible for SDZ degradation in the UV_{222}-PB process were •Br (34.13%), $•Br_2^-$ (20.44%), HOBr (12.71%), and $•CH_3C(O)OO$ (11.98%), while •OH contributed only 6.11%, and contributions from other reactive oxygen species were relatively minor.

Furthermore, the kinetic model effectively validated the Br^- cycling mechanism. The model predictions showed that after the reaction started, Br^- first reacted with PAA to generate HOBr, while simultaneously generating •Br under UV_{222} irradiation. Subsequently, HOBr and •Br were further converted into other reactive bromine species such as $•Br_2^-$ and Br_2, which rapidly attacked SDZ and led to its oxidative degradation. The fitting results indicated that the Br^- concentration remained almost unchanged during the UV_{222}-PB process, and the concentrations of HOBr, •Br, and other species gradually approached steady state, suggesting that a dynamic equilibrium was established among the reactive bromine species, and Br^- accomplished a closed loop of

oxidation, radical chain reactions, and regeneration. In addition, considering the crucial role of H_2O_2 in the Br^- cycling and regeneration process, the study tracked the concentration changes of PAA and H_2O_2 during the reaction using both experimental measurements and model predictions (Supplementary Fig. 17f). Obviously, the experimental values and model predictions agreed well over the entire time scale. At the end of the reaction, the consumption rates of PAA and H_2O_2 were approximately 45.19% and 36.90%, respectively. Given that the second-order reaction rate constant between H_2O_2 and HOBr is 1.50×10^4 $M^{-1} \cdot s^{-1}$, and the residual concentration of H_2O_2 (0.1262 μM) is much higher than the steady-state concentration of HOBr, it is demonstrated that H_2O_2 in the system is sufficient to continuously reduce HOBr back to Br^-, thereby ensuring the normal operation of the bromine catalytic cycle."

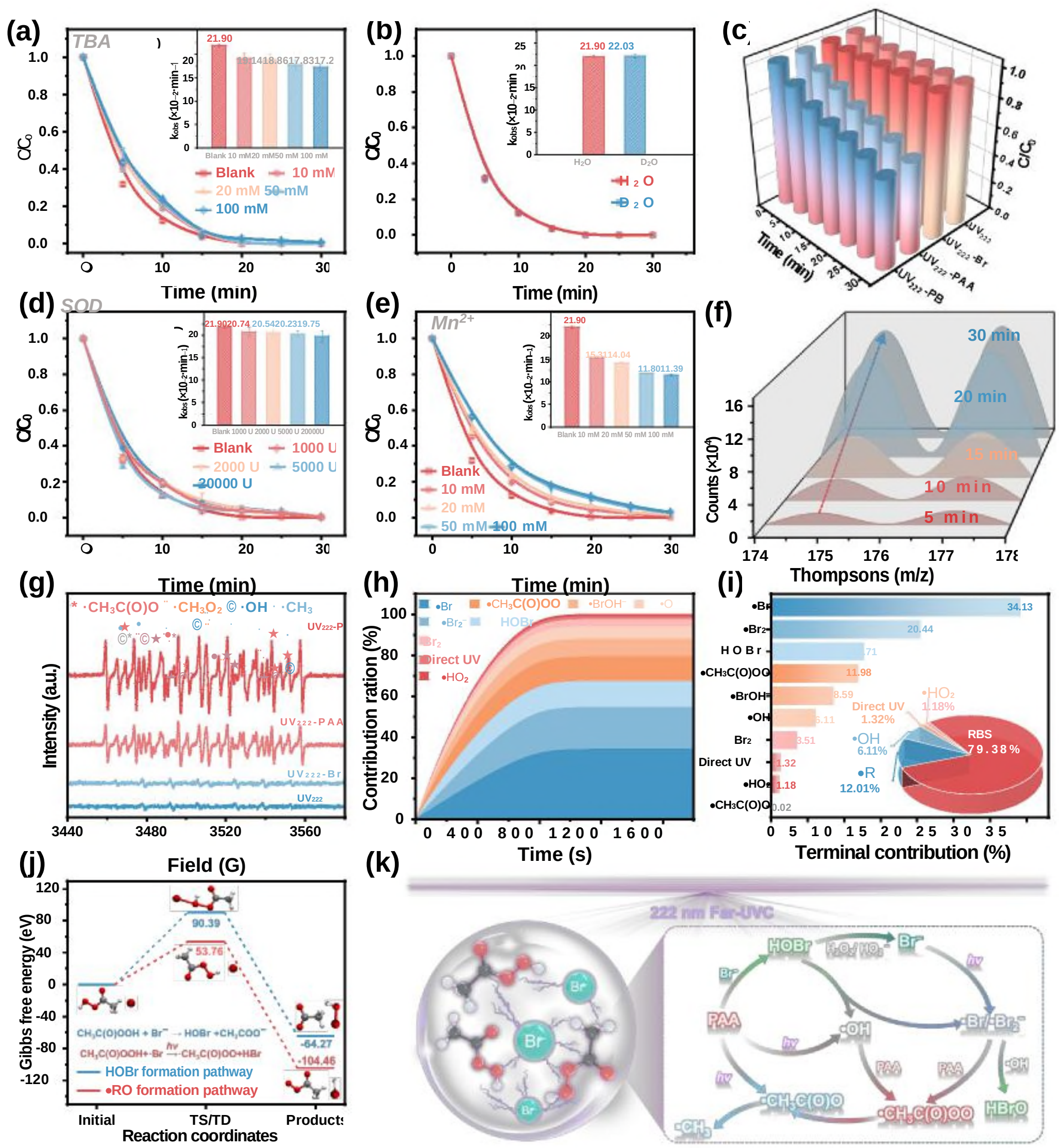

Fig. 2 | Identification of active species and their interaction mechanisms. SDZ degradation curves and k_{obs} values in UV_{222}-PB system at different (a) TBA concentrations and (b) solvents. (c) BA degradation following various UV_{222} treatment procedures. Degradation of SDZ using the UV_{222}-PB process at different (d) SOD and (e) Mn^{2+} concentrations. (f) Concentration of 4-Br-DMPZ at different time points during the UV_{222}-PB process. (g) ESR spectra obtained by using DIPPMPO as spin trapping agents in different processes. In the UV_{222}-PB model, (h) the effect of various active substances on SDZ removal and (i) the terminal contribution. (j) Gibbs free energy change diagram for the reaction between HOBr and $•CH_3C(O)OO$. (k)

Mechanism of synergistic interaction in UV_{222}-PB process.

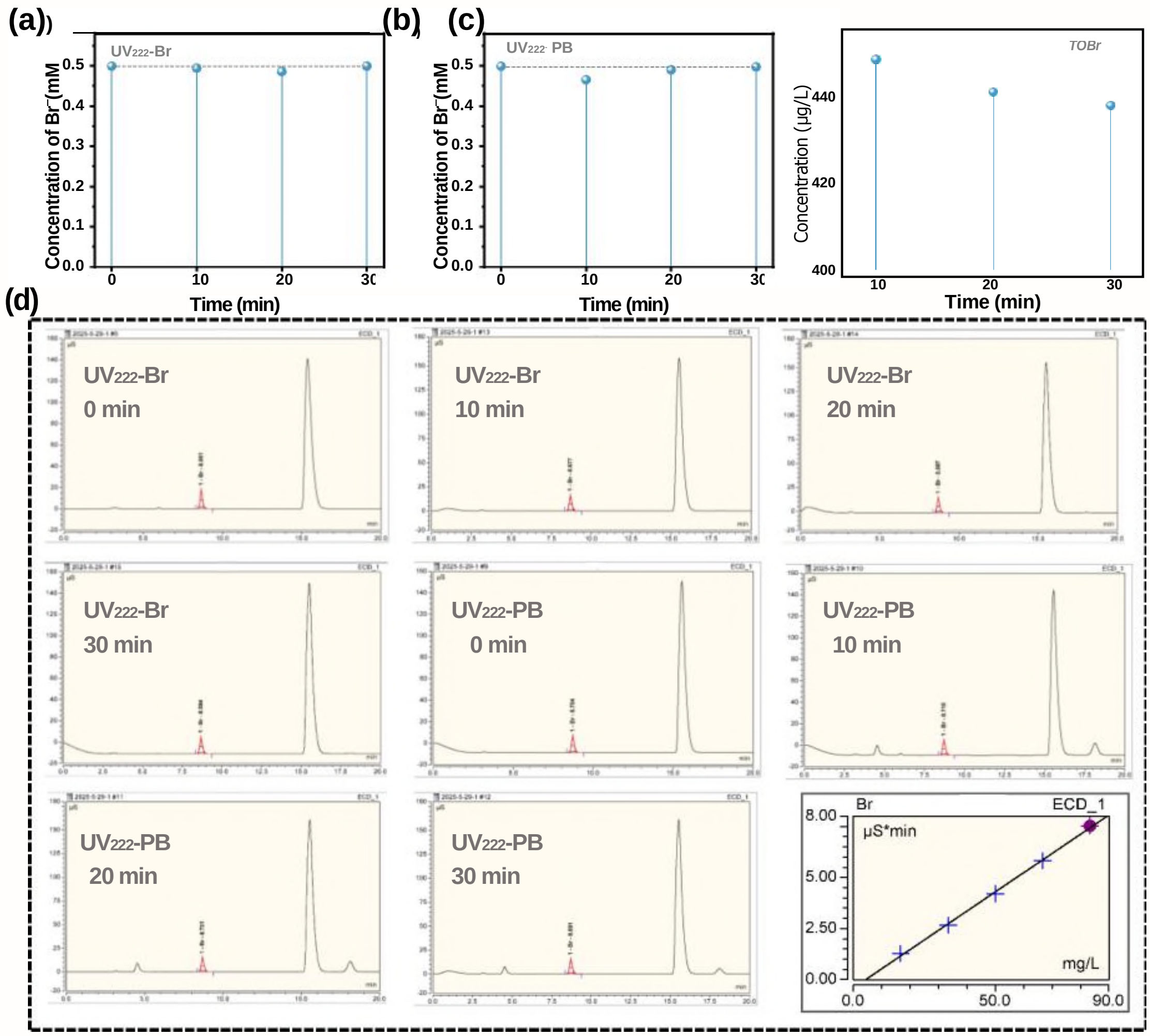

Supplementary Fig. 14 | (a) Concentration changes of Br^- in the UV_{222}-Br and (b) UV_{222}-PB systems. (c) Concentrations of total organic bromine (TOBr) generated during the UV_{222}-PB process at different time points. (d) Ion chromatograms of Br^- in the UV_{222}-Br and UV_{222}-PB systems at different time points, and standard curve of Br^- under IC measurement.

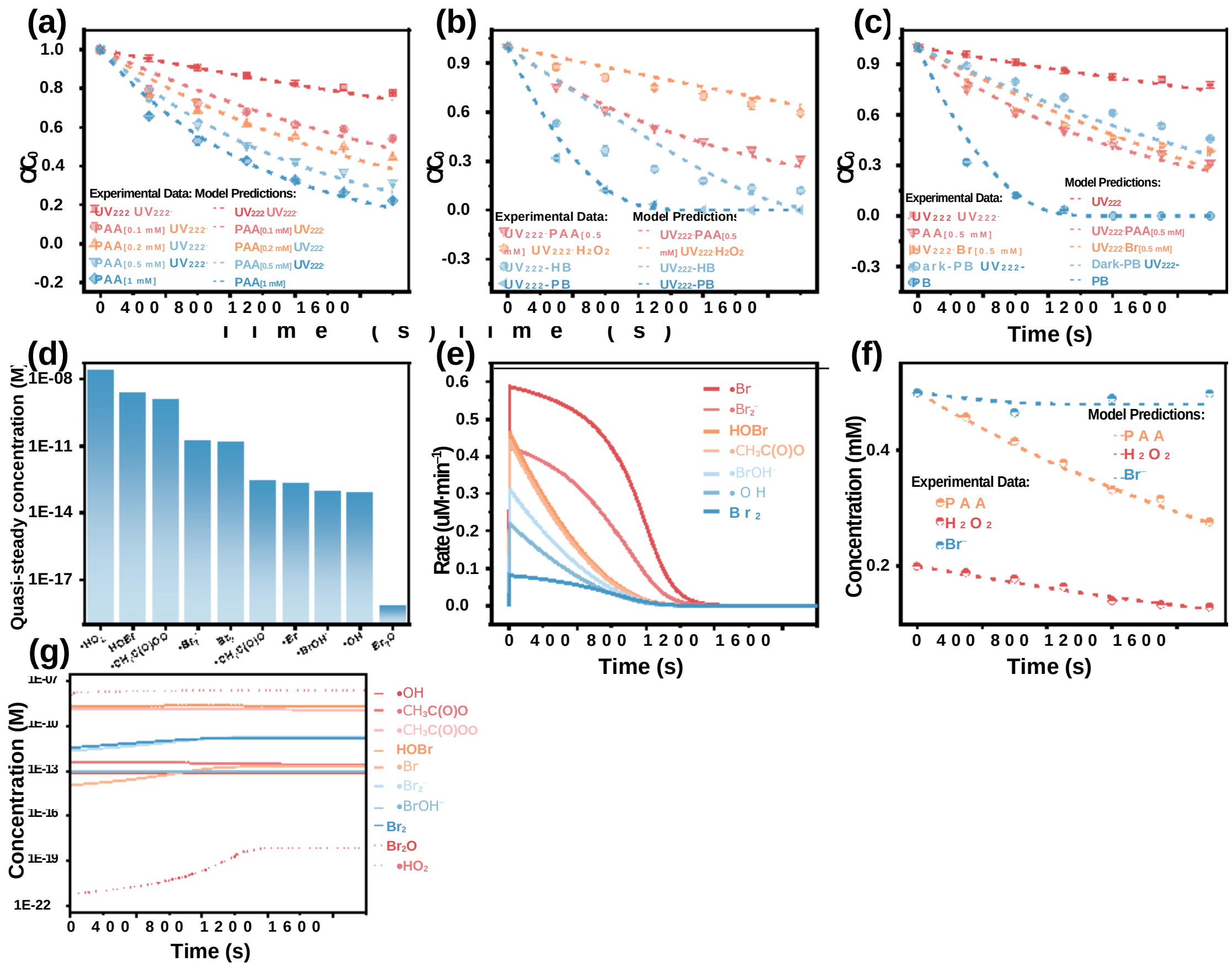

Supplementary Fig. 17 | (a–c) Degradation model curves of the UV_{222}-PB process (symbols represent experimental data, dashed lines represent model predictions). (d) Final steady-state concentrations of different reactive species, (e) reaction rate curves, and (g) species concentration curves in the model. (f) Concentration variations of PAA, H_2O_2, and Br^-. symbols represent experimental data, dashed lines represent model predictions.

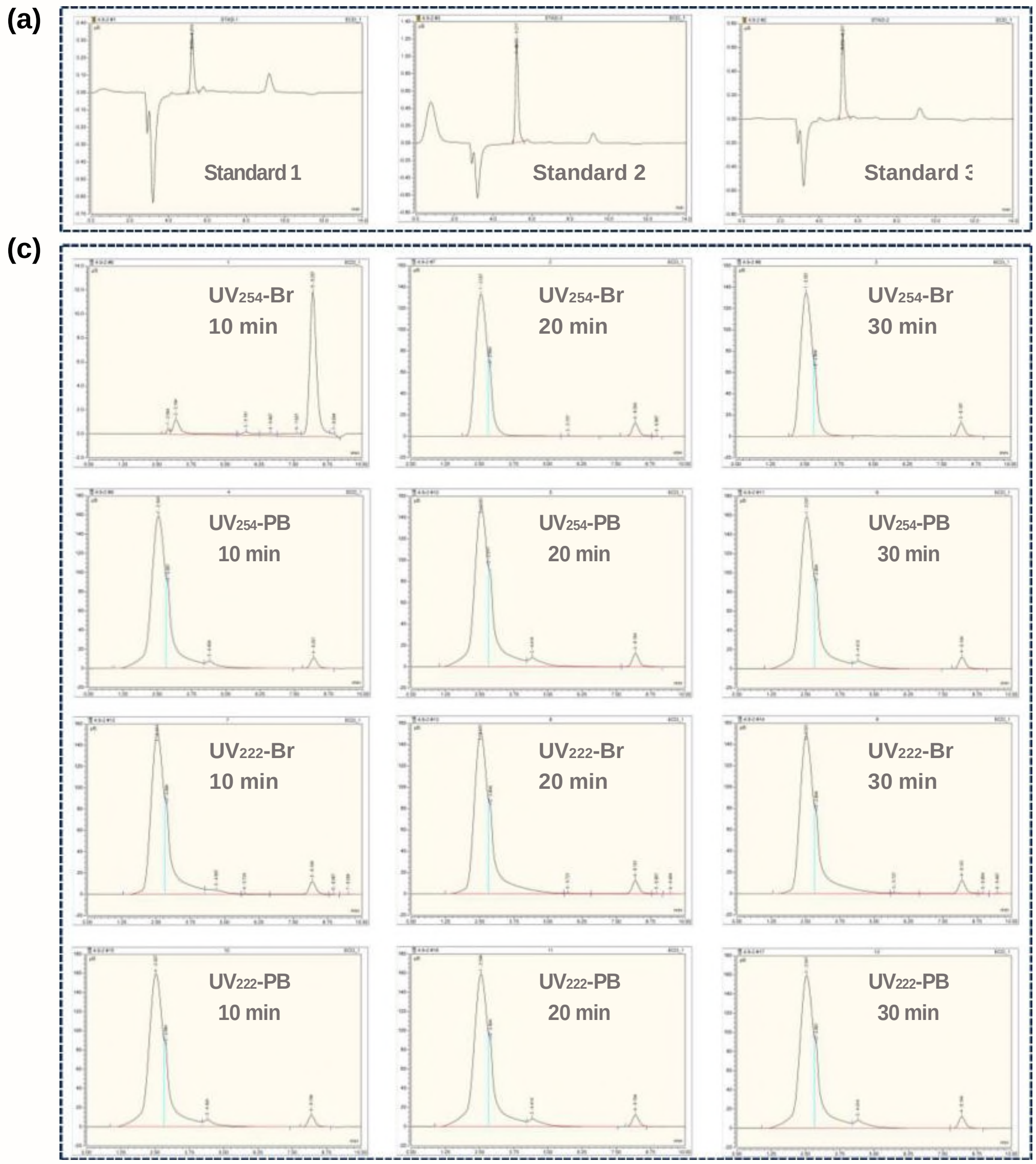

Supplementary Fig. 23 | (a) BrO_3^- standard sample detected by IC. (b) Ion chromatograms of BrO_3^- in the UV_{254}-Br, UV_{254}-PB, UV_{222}-Br, UV_{222}-PB systems at different time points.

Comment 7: In investigating the effect of pH on the UV_{222}-PB system, only three pH values (3, 7, and 11) were tested. It is suggested to add more gradient pH points in the neutral to alkaline range to more accurately identify the optimal pH range of the system and the critical pH value for performance changes, making the conclusion more precise.

Response: Thank you for raising this key question, which has helped make the

logic of our manuscript more rigorous. In the UV_{222}-PB process, SDZ (pK_{a1} = 1.57, pK_{a2} = 6.5), PAA (pK_a = 8.2), and HOBr (pK_a = 8.8) are three important factors affecting process performance. Their dissociation constants determine the speciation of each component under different pH conditions, thereby influencing pollutant degradation efficiency. To address this, we added an experimental point at **pH 6** near the second pK_a of SDZ to more clearly investigate the effect of different SDZ species on degradation behavior. Meanwhile, we added experimental points at **pH 8 and pH 9** on either side of the pKa values of PAA and HOBr to capture the critical transition from their protonated to deprotonated forms. Detailed descriptions are provided in **Lines 584–621**, **Fig. 6a** and **Supplementary Figs. 24, 28** of the revised manuscript. The results show that the UV_{222}-PB system maintains high degradation efficiency across the pH range of 6–9. These findings not only confirm that the UV_{222}-PB system exhibits good environmental adaptability under environmentally relevant neutral to weakly alkaline conditions, but also provide a mechanistic basis for understanding how pH regulates the synergistic interactions among PAA, Br^-, and related species. Once again, we appreciate your meticulous guidance, which has significantly enhanced the environmental relevance and mechanistic clarity of this manuscript.

The related descriptions and images are presented below for your convenience:

Lines 584–621: "The pH of solution is an important parameter that influences pollutant degradation and the production of reactive chemicals. Therefore, this study investigated the effect of the UV_{222}-PB system on the degradation of SDZ under different pH conditions. It is evident that, in the UV_{222} system, SDZ exhibits the best degradation performance under neutral conditions (Supplementary Fig. 24). This is because SDZ, as an amphiprotic compound (pK_{a1} = 1.57, pK_{a2} = 6.5), exists in different forms depending on the pH of the solution. At pH 3, SDZ predominantly exists in its neutral form (SDZ^0). As the pH increases further, the deprotonated form (SDZ^-) gradually replaces SDZ^0 as the dominant species (Supplementary Fig. 25). DFT calculations indicate that compared to SDZ^+ (5.29 eV) and SDZ^- (4.82 eV), SDZ^0 has a smaller HOMO-LUMO energy gap (4.67 eV), thus requiring lower energy for

excitation, which results in a faster reaction rate (Supplementary Fig. 26)[9]. On the other hand, in the UV_{222}-Br system, increasing the Br^- concentration from 0 to 1 mM gradually enhances the degradation efficiency of SDZ. Interestingly, the UV_{222}-Br process shows better degradation performance at pH 7. This phenomenon may be attributed to the reduced generation of •OH under acidic conditions (Supplementary Fig. 27), while alkaline conditions are unfavorable for the formation of HOBr[25]. Moreover, in UV_{222}-PB system, when the Br^- concentration was within 50 μM, the removal efficiency of PB for SDZ correlated positively with pH. When the Br^- concentration surpassed the threshold, an inverse pH–dependent relationship appeared (Fig. 6a and Supplementary Fig. 28). Under different pH values, the differences in SDZ degradation by UV_{222}-PB system are attributed to: (1) low Br^- concentration. PAA oxidation of Br^- results in limited HOBr, and SDZ elimination relies on the reactive species produced by PAA. However, the morphology of PAA (pK_a = 8.2) is influenced by pH. As the solution pH rises, deprotonated PAA (PAA^-) increasingly dominates. The reaction rate constant of PAA^- ($k = 3.25 \times 10^{-5}\ s^{-1}$) activated by far-UVC in alkaline condition was higher than that of PAA ($k = 2.98 \times 10^{-5}\ s^{-1}$) in acidic condition, thus demonstrating superior removal efficiency of SDZ in alkaline environments[18]. (2) High Br^- concentration. The pH of the solution affects the species distribution of SDZ and HOBr (pK_a = 8.8)[64]. Since SDZ^- becomes the predominant species in alkaline conditions, electrostatic repulsion causes a decrease in the degradation rate. Furthermore, the oxidation potential of PAA^- (1.01 V vs. SHE) was weaker than that of PAA (1.75 V vs. SHE)[31]. Therefore, as the pH rose, the amount of HOBr formed by oxidation in system gradually decreased. Meanwhile, under alkaline conditions, HOBr underwent self-decomposition to form BrO^-, which exhibited weaker electrophilic and thus less efficient in attacking the sulfonamide group of SDZ and the amino groups in its aniline ring[23]. Therefore, compared to acidic environments, the UV_{222}-PB system with higher Br^- concentrations in alkaline circumstances exhibited significantly reduced degradation performance toward SDZ."

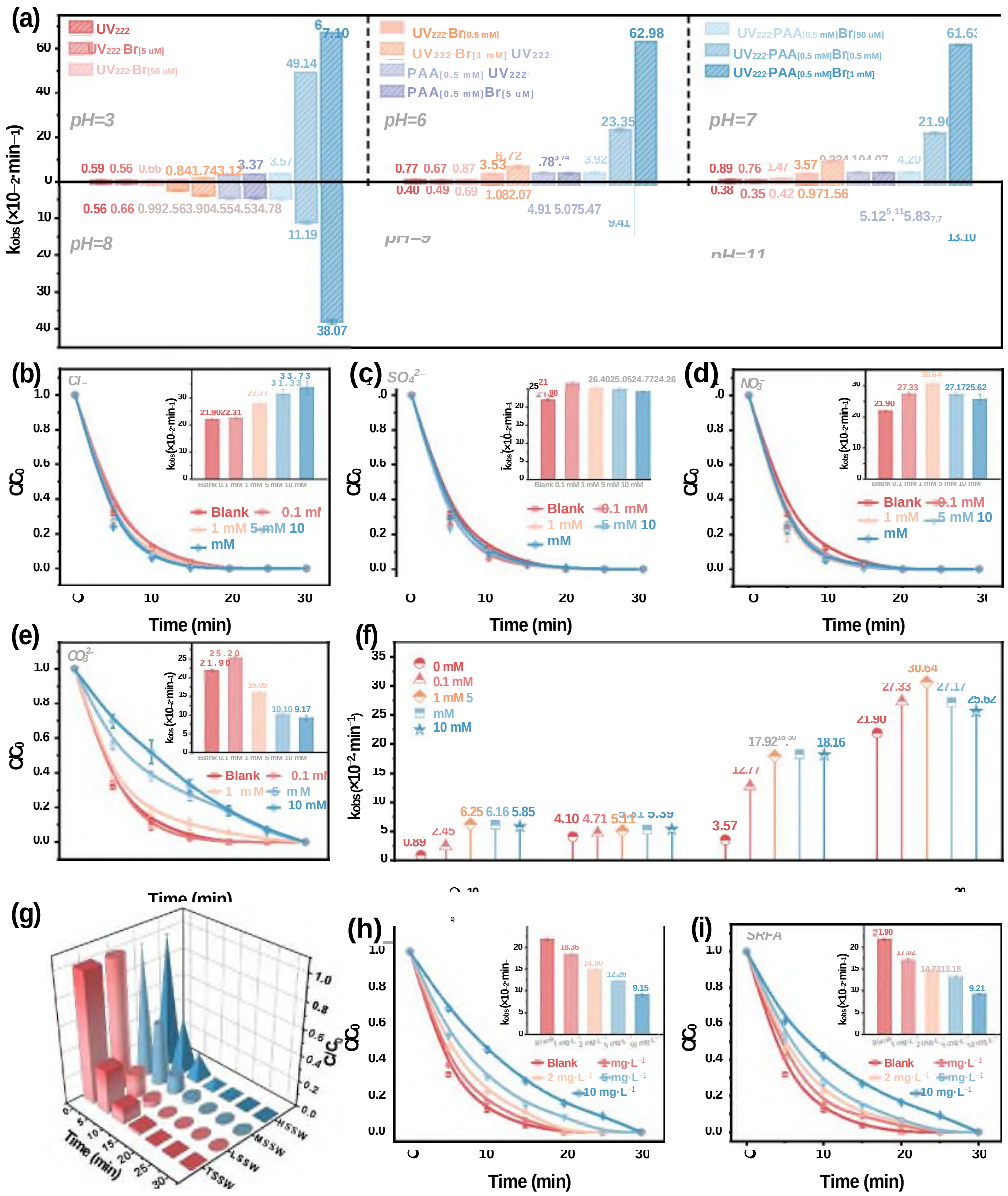

Fig. 6 | The Regulatory Effects and Mechanisms of Water Quality Parameters on SDZ Degradation. (a) k_{obs} values of SDZ in different processes at multiple pH levels. Degradation curves and k_{obs} values of SDZ degraded by UV_{222}-PB in the presence of (b) Cl^-, (c) SO_4^{2-}, (d) NO_3^-, (e) CO_3^{2-}. (f) The effect of coexisting NO_3^- on the degradation of SDZ under UV_{222} using different processes. (g) The performance of UV_{222}-PB in simulated water bodies with different salinities. Degradation curves and k_{obs} values of SDZ degraded by UV_{222}-PB in the presence of (h) SRHA and (i) SRFA.

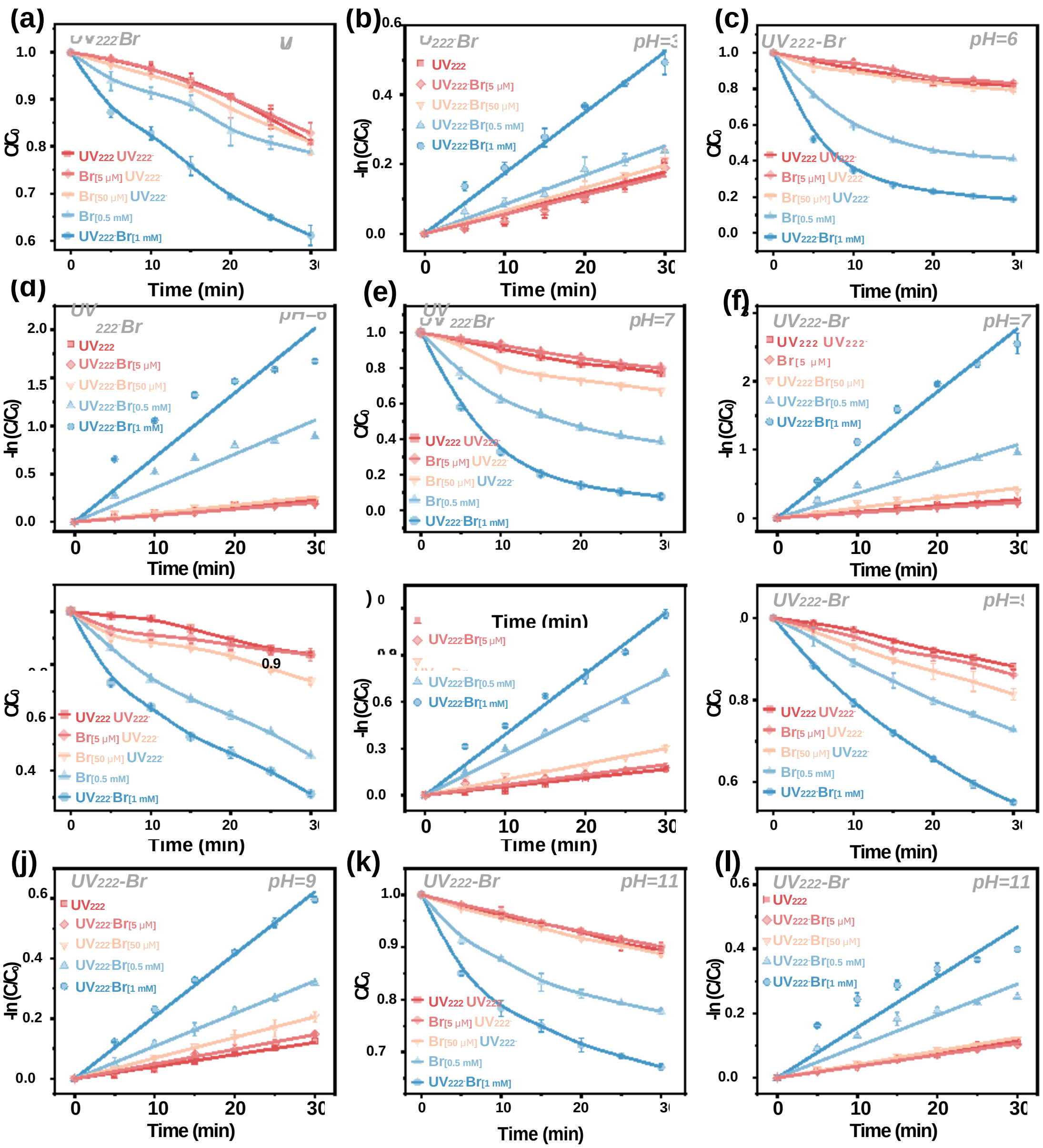

Supplementary Fig. 24 | Degradation curves and pseudo-first-order kinetic curves of SDZ in the UV_{222}-Br system at (a–b) pH 3, (c–d) pH 6, (e–f) pH 7, (g–h) pH 8, (i–j) pH 9, and (k-l) pH 11. Conditions: $[SDZ]_0$=20 uM, $[Br]_0$=0.5 mM, pH = 3.0-11.0.

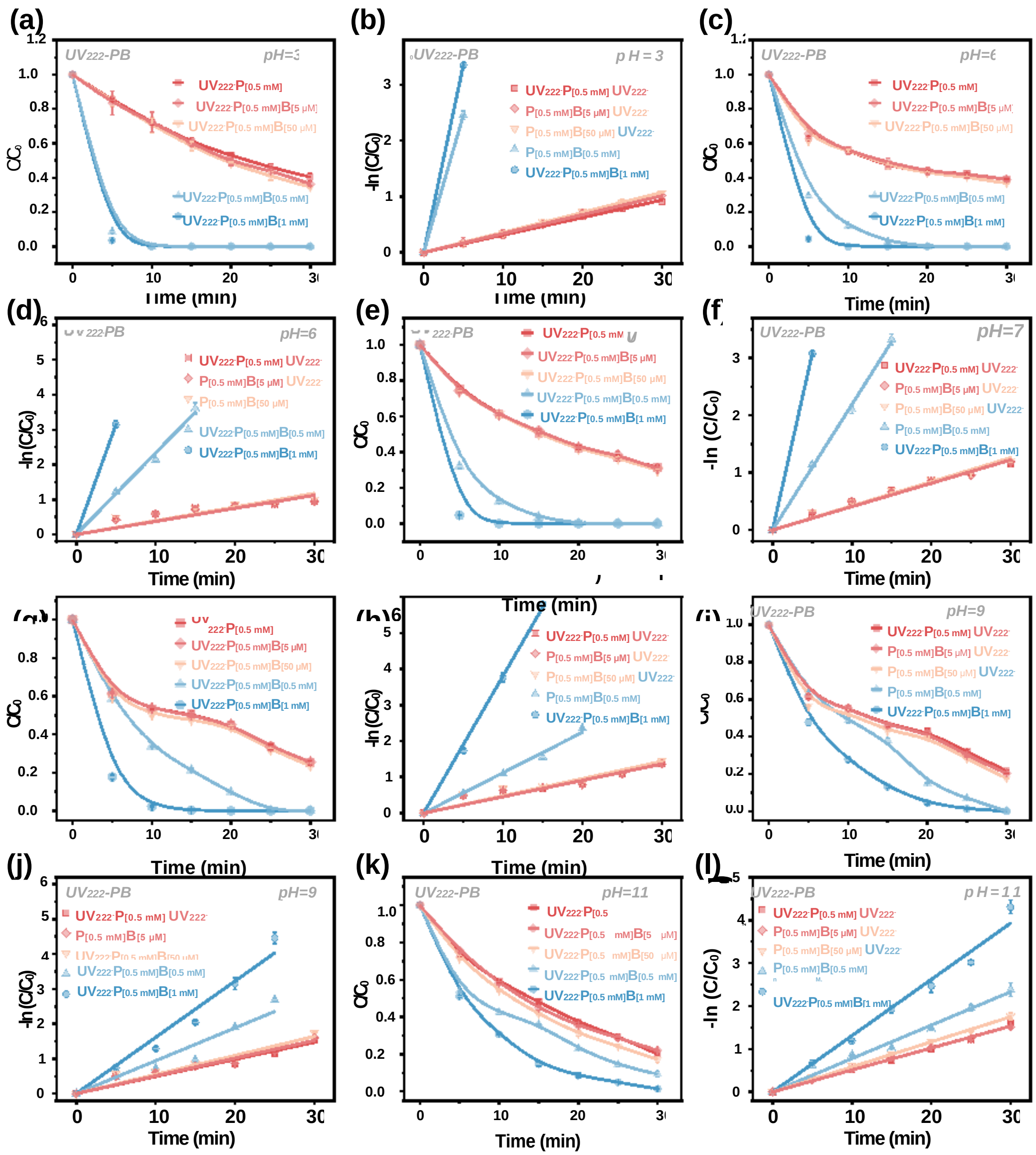

Supplementary Fig. 28 | Degradation curves and pseudo-first-order kinetic curves of SDZ in the UV222-PB system at (a–b) pH 3, (c–d) pH 6, (e–f) pH 7, (g–h) pH 8, (i–j) pH 9, and (k-l) pH 11. Conditions: $[SDZ]_0$=20 uM, $[PAA]_0$=$[Br]_0$=0.5 mM, pH = 3.0-11.0.

Comment 8: When examining the impact of inorganic anions, the authors analyzes the promoting or inhibiting effects of Cl^-, NO_3^-, SO_4^{2-} and CO_3^{2-}, but does not consider the simultaneous presence of multiple anions in real water matrices. It is suggested to

add experiments with mixed anions to better simulate realistic water conditions and evaluate their combined effects on system performance.

Response: We sincerely appreciate your rigorous review and particularly value your suggestion regarding the supplementary experiment on mixed anions. In response to your comments, we designed four mixed-anion systems to simulate water bodies with different salinity levels, namely trace-salinity simulated water (TSSW, 1 mM Cl^-, 0.1 mM SO_4^{2-}, 0.1 mM CO_3^{2-}, 0.1 mM NO_3^-), low-salinity simulated water (LSSW, 1 mM Cl^-, 1 mM SO_4^{2-}, 1 mM CO_3^{2-}, 1 mM NO_3^-), medium-salinity simulated water (MSSW, 10 mM Cl^-, 5 mM SO_4^{2-}, 5 mM CO_3^{2-}, 5 mM NO_3^-), and high-salinity simulated water (HSSW, 0.5 M Cl^-, 10 mM SO_4^{2-}, 10 mM CO_3^{2-}, 10 mM NO_3^-). The selected concentrations cover a broad salinity range from freshwater to seawater. The supplementary results are presented in **Lines 675–690**, **Fig. 6g** and **Supplementary Fig. 30** of the revised manuscript, showing that the UV_{222}-PB process maintains good performance across a wide salinity range. Once again, we thank you for you for your valuable comments, which have further enhanced the environmental relevance of this work.

The related descriptions and images are presented below for your convenience:

Lines 675–690: "Real water matrices often consist of multiple complex ions. Therefore, this study configured trace-salinity simulated water (TSSW), low-salinity simulated water (LSSW), medium-salinity simulated water (MSSW), and high-salinity simulated water (HSSW) to better evaluate the performance of the UV_{222}-PB process in water bodies with different salinity ranges. It can be observed that the UV_{222}-PB process exhibited good degradation performance in all four simulated water matrices, with the pollutant being almost completely degraded within 20 minutes (Fig. 6g and Supplementary Figs. 30a–b). Moreover, the performance of the UV_{222}-PB process was optimal in LSSW. Compared with TSSW, the appropriate concentrations of NO_3^-, SO_4^{2-}, and CO_3^{2-} in LSSW promoted the generation of more reactive species in the system. However, as the salinity further increased, the degradation rate of SDZ gradually decreased, with a k_{obs} value of 0.2093 min^{-1} in HSSW. This may be attributed

to the increase in pH caused by the addition of a higher concentration of CO_3^{2-} as well as the light-shielding effect of NO_3^- (Supplementary Table 15). Overall, the UV_{222}-PB process can operate stably across a wide salinity range from freshwater to seawater, providing strong support for its application in practical high-salinity water bodies."

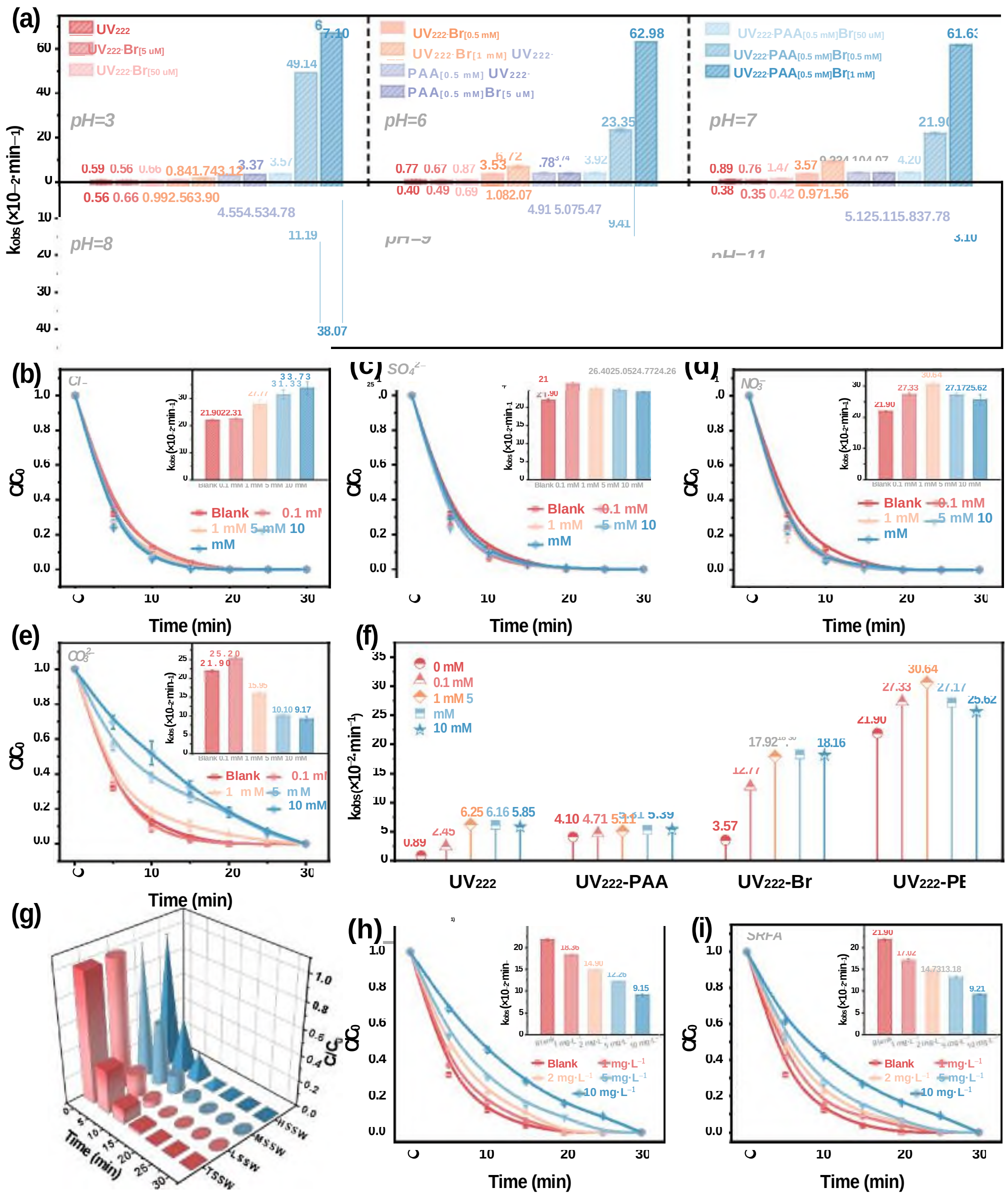

Fig. 6 | The Regulatory Effects and Mechanisms of Water Quality Parameters on SDZ Degradation. (a) k_{obs} values of SDZ in different processes at multiple pH levels. Degradation curves and k_{obs} values of SDZ degraded by UV_{222}-PB in the presence of (b) Cl^-, (c) SO_4^{2-}, (d) NO_3^-, (e) CO_3^{2-}. (f) The effect of coexisting NO_3^- on the

degradation of SDZ under UV_{222} using different processes. (g) The performance of UV_{222}-PB in simulated water bodies with different salinities. Degradation curves and k_{obs} values of SDZ degraded by UV_{222}-PB in the presence of (h) SRHA and (i) SRFA.

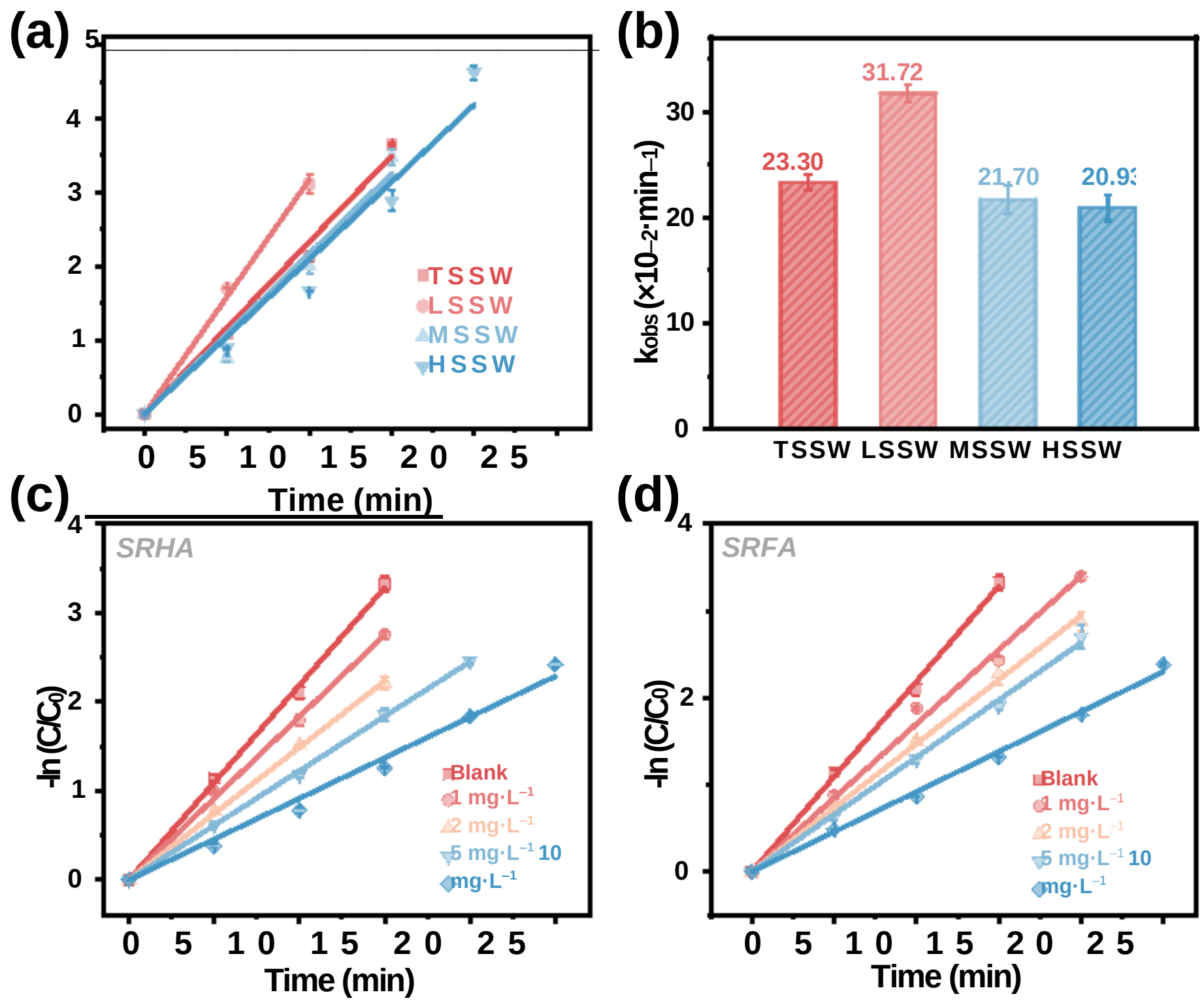

Supplementary Fig. 30 | (a) Pseudo-first-order kinetic fitting curves and (b) k_{obs} values for different simulated salinity water bodies in the UV_{222}-PB system. Pseudo-first-order kinetic fitting curves of SDZ under different concentrations of (c) SRHA and (d) SRFA in the UV_{222}-PB system. Conditions: $[SDZ]_0 = 20$ μM, $[PAA]_0 = [Br]_0 = 0.5$ mM.

Comment 9: In the section "Analysis of pollutant degradation pathways and systemic toxicity assessment", density functional theory (DFT) calculations provide the energy barriers for HOBr generation and organic radical reactions. Were temperature effects considered in these calculations? If not, it is suggested to add relevant discussion or explain why temperature factors were not considered in the experiment.

Response: Thank you for raising this key question, which has helped make our manuscript more rigorous. In the thermodynamic calculations of HOBr generation and

organic radical formation, temperature effects were taken into account. As stated in the **Supplementary Information (Lines 239–242)**, "the Gibbs free energy is calculated at 298.15 K and 1 atm," which is consistent with the actual experimental temperature. Therefore, the energy barriers for HOBr generation and organic radical reactions reported in the manuscript already include the temperature contribution at 298.15 K. We have clarified this point in the Supplementary Information of the revised manuscript.

The related descriptions are presented below for your convenience:

Lines 239–242: "Vibrational frequencies were calculated at the same level to ensure no imaginary frequencies at the minima and saddle points, and the Gibbs free energy was calculated at 298.15 K and 1 atm."

Comment 10: In the toxicity assessment, the zebrafish embryo culture experiment only records hatching status. It does not evaluate teratogenic rate, types of malformations, or post-hatching larval survival. It is suggested to add relevant toxicological indicators to make the toxicity assessment more comprehensive and in-depth.

Response: Thank you for pointing out the deficiencies in our zebrafish embryo toxicity assessment, which has been very helpful in improving the ecological toxicity evaluation of our study. In response, we have extended the zebrafish embryo toxicity assessment by adding additional toxicological endpoints, including malformation rate, types of malformations (e.g., pericardial edema, eye reduction, yolk sac absorption delay, spinal deformity, etc.), and zebrafish survival rate. The detailed results have been added to the revised manuscript (**Lines 521–534** and **Fig. 5d–f**). These supplementary data further confirm that the overall toxicity of the water sample treated by the UV_{222}-PB process is significantly reduced. Once again, we appreciate your guidance, which has made the toxicity assessment more comprehensive and in-depth.

The specific modifications are as follows:

Lines 521–534: "The results of the zebrafish embryo culture experiment showed that, except for the blank control group, all treatment groups exhibited varying degrees of morphological abnormalities. Among them, the SDZ treatment group showed the

highest malformation rate, reaching 100% at 96 hours post-fertilization (hpf). Zebrafish exposed to the original SDZ solution began to show morphological malformations such as pericardial edema (PE), eye deformation (ED), and yolk sac edema (YSE) at 72 hpf (Fig. 5d), indicating that the SDZ solution has high animal toxicity. Zebrafish cultured in solutions treated with the UV_{222}-PAA and UV_{222}-Br processes also exhibited PE developmental abnormalities at 72 hpf. Compared with the original SDZ group, both the survival rate and hatching rate of zebrafish at 96 hpf were improved after treatment with the various UV_{222}-based processes (Figs. 5e–f). Among them, the UV_{222}-PB process exhibited the most significant detoxification effect, with its hatching rate at 96 hpf being only 4.17 % lower than that of the blank control group."

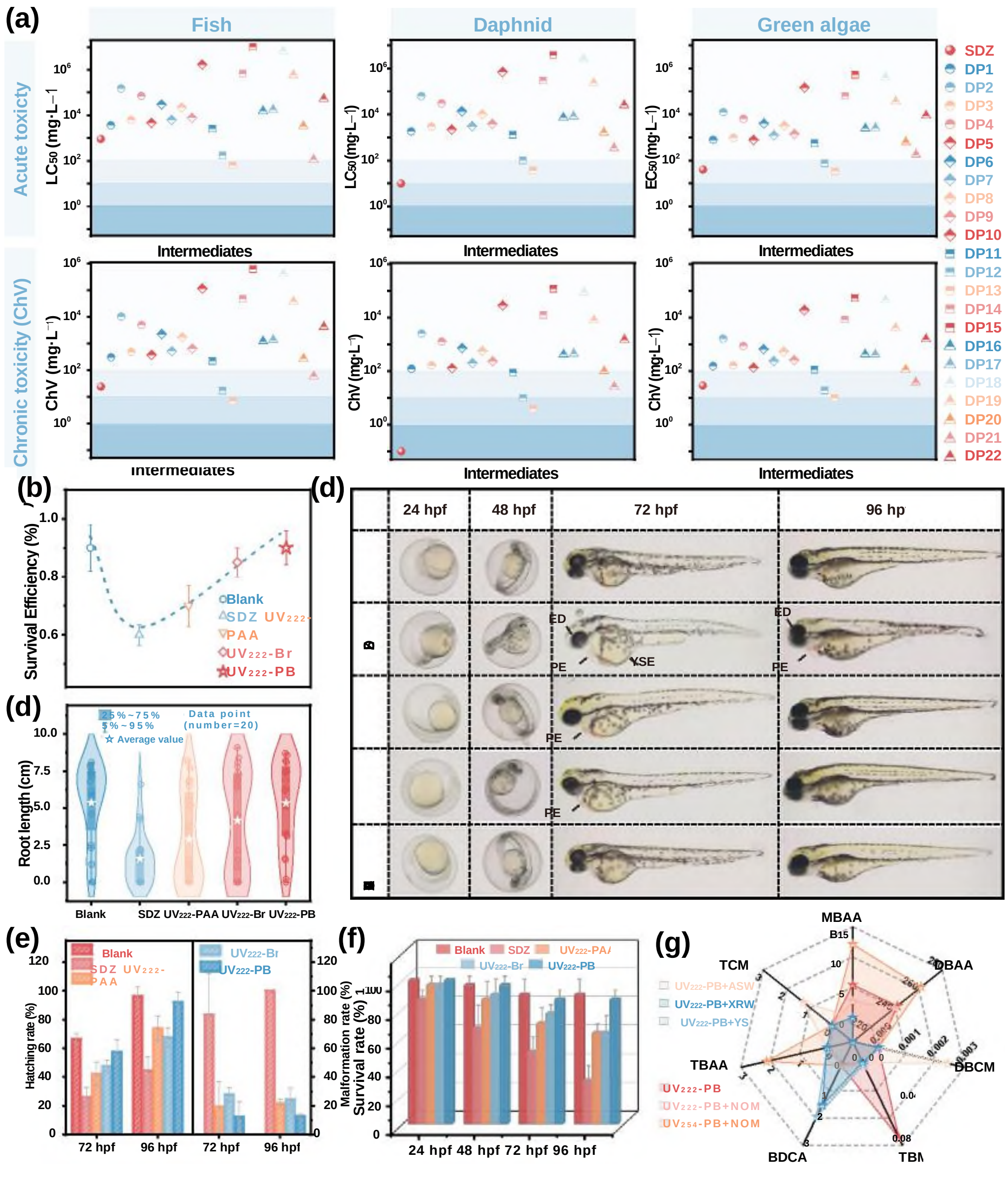

Fig. 5 | Multi-model toxicological effect assessment. (a) Acute and chronic toxicity of SDZ and its intermediates. In different treatment conditions (b) wheat germination rates, (c) violin diagram of wheat bud length distribution and zebrafish embryo (d) development, (e) hatching rates, (f) survival rates. (g) The amount of DBPs produced in different processes.

Comment 11: The manuscript contains several grammatical errors and awkward phrasings (e.g., Line 471:" UV_{222}-PB the survival rates of wheat seeds were 90%, 60%,

70%, 85%, and 90%,"). Additionally, some abbreviations are not defined at their first appearance in the text (e.g., SRHA, SRFA, NOM). A thorough revision of the language and formatting is recommended to ensure clarity and adherence to standard academic writing conventions.

Response: We sincerely thank you for your careful review of the manuscript and your valuable suggestions. In response to your comments, we have thoroughly revised the language throughout the manuscript to improve clarity and readability. Moreover, all abbreviations in the text have been defined at their first appearance. Once again, we are deeply grateful for your patience and guidance. Your suggestions have helped enhance the overall quality of our manuscript, and we have greatly benefited from them.

Responses to Reviewers' Comments

Journal: Nature Communications

Manuscript Number: NCOMMS-26-008494B

Title: Bromide-Mediated Enhancement of Far-UVC/Peracetic Acid Process for Selective Micropollutant Abatement

Author(s): Genying Zeng [a b], Guoyu Li [a], Hou Wang [a], Chen Zhang [a], Qiang Chen [a b], Qinyuan Tang [a b], Tangshan Wu [a b], Li Li [c], Xinjiang Hu [d *], Ran Yin [e f *], Xiaofei Tan [a b *]

General note: Comments from reviewers are presented in black *italic* style. Responses to each comment are in blue and regular style. "Revised text as it appears in the manuscript" is indented and in red.

Reviewer #1

General Comments: *The authors are appreciated for their efforts in responding to the previous comments and making corresponding revisions. Nevertheless, there remain several important issues, particularly those related to the core reaction mechanisms, that would benefit from further clarification.*

Response: We are deeply grateful to you for re-reviewing our manuscript and for your recognition of our previous revisions. Your affirmation has greatly encouraged us. In response to the key concerns you have raised regarding the core reaction mechanisms and engineering applicability, we have conducted systematic supplementary investigations in the revised manuscript, focusing on the following four aspects: (1) Through kinetic model-based pathway blocking analysis and sensitivity analysis, the primary source of •Br in the UV_{222}-PB system was identified, and its chain-cycling mechanism was elucidated (**Lines 443–462**). (2) Performed control experiments under dark conditions with PAA and Br^- to evaluate the contribution of HOBr to SDZ degradation (**Lines 152–160**). (3) Via externally added H_2O_2 and catalase scavenging experiments, we systematically revealed the multifaceted role of coexisting H_2O_2 in the system (**Lines 166–199**). (4) Supplemented the LCA assessment based on continuous-flow experimental data and future clean energy scenario analyses, further validating the environmental sustainability of the process (**Lines 882–933**). Detailed revisions are provided in our point-by-point responses below. Your professional insights have been invaluable in improving the quality of this work, and we sincerely hope these revisions meet your expectations. Once again, we extend our deepest gratitude to you. The depth of your expertise and the rigor of your scholarship will continue to inspire our future research endeavors.

Comment 1: The authors indicated that •BrCl reacts rapidly with Br^- to generate •Br, so •Br is the main reactive species. However, as shown in Figure 7e, the contribution of •BrCl to SDZ removal is only slightly lower than that of •Br. In Supplementary Table 18, the authors determined the second-order rate constants of SDZ with $•Br_2^-$ and $•BrCl^-$ as 1.53×10^8 $M^{-1}·s^{-1}$ and 1.78×10^8 $M^{-1}·s^{-1}$, respectively. The second-order rate constant for the reaction of $•BrCl^-$ with Br^- to generate $•Br_2^-$ is 8.00×10^9 $M^{-1}·s^{-1}$. Moreover, in the reaction, the concentration of Br^- is 25 times that of SDZ, so $•BrCl^-$ should be rapidly scavenged. Then what is the reason for the high contribution proportion of $•BrCl^-$ in Figure 7e?

Response: Thank you very much for raising this important question, which is crucial for understanding the contributions of various species under high Cl^- concentrations. Regarding your point that $•BrCl^-$ can rapidly react with Br^- to generate $•Br_2^-$ (Reaction 63: $•BrCl^- + Br^- \rightarrow •Br_2^- + Cl^-$, k = 8.00×10^9 $M^{-1}·s^{-1}$), and that the concentration of Br^- in the reaction system is significantly higher than that of SDZ (approximately 25-fold), it is reasonable to expect that $•BrCl^-$ would be rapidly consumed. This argument is valid when considering this single reaction pathway in isolation. However, terminal contribution reflects the cumulative oxidative degradation flux of a given reactive species over the entire reaction time window. Therefore, the cumulative degradation contribution of $•BrCl^-$ is governed by the balance between its generation rate and consumption rate; the rate constant of a single consumption pathway alone cannot determine its final contribution.

In the high-chloride system, the formation and consumption of $•BrCl^-$ are in a state of dynamic coupling, and its steady-state maintenance can be elucidated from both the Br^- and Cl^- perspectives. With respect to Br^-, it participates in both the formation and consumption pathways of $•BrCl^-$. In the formation direction, •Cl (Reaction 56: $•Cl + Br^- \rightarrow •BrCl^-$, k = 1.20×10^{10} $M^{-1}·s^{-1}$) and $•Cl_2^-$ (Reaction 58: $•Cl_2^- + Br^- \rightarrow •BrCl^- + Cl^-$, k = 4.00×10^9 $M^{-1}·s^{-1}$) can rapidly react with Br^- to generate $•BrCl^-$, with their reverse reaction rates (Reaction 57: $•BrCl^- \rightarrow •Cl + Br^-$, k = 2.00×10^3 s^{-1}; Reaction 59: $•BrCl^- + Cl^- \rightarrow •Cl_2^- + Br^-$, k = 1.10×10^2 $M^{-1}·s^{-1}$) being substantially lower than the forward rates. In the consumption direction, $•BrCl^-$ further reacts with Br^- to

produce $\bullet Br_2^-$ (Reaction 63: $\bullet BrCl^- + Br^- \rightarrow \bullet Br_2^- + Cl^-$, k = 8.00 × 10^9 $M^{-1}\cdot s^{-1}$). Thus, Br^- serves not only as a key reactant in the formation pathway of $\bullet BrCl^-$ but also as an indispensable participant in its consumption pathway; both pathways are dependent on the Br^- concentration in the system. With respect to Cl^-, it provides a compensatory regeneration route for $\bullet BrCl^-$ through the reverse reaction. Once $\bullet BrCl^-$ is converted to $\bullet Br_2^-$ by Br^- (Reaction 63: $\bullet BrCl^- + Br^- \rightarrow \bullet Br_2^- + Cl^-$, k = 8.00 × 10^9 $M^{-1}\cdot s^{-1}$), the high concentration of Cl^- can regenerate $\bullet BrCl^-$ via Reaction 62 ($\bullet Br_2^- + Cl^- \rightarrow \bullet BrCl^- + Br^-$, k = 4.30 × 10^6 $M^{-1}\cdot s^{-1}$). Although the rate constant of this reaction is approximately three orders of magnitude lower than that of Reaction 63, the concentration advantage of Cl^- under high-chloride conditions effectively compensates for the kinetic deficiency, rendering this reverse transformation pathway non-negligible. Overall, in the UV_{222}-PBC system, Br^- and Cl^- synergistically sustain the dynamic interconversion cycle between $\bullet BrCl^-$ and $\bullet Br_2^-$, ensuring the continuous supply of $\bullet BrCl^-$ throughout the reaction time window.

Meanwhile, the UV_{222}-PBC kinetic model predicts a steady-state concentration of $\bullet BrCl^-$ at 2.97 × 10^{-11} M, approximately 0.58 times that of $\bullet Br_2^-$ (5.19 × 10^{-11} M). Although $\bullet Br_2^-$ has a concentration advantage, the second-order rate constant of $\bullet BrCl^-$ with SDZ (1.78 × 10^8 $M^{-1}\cdot s^{-1}$) is slightly higher than that of $\bullet Br_2^-$ (1.53 × 10^8 $M^{-1}\cdot s^{-1}$). Therefore, $\bullet BrCl^-$ occupies a significant position in the cumulative degradation contribution, second only to $\bullet Br_2^-$. To facilitate a better understanding of the mechanisms in the UV_{222}-PBC system, we have added corresponding descriptions in **Lines 802–829** and **Supplementary Fig. 32** of the revised manuscript. Thank you once again for raising this important point, which has been invaluable in refining the mechanistic interpretation of this study under high chloride conditions.

The related descriptions and images are presented below for your convenience:

Lines 802–829: "Furthermore, to further elucidate the attribution and interaction of dominant reactive species in the UV_{222}-PB process under high chloride concentration, we introduced 0.5 M Cl^- into the original kinetic model and constructed the UV_{222}-PBC model to better simulate seawater and seawater-affected high-salinity water bodies. Remarkably, the terminal contribution analysis of the UV_{222}-PBC model revealed that

RBS (66.02%) contributed far more to SDZ degradation than RCS (1.55%), with $•Br_2^-$ becoming the dominant reactive species, contributing as much as 48.08% (Figs. 7e–f and Supplementary Figs. 32a–e). This phenomenon is attributed to the rapid conversion of RCS to RBS under high Cl^- concentration. Specifically, •Cl and $•Cl_2^-$ generated in the system can rapidly react with Br^- to form $•BrCl^-$, resulting in low steady-state concentrations of •Cl and $•Cl_2^-$ at 5.57×10^{-15} M and 3.83×10^{-14} M, respectively. Moreover, although HOCl is present in high abundance, its second-order rate constant with SDZ is relatively low. Consequently, the contribution of RCS to SDZ degradation is rather limited. Model predictions indicate that $•Br_2^-$ and $•BrCl^-$ are the two major contributing bromine-related species, with steady-state concentrations of 5.19×10^{-11} M and 2.97×10^{-11} M, respectively. This pattern can be explained by the fact that RCS promotes the generation of $•BrCl^-$, while $•BrCl^-$ can rapidly react with Br^- to form $•Br_2^-$ ($k = 8.00 \times 10^9$ $M^{-1}·s^{-1}$), leading to an approximately 181% increase in the steady-state concentration of $•Br_2^-$ in the UV_{222}-PBC model compared with the UV_{222}-PB model. Although the steady-state concentration of $•BrCl^-$ is only 0.59 times that of $•Br_2^-$, its second-order rate constant with SDZ (1.78×10^8 $M^{-1}·s^{-1}$) is slightly higher than that of

$•Br_2^-$ (1.53×10^8 $M^{-1}·s^{-1}$), making $•BrCl^-$ an important contributor to SDZ degradation. Meanwhile, the generation of •Br is significantly inhibited. The relatively low H+ concentration in the neutral environment limits the conversion of $•BrOH^-$ to •Br, and the high concentration of Cl^- also consumes •Br, jointly leading to an approximately 5.6-fold decrease in the steady-state concentration of •Br. Therefore, multiple factors collectively result in $•Br_2^-$ far exceeding •Br in its contribution to SDZ degradation, making it the dominant reactive bromine species in the UV_{222}-PBC system.”

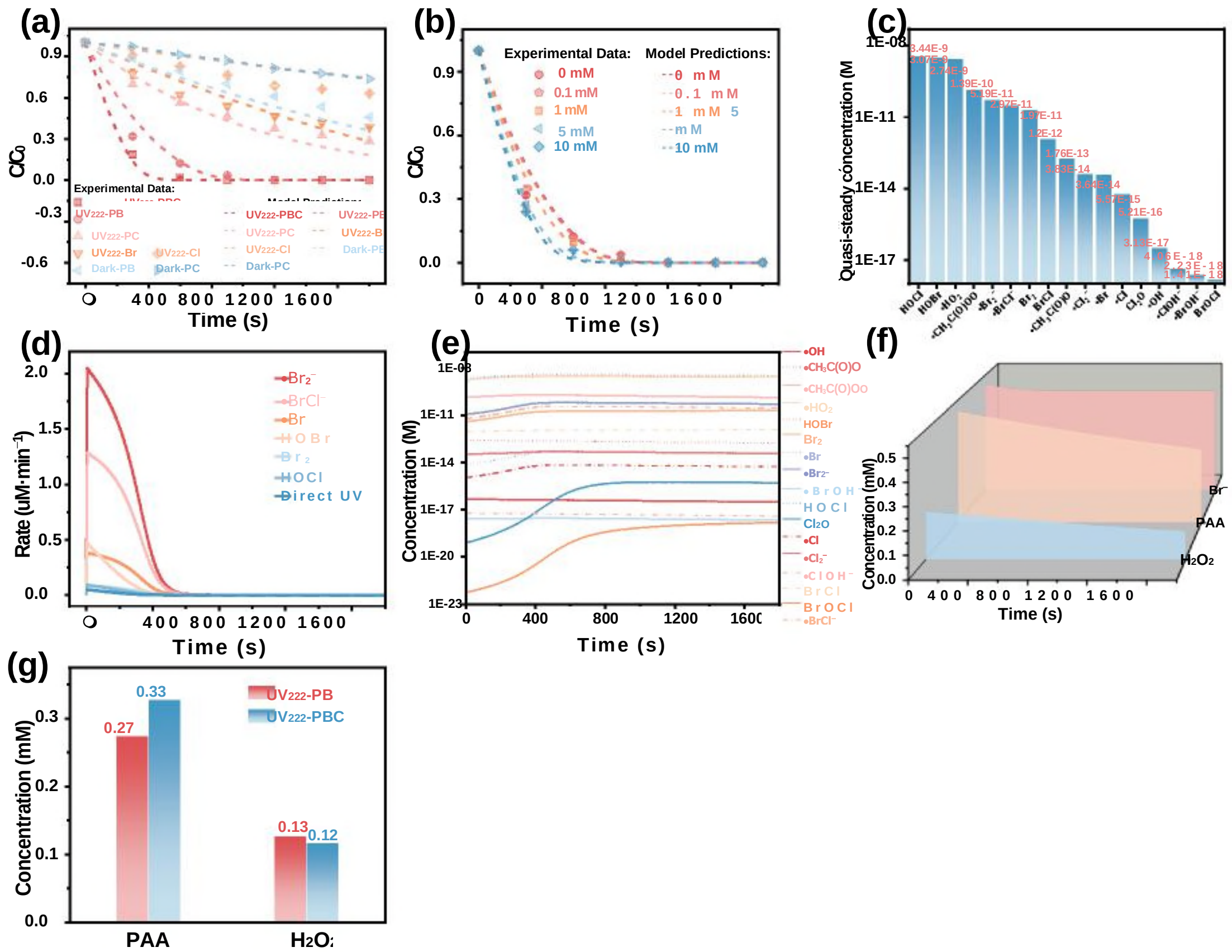

Supplementary Fig. 32 | (a) Degradation model curves for different systems in the UV_{222}-PBC process. (b) Degradation simulation curves of UV_{222}-PB at different Cl^- concentrations. (c) Final steady-state concentrations of different reactive species, (d) reaction rate curves, and (e) species concentration curves in the model. (f) Predicted curves and (g) residual concentrations of PAA, H_2O_2 and Br^-.

Comment 2: The degradation rate of SDZ by UV_{222}-PB is much higher than that by UV_{222}-Br^-, and bromine radicals are the main factor for degradation. Therefore, is the main source of bromine radicals the photolysis of HBrO by UV_{222}? What is the steady-state concentration of HBrO in the reaction system? Could you supplement the degradation experiment of SDZ by UV_{222}/HBrO to better understand the reaction mechanism?

Response: Thank you for raising this key question regarding the source of bromine radicals. It provides important guidance for our further clarification of the

mechanisms underlying bromine radical chain reactions. Based on the kinetic model developed, the study predicted the steady-state concentrations of the various reactive species in the UV_{222}-PB system. Although HOBr had the highest steady-state concentration among the reactive bromine species, its concentration was still only 2.36×10^{-9} M. Furthermore, we fully agree with your suggestion to conduct supplementary UV_{222}/HOBr experiments, as this would indeed help us further clarify the source of •Br. However, HOBr itself is highly unstable and cannot persist stably in solution. Moreover, its preparation and purification inevitably introduce coexisting Br^-, Br_2, or other substances, which would interfere with the independent assessment of the photolytic contribution of HOBr. Therefore, we performed pathway deactivation simulations and local sensitivity analyses within the kinetic model to differentiate the primary generation sources of •Br. Specifically, we set the rate constants of Br^- photolysis, HOBr photolysis, and $•BrOH^-$ protonation–dehydration pathways to zero, respectively, for the deactivation simulations. The results showed that when the Br^- photolysis and

$•BrOH^-$ protonation–dehydration pathways were individually blocked, the simulated SDZ degradation curves deviated to different extents; however, when the HOBr photolysis pathway was blocked, the fitted curve only changed marginally. The local sensitivity analysis further confirmed that the sensitivity coefficients of the Br^- photolysis channel and the $•BrOH^-$ protonation pathway were substantially higher than that of the HOBr photolysis channel. Collectively, these results demonstrate that in the UV_{222}-PB process, the direct photolysis of Br^- is the primary initial source of •Br, while the protonation of $•BrOH^-$ serves as an important synergistic pathway for sustaining the chain cycle. Relevant discussions have been added to the manuscript (**Lines 443– 462**, **Text S7** and **Supplementary Fig. 17**). We would like to once again express our sincere gratitude for your thorough review of this manuscript, which has provided us with valuable guidance for further refining our research on this mechanism.

The related descriptions and images are presented below for your convenience:

Lines 443–462: "To elucidate the primary source of •Br generation in the system, the study conducted a simulated blocking analysis by setting the rate constants for the photolysis of Br^- and HOBr, as well as the protonation and dehydration pathway of

•$BrOH^-$, to zero in the reaction network (Supplementary Fig. 17g). The results showed that when the HOBr photolysis pathway was deactivated, the simulated SDZ degradation curve exhibited only a minor change. In contrast, when the Br^- photolysis pathway was blocked, the fitted curve deviated significantly, indicating that the direct photolysis of Br^- is the main initial source of •Br. Furthermore, deactivation of the •$BrOH^-$ protonation–dehydration pathway also resulted in a noticeable deviation of the degradation curve from the original fit, suggesting that •$BrOH^-$ serves as an important regeneration channel for •Br during the chain reaction. To verify the robustness of the model predictions, a local sensitivity analysis was further conducted by perturbing the rate constants of each reaction channel by ±10% relative to the original simulation curve (Text S7, Supplementary Figs. 17h–k). The results clearly showed that the Br^- photolysis channel exhibited the highest sensitivity coefficient, being 1.63 and 4.48 times higher than those of the •$BrOH^-$ and HOBr channels, respectively. Even minor fluctuations in its rate constant led to significant changes in the degradation curve, further confirming that Br^- photolysis is the critical initiation step of the bromine radical chain reaction, while •$BrOH^-$ protonation serves as an important synergistic pathway for sustaining the chain cycle."

Text S7. Sensitivity Analysis

Based on the original simulation curve, the rate constant of each reaction pathway was individually increased and decreased by 10%, and the resulting C/C_0 degradation curves of SDZ were recalculated. The difference between the two perturbed curves was then compared, and the sensitivity coefficient for each reaction was calculated using the central difference method:

$$\text{Sensitivity} = \frac{C/C_0\,(+10\%) - C/C_0(-10\%)}{[\ln(1.1) - \ln(0.9)]}$$

The maximum absolute value of the sensitivity over the entire reaction time was taken as the bar height, representing the maximum possible influence of each reaction pathway during the reaction process. A higher bar indicates that the SDZ degradation result is more sensitive to the rate of that reaction, whereas a lower bar suggests a weaker impact of that reaction on the current simulation results.

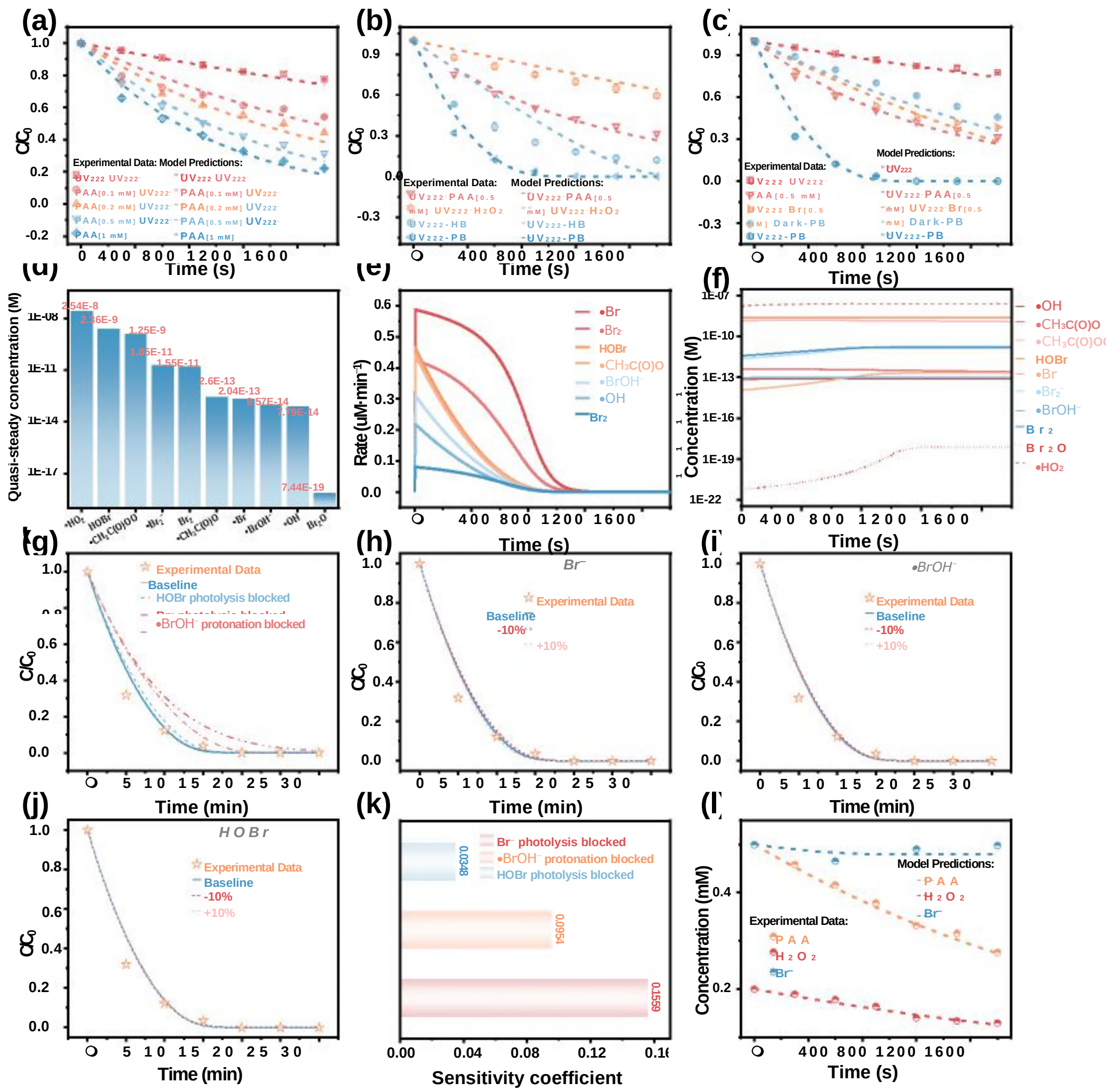

Supplementary Fig. 17 | (a–c) Degradation model curves of the UV_{222}-PB process. (d) Final steady-state concentrations of different reactive species, (e) reaction rate curves, and (f) species concentration curves in the model. (g) Simulated curves of SDZ degradation under different pathway deactivation conditions in the UV_{222}-PB system. (h–j) Sensitivity curves for SDZ degradation via different reaction pathways in the UV_{222}-PB system, and (k) sensitivity coefficients. (l) Concentration variations of PAA, H_2O_2, and Br^-. symbols represent experimental data, dashed lines represent model predictions.

Comment 3: How long does it take for PAA + Br^- to generate HBrO? Is it necessary to conduct a dark reaction before UV_{222} irradiation?

Response: Thank you for this insightful question. According to previous reports,

the rate constant for the reaction between PAA and Br^- is 0.24 $M^{-1}\cdot s^{-1}$, when the two are mixed, HOBr begins to form gradually. In fact, in our supplementary Dark-PB experiment, SDZ degradation was already observable at 30 s after the reaction started and proceeded continuously thereafter, confirming the rapid formation of HOBr and its oxidative capacity. Therefore, this study did not incorporate a dark reaction stage prior to UV_{222} irradiation; instead, the contribution of the dark reaction pathway was isolated and quantified through a comparative analysis of the Dark-PB and UV_{222}-PB experiments. Notably, the k_{obs} value for Dark-PB (0.0252 min^{-1}) is only 0.12 times that of UV_{222}-PB (0.2190 min^{-1}), indicating that far-ultraviolet light is a key factor in the efficient degradation of pollutants. Detailed descriptions and the relevant figure can be found in **Lines 152–155** and **Supplementary Fig. 1** of the manuscript. Thank you again for your attention to the experimental details and your valuable suggestions.

The related descriptions and images are presented below for your convenience:

Lines 152–155: "Notably, the Dark-PB system under dark conditions achieved 54.27% removal of SDZ within 30 minutes (Supplementary Fig. 1), which can be attributed to the reaction between PAA and Br^- to generate HOBr (k = 0.24 $M^{-1}\cdot s^{-1}$), leading to the oxidative degradation of SDZ[24]."

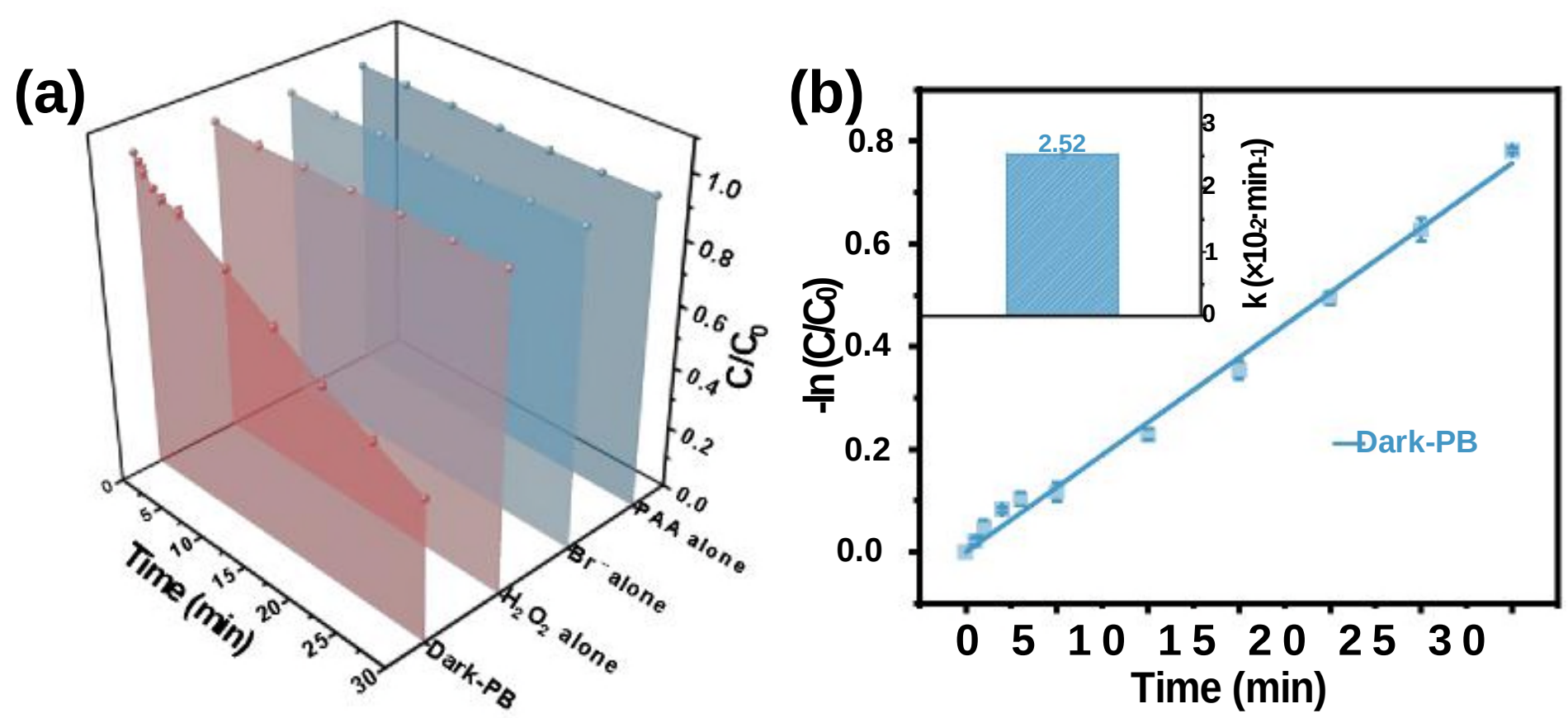

Supplementary Fig. 1 | (a) Degradation of SDZ in different systems under dark conditions. (b) Pseudo-first-order kinetic fitting curves and k_{obs} values for SDZ degradation in the Dark-PB system. $[SDZ]_0$ = 20 μM, pH=7.0, $[PAA]_0$ = $[Br]_0$ = 0.5 mM, $[H_2O_2]_0$= 0.2 mM.

Comment 4: Does the H_2O_2 content in PAA affect the performance of UV_{222}-PB? HBrO can be reduced by H_2O_2, and the photolysis of H_2O_2 contributes little to SDZ degradation.

Response: Thank you very much for your insightful comments, which are crucial for clarifying the role of coexisting H_2O_2 in PAA solutions within the UV_{222}-PB system. In response to your valuable feedback, we have supplemented our study with an investigation into the effects of the UV_{222}-PB system on SDZ degradation in the presence of exogenous H_2O_2 at different concentrations and catalase (CAT). Specifically, when exogenous H_2O_2 at different concentrations was introduced into the UV_{222}-PB system, the degradation rate of SDZ showed a gradual decrease with increasing H_2O_2 concentration due to the light-shielding and radical quenching effects caused by high concentrations of H_2O_2. Furthermore, we added CAT to the system to remove coexisting H_2O_2. Under dark reaction conditions, the degradation rate of the Dark-PB with CAT system was 4.30 times that of the Dark-PB system. This confirms that HOBr can indeed be reduced by H_2O_2, when CAT removes H_2O_2, the reduction pathway of HOBr is blocked, thereby unleashing the oxidative potential of HOBr. In the UV_{222}-PB with CAT system, the decomposition of H_2O_2 leads to an increase in the concentration of dissolved oxygen in the water. Under UV_{222} irradiation, O_2 can be converted into reactive oxygen species such as •OH through chain reactions, thereby enhancing the oxidative degradation of pollutants. Overall, although H_2O_2 may cause a photoblocking effect in the system, its reduction of substances such as HOBr remains a crucial component of the bromine cycle in the UV_{222}-PB system. Relevant experimental data can be found in **Lines 166–199** of the manuscript and **Supplementary Fig. 3**. Once again, we sincerely thank you for your valuable suggestions, which have helped us further refine the mechanistic explanation of this study.

The related descriptions and images are presented below for your convenience:

Lines 166–199: "Given the inevitable presence of H_2O_2 in PAA solutions, this study evaluated the potential impact of H_2O_2 on the UV_{222}-PB system. Based on previous reports and experimental measurements, this study selected a concentration of

200 μM H_2O_2 for irradiation over the same time range (Supplementary Figs. 3a–b)[8]. The relative effect of H_2O_2 can be assessed by the ratio of rate constants. The k_{obs} values for SDZ degradation in the UV_{222}-PB and UV_{222}-HB systems were 0.2190 min^{-1} and 0.0410 min^{-1}, respectively, with a ratio of 5.34, indicating that SDZ degradation is primarily attributable to PAA rather than H_2O_2[15]. To further investigate the role of H_2O_2, exogenous H_2O_2 at different concentrations was introduced into the UV_{222}-PB system (Supplementary Figs. 3c–e). As the concentration of added H_2O_2 increased, the degradation rate of SDZ showed a gradual decrease. When the total H_2O_2 concentration reached four times the PAA concentration, the k_{obs} value of SDZ was only 0.0640 min^{-1}. This inhibitory effect can be attributed to the light-shielding effect caused by high H_2O_2 concentrations, which weakens the effective photoactivation of PAA and Br^- in the solution[28]. At the same time, excess H_2O_2 scavenges reactive species such as HOBr from the system, hindering the oxidative degradation of pollutants[29,30]. On the other hand, 20 units·mL^{-1} of catalase (CAT) was added to remove the coexisting H_2O_2 from the solution (Supplementary Figs. 3f–h). Unexpectedly, compared to the Dark-PB system (0.0252 min^{-1}), the Dark-PB with CAT system (0.1083 min^{-1}) exhibited a 3.30-fold increase in the rate constant for SDZ degradation. This is because HOBr, generated by the reaction of PAA with Br^-, can be reduced by coexisting H_2O_2, but when CAT is introduced into the reaction solution, H_2O_2 is removed, blocking the reduction pathway of HOBr and thereby unleashing the oxidative potential of HOBr. Similarly, CAT alone had almost no effect on promoting the degradation of SDZ under UV_{222} irradiation. However, when CAT was added, the degradation rate of SDZ in the UV_{222}-PB system increased by 41.05%. Since CAT can decompose H_2O_2 into O_2 and H_2O, we measured the changes in dissolved oxygen (DO) in the UV_{222}-PB system with and without CAT. Evidently, upon the addition of CAT, the initial DO concentration in the reaction solution rose from 8.24 mg·L^{-1} to 16.50 mg·L^{-1}, and as the reaction proceeded, the DO concentration in the solution gradually decreased (Supplementary Fig. 3i). Previous studies have shown that an increase in the DO concentration in the solution can effectively drive the chain reaction initiated by the UV_{222} photolysis of O_2, promoting the generation of reactive oxygen species such as •OH, which in turn enhances the

system's oxidative degradation of OMPs[11]."

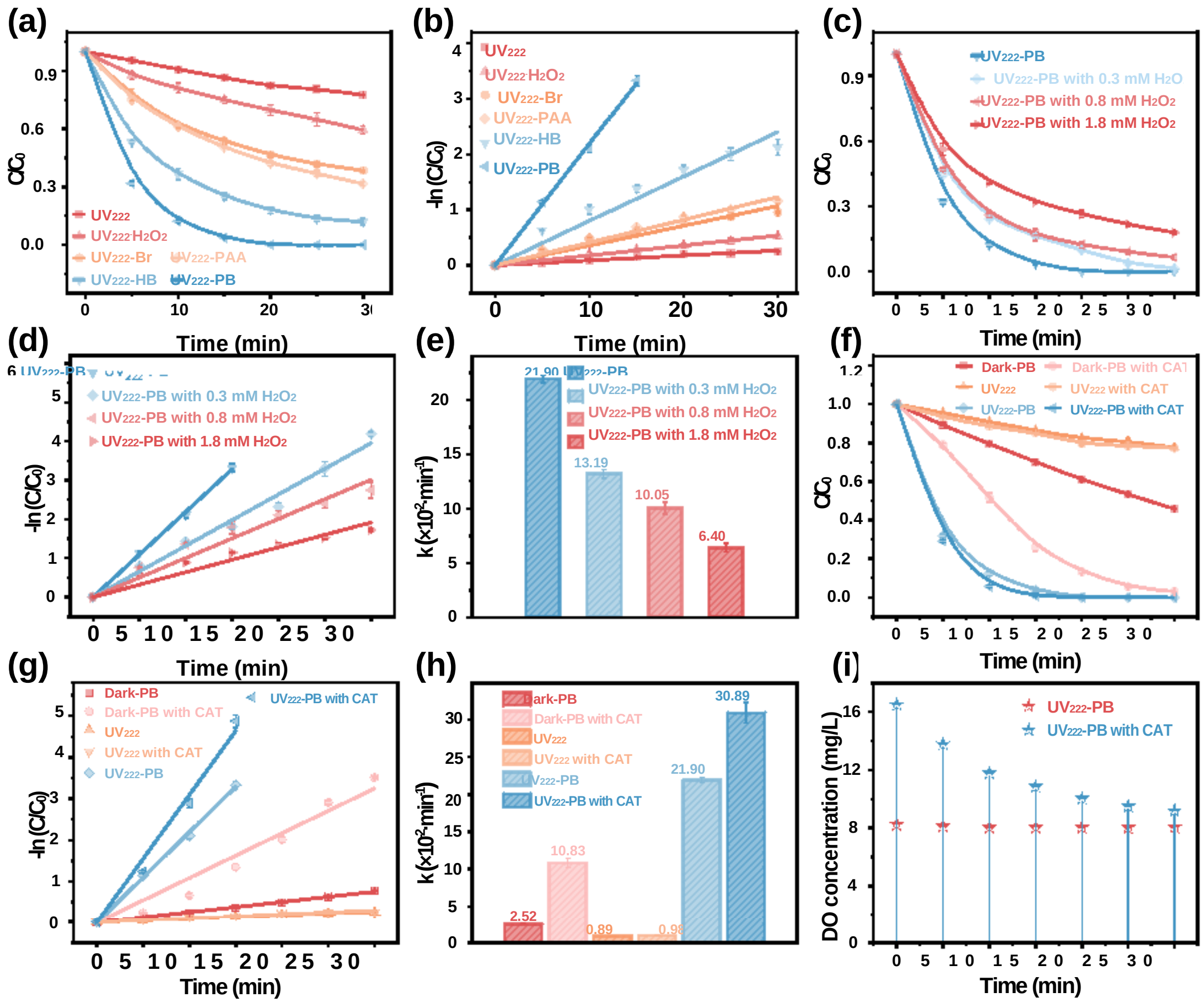

Supplementary Fig. 3 | (a) SDZ degradation curve and (b) pseudo first-order kinetic fitting curve of different processes under UV_{222}. (c) Degradation curves of SDZ at different H_2O_2 concentrations, (d) pseudo-first-order kinetic fitting curves, and (e) k_{obs} values. (f) Degradation curves of SDZ in the presence of CAT, (g) pseudo-first-order kinetic fitting curves, and (h) k_{obs} values. (i) Dissolved oxygen variations with and without CAT. Conditions: $[SDZ]_0$ = 20 μM, pH = 7.0, $[CAT]_0$ = 20 units·mL^{-1}, $[PAA]_0 = [Br]_0$ = 0.5 mM.

Comment 5: In the control experiments, the degradation of SDZ by the dark reaction of PAA + Br^- should be supplemented to evaluate the oxidizing capacity of HBrO.

Response: Thank you for your professional and meticulous review of our manuscript. In response to your valuable comment, we have supplemented control experiments under dark conditions for PAA alone, Br^- alone, H_2O_2 alone, and Dark-

PAA-Br (Dark-PB). The results showed that PAA, Br^-, and H_2O_2 exhibited negligible degradation of SDZ in the dark. However, because PAA can react with Br^- to generate HOBr ($k = 0.24\ M^{-1}\cdot s^{-1}$), Dark-PB achieved 54.27% SDZ removal within 30 min, confirming that HOBr indeed possesses a certain oxidative capacity toward SDZ. Nevertheless, the degradation rate of SDZ in UV_{222}-PB (0.2190 min^{-1}) was approximately 8.69 times that of Dark-PB (0.0252 min^{-1}), indicating that in the UV_{222}-PB process, the highly efficient degradation of pollutants originates not only from the formation of HOBr, but more critically from the synergistic activation between PAA and Br^- under far-UVC irradiation. These results have been presented in **Lines 134–135, 152–160** and **Supplementary Fig. 1** of the manuscript. Thank you again for your insightful correction, which has allowed us to gain a clearer understanding of the core role of UV_{222} photoactivation.

The related descriptions and images are presented below for your convenience:

Lines 134–135: "The results showed that under dark conditions, the degradation of SDZ by PAA alone or Br^- alone was almost negligible (Supplementary Fig. 1a)."

Lines 152–160: "Notably, the Dark-PB system under dark conditions achieved 54.27% removal of SDZ within 30 minutes (Supplementary Fig. 1), which can be attributed to the reaction between PAA and Br^- to generate HOBr ($k = 0.24\ M^{-1}\cdot s^{-1}$), leading to the oxidative degradation of SDZ[24]. However, the k_{obs} value of Dark-PB (0.0252 min^{-1}) was only 0.12 times that of UV_{222}-PB, indicating that under far-UVC irradiation, the interaction between PAA and Br^- may trigger extended radical chain reactions, thereby increasing the amount of reactive species in the system and enhancing the degradation efficiency (see synergistic interaction mechanism for more information)."

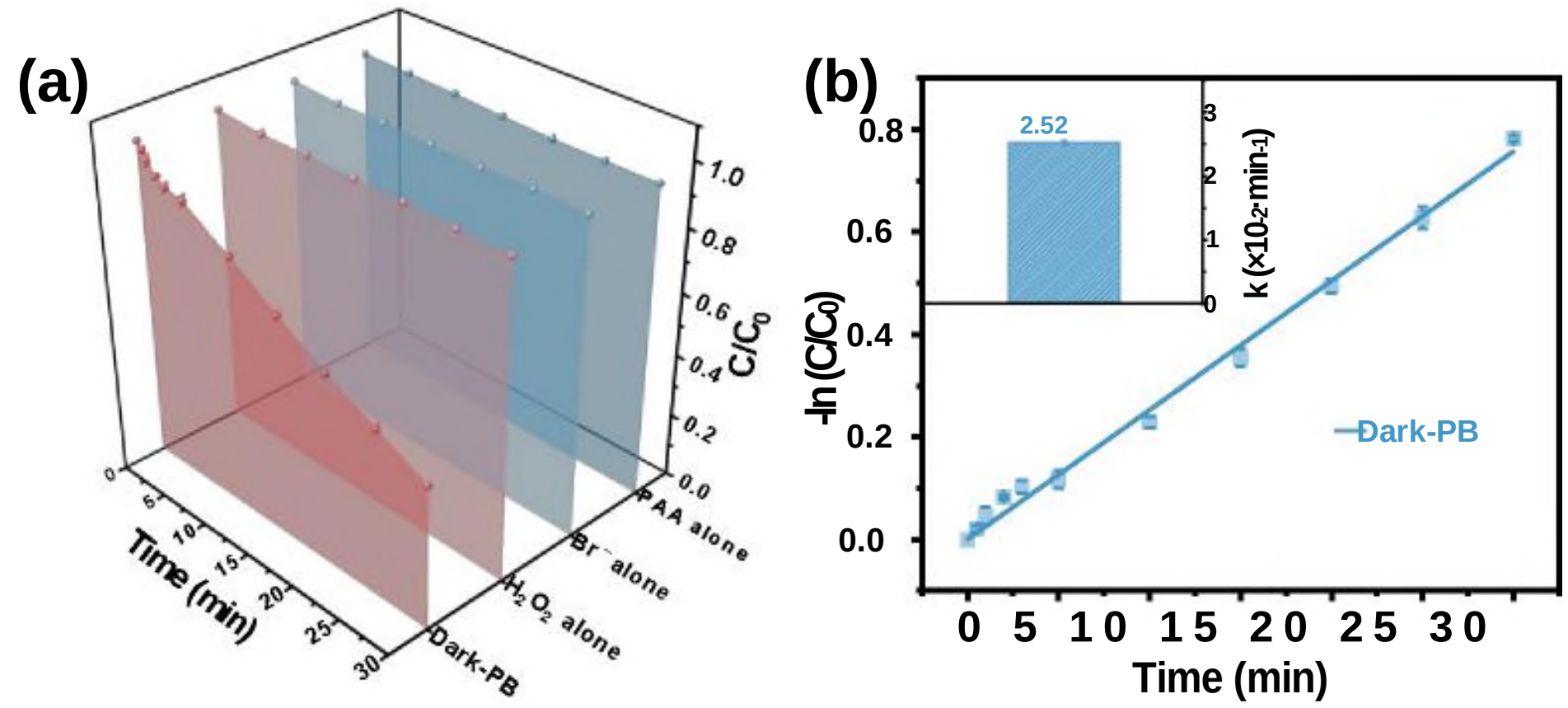

Supplementary Fig. 1 | (a) Degradation of SDZ in different systems under dark conditions. (b) Pseudo-first-order kinetic fitting curves and k_{obs} values for SDZ degradation in the Dark-PB system.

Comment 6: In the LCA evaluation, is it appropriate to use the degradation data from batch reactions (50 mL SDZ) as input parameters? Moreover, the LCA assessment only compares different combinations of UV_{222}, PAA, and Br^-, which is somewhat one-sided; it should be compared with conventional seawater treatment technologies.

Response: Thank you very much for pointing out these two critical issues in the LCA evaluation. In the foundational stage of process development, using laboratory batch experimental data allows the core parameters such as electrical energy consumption and chemical dosage to be obtained under more strictly controlled conditions, thereby revealing the intrinsic performance boundary of the process under ideal conditions. However, evaluation results based solely on ideal conditions are insufficient to fully reflect the environmental performance in practical applications. Therefore, we further conducted an LCA based on continuous-flow experiments. The results show that, in continuous experiments, the UV_{222}-PB process demonstrates excellent environmental performance while maintaining a low carbon footprint, which further enhances the engineering application potential of this process. Meanwhile, electricity input is the primary factor influencing the environmental pressure of this process. Therefore, we further incorporated the electricity mix change trends reported in previous studies to evaluate the impact of grid cleaning on the environmental

performance of the UV_{222}-PB process against the backdrop of the deepening global energy transition. The results show that by 2060, with the substantial increase in the share of clean energy in the electricity mix, the environmental advantages of this process will be further amplified.

In addition, we selected typical water treatment processes such as O_3 oxidation, O_3/H_2O_2, and TiO_2 photocatalysis for comparative analysis. To ensure fairness and consistency in the comparison, we extracted life cycle inventory data for each process from the literature and re-calculated them using the same LCA methodology and system boundary as in this study. The relevant supplementary results are presented in **Lines 882–933**, **Fig.7** and **Supplementary Figs. 38–39** of the manuscript. Once again, thank you for your constructive suggestion, which is of great significance for enhancing the engineering basis and the reliability of the LCA assessment in this study.

The related descriptions and images are presented below for your convenience:

Lines 882–933: "Based on the aforementioned water quality parameters and the excellent results from in-situ water body experiments, this study further investigated the long-term stability of the UV_{222}-PB system in a custom-built continuous operation setup to provide a reference for its industrial-scale upscaling. The results showed that after 72 h of continuous operation, the degradation efficiency of SDZ by the UV_{222}-PB system remained above 98%, with a cumulative treated wastewater volume of 16.8 L. Moreover, considering that the theoretical hydraulic retention time of the pollutant solution in the reactor was approximately 1 h, we monitored the degradation of SDZ during the first 1 h of reactor operation. The results indicated that the k_{obs} value for SDZ degradation reached 5.32 h^{-1}, and SDZ was almost completely degraded in the reactor effluent after 1 h of operation (Fig. 7h and Supplementary Figs. 34–35). This occurrence further demonstrated the robust stability and significant potential for effective pollutant removal of UV_{222}-PB system, offering new insights for practical water treatment applications.

Additionally, the sustainability and environmental impacts of different processes were comprehensively and systematically quantified through life cycle assessment (LCA) (Supplementary Table 17)[77]. The results showed that, compared with processes

such as UV_{254}-PB, the UV_{222}-PB process demonstrated superior environmental performance across multiple environmental impact indicators, confirming its favorable environmental friendliness and sustainability (Fig. 7i and Supplementary Figs. 36–37). Moreover, in the 16.8 L continuous-flow experiment, the UV_{222}-PB (CF) process maintained high pollutant removal efficiency while sustaining a low carbon emission level. This further verified a good balance between its environmental friendliness and treatment efficiency, providing solid data support for its future engineering application. To further clarify the positioning and advantages of the UV_{222}-PB (CF) process among water treatment technologies, this study compared it with other typical water treatment processes, targeting the treatment of 1 m^3 of water[33,78]. To strengthen the fairness and consistency of the comparison, we extracted the life cycle inventory data of each process and recalculated them using the same LCA methodology as in this study. It can be seen that the UV_{222}-PB (CF) process outperformed the compared processes such as O_3, CaO_2/O_3, and TiO_2 photocatalysis in most environmental impact categories (Supplementary Fig. 38). Among them, the CO_2 emissions of UV_{222}-PB were 31.34 kg CO_2 eq·m^{-3}, which is only 35.32% of that of the CN-KI/PI/Sunlight process (88.72 kg CO_2 eq·m^{-3}). Notably, since the UV_{222}-PB process does not involve catalyst preparation, electricity input has become the main factor affecting its environmental pressure. According to Zhu et al.'s projection of China's electricity mix in 2060, under the policy-driven scenario, the share of thermal power will drop to approximately 2.07%, while wind power and photovoltaic power will account for 36.5% and 36.9%, respectively[79]. Based on this, this study further assessed the life cycle environmental impacts of the UV_{222}-PB (CF) process under the different electricity mixes in 2025 and 2060 (Fig. 7j and Supplementary Fig. 39). The results indicate that when the electricity mix changes, indicators such as surplus ore potential (SOP) and terrestrial ecotoxicity potential (TETP) show an upward trend due to the upstream metal mining and refining activities driven by the large-scale deployment of wind and solar power[80]. However, with the continuous clean transition of the electricity mix, environmental impact indicators such as global warming potential (GWP) and fossil fuel potential (FFP) of the UV_{222}-PB process decrease significantly, with CO_2 emissions dropping from 31.34 kg CO_2 eq·m_{-3}

to 3.73 kg CO_2 eq·m^{-3}. Overall, against the backdrop of the deepening global energy transition, grid decarbonization will further amplify the environmental advantages of the UV_{222}-PB process. For future development, it is recommended to increase the operating load rate of electrical equipment to as close to saturation as possible, thereby helping to reduce the environmental burden.”

(Redaction)

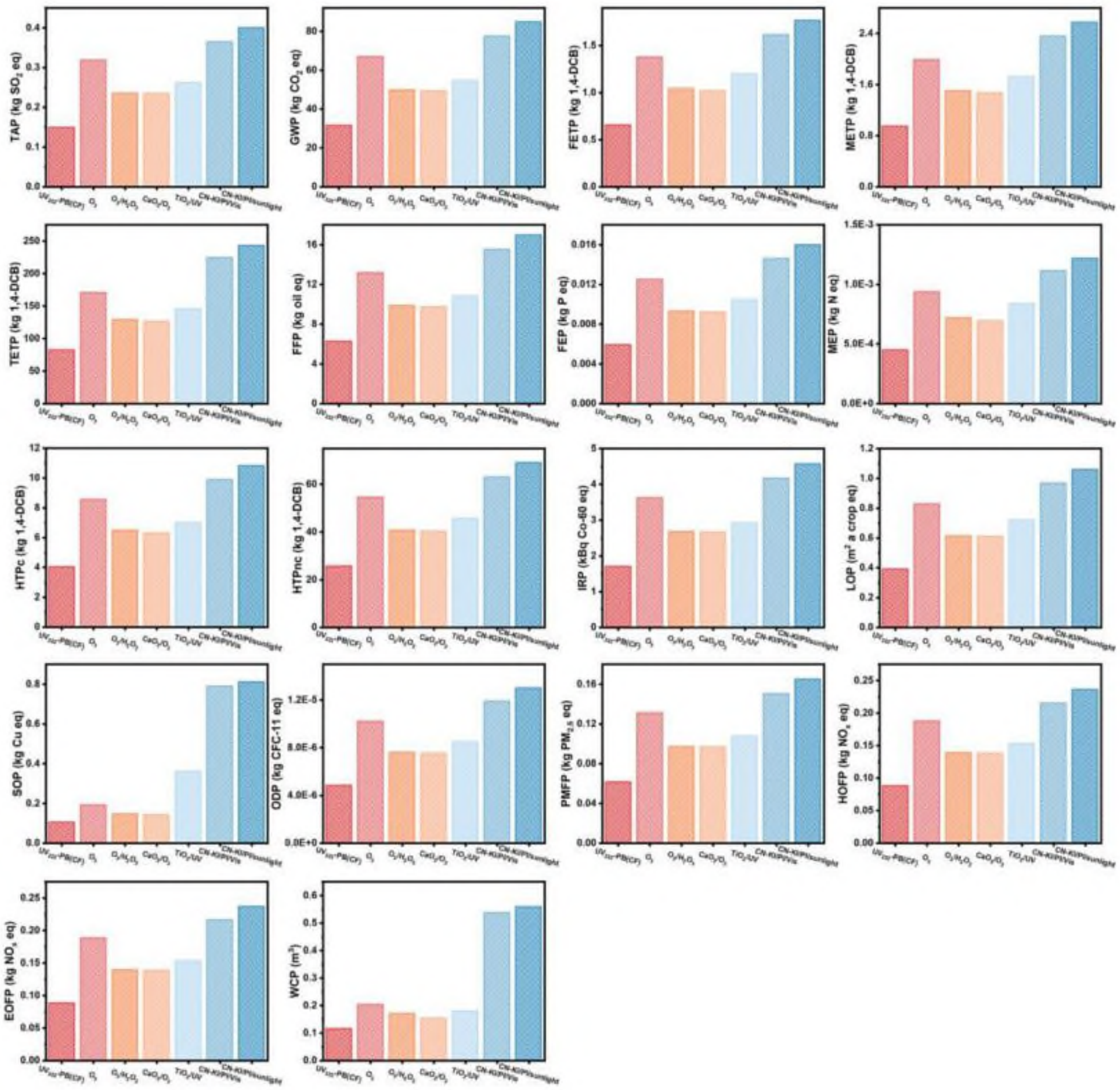

Supplementary Fig. 38 | Comparison of environmental impacts in life cycle assessments of different processes.

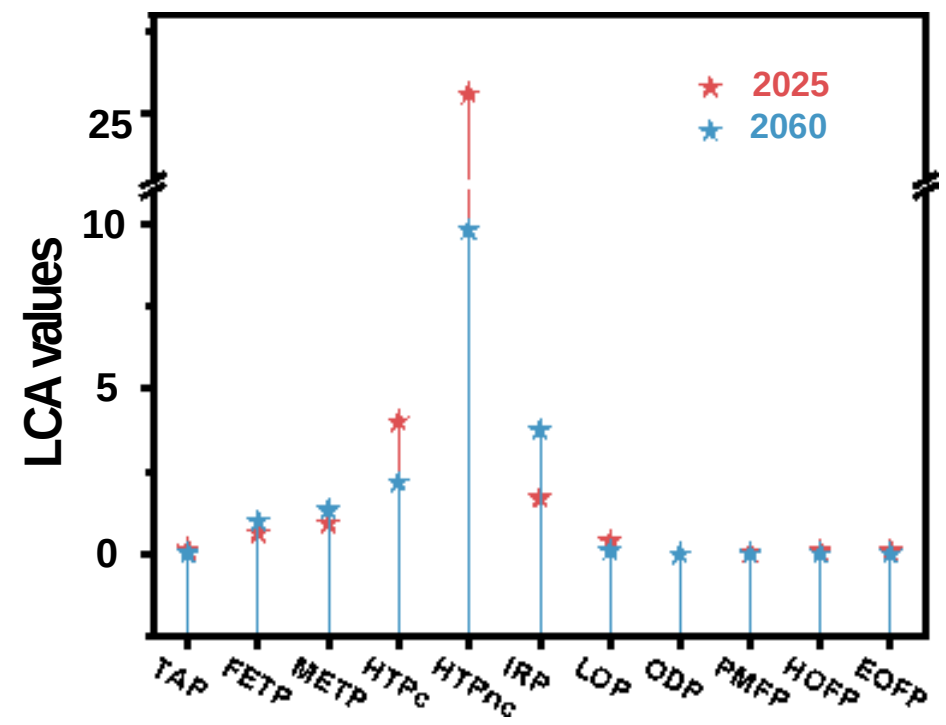

Supplementary Fig. 39 | Comparison of the environmental impacts of UV_{222}-PB (CF) in life cycle assessments for 2025 and 2026.

Comment 7: What is the degradation rate of SDZ in the continuous-flow experiment? Why were the batch experiments chosen for the LCA assessment instead of the continuous-flow experiment? In the continuous experiment, Br^- should be pre-mixed with SDZ rather than injected separately like PAA, because Br^- is a naturally coexisting inorganic ion in the water to be treated (seawater).

Response: Thank you very much for your thorough review and meticulous corrections of this work. The comments you raised guided us to re-examine the design logic of the continuous operation experimental scheme, which is of great guiding value for improving the methodological logic of this study. Based on your valuable suggestions, we have refined the continuous operation experiments. Specifically, a mixed solution containing 20 μM SDZ and 0.5 mM Br^- was continuously delivered to the reactor through a peristaltic pump, while PAA was simultaneously injected into the same reactor via a syringe pump. The reactor was continuously exposed to far-UVC radiation throughout the entire operation period. The experimental results show that the degradation efficiency of SDZ in the UV_{222}-PB system remained above 98% after 72 h of continuous operation. Given that the theoretical hydraulic retention time of the reaction solution in the reactor is approximately 1 h, we monitored the SDZ degradation during the first 1 h of the continuous operation experiment and fitted the data using a pseudo-first-order kinetic model, yielding a degradation rate constant of 5.32 h^{-1}.

Furthermore, regarding the selection of LCA evaluation data, the main reason for employing laboratory batch experimental data for LCA assessment during the fundamental research stage is that it can better reveal the intrinsic performance boundary of the process. Under batch experimental conditions, the intrinsic kinetic parameters of the reaction (such as electrical energy consumption and PAA dosage) can be obtained in a more strictly controlled environment. At the same time, we fully agree with your emphasis on continuous-flow experimental evaluation, which provides an important reference for the industrial scale-up of our process. Therefore, we have supplemented the manuscript with an LCA evaluation under continuous operation experiments, aiming to more comprehensively reveal the environmental impact potential of this process under practical application scenarios. The relevant results are presented in **Lines 882–933** of the manuscript, **Lines 165–173** of the supplementary information, **Fig.7**, **Supplementary Figs. 34–35, 38–39**. Once again, we sincerely thank you for your valuable comments, which are of great guiding significance for enhancing the practical application value of this process.

The related descriptions and images are presented below for your convenience:

Lines 882–933: "Based on the aforementioned water quality parameters and the excellent results from in-situ water body experiments, this study further investigated the long-term stability of the UV_{222}-PB system in a custom-built continuous operation setup to provide a reference for its industrial-scale upscaling. The results showed that after 72 h of continuous operation, the degradation efficiency of SDZ by the UV_{222}-PB system remained above 98%, with a cumulative treated wastewater volume of 16.8 L. Moreover, considering that the theoretical hydraulic retention time of the pollutant solution in the reactor was approximately 1 h, we monitored the degradation of SDZ during the first 1 h of reactor operation. The results indicated that the k_{obs} value for SDZ degradation reached 5.32 h^{-1}, and SDZ was almost completely degraded in the reactor effluent after 1 h of operation (Fig. 7h and Supplementary Figs. 34–35). This occurrence further demonstrated the robust stability and significant potential for effective pollutant removal of UV_{222}-PB system, offering new insights for practical water treatment applications.

Additionally, the sustainability and environmental impacts of different processes were comprehensively and systematically quantified through life cycle assessment (LCA) (Supplementary Table 17)[77]. The results showed that, compared with processes such as UV_{254}-PB, the UV_{222}-PB process demonstrated superior environmental performance across multiple environmental impact indicators, confirming its favorable environmental friendliness and sustainability (Fig. 7i and Supplementary Figs. 36–37). Moreover, in the 16.8 L continuous-flow experiment, the UV_{222}-PB (CF) process maintained high pollutant removal efficiency while sustaining a low carbon emission level. This further verified a good balance between its environmental friendliness and treatment efficiency, providing solid data support for its future engineering application. To further clarify the positioning and advantages of the UV_{222}-PB (CF) process among water treatment technologies, this study compared it with other typical water treatment processes, targeting the treatment of 1 m^3 of water[33,78]. To strengthen the fairness and consistency of the comparison, we extracted the life cycle inventory data of each process and recalculated them using the same LCA methodology as in this study. It can be seen that the UV_{222}-PB (CF) process outperformed the compared processes such as O_3, CaO_2/O_3, and TiO_2 photocatalysis in most environmental impact categories (Supplementary Fig. 38). Among them, the CO_2 emissions of UV_{222}-PB were 31.34 kg CO_2 eq·m^{-3}, which is only 35.32% of that of the CN-KI/PI/Sunlight process (88.72 kg CO_2 eq·m^{-3}). Notably, since the UV_{222}-PB process does not involve catalyst preparation, electricity input has become the main factor affecting its environmental pressure. According to Zhu et al.'s projection of China's electricity mix in 2060, under the policy-driven scenario, the share of thermal power will drop to approximately 2.07%, while wind power and photovoltaic power will account for 36.5% and 36.9%, respectively[79]. Based on this, this study further assessed the life cycle environmental impacts of the UV_{222}-PB (CF) process under the different electricity mixes in 2025 and 2060 (Fig. 7j and Supplementary Fig. 39). The results indicate that when the electricity mix changes, indicators such as surplus ore potential (SOP) and terrestrial ecotoxicity potential (TETP) show an upward trend due to the upstream metal mining and refining activities driven by the large-scale deployment of wind and solar power[80]. However, with the

continuous clean transition of the electricity mix, environmental impact indicators such as global warming potential (GWP) and fossil fuel potential (FFP) of the UV_{222}-PB process decrease significantly, with CO_2 emissions dropping from 31.34 kg CO_2 eq·m^{-3} to 3.73 kg CO_2 eq·m^{-3}. Overall, against the backdrop of the deepening global energy transition, grid decarbonization will further amplify the environmental advantages of the UV_{222}-PB process. For future development, it is recommended to increase the operating load rate of electrical equipment to as close to saturation as possible, thereby helping to reduce the environmental burden.

Supplementary Information Lines 165–173: "The continuous-flow experimental apparatus employed a peristaltic pump (LabV) to deliver a mixed reaction solution containing 20 μM SDZ and 0.5 mM Br^- into a 250 mL reactor at a flow rate of 240.0 mL·h^{-1}. Simultaneously, a syringe pump (XMSP-3C channel) was used to inject a 25 mM PAA solution into the same reactor at a flow rate of 4.8 mL·h^{-1}. The reactor was continuously exposed to 222 nm far-UVC radiation throughout the operation. At predetermined intervals, collect 0.7 mL samples, filter them through a 0.22 μm membrane filter, and inject them into brown sample vials. Subsequently, analyze the residual SDZ concentration in the solution via high-performance liquid chromatography."

(Redaction)

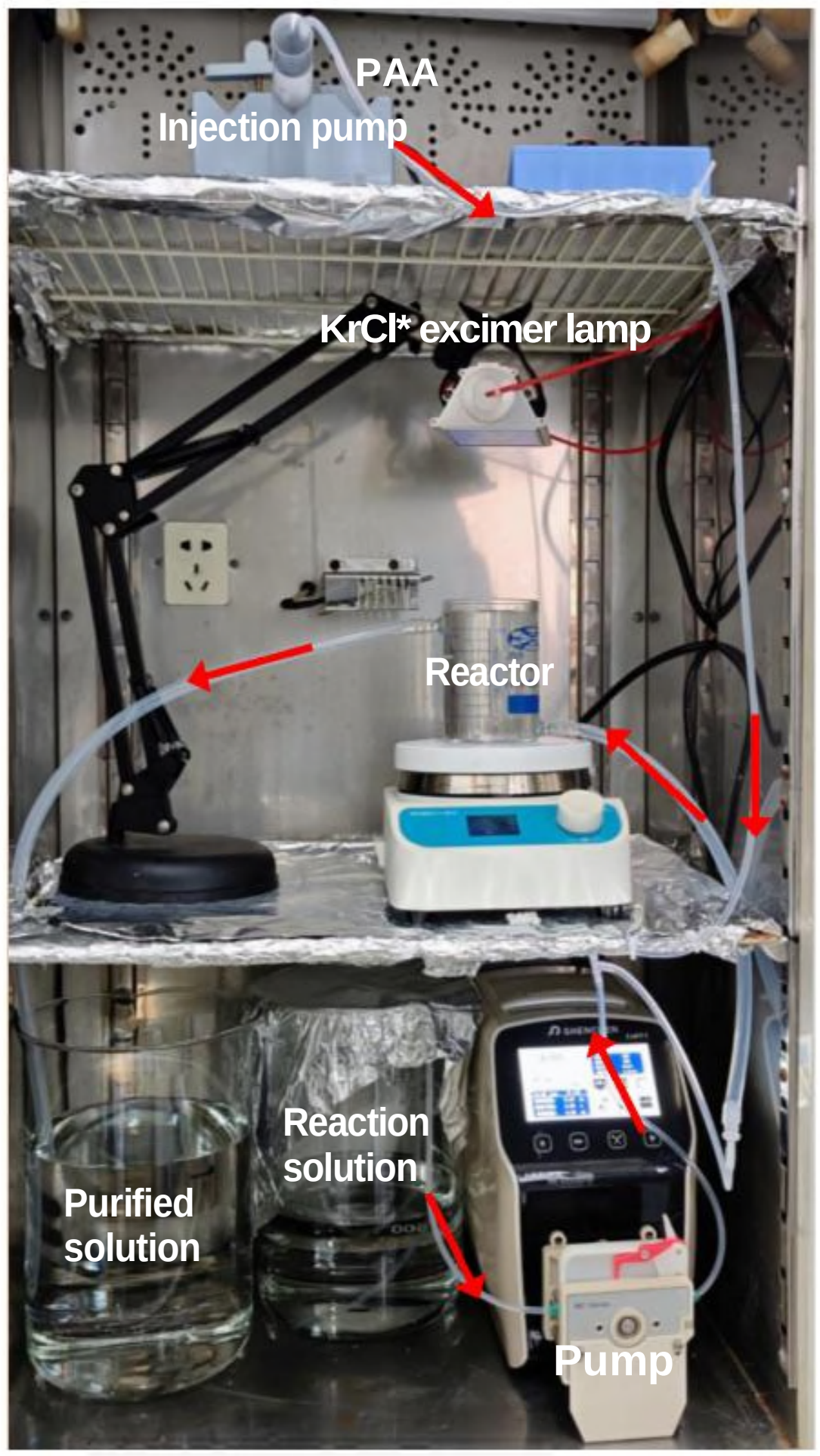

Supplementary Fig. 34 | Actual digital images of the continuous-flow apparatus

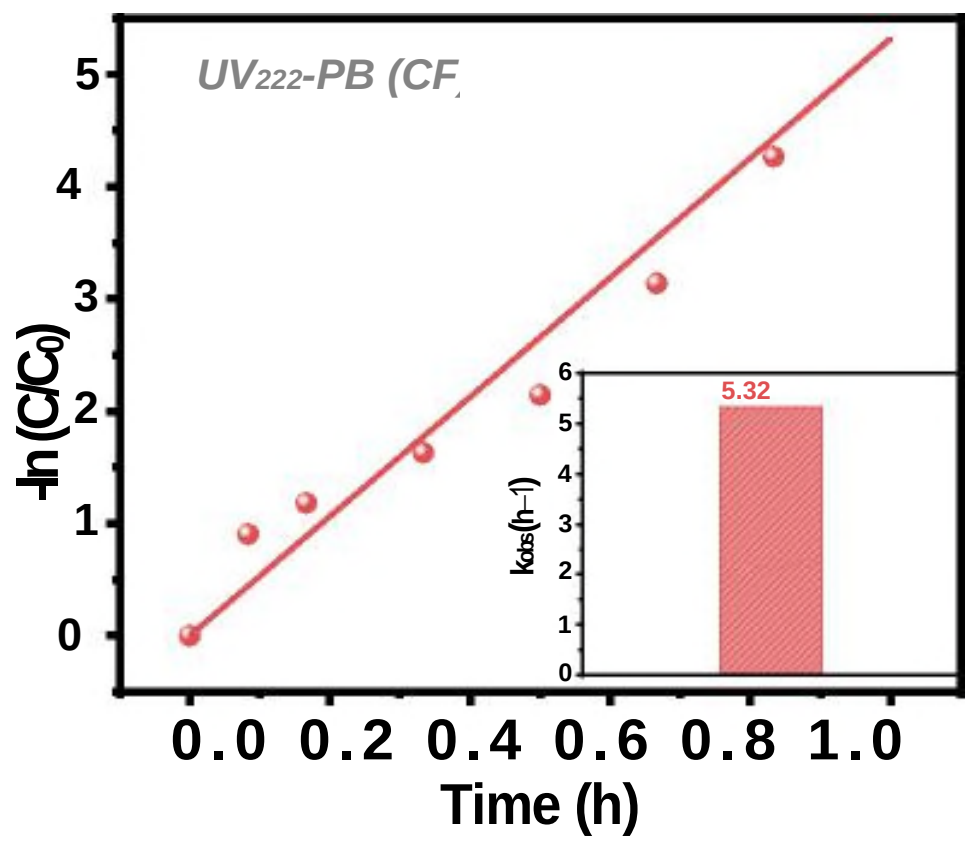

Supplementary Fig. 35 | Pseudo-first-order kinetic fit curves and kobs values for the UV222-PB continuous-flow UV222-PB (CF) system.

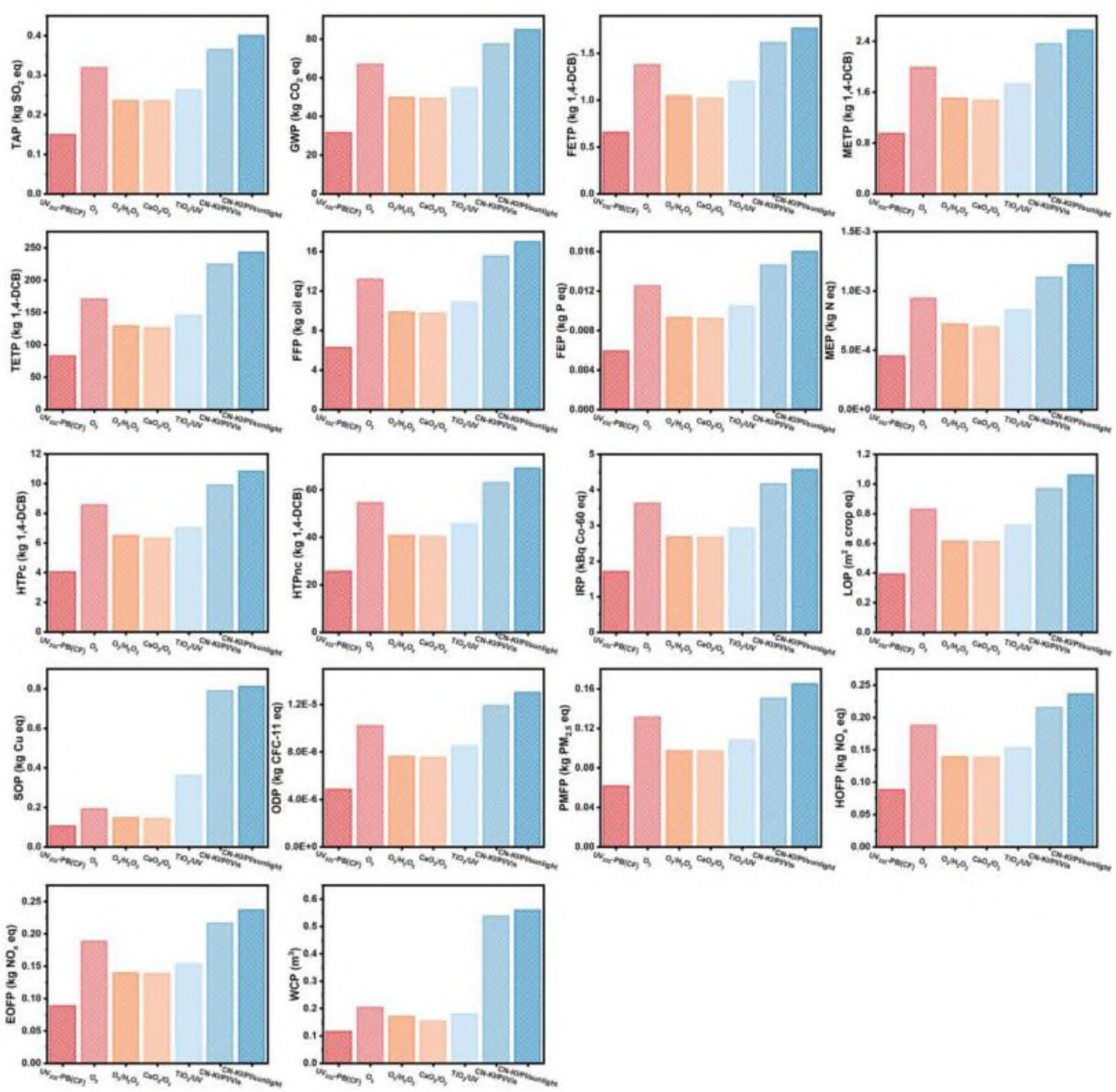

Supplementary Fig. 38 | Comparison of environmental impacts in life cycle assessments of different processes.

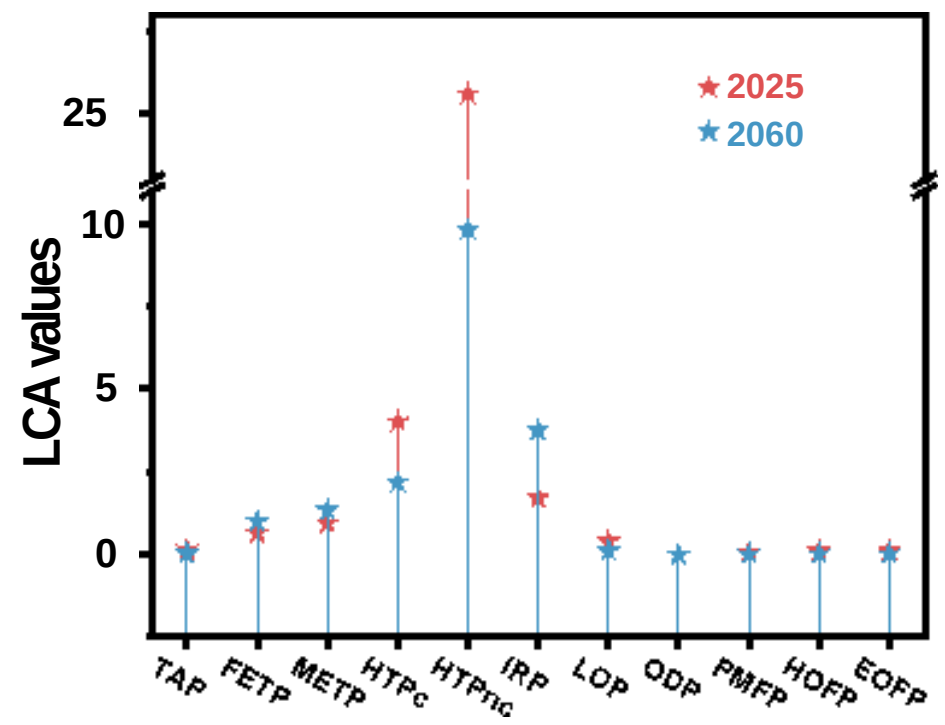

Supplementary Fig. 39 | Comparison of the environmental impacts of UV_{222}-PB (CF) in life cycle assessments for 2025 and 2026.

Comment 8: The authors seem to have only provided a method for correcting light intensity, but not a method for measuring it. Please supplement the emission spectrum diagram with information on UV_{365}.

Response: We sincerely thank you for your comprehensive and insightful comments on our manuscript. In this study, the ultraviolet irradiance at the center of the solution surface (E_0) was directly measured using a UV radiometer (TS280E, Shenzhen Suderui Technology Co., Ltd., China). The measured E_0 values for the three light sources were 0.4770 mW·cm^{-2} for UV_{222}, 0.9483 mW·cm^{-2} for UV_{254}, and 0.5895 mW·cm^{-2} for UV_{365}, respectively. In the subsequent calculation of the average fluence rate (E_{avg}), we introduced the water factor (WF), pries factor (PF), reflectance factor (RF), and divergence factor (DF) to correct the measured E_0 values, thereby more accurately reflecting the effective light intensity actually received by the solution in the reaction system. In addition, we have supplemented the relevant information regarding the UV_{365} light source in both the revised manuscript and the Supporting Information. The specific additions have been incorporated into the revised manuscript (**Lines 974– 983**) and the Supporting Information (**Lines 51–57** and **Supplementary Fig. 41**). Once again, we sincerely appreciate your professional review, which has significantly enhanced the rigor of our work.

The related descriptions and images are presented below for your convenience:

Lines 974–983: "In pollutant degradation experiments, the UV irradiation apparatus (Supplementary Fig. 40) employed either a KrCl* excimer lamp (UV_{222}, FIRST B2-15W, Guangdong Excimer Optoelectronic Technology Co., Ltd., China), an LPUV lamp (UV_{254}, 10W, Guangzhou Xingchuang Electronics Co., Ltd., China), or an MPUV lamp (UV_{365}, 10W, Guangzhou Xingchuang Electronics Co., Ltd., China) as the ultraviolet radiation source, coupled with a bandpass filter. The emission spectra of the lamps are shown in Supplementary Fig. 41. Before experiment began, the KrCl* excimer lamp required 5 minutes warm-up period (LPUV and MUPV lamps required 30 minutes) to ensure stable light intensity. For detailed calculations of UV measurements, refer to Text S2."

Supporting Information Lines 51–57: "Among them, E_{avg} represents the average flux rate (mW·cm^{-2}), t represents the exposure time (s) of pollutants, E_{Inc} represents the incident ultraviolet flux rate (mW·cm^{-2}), and E_0 represents the ultraviolet irradiance at the center of the solution surface (mW·cm^{-2}), as measured using ultraviolet radiometer (TS280E, Shenzhen Suderui Technology Co., Ltd., China. 0.4770 mW·cm^{-2} for UV_{222}, 0.9483 mW·cm^{-2} for UV_{254} and 0.5895 mW·cm^{-2} for UV_{365}). WF, PF, RF, DF represent the water factor, pries factor (0.90–0.91), reflectance factor (0.973), and divergence factor, respectively."

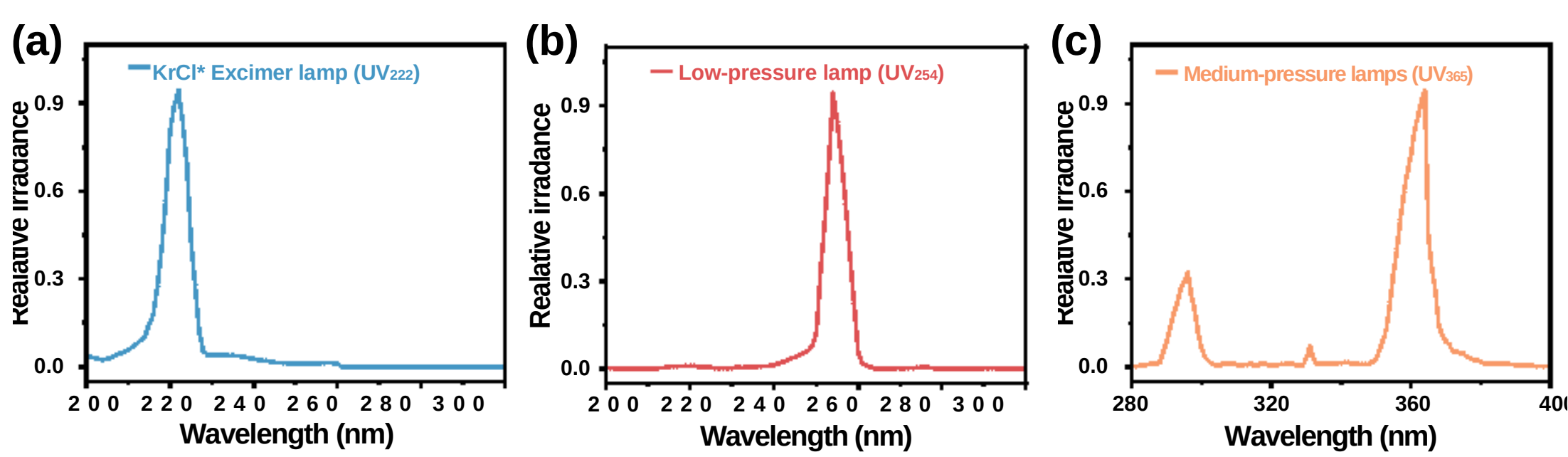

Supplementary Fig. 41 | Emission spectra of different light sources.

Reviewer #2

General Comments: *The author's response meets all my concerns, addressing each point with clarity and thoroughness, demonstrating a deep understanding of the subject matter. The revisions made to the manuscript significantly enhance its quality and rigor, making it a valuable contribution to the field. Given the importance of the research findings and the meticulous attention to detail in the author's revisions, I believe this manuscript is now well-suited for publication in Nature Communications.*

Response: We would like to express our sincere gratitude for your thorough and professional review of our manuscript. Your affirmation of this study and your recognition of the quality of our work have been a great encouragement to us. The rigorous academic attitude and profound insight you have demonstrated throughout the review process have not only significantly enhanced the scholarly merit of this paper, but also instilled in us greater confidence in pursuing our future research. Once again, we extend our most heartfelt thanks to you.

Reviewer #3

General Comments: *The authors of the manuscript titled "Bromide-Mediated Enhancement of Far-UVC/Peracetic Acid Process for Selective..." have appropriately revised the paper in response to the reviewers' comments, and the revised version now meets the requirements of Nature Communications. I recommend acceptance of this paper.*

Response: Your recognition of our work has greatly encouraged us. The rigorous academic approach and professional dedication you have demonstrated throughout the review process have set a benchmark for our research. We will take this as motivation to continue delving deeper into this field and to approach our future work with even greater rigor and commitment. Once again, we extend our sincerest gratitude to you.
