# Supplementary material for: Bromide-mediated enhancement of far-UVC/peracetic acid process for selective micropollutant abatement
Source: Nat Commun. 2026 Oct 8;17:11295. doi: 10.1038/s41467-026-78410-2 (PMC13722704; doi:10.1038/s41467-026-78410-2)
Supplement: Supplementary file 4 [file 41467_2026_78410_MOESM2_ESM.docx]

Supporting Information For

**Bromide-Mediated Enhancement of Far-UVC/Peracetic Acid Process for Selective Micropollutant Abatement**

Genying Zeng ^1 2^, Guoyu Li ^1^, Hou Wang ^1^, Chen Zhang ^1^, Qiang Chen ^1 2^, Qinyuan Tang ^1 2^, Tangshan Wu ^1 2^, Li Li ^3^, Xinjiang Hu ^4 *^, Ran Yin ^5 6*^, Xiaofei Tan ^1 2 *^

^1^ College of Environmental Science and Engineering and Key Laboratory of Environmental Biology and Pollution Control (Ministry of Education), Hunan University, Changsha 410082, PR China

^2^ Shenzhen Research Institute of Hunan University, Shenzhen 518055, China

^3^ School of Plant Protection and Environment, Henan Institute of Science and Technology, Xinxiang, Henan, 453003, China

^4^ School of Ecology and Environment, Central South University of Forestry and Technology, Changsha 410004, PR China

^5^ State Key Laboratory of Water Pollution Control and Green Resource Reuse, School of the Environment, Nanjing University, Nanjing 210023, China

^6^ Institute for the Environment and Health, Nanjing University Suzhou Campus, Suzhou 215163, China

^*^Corresponding author: [xjhu@csuft.edu.cn](mailto:xjhu@csuft.edu.cn) (X. Hu), [yinran@nju.edu.cn](mailto:yinran@nju.edu.cn) (R. Yin), [tanxf@hnu.edu.cn](mailto:tanxf@hnu.edu.cn) (X. Tan).

The Supporting Information consists of 84 pages containing 12 texts, 18 tables, and 41 figures.

1. Supplementary Text
Text S1. Chemicals and Materials

Sodium hydroxide (NaOH, ≥ 96%), hydrochloric acid (HCl, 36 ~ 38%), disodium hydrogen phosphate (Na_2_HPO_4_, ≥ 99%), sodium dihydrogen phosphate (NaH_2_PO_4_, ≥ 98%), formic acid (≥ 99.5%), methanol (≥ 99.8%), potassium chloride (KCl, ≥ 99.5%), potassium nitrate (KNO_3_, ≥ 99%), potassium sulfate (K_2_SO_4_, ≥ 99%), potassium carbonate (K_2_CO_3_, ≥ 99%), sodium bicarbonate (NaHCO_3_, ≥ 99%), anhydrous sodium sulfate (Na_2_SO_4_, ≥ 99%), concentrated sulfuric acid (H_2_SO_4_, 95 ~ 98%), and 30% hydrogen peroxide (H_2_O_2_, ≥ 30%) were purchased from Sinopharm Chemical Reagent Co., Ltd. (Shanghai, China). Sulfadiazine (SDZ, ≥ 98%), sulfamethoxazole (SMX, ≥ 98%), sulfamethazine (SMZ, ≥ 99%), sulfathiazole (STZ, ≥ 98%), sulfaguanidine (SGD, ≥ 98%), tetracycline hydrochloride (TC, ≥ 96%), oxytetracycline hydrochloride (OTC, ≥ 95%), florfenicol (FLO, ≥ 98%), carbamazepine (CBZ, ≥ 98%), ciprofloxacin (CIP, ≥ 98%), tert-butanol (TBA, ≥ 99.5%), ammonium sulfate ((NH_4_)_2_SO_4_, ≥ 99%), Manganese sulfate(MnSO_4_, ≥ 99%), 4-bromo-3,5-dimethylpyrazole (4-Br-DMPZ, ≥ 99%), 3,5-dimethylpyrazole (DMPZ, ≥ 99%), 2,4-hexadiene (2,4-HD, ≥ 95%), benzoic acid (BA, ≥ 99.5%), sodium thiosulfate (Na_2_S_2_O_3_, ≥ 99%), potassium iodide (KI, ≥ 99%), catalase (CAT, ≥ 200000 unit/g) and methyl tert-butyl ether (MTBE, ≥ 99.9%) were supplied by Aladdin Biological Technology Co., Ltd. (Shanghai, China). Suwannee River III FA (3S101F, SRFA), Suwannee River III HA (3S101H, SRHA), and Suwannee River III NOM (2R101N, SRNOM) were purchased from the International Humic Substances Society (IHSS, USA). Superoxide dismutase (SOD, ≥ 20000 u/mg), potassium bromide (KBr, ≥99%), nitrotetrazolium blue chloride (NBT, ≥ 98%), and deuterated water (D_2_O, ≥99.9%) were purchased from Shanghai E-N Chemical Technology Co., Ltd. Commercial PAA stock solution (approximately 1.963 M PAA and 0.786 M H_2_O_2_) was purchased from Hunan Huihong Reagent Co., Ltd. Unless otherwise specified, chemicals and reagents used in this study were of analytical grade or higher and required no further purification. Moreover, deionized water (18.25 MΩ·cm) was utilized in all experimental techniques.

**Text S2.** Detailed information on ultraviolet dose calculation.

According to the research of Bolton and Linden et al.^1^, the ultraviolet dose (mW·cm^−2^) can be calculated by the following equations S1−S2:

$$\text{UV dose = }\text{E}_{\text{avg}}\text{×}\text{t}$$

(S1)

$$\text{E}_{\text{avg}}\text{ = }\text{E}_{\text{Inc}}\left( \text{WF} \right)\text{ = }\text{E}_{\text{0}}\text{(WF)(PF)(RF)(DF)}$$

(S2)

Among them, *E*_avg_ represents the average flux rate (mW·cm^−2^), *t* represents the exposure time (s) of pollutants, *E*_Inc_ represents the incident ultraviolet flux rate (mW·cm^−2^), and *E*_0_ represents the ultraviolet irradiance at the center of the solution surface (mW·cm^−2^), as measured using ultraviolet radiometer (TS280E, Shenzhen Suderui Technology Co., Ltd., China. 0.4770 mW·cm^−2^ for UV_222_, 0.9483 mW·cm^−2^ for UV_254_ and 0.5895 mW·cm^−2^ for UV_365_). WF, PF, RF, DF represent the water factor, pries factor (0.90–0.91), reflectance factor (0.973), and divergence factor, respectively.

DF is used to evaluate the effect of the distance between UV lamp and the surface of aqueous solution on UV irradiation intensity. WF is used to evaluate the attenuation effect of water on UV light, which is related to the background aqueous matrix. These parameters can be calculated respectively using equations S3−S4:

|  | DF = $\frac{L}{L+l}$ | (S3) |
| --- | --- | --- |
|  | $\text{WF = }\frac{\text{1-}\text{10}^{-\text{al}}}{\text{al}\text{·}\text{ln}\text{(10)}}$ | (S4) |

Herein, L represents the distance from the UV lamp to the surface of aqueous solution (cm), *a* denotes the decimal absorption coefficient (cm^−1^) or the absorbance at 1 cm path length, and *l* indicates the vertical path length of water in the petri dish (cm).

**Text S3.** Kinetic models for the degradation of organic pollutants.

The degradation of organic pollutants follows pseudo-first-order kinetics, with the degradation efficiency (DE) and reaction rate constant calculable via equations S5−S7, respectively:

$$\text{DE}\left( \text{\%} \right)\text{ = }\left（ \text{1-}\frac{\text{C}_{\text{t}}}{\text{C}_{\text{0}}} \right）\text{×100\%}$$

(S5)

$$\ln\left( \frac{\text{C}_{\text{t}}}{\text{C}_{\text{0}}} \right)\text{ = }{\text{-}\text{k}}_{\text{obs}}\text{×}\text{t}$$

(S6)

$$\ln\left( \frac{\text{C}_{\text{t}}}{\text{C}_{\text{0}}} \right)\text{ = }{\text{-}\text{k'}}_{\text{obs}}\text{×UV dose}$$

(S7)

Among them, *C*_0_ and *C_t_* represent the concentration of organic pollutants in the solution at 0 and *t* min respectively, *t* represents the reaction time (min), *k*_obs_ represents the pseudo first-order reaction rate constant (min^−1^), *k'*_obs_ represents the fluence-based degradation rate constants (cm^2^·mJ^−1^).

**Text S4.** Calculation of molar absorption coefficient and apparent quantum yield.

The molar absorption coefficient (*ε_λ_*, M^−1^·cm^−1^) of the target compound is calculated by equation S8:

$$\text{ε}_{\text{λ}}\text{ = }\frac{\text{A}}{\text{d C}}$$

(S8)

Where *A* is the absorbance of the target compound at *λ* nm, *d* (cm) is the path length of the quartz cuvette, and *C* (M) is the concentration of the target compound.

The apparent quantum yield ($\text{Φ}_{\text{λ}}$, M·Einstein^−1^) reflects the efficiency with which photon energy is utilized and is one of the key parameters for evaluating photolysis reactions. It can be calculated using the following equations S9−S10:

$$\text{Φ}_{\text{λ}}\text{ = }\frac{\text{10}\text{k}_{\text{λ}}\text{U}_{\text{λ}}}{\text{ln(10)}\text{E}_{\text{0,}\text{λ}}\text{ε}_{\text{λ}}}$$

(S9)

$$\text{U}_{\text{λ}}\text{ = }\frac{\text{hc}\text{N}_{\text{A}}}{\text{λ}}$$

(S10)

Among these, *k_λ_* (s^−1^) denotes the pseudo-first-order photolysis rate constant of the target compound under UV_222_ or UV_254_ irradiation, *U_λ_* (J·Einstein^−1^) represents the molar photon energy, and $\text{E}_{\text{0, }\text{λ}}$ is the corrected flux rate. *λ*, *h*, *c* and *N_A_* represent wavelength (222 nm or 254 nm), Planck constant (6.626×10^−34^ J·s), light speed (2.997×10^8^ m·s^−1^) and Avogadro constant (6.022×10^23^ mol^−1^), respectively.

**Text S5.** Evaluation of the performance of the UV-PAA/Br process under 365 nm UV wavelength.

Since SDZ and Br^−^ exhibit almost no light absorption at 365 nm, the degradation of SDZ in the UV_365_ direct photolysis system and the UV_365_-Br system is negligible (Supplementary Fig. 5c, f−g). According to previous studies, although PAA cannot generate organic radicals under 365 nm irradiation, it can still produce a small amount of •OH, which explains the slight enhancement of SDZ degradation in the UV_365_-PAA system^2^. Notably, the fluence-based degradation rate constant (*k′*_obs_) of SDZ in the UV_365_-PB system is 721.43 times and 2.75 times higher than that in the UV_365_-Br and UV_365_-PAA systems, respectively. This is because PAA and Br^−^ can react to form HOBr, thereby promoting SDZ degradation. However, compared with the UV_365_-PB process (*k*′_obs_ = 0.00101 cm^2^·mJ^−1^), the degradation rate of the UV_222_-PB process (*k*′_obs_ = 0.0159 cm^2^·mJ^−1^) is 15.74 times higher, which further fully demonstrates the unique advantage of UV_222_ in synergistically activating PAA and Br^−^.

**Text S6.** The calculation of EE/O and electricity cost.

Electrical Energy per Order (EE/O) can be used to evaluate energy consumption efficiency. An economic assessment of the practical application potential of systems such as UV_222_-PB was conducted using EE/O. The total EE/O_T_ of the system includes the electrical energy consumption (EE/O_UV222, UV254_) for UV_254_ or UV_222_ irradiation, the equivalent electrical energy consumption (EE/O_PAA_) for PAA, and the equivalent electrical energy consumption (EE/O_Br_) for Br^−^, which can be calculated using equations S11−S14:

$$\text{EE/O}_{\text{T}}\text{ = }\text{EE/O}\text{UV}\text{222}\text{, UV}\text{254}\text{+}\text{EE/O}_{\text{PAA}}\text{+}\text{EE/O}_{\text{Br}}$$

(S11)

$$\text{EE/O}_{\text{UV}_{\text{222}}{\text{,}\text{UV}}_{\text{254}}}\text{ = }\frac{\text{P×t}}{\text{V×log}\text{(}\frac{\text{C}_{\text{0}}}{\text{C}_{\text{t}}}\text{)}}$$

(S12)

$$\text{EE/O}_{\text{A}}\text{ = }\text{α}_{\text{A}}\text{×}\frac{\text{[A]×1000}}{\text{log(}\frac{\text{C}_{\text{0}}}{\text{C}_{\text{t}}}\text{)}}$$

(S13)

$$\text{α}_{\text{A}}\text{ = }\frac{\text{Price×}\text{M}_{\text{A}}}{\text{10}^{\text{6}}\text{×}\text{Purity×AEC}}$$

(S14)

Where P denotes the total electrical power input to the system (kW), t represents the reaction time (h), V denotes the reaction solution volume (L, V = 0.05 L), *C*_0_ and *C_t_* represent the SDZ concentrations in the solution at 0 and t min, respectively (mol·L^−1^), *α_A_* is the energy consumed per mole of substance produced by equivalent electricity generation (kW·h·mol^−1^)^3^, [A] denotes the concentration of Br^−^ or PAA (mol·L^−1^), Price represents the average price of Br^−^ (RMB·ton^−1^), *M*_A_ is the molar mass (g·mol^−1^), Purity indicates the purity (%), and AEC denotes the average electricity cost (0.65 RMB·kW^−1^·h^−1^)^4^. The average price of KBr (99% by weight) on Alibaba.com was 10000 RMB·ton^−1^ (2025). Therefore, α_PAA_ and α_Br_ are 1.41 kW·h·mol^−1^ and 1.85 kW·h·mol^−1^, respectively. The calculated EE/O values for different process systems are shown in Fig. 1g.

**Text S7.** Sensitivity analysis.

Based on the original simulation curve, the rate constant of each reaction pathway was individually increased and decreased by 10%, and the resulting C*_t_*/C_0_ degradation curves of SDZ were recalculated. The difference between the two perturbed curves was then compared, and the sensitivity coefficient for each reaction was calculated using the central difference method (equation S15):

$$\text{Sensitivity = }\frac{\text{C}_{\text{t}}\text{/}\text{C}_{\text{0}}\text{(+10\%)}\text{-}\text{C}_{\text{t}}\text{/}\text{C}_{\text{0}}\text{(-10\%)}}{\left[ \text{ln(1.1)-ln(0.9)} \right]}$$

(S15)

The maximum absolute value of the sensitivity over the entire reaction time was taken as the bar height, representing the maximum possible influence of each reaction pathway during the reaction process. A higher bar indicates that the SDZ degradation result is more sensitive to the rate of that reaction, whereas a lower bar suggests a weaker impact of that reaction on the current simulation results.

**Text S8.** Experimental design.

In the radical scavenging experiments, 10 mM-100 mM TBA, MnSO_4_, (NH_4_)_2_SO_4_, and 1000 U-20000 U SOD were introduced into the system as quenchers for •HO, •RO, HOBr, and •O_2_^−^, respectively. In the probe experiments, 0.2 mM BA and 50 μM NBT were selected as probe compounds to further detect •HO and •O_2_^−^. In this process, BA reacts with •OH to form p-hydroxybenzoic acid (p-HBA), m-hydroxybenzoic acid (m-HBA), and o-hydroxybenzoic acid (o-HBA), with a production ratio of approximately 1.2:2.3:1.7. Using a conversion factor of 5.87, the concentration of •OH can be estimated from the measured concentration of p-HBA^5^. Additionally, DMPZ was selected as the probe compound for HOBr due to its inherent reactivity during halogenation, enabling it to rapidly react with HOBr to form 4-Br-DMPZ (equation S16)^6^.

$$\text{}\text{+}\text{HOBr}\text{ → }\text{}\text{ +}\text{H}_{\text{2}}\text{O}$$

(S16)

In the water quality parameter experiments, the initial pH of the solution was adjusted to 3, 6, 7, 8, 9 and 11 using 0.1 M HCl and 0.1 M NaOH, respectively, to investigate the effect of pH on the UV_222_-PB system. On the other hand, common inorganic anions (Cl^−^, NO_3_^−^, SO_4_^2−^ and CO_3_^2−^) and natural organic compounds (SRHA and SRFA) were selected to evaluate the stability of UV_222_-PB system. Specifically, in SDZ solutions adjusted to pH 7, inorganic anions at concentrations of 0.1 mM, 1 mM, 5 mM, and 10 mM, or natural organic matter at concentrations of 1 mg·L^−1^, 2 mg·L^−1^, 5 mg·L^−1^, and 10 mg·L^−1^, were added separately. After thorough mixing, the solutions were treated with an oxidizing agent and immediately exposed to UV_222_ irradiation to initiate the reaction. In the mixed anion experiments, the following solutions were prepared: trace-salinity simulated water (TSSW, 1 mM Cl^−^, 0.1 mM SO_4_^2−^, 0.1 mM CO_3_^2−^, 0.1 mM NO_3_^−^), low-salinity simulated water (LSSW, 1 mM Cl^−^, 1 mM SO_4_^2−^, 1 mM CO_3_^2−^, 1 mM NO_3_^−^), medium-salinity simulated water (MSSW, 10 mM Cl^−^, 5 mM SO_4_^2−^, 5 mM CO_3_^2−^, 5 mM NO_3_^−^), high-salinity simulated water (HSSW, 0.5 M Cl^−^, 10 mM SO_4_^2−^, 10 mM CO_3_^2−^, 10 mM NO_3_^−^), and other simulated water solutions at four different salinity levels. Additionally, the degradation of SDZ was evaluated in various aquatic environments, including Deionized Water (DW), Xiangjiang River water (XRW), Liuyang River water (LRW), Dongting Lake water (DLW), Yellow Sea (YS), and Aquaculture Seawater Wastewater (ASW). Detailed water quality parameters are provided in Supplementary Table 16

The continuous-flow experimental apparatus employed a peristaltic pump (LabV) to deliver a mixed reaction solution containing 20 μM SDZ and 0.5 mM Br^−^ into a 250 mL reactor at a flow rate of 240.0 mL·h^−1^. Simultaneously, a syringe pump (XMSP-3C channel) was used to inject a 25 mM PAA solution into the same reactor at a flow rate of 4.8 mL·h^−1^. The reactor was continuously exposed to 222 nm far-UVC radiation throughout the operation. At predetermined intervals, collect 0.7 mL samples, filter them through a 0.22 μm membrane filter, and inject them into brown sample vials. Subsequently, analyze the residual SDZ concentration in the solution via high-performance liquid chromatography.

**Text S9.** Details of analysis method.

HPLC (Agilent 1100 Infinity II) with an ultraviolet detector and a C18 column (5 μm, 4.6 × 250 mm) was used to quantify residual contaminant levels in the solution. Detailed measurement parameters for different contaminants are listed in Supplementary Table 1. Using the UV-visible spectrophotometer, the concentrations of PAA and coexisting H_2_O_2_ in the solution were determined with potassium iodide as an indicator^7^. Dissolved oxygen (DO) concentration was measured using a DO meter (HQ2200). NBT concentration was determined using a UV-visible spectrophotometer at a detection wavelength of 259 nm. The concentration of Br^−^ and BrO_3_^−^ in the sample was determined by ion chromatography (IC, AQUION) with an injection volume of 25 μL. The eluent consisted of Na_2_CO_3_ (3.2 mmol·L^−1^) and NaHCO_3_ (1.0 mmol·L^−1^), and the flow rate was 1.0 mL·min^−1^.

**Text S10.** HPLC-MS method.

The formation of 4-Br-DMPZ and intermediate products of SDZ degradation was analyzed using an Agilent 1290 ultra-performance liquid chromatography-Q-Tof 6550 mass spectrometer (UPLC-MS). The mobile phase consists of 0.1% formic acid aqueous solution (A) and methanol solution (B). The injection volume is 5 μL, the flow rate is 0.3 mL·min^−1^, and the column is a Waters BEH C18 (1.7 μm, 2.1×100 mm). The elution procedures are: 95% A and 5% B at 0 to 1 min, B increased from 5% to 95% at 1 to 8 min, 5% A and 95% B at 8 to 13 min, B decreased from 95% to 5% at 13 to 14 min, and 95% A and 5% B at 14 to 15 min. The ion source conditions are: sheath gas flow rate 12 L·min^−1^, sheath gas temperature 350℃, capillary voltage 4000 V. Among them, the mass spectrum scanning range of 4-Br-DMPZ is 50-1000 m/z, and the intermediate product of SDZ is 100-1500 m/z.

**Text S11.** ESR analysis.

Using 2,2,6,6-Tetramethylpiperidine (TEMP), 5,5-Dimethyl-1-pyrroline N-oxide (DMPO), and 5-Diisopropoxyphosphoryl-5-methyl-1-pyrroline N-oxide (DIPPMPO) as tracers, the types of free radicals in the system were identified through electron spin resonance (ESR, Bruker A300) to confirm the types of radicals present in the system. By adding specific system reagents to solutions containing TEMP or other trapping agents (e.g., 0.5 mM PAA for the UV_222_-PAA system, 0.5 mM Br^−^ for the UV_222_-Br system), reactions were conducted with UV_222_ irradiation. After 5 minutes of reaction, ESR signals in the solutions were detected. Detailed parameters of the ESR spectrometer are as follows: central field 3510 G, scan width 100 G, microwave frequency 9.85 GHz, modulation amplitude 1.0 G, modulation frequency 100 kHz.
**Text S12.** Ordinary differential equation model construction.

The model employs fully coupled ordinary differential equation (ODE) to describe the generation and consumption of pollutants, oxidants, free radicals, and halogen species. For each terminal reaction of SDZ, an accumulation variable *D_i_* is defined to directly calculate the contribution ratio of each pathway through the terminal state of the ODE. The SDZ degradation term and the terminal contribution variables are as follows (equations S17−S18):

$$\frac{\text{d}_{\text{[SDZ]}}}{\text{d}_{\text{t}}}\text{ = }{\text{-}\text{k}}_{\text{uv}\text{[SDZ]}}\text{-}\sum\text{k}_{\text{i}\left[ \text{C}_{\text{i}} \right]\text{[SDZ]}}$$

(S17)

$$\frac{\text{d}_{\text{D}_{\text{i}}}}{\text{d}_{\text{t}}}\text{ = }\text{k}_{\text{i}\left[ \text{C}_{\text{i}} \right]\text{[SDZ]}}$$

(S18)

Among them, *k*_uv_ represents the first-order rate constant (s^−1^) for direct photolysis of SDZ, *k_i_* represents the second-order rate constant (M^−1^·s^−1^) for the reaction between SDZ and the i-th reactive species, *C_i_* represents the instantaneous concentration (M) of the i-th reactive species, and Σ denotes the summation over all reactive species.

**2. Supplementary Tables**
**Supplementary Table 1 |** HPLC for different pollutants.

| Compound | Structure | Mobile phase | Flow speed | Detection wavelength |
| --- | --- | --- | --- | --- |
| SDZ |  | Methanol:0.1% formic acid = 20:80 | 1 mL·min^−1^ | 266 nm |
| SMX |  | Methanol:0.1% formic acid = 45:55 | 1 mL·min^−1^ | 267 nm |
| SMZ |  | Methanol:0.1% formic acid = 35:65 | 1 mL·min^−1^ | 263 nm |
| STZ |  | Methanol:0.1% formic acid = 25:75 | 1 mL·min^−1^ | 283 nm |
| SGD |  | Methanol:0.1% formic acid = 5:95 | 1 mL·min^−1^ | 259 nm |
| CIP |  | Methanol:0.1% formic acid = 25:75 | 1 mL·min^−1^ | 278 nm |
| CBZ |  | Methanol:Water  = 60:40 | 1 mL·min^−1^ | 280 nm |
| TC |  | Methanol:0.1% formic acid = 30:70 | 1 mL·min^−1^ | 356 nm |
| OTC |  | Methanol:0.1% formic acid = 25:75 | 1 mL·min^−1^ | 353 nm |
| FLO |  | Methanol:Water  = 60:40 | 1 mL·min^−1^ | 225 nm |
| p-HBA |  | Methanol:0.1% formic acid = 55:45 | 1 mL·min^−1^ | 255 nm |
| BA |  | Methanol:0.1% formic acid = 55:45 | 1 mL·min^−1^ | 254 nm |

**Supplementary Table 2 |** *k*_obs_ values of OMPs under UV_222_ irradiation for different processes.

| OMPs | UV_222_ | UV_222_-Br | UV_222_-PAA | UV_222_-PB |
| --- | --- | --- | --- | --- |
| SDZ | 0.0089 min^−1^ | 0.0356 min^−1^ | 0.0410 min^−1^ | 0.2190 min^−1^ |
| SMZ | 0.0080 min^−1^ | 0.0210 min^−1^ | 0.0325 min^−1^ | 0.2621 min^−1^ |
| CIP | 0.0065 min^−1^ | 0.0092 min^−1^ | 0.0223 min^−1^ | 0.1025 min^−1^ |
| CBZ | 0.0009 min^−1^ | 0.0011 min^−1^ | 0.0095 min^−1^ | 0.0207 min^−1^ |
| TC | 0.0108 min^−1^ | 0.0374 min^−1^ | 0.0609 min^−1^ | 1.1028 min^−1^ |

**Supplementary Table 3 |** Molar absorption coefficient of the target compound.

| Chemical | *ε_222_* (M^−1^·cm^−1^) | *ε_254_* (M^−1^·cm^−1^) |
| --- | --- | --- |
| SDZ | 10605 | 21210 |
| PAA | 284 | 44 |
| Br^−^ | 78 | 2 |

**Supplementary Table 4 |** The vertical excitation energy, excitation wavelength, and oscillator strength of Br^−^.

| Excited state | | Vertical excitation energy | Wavelength | Oscillator strengths |
| --- | --- | --- | --- | --- |
|  | S1 | 5.9875 eV | 207.07 nm | f = 0.2035 |
|  | S2 | 5.9875 eV | 207.07 nm | f = 0.2035 |
|  | S3 | 5.9875 eV | 207.07 nm | f = 0.2035 |
|  | S4 | 7.0840 eV | 175.02 nm | f = 0.0000 |
|  | S5 | 7.0840 eV | 175.02 nm | f = 0.0000 |

**Supplementary Table 5 |** The vertical excitation energy, excitation wavelength, and oscillator strength of SDZ.

| Excited state | Vertical excitation energy | Wavelength | Oscillator strengths |
| --- | --- | --- | --- |
| S1 | 4.6800 eV | 264.92 nm | f = 0.0122 |
| S2 | 4.9941 eV | 248.26 nm | f = 0.0916 |
| S3 | 5.1301 eV | 241.68 nm | f = 0.3632 |
| S4 | 5.3016 eV | 233.86 nm | f = 0.1365 |
| S5 | 5.5286 eV | 224.26 nm | f = 0.0032 |
| S6 | 5.7359 eV | 216.15 nm | f = 0.0102 |
| S7 | 6.0301 eV | 205.61 nm | f = 0.2767 |
| S8 | 6.3428 eV | 195.47 nm | f = 0.1523 |
| S9 | 6.3973 eV | 193.81 nm | f = 0.1436 |
| S10 | 6.4902 eV | 191.03 nm | f = 0.0198 |

**Supplementary Table 6 |** The vertical excitation energy, excitation wavelength, and oscillator strength of SDZ+Br^−^ site1.

| Excited state | Vertical excitation energy | Wavelength | Oscillator strengths |
| --- | --- | --- | --- |
| S1 | 4.6948 eV | 264.09 nm | f = 0.0087 |
| S2 | 4.9994 eV | 248.00 nm | f = 0.0472 |
| S3 | 5.1450 eV | 240.98 nm | f = 0.3990 |
| S4 | 5.2586 eV | 235.77 nm | f = 0.1528 |
| S5 | 5.3239 eV | 232.88 nm | f = 0.0031 |
| S6 | 5.4285 eV | 228.39 nm | f = 0.0051 |
| S7 | 5.4707 eV | 226.63 nm | f = 0.0061 |
| S8 | 5.5791 eV | 222.23 nm | f = 0.0018 |
| S9 | 5.8211 eV | 212.99 nm | f = 0.0096 |
| S10 | 5.8309 eV | 212.63 nm | f = 0.0083 |

**Supplementary Table 7 |** The vertical excitation energy, excitation wavelength, and oscillator strength of SDZ+Br^−^ site2.

| Excited state | Vertical excitation energy | Wavelength | Oscillator strengths |
| --- | --- | --- | --- |
| S1 | 4.6191 eV | 268.42 nm | f = 0.0113 |
| S2 | 4.9972 eV | 248.11 nm | f = 0.0656 |
| S3 | 5.1664 eV | 239.98 nm | f = 0.3229 |
| S4 | 5.3088 eV | 233.54 nm | f = 0.1840 |
| S5 | 5.4659 eV | 226.83 nm | f = 0.0053 |
| S6 | 5.7333 eV | 216.25 nm | f = 0.0102 |
| S7 | 5.9069 eV | 209.90 nm | f = 0.0062 |
| S8 | 5.9369 eV | 208.84 nm | f = 0.0142 |
| S9 | 5.9818 eV | 207.27 nm | f = 0.0010 |
| S10 | 6.0124 eV | 206.21 nm | f = 0.2706 |

**Supplementary Table 8 |** The vertical excitation energy, excitation wavelength, and oscillator strength of SDZ+Br^−^ site3.

| Excited state | Vertical excitation energy | Wavelength | Oscillator strengths |
| --- | --- | --- | --- |
| S1 | 4.6768 eV | 265.10 nm | f = 0.0135 |
| S2 | 4.9749 eV | 249.22 nm | f = 0.0941 |
| S3 | 5.1245 eV | 241.94 nm | f = 0.3433 |
| S4 | 5.3028 eV | 233.81 nm | f = 0.1351 |
| S5 | 5.5339 eV | 224.04 nm | f = 0.0033 |
| S6 | 5.6823 eV | 218.19 nm | f = 0.0130 |
| S7 | 5.8166 eV | 213.16 nm | f = 0.0030 |
| S8 | 5.8878 eV | 210.58 nm | f = 0.0065 |
| S9 | 5.8980 eV | 210.21 nm | f = 0.0055 |
| S10 | 6.0032 eV | 206.53 nm | f = 0.2181 |

**Supplementary Table 9 |** Relative parameters of different pollutants.

| Pollutants | HOMO  (eV) | LUMO  (eV) | Band gap  (eV) | Vertical  IP (eV) | Vertical  EA (eV) | Mulliken  Electroneg  -ativity (eV) | Electrophilic  indexes (eV) | Nucleophilic  indexes (eV) |
| --- | --- | --- | --- | --- | --- | --- | --- | --- |
| SDZ | -6.261 | -1.587 | 4.674 | 6.147 | 1.749 | 3.948 | 1.772 | 1.690 |
| SMX | -6.371 | -1.179 | 5.192 | 6.208 | 1.367 | 3.788 | 1.482 | 1.606 |
| SMZ | -6.247 | -1.392 | 4.855 | 6.139 | 1.518 | 3.829 | 1.586 | 1.699 |
| STZ | -6.233 | -1.080 | 5.153 | 6.157 | 0.989 | 3.773 | 1.278 | 1.703 |
| SGD | -6.127 | -0.802 | 5.325 | 6.046 | 0.946 | 3.496 | 1.198 | 1.796 |
| CIP | -6.363 | -1.868 | 4.495 | 6.252 | 2.038 | 3.945 | 2.041 | 2.007 |
| CBZ | -6.391 | -1.729 | 4.662 | 6.288 | 1.855 | 4.071 | 1.870 | 1.696 |
| TC | -6.141 | -2.678 | 3.463 | 5.802 | 2.561 | 4.482 | 2.615 | 1.418 |
| OTC | -6.093 | -2.650 | 3.443 | 5.636 | 2.728 | 4.658 | 2.743 | 1.401 |
| FLO | -7.415 | -1.537 | 5.878 | 7.448 | 1.676 | 4.562 | 1.803 | 0.442 |

Supplementary Table 10 | Fukui index of SDZ.

| Atom | ***f* ^−^** | ***f* ^+^** | ***f* ^0^** | CDD |
| --- | --- | --- | --- | --- |
| C1 | 0.0047 | 0.174 | 0.0893 | 0.1693 |
| C2 | 0.0064 | 0.0782 | 0.0423 | 0.0718 |
| C3 | 0.0048 | 0.1731 | 0.0889 | 0.1682 |
| N4 | 0.0026 | 0.1061 | 0.0544 | 0.1035 |
| C5 | 0.0027 | 0.0494 | 0.0261 | 0.0467 |
| N6 | 0.0057 | 0.1251 | 0.0654 | 0.1195 |
| N7 | 0.015 | 0.0157 | 0.0153 | 0.0008 |
| S8 | 0.0213 | 0.008 | 0.0147 | -0.0133 |
| S9 | 0.0933 | 0.0005 | 0.0469 | -0.0928 |
| C10 | 0.0375 | 0.0038 | 0.0206 | -0.0337 |
| C11 | 0.0975 | 0.0044 | 0.0509 | -0.0931 |
| C12 | 0.0719 | 0.0043 | 0.0381 | -0.0676 |
| C13 | 0.0957 | 0.0045 | 0.0501 | -0.0912 |
| C14 | 0.0399 | 0.0047 | 0.0223 | -0.0353 |
| N15 | 0.1888 | 0.0033 | 0.0961 | -0.1856 |
| H16 | 0.0025 | 0.0741 | 0.0383 | 0.0716 |
| H17 | 0.003 | 0.039 | 0.021 | 0.0360 |
| H18 | 0.0028 | 0.0742 | 0.0385 | 0.0714 |
| H19 | 0.0088 | 0.0182 | 0.0135 | 0.0094 |
| H20 | 0.0223 | 0.0048 | 0.0135 | -0.0175 |
| H21 | 0.0376 | 0.0024 | 0.02 | -0.0353 |
| H22 | 0.0371 | 0.0024 | 0.0197 | -0.0348 |
| H23 | 0.0236 | 0.0028 | 0.0132 | -0.0208 |
| H24 | 0.0608 | 0.0016 | 0.0312 | -0.0592 |
| H25 | 0.0608 | 0.0017 | 0.0312 | -0.0591 |
| O_2_6 | 0.0264 | 0.0118 | 0.0191 | -0.0146 |
| O_2_7 | 0.0268 | 0.0122 | 0.0195 | -0.0146 |

**Supplementary Table 11 |** Bond length and bond order of chemical bonds in SDZ.

| Bond | Bond length (Å) | Bond order |
| --- | --- | --- |
| C1-C2 | 1.391 | 0.979 |
| C1-N6 | 1.333 | 1.013 |
| C1-H16 | 1.087 | 0.846 |
| C2-C3 | 1.388 | 0.963 |
| C2-H17 | 1.082 | 0.818 |
| C3-N4 | 1.335 | 0.988 |
| C3-H18 | 1.087 | 0.860 |
| N4-C5 | 1.331 | 0.941 |
| C5-N6 | 1.335 | 1.037 |
| C5-N7 | 1.393 | 0.702 |
| N7-S8 | 1.682 | 0.563 |
| N7-H19 | 1.016 | 0.688 |
| S8-C9 | 1.754 | 0.718 |
| S8-O26 | 1.459 | 1.117 |
| S8-O27 | 1.453 | 1.127 |
| C9-C10 | 1.396 | 0.621 |
| C9-C14 | 1.397 | 0.633 |
| C10-C11 | 1.383 | 0.813 |
| C10-H20 | 1.084 | 0.893 |
| C11-C12 | 1.409 | 0.773 |
| C11-H21 | 1.086 | 0.879 |
| C12-C13 | 1.410 | 0.770 |
| C12-N15 | 1.373 | 0.733 |
| C13-C14 | 1.382 | 0.820 |
| C13-H22 | 1.086 | 0.894 |
| C14-H23 | 1.086 | 0.879 |
| N15-H24 | 1.011 | 0.747 |
| N15-H25 | 1.011 | 0.747 |

**Supplementary Table 12 |** Acute and chronic toxicity data for SDZ and its degradation intermediates.

| Compound | m/z | **Acute toxicity (**mg·L^−1^**)** | | | **Chronic toxicity (**mg·L^−1^**)** | | |
| --- | --- | --- | --- | --- | --- | --- | --- |
|  |  | Fish  (LC50) | Daphnid (LC50) | Green algae (LC50) | Fish (ChV) | Daphnid (ChV) | Green algae (ChV) |
| SDZ | 251 | 907 | 10 | 40 | 24 | 0.10 | 29 |
| DP1 | 235 | 3650 | 1820 | 787 | 305 | 123 | 154 |
| DP2 | 159 | 152000 | 62800 | 12700 | 10200 | 2550 | 1650 |
| DP3 | 96 | 6270 | 2930 | 970 | 486 | 165 | 164 |
| DP4 | 104 | 72000 | 30200 | 6490 | 4930 | 1280 | 869 |
| DP5 | 99 | 4680 | 2220 | 783 | 370 | 131 | 137 |
| DP6 | 267 | 30000 | 13700 | 4110 | 2270 | 724 | 660 |
| DP7 | 281 | 6340 | 3100 | 1250 | 520 | 200 | 236 |
| DP8 | 273 | 22200 | 10300 | 3280 | 1710 | 567 | 544 |
| DP9 | 245 | 7920 | 3820 | 1440 | 638 | 236 | 262 |
| DP10 | 203 | 1690000 | 706000 | 146000 | 115000 | 29100 | 19300 |
| DP11 | 186 | 2610 | 1310 | 576 | 220 | 90 | 114 |
| DP12 | 200 | 177 | 100 | 74 | 17 | 10 | 19 |
| DP13 | 216 | 64 | 38 | 34 | 7 | 4 | 10 |
| DP14 | 104 | 702000 | 296000 | 63800 | 48200 | 12500 | 8570 |
| DP15 | 120 | 10200000 | 3850000 | 520000 | 615000 | 119000 | 54300 |
| DP16 | 267 | 15700 | 7390 | 2500 | 1230 | 423 | 428 |
| DP17 | 199 | 17800 | 8200 | 2570 | 1360 | 447 | 422 |
| DP18 | 173 | 6590000 | 2570000 | 403000 | 413000 | 87700 | 45600 |
| DP19 | 157 | 601000 | 234000 | 36700 | 37700 | 7990 | 4150 |
| DP20 | 109 | 3380 | 1630 | 622 | 273 | 101 | 114 |
| DP21 | 110 | 114 | 347 | 179 | 59 | 26 | 38 |
| DP22 | 116 | 53300 | 25300 | 8960 | 4220 | 1500 | 1570 |

**Supplementary Table 13 |** DBPs production in different systems.

| Concentration  (μg/L) | UV_222_-PB | UV_222_-PB+NOM | UV_254_-PB+NOM | UV_222_-PB+ASW | UV_222_-PB+YS | UV_222_-PB+XRW |
| --- | --- | --- | --- | --- | --- | --- |
| MBAA | 5.41 | 2.69 | 12.12 | N.D. | N.D. | N.D. |
| DBAA | 244.91 | 242.89 | 263.46 | N.D. | N.D. | N.D. |
| DBCM | N.D. | N.D. | N.D. | 0.00241 | N.D. | N.D. |
| TBM | 0.073 | 0.00373 | N.D. | N.D. | 0.00215 | N.D. |
| BDCA | N.D. | N.D. | N.D. | 1.899 | 1.929 | 1.488 |
| TBAA | N.D. | N.D. | 2.108 | N.D. | N.D. | N.D. |
| TCM | N.D. | N.D. | N.D. | 1.2536 | N.D. | N.D. |

N.D. indicates not detected.

**Supplementary Table 14 |** pH values of solutions at different CO_3_^2−^ concentrations.

| Dosage of CO_3_^2−^ | 0 mM | 0.1 mM | 1 mM | 5 mM | 10 mM |
| --- | --- | --- | --- | --- | --- |
| pH | 7.01 | 7.41 | 7.64 | 9.89 | 10.39 |

**Supplementary Table 15 |** pH values of different mixed anion solutions.

| water sample | TSSW | LSSW | MSSW | HSSW |
| --- | --- | --- | --- | --- |
| pH | 7.39 | 7.65 | 9.85 | 10.31 |

**Supplementary Table 16 |** Water quality parameters of different water environment samples.

| Parameter | XRW | LRW | DLW | YS | ASW |
| --- | --- | --- | --- | --- | --- |
| TOC (mg·L^−1^) | 2.11 | 2.19 | 3.50 | 0.53 | 31.02 |
| COD (mg·L^−1^) | 8 | 11 | 9 | / | 30 |
| BOD_5_ (mg·L^−1^) | < 0.5 | < 0.5 | < 0.5 | < 0.5 | 1.62 |
| Fluoride (mg·L^−1^) | 0.36 | 0.34 | 0.19 | / | / |
| Cl^−^ (mg·L^−1^) | 56.2 | 59.6 | 17.7 | 25367 | 2862 |
| Nitrite (as N) (mg·L^−1^) | 0.51 | 0.59 | 0.16 | / | / |
| Nitrate (as N) (mg·L^−1^) | 4.58 | 4.65 | 1.77 | / | / |
| Br^−^ (mg·L^−1^) | <0.016 | <0.016 | <0.016 | 33.29 | 21.25 |
| pH | 8.35 | 8.24 | 7.40 | 7.23 | 7.39 |
| PO_4_^3−^ (mg·L^−1^) | <0.051 | <0.051 | <0.051 | / | / |
| SO_4_^2−^ (mg·L^−1^) | 38.7 | 38.4 | 35.3 | 1745 | 313 |
| Na^+^ (mg·L^−1^) | 46 | 49.1 | 17.57 | 1125 | 1711 |
| K^+^ (mg·L^−1^) | 10.6 | 11.2 | 3.35 | / | / |
| CO_3_^2−^ (mg·L^−1^) | / | / | / | 53 | / |
| HCO_3_^−^ (mg·L^−1^) | 118 | 115 | 122 | 102 | 107 |

**Supplementary Table 17 |** Life cycle inventory data for 50 mL (1 m^3^) SDZ (20 μM) processed through different manufacturing processes.

|  | UV_222_-PAA | UV_222_-Br | UV_222_-PB | UV_254_-PB | UV_222_-PB (CF) |
| --- | --- | --- | --- | --- | --- |
| Peracetic acid (mg) | 1.901 | / | 1.901 | 1.901 | 638.8 |
| KBr (mg) | / | 2.975 | 2.975 | 2.975 | 999.6 |
| Magnetic stirrer (kW·h) | 0.0139 | 0.0203 | 0.0038 | 0.0085 | / |
| UV radiation source (kW·h) | 0.0163 | 0.0238 | 0.0045 | 0.0067 | 0.216 |
| Reaction box (kW·h) | 0.0488 | 0.0713 | 0.0135 | 0.03 | / |
| Peristaltic Pump (kW·h) | / | / | / | / | 0.27 |
| Infusion Pump (kW·h) | / | / | / | / | 0.36 |

**Supplementary Table 18 |** Reactions for kinetic simulations

| No | Reaction | Rate Constant | Reference |
| --- | --- | --- | --- |
| (1) | CH_3_C(O)OOH + hv → •OH + •CH_3_C(O)O | 1.20 × 10^−4^ s^−1^ | ^8^ |
| (2) | CH_3_C(O)OOH + •OH → •CH_3_C(O)OO + H_2_O | 1.30 × 10^9^ M^−1^·s^−1^ | ^9^ |
| (3) | CH_3_C(O)OOH + •CH_3_C(O)O → •CH_3_C(O)OO + CH_3_C(O)OH | 1.00 × 10^7^ M^−1^·s^−1^ | ^10^ |
| (4) | •CH_3_C(O)OO + •CH_3_C(O)OO → 2•CH_3_C(O)O + O_2_ | 8.30 × 10^9^ M^−1^·s^−1^ | ^11^ |
| (5) | •CH_3_C(O)O + •CH_3_C(O)O → (CH_3_C(O)O)_2_ | 1.00 × 10^9^ M^−1^·s^−1^ | ^12^ |
| (6) | •CH_3_C(O)O → •CH_3_ + CO_2_ | 2.30 × 10^5^ s^−1^ | ^12^ |
| (7) | •CH_3_ + O_2_ → •CH_3_O_2_ | 4.70 × 10^9^ M^−1^·s^−1^ | ^13^ |
| (8) | H_2_O_2_ + hv → 2•OH | 8.80 × 10^−6^ s^−1^ | ^14^ |
| (9) | H_2_O_2_ → HO_2_^−^ + H^+^ | 1.26 × 10^−1^ s^−1^ | ^15^ |
| (10) | HO_2_^−^ + H^+^ → H_2_O_2_ | 5.00 × 10^10^ M^−1^·s^−1^ | ^15^ |
| (11) | H_2_O_2_ + •OH → •HO_2_ + H_2_O | 2.70 × 10^7^ M^−1^·s^−1^ | ^16^ |
| (12) | •OH + •OH → H_2_O_2_ | 5.50 × 10^9^ M^−1^·s^−1^ | ^16^ |
| (13) | •HO_2_ + •HO_2_ → H_2_O_2_ + O_2_ | 8.60 × 10^5^ M^−1^·s^−1^ | ^17^ |
| (14) | CH_3_C(O)OOH + •HO_2_ → •CH_3_C(O)OO + H_2_O_2_ | 2.00 × 10^2^ M^−1^·s^−1^ | ^9^ |
| (15) | •CH_3_C(O)OO → •HO_2_ + CH_2_CO | 1.82 s^−1^ | ^11^ |
| (16) | CH_2_CO + H_2_O → CH_3_C(O)OH | 44 s^−1^ | ^11^ |
| (17) | CH_3_C(O)OOH + Br^−^ → HOBr + CH_3_COO^−^ + H_2_O | 2.40 × 10^−1^ M^−1^·s^−1^ | ^18^ |
| (18) | HOBr + H_2_O_2_ → Br^−^ + O_2_ + H_2_O | 1.50 × 10^4^ M^−1^·s^−1^ | ^15^ |
| (19) | HOBr + HO_2_^−^ → Br^−^ + O_2_ + H_2_O | 7.60 × 10^8^ M^−1^·s^−1^ | ^18^ |
| (20) | OBr^−^ + H_2_O_2_ → Br^−^ + O_2_ + H_2_O | 1.20 × 10^6^ M^−1^·s^−1^ | ^15^ |
| (21) | Br^−^ + HOBr + H^+^ → Br_2_ + H_2_O | 1.60 × 10^10^ M^−2^·s^−1^ | ^15^ |
| (22) | Br_2_ + H_2_O → Br^−^ + HOBr + H^+^ | 1.10 × 10^2^ s^−1^ | ^15^ |
| (23) | 2HOBr → Br_2_O + H_2_O | 2.40 × 10^−2^ M^−1^·s^−1^ | ^15^ |
| (24) | Br_2_O + H_2_O → 2HOBr | 3.90 × 10^−3^ s^−1^ | ^15^ |
| (25) | Br^−^ + hv → •Br | 2.00 × 10^−5^ s^−1^ | This work |
| (26) | HOBr + hv → •Br + •OH | 1.00 × 10^−5^ s^−1^ | This work |
| (27) | •OH + Br^−^ → •BrOH^−^ | 1.10 × 10^10^ M^−1^·s^−1^ | ^19^ |
| (28) | •BrOH^−^ → •OH + Br^−^ | 4.20 × 10^6^ s^−1^ | ^19^ |
| (29) | •BrOH^−^ + H^+^ → •Br + H_2_O | 4.40 × 10^10^ M^−1^·s^−1^ | ^19^ |
| (30) | •BrOH^−^ + Br^−^ → •Br_2_^−^ + OH^−^ | 1.90 × 10^8^ M^−1^·s^−1^ | ^19^ |
| (31) | •Br + H_2_O_2_ → •HO_2_ + Br^−^ + H^+^ | 4.00 × 10^9^ M^−1^·s^−1^ | ^19^ |
| (32) | •Br + •HO_2_ → O_2_ + Br^−^ + H^+^ | 1.60 × 10^8^ M^−1^·s^−1^ | ^19^ |
| (33) | •Br + Br^−^ → •Br_2_^−^ | 1.20 × 10^10^ M^−1^·s^−1^ | ^19^ |
| (34) | •Br_2_^−^+ •OH → HOBr + Br^−^ | 1.00 × 10^9^ M^−1^·s^−1^ | ^20^ |
| (35) | CH_3_C(O)OOH + Cl^−^ → HOCl + CH_3_COO^−^ + H_2_O | 1.47 × 10^−5^ M^−1^·s^−1^ | ^18^ |
| (36) | HOCl + H_2_O_2_ → Cl^−^ + O_2_ + H_2_O | 1.96 × 10^2^ M^−1^·s^−1^ | ^15^ |
| (37) | HOCl + HO_2_^−^ → Cl^−^ + O_2_ + H_2_O | 4.40 × 10^7^ M^−1^·s^−1^ | ^15^ |
| (38) | OCl^−^ + H_2_O_2_ → Cl^−^ + O_2_ + H_2_O | 3.30 × 10^3^ M^−1^·s^−1^ | ^15^ |
| (39) | HOCl + Br^−^ → HOBr + Cl^−^ | 1.55 × 10^3^ M^−1^·s^−1^ | ^15^ |
| (40) | OCl^−^ + Br^−^ → OBr^−^ + Cl^−^ | 9.00 × 10^−4^ M^−1^·s^−1^ | ^15^ |
| (41) | Cl^−^ + HOCl + H^+^ → Cl_2_ + H_2_O | 1.82 × 10^−1^ M^−2^·s^−1^ | ^15^ |
| (42) | Cl_2_ + H_2_O → Cl^−^ + HOCl + H^+^ | 4.01 × 10^−2^ s^−1^ | ^15^ |
| (43) | 2HOCl → Cl_2_O + H_2_O | 8.22 × 10^−1^ M^−1^·s^−1^ | ^15^ |
| (44) | Cl_2_O + H_2_O → 2HOCl | 7.15 × 10^−3^ s^−1^ | ^15^ |
| (45) | Cl^−^ + HOBr + H^+^ → BrCl + H_2_O | 2.50 × 10^10^ M^−2^·s^−1^ | ^15^ |
| (46) | BrCl + H_2_O → Cl^−^ + HOBr + H^+^ | 3.00 × 10^6^ s^−1^ | ^15^ |
| (47) | HOBr + HOCl → BrOCl + H_2_O | 3.60 × 10^−4^ M^−1^·s^−1^ | ^15^ |
| (48) | BrOCl + H_2_O → HOBr + HOCl | 1.00 × 10^−3^ s^−1^ | ^15^ |
| (49) | HOCl + hv → •Cl + •OH | 2.82 × 10^−5^ s^−1^ | This work |
| (50) | •ClOH^−^ + H^+^ → •Cl + H_2_O | 2.10 × 10^10^ M^−1^·s^−1^ | ^21^ |
| (51) | •OH + Cl^−^ → •ClOH^−^ | 4.30 × 10^9^ M^−1^·s^−1^ | ^19^ |
| (52) | •ClOH^−^ → •OH + Cl^−^ | 6.10 × 10^9^ s^−1^ | ^19^ |
| (53) | •ClOH^−^ + Cl^−^ → •Cl_2_^−^ + OH^−^ | 1.00 × 10^5^ M^−1^·s^−1^ | ^21^ |
| (54) | •Cl + Cl^−^ → •Cl_2_^−^ | 6.5 × 10^9^ M^−1^·s^−1^ | ^21^ |
| (55) | •Cl + •Cl → Cl_2_ | 8.8 × 10^7^ M^−1^·s^−1^ | ^8^ |
| (56) | •Cl + Br^−^ → •BrCl^−^ | 1.20 × 10^10^ M^−1^·s^−1^ | ^22^ |
| (57) | •BrCl^−^ → •Cl + Br^−^ | 2.00 × 10^3^ s^−1^ | ^22^ |
| (58) | •Cl_2_^−^ + Br^−^ → •BrCl^−^ + Cl^−^ | 4.00 × 10^9^ M^−1^·s^−1^ | ^22^ |
| (59) | •BrCl^−^ + Cl^−^ → •Cl_2_^−^ + Br^−^ | 1.10 × 10^2^ M^−1^·s^−1^ | ^22^ |
| (60) | •Br + Cl^−^ → •BrCl^−^ | 1.00 × 10^8^ M^−1^·s^−1^ | ^22^ |
| (61) | •BrCl^−^ → •Br + Cl^−^ | 6.10 × 10^4^ s^−1^ | ^22^ |
| (62) | •Br_2_^−^ + Cl^−^ → •BrCl^−^ + Br^−^ | 4.30 × 10^6^ M^−1^·s^−1^ | ^22^ |
| (63) | •BrCl^−^ + Br^−^ → •Br_2_^−^ + Cl^−^ | 8.00 × 10^9^ M^−1^·s^−1^ | ^22^ |
| (64) | •Br_2_^−^ + •Cl_2_^−^ → Br_2_ + 2Cl^−^ | 4.00 × 10^9^ M^−1^·s^−1^ | ^22^ |
| (65) | •BrCl^−^ + •OH → BrCl + OH^−^ | 1.00 × 10^9^ M^−1^·s^−1^ | ^19^ |
| (66) | •BrCl^−^ + •HO_2_ → BrCl + O_2_ + H^+^ | 1.00 × 10^9^ M^−1^·s^−1^ | ^19^ |
| (67) | •BrCl^−^ + H_2_O_2_ → Br^−^ + Cl^−^ + HO_2_ | 5.00 × 10^3^ M^−1^·s^−1^ | ^19^ |
| (68) | •BrCl^−^ + •Cl_2_^−^ → BrCl + 2Cl^−^ | 2.00 × 10^9^ M^−1^·s^−1^ | ^19^ |
| (69) | •BrCl^−^ + •Br_2_^−^ → BrCl + 2Br^−^ | 4.00 × 10^9^ M^−1^·s^−1^ | ^19^ |
| (70) | •BrCl^−^ + •BrCl^−^ → BrCl + Br^−^ + Cl^−^ | 4.70 × 10^9^ M^−1^·s^−1^ | ^22^ |
| (71) | •BrCl^−^ + OH^−^ → •BrOH^−^ + Cl^−^ | 2.00 × 10^7^ M^−1^·s^−1^ | ^22^ |
| (72) | •BrCl^−^ + OH^−^ → Br^−^ + •ClOH^−^ | 3.00 × 10^6^ M^−1^·s^−1^ | ^19^ |
| (73) | •BrOH^−^ + Cl^−^ → •BrCl^−^ + OH^−^ | 1.90 × 10^8^ M^−1^·s^−1^ | ^19^ |
| (74) | •ClOH^−^ + Br^−^ → •BrCl^−^ + OH^−^ | 1.00 × 10^9^ M^−1^·s^−1^ | ^19^ |
| (75) | BrCl + •HO_2_ → •BrCl^−^ + O_2_ + H^+^ | 5.00 × 10^8^ M^−1^·s^−1^ | ^20^ |
| (76) | BrCl + H_2_O_2_ → HOBr + Cl^−^ + H^+^ + O_2_ | 1.30 × 10^4^ M^−1^·s^−1^ | ^20^ |
| (77) | SDZ + hv (222 nm) → products | 4.17 × 10^−5^ s^−1^ | This work |
| (78) | SDZ + CH_3_C(O)OOH → products | 5.01 × 10^−9^ M^−1^·s^−1^ | This work |
| (79) | SDZ + OH → products | 2.51 × 10^9^ M^−1^·s^−1^ | This work |
| (80) | SDZ + •CH_3_C(O)O → products | 1.78 × 10^6^ M^−1^·s^−1^ | This work |
| (81) | SDZ + •CH_3_C(O)OO → products | 2.51 × 10^5^ M^−1^·s^−1^ | This work |
| (82) | SDZ + •Br → products | 4.28 × 10^10^ M^−1^·s^−1^ | This work |
| (83) | SDZ + •Br_2_^−^ → products | 1.53 × 10^8^ M^−1^·s^−1^ | This work |
| (84) | SDZ + •BrOH^−^ → products | 2.82 × 10^9^ M^−1^·s^−1^ | This work |
| (85) | SDZ + HOBr → products | 1.78 × 10^5^ M^−1^·s^−1^ | This work |
| (86) | SDZ + Br_2_ → products | 1.78 × 10^7^ M^−1^·s^−1^ | This work |
| (87) | SDZ + Br_2_O → products | 1.00 × 10^7^ M^−1^·s^−1^ | This work |
| (88) | SDZ + HOCl → products | 5.62 × 10^4^ M^−1^·s^−1^ | This work |
| (89) | SDZ + Cl_2_ → products | 7.08 × 10^6^ M^−1^·s^−1^ | This work |
| (90) | SDZ + Cl_2_O → products | 1.00 × 10^6^ M^−1^·s^−1^ | This work |
| (91) | SDZ + •Cl → products | 2.51 × 10^8^ M^−1^·s^−1^ | This work |
| (92) | SDZ + •Cl_2_^−^ → products | 1.42 × 10^7^ M^−1^·s^−1^ | This work |
| (93) | SDZ + •ClOH^−^ → products | 5.01 × 10^8^ M^−1^·s^−1^ | This work |
| (94) | SDZ + BrCl → products | 7.06 × 10^2^ M^−1^·s^−1^ | This work |
| (95) | SDZ + •BrCl^−^ → products | 1.78 × 10^8^ M^−1^·s^−1^ | This work |
| (96) | SDZ + BrOCl → products | 1.00 × 10^6^ M^−1^·s^−1^ | This work |
| (97) | SDZ + H_2_O_2_ → products | 1.00 × 10^−6^ M^−1^·s^−1^ | This work |
| (98) | SDZ + •HO_2_ → products | 1.78 × 10^3^ M^−1^·s^−1^ | This work |

**3. Supplementary Figures**

**Supplementary Fig. 1 |** **Degradation of SDZ under dark reaction conditions.** (a) Degradation of SDZ in different systems under dark conditions. (b) Pseudo-first-order kinetic fitting curves and pseudo-first-order rate constant (*k*_obs_) values for SDZ degradation in the Dark-PB system. [SDZ]_0_ = 20 μM, pH = 7.0, [PAA]_0_ = [Br]_0_ = 0.5 mM, [H_2_O_2_]_0_ = 0.2 mM. Error bars represent SD of three replicate experiments, and the central value is the mean of the data. Data are presented as mean values +/- SD.

**Supplementary Fig. 2 |** **SDZ degradation kinetics and species contributions in UV_222_-PB.** Pseudo-first-order kinetic fitting curves of SDZ degradation in (a) UV_222_-PAA, (b) UV_222_-Br and (c) UV_222_-PB processes. (d) Relative contributions of direct photolysis and oxidative degradation in the UV_222_-PB system. Conditions: [SDZ]_0_ = 20 uM, pH = 7.0, [PAA]_0_ = 0.1 mM-1 mM, [Br]_0_ = 5 μM-1 mM. Error bars represent SD of three replicate experiments, and the central value is the mean of the data. Data are presented as mean values +/- SD.

**Supplementary Fig. 3 |** **Role of H_2_O_2_ in SDZ degradation.** (a) SDZ degradation curve and (b) pseudo first-order kinetic fitting curve of different processes under UV_222_. (c) Degradation curves of SDZ at different H_2_O_2_ concentrations, (d) pseudo-first-order kinetic fitting curves, and (e) pseudo-first-order rate constant (*k*_obs_) values. (f) Degradation curves of SDZ in the presence of catalase (CAT), (g) pseudo-first-order kinetic fitting curves, and (h) *k*_obs_ values. (i) Dissolved oxygen variations with and without CAT. Conditions: [SDZ]_0_ = 20 μM, pH = 7.0, [CAT]_0_ = 20 units·mL^−1^, [PAA]_0_ = [Br]_0_ = 0.5 mM. Error bars represent SD of three replicate experiments, and the central value is the mean of the data. Data are presented as mean values +/- SD.

**Supplementary Fig. 4 |** **Degradation of organic micropollutants (OMPs) under different UV_222_-based processes.** (a, f) sulfadiazine (SDZ), (b, g) sulfamethazine (SMZ), (c, h) ciprofloxacin (CIP), (d, i) carbamazepine (CBZ), and (e, j) tetracycline hydrochloride (TC) degradation curves and pseudo-first-order kinetic fitting curves under different UV_222_ processes. Conditions: [OMPs]_0_ = 20 uM, pH = 7.0, [PAA]_0_ = [Br]_0_ = 0.5 mM. Error bars represent SD of three replicate experiments, and the central value is the mean of the data. Data are presented as mean values +/- SD.

**Supplementary Fig. 5 |** **Degradation of SDZ under different wavelengths.** Degradation curves and pseudo-first-order kinetic fitting curves of SDZ under irradiation at (a, d) UV_222_, (b, e) UV_254_, (c, f) and UV_365_. (g) Fluence-based pseudo-first-order rate constant (*k*′_obs_) values for different processes under UV_365_. (h) Molar absorptivity (ε) and quantum yield (Φ) of SDZ at 222 and 254 nm. Conditions: [SDZ]_0_ = 20 uM, pH = 7.0, [PAA]_0_ = [Br]_0_ = 0.5 mM. Error bars represent SD of three replicate experiments, and the central value is the mean of the data. Data are presented as mean values +/- SD.

**Supplementary Fig. 6 |** The UV-vis spectra of SDZ、Br^−^ and PAA.

**Supplementary Fig. 7 |** **Interaction mechanism between SDZ and Br^−^.** Oscillator strengths and molar absorption coefficients (ε) for (a) Br^−^, (b) SDZ, (c) SDZ+Br^−^ site1, (d) SDZ+Br^−^ site2, and (e) SDZ+Br^−^ site3. (f) Gibbs free energy changes for the reaction of SDZ with Br^−^ under different electronic states. (g) Highest occupied molecular orbital (HOMO) and lowest unoccupied molecular orbital (LUMO) of SDZ and Br^−^ at different complexation sites.

**Supplementary Fig. 8 |** **Assessment of reactive oxygen species roles in the UV_222_-PB system.** (a) Evaluation of interference experiments and (b) absorption spectra for different quenchers at a concentration of 100 mM. Pseudo-first-order kinetic fitting curves of SDZ degradation in the UV_222_-PB system at different (c) tert-butanol (TBA), (d) solvents, (e) superoxide dismutase (SOD) concentrations. Conditions: [SDZ]_0_ = 20 uM, pH = 7.0, [PAA]_0_ = [Br]_0_ = 0.5 mM. Error bars represent SD of three replicate experiments, and the central value is the mean of the data. Data are presented as mean values +/- SD.

**Supplementary Fig. 9 | Assessment of •OH generation in different systems.** (a) Degradation of benzoic acid (BA) and (b) formation of p-hydroxybenzoic acid (p-HBA) in different systems under UV_222_ irradiation. Conditions: [SDZ]_0_ = 20 uM, pH = 7.0, [PAA]_0_ = [Br]_0_ = 0.5 mM. Error bars represent SD of three replicate experiments, and the central value is the mean of the data. Data are presented as mean values +/- SD.

**Supplementary Fig. 10 | Assessment of •O_2_^−^ generation in different systems.** The UV-vis absorption spectrum of nitrotetrazolium blue chloride (NBT) in (a) UV_222_-PAA, (b) UV_222_-Br and (c) UV_222_-PB systems. Conditions: [NBT]_0_ = 50 uM, pH = 7.0, [PAA]_0_ = [Br]_0_ = 0.5 mM.

**Supplementary Fig. 11 |** **Electron spin resonance (ESR) spectra of reactive oxygen species in different processes.** ESR spectra obtained by using (a) 2,2,6,6-Tetramethylpiperidine (TEMP) and (b) 5,5-Dimethyl-1-pyrroline N-oxide (DMPO) as spin trapping agents in different processes.

**Supplementary Fig. 12 |** **Assessment of the role of HOBr in the UV_222_-PB system.** (a) Degradation curve and (b) pseudo-first-order kinetic fitting curve of SDZ at different concentrations of (NH_4_)_2_SO_4_ in UV_222_-PB system. The inset shows the rate constant derived from the pseudo-first-order kinetic fitting. Conditions: [SDZ]_0_ = 20 uM, pH = 7.0, [PAA]_0_ = [Br]_0_ = 0.5 mM, [(NH_4_)_2_SO_4_]_0_ = 10 mM-100 mM. Error bars represent SD of three replicate experiments, and the central value is the mean of the data. Data are presented as mean values +/- SD.

**Supplementary Fig. 13 | LC-MS analysis of HOBr formation at different reaction times.** HOBr formation in the UV_222_-Br system after (a) 10 minutes, (b) 20 minutes, and (c) 30 minutes of reaction. HOBr formation in the UV_222_-PB system after (d) 5 minutes, (e) 10 minutes, (f) 15 minutes, (g) 20 minutes, and (h) 30 minutes of reaction.

**Supplementary Fig. 14 |** **Br^−^ concentration changes and total organic bromine (****TOBr) formation in different systems.** (a) Concentration changes of Br^−^ in the UV_222_-Br and (b) UV_222_-PB systems. (c) Concentrations of TOBr generated during the UV_222_-PB process at different time points. (d) Ion chromatograms (IC) of Br^−^ in the UV_222_-Br and UV_222_-PB systems at different time points, and standard curve of Br^−^ under IC measurement.

**Supplementary Fig. 15 |** **Assessment of Mn^2+^ effects and quenching experiments.** (a) Concentration changes of 100 mM Mn^2+^ under different processes. (b) Degradation of SDZ by 100 mM Mn^2+^. Concentration changes of Br^−^ in the system of (c) 100 mM Mn^2+^, (d) UV_222_-PB (without SDZ), and (e) UV_222_-PB (without SDZ) + 100 mM Mn^2+^ (Br^−^ concentration diluted 30 times). (f) The effect of 100 mM Mn^2+^ on the degradation of SDZ in the UV_222_ system. (g) Pseudo first-order kinetic fitting curve of SDZ at different concentrations of Mn^2+^. Conditions: [SDZ]_0_ = 20 uM, pH = 7.0, [PAA]_0_ = [Br]_0_ = 0.5 mM, [Mn^2+^]_0_ = 10 mM-100 mM. Error bars represent SD of three replicate experiments, and the central value is the mean of the data. Data are presented as mean values +/- SD.

**Supplementary Fig. 16 |** Ion chromatograms of Br^−^ at different time points for 100 mM Mn^2+^, UV_222_-PB (without SDZ), and UV_222_-PB (without SDZ) + 100 mM Mn^2+^ (Br^−^ concentration diluted 30 times).

**Supplementary Fig. 17 |** **Kinetic modeling and cycling mechanisms of the UV_222_-PB system.** (a–c) Degradation model curves of the UV_222_-PB process. (d) Quasi-steady concentrations of different reactive species, (e) reaction rate curves, and (f) species concentration curves in the model. (g) Simulated curves of SDZ degradation under different pathway deactivation conditions in the UV_222_-PB system. (h–j) Sensitivity curves for SDZ degradation via different reaction pathways in the UV_222_-PB system, and (k) sensitivity coefficients. (l) Concentration variations of PAA, H_2_O_2_, and Br^−^. symbols represent experimental data, dashed lines represent model predictions.

**Supplementary Fig. 18 |** **Targeted digestion of organic micropollutants (OMPs) and kinetic coupling of molecular parameters.** (a) Degradation of different OMPs in the UV_222_ system. Pseudo first-order kinetic fitting curve of different OMPs in the (b) UV_222_ and (c) UV_222_-PB system. Correlation analysis of ln(*k*_obs_) after separation of 10 different OMPs with (d) Vertical EA and (e) Mulliken electronegativity. The selected OMPs include sulfadiazine (SDZ), sulfamethoxazole (SMX), sulfamethazine (SMZ), sulfathiazole (STZ), sulfaguanidine (SGD), ciprofloxacin (CIP), carbamazepine (CBZ), tetracycline hydrochloride (TC), oxytetracycline hydrochloride (OTC), and florfenicol (FLO). Conditions: [OMPs]_0_ = 20 uM, pH = 7.0, [PAA]_0_ = [Br]_0_ = 0.5 mM. Error bars represent SD of three replicate experiments, and the central value is the mean of the data. Data are presented as mean values +/- SD.

Supplementary Fig. 19 | Formation of degradation products of SDZ in the UV_222_-PB system. LC-MS analysis of SDZ in the UV_222_-PB system at (a) 10 min, (b) 20 min, and (c) 30 min.

**Supplementary Fig. 20 |** Digital images of wheat seeds growing continuously for 120 h under different treatment solutions.

**Supplementary Fig. 21 |** Digital images of amaranth seeds after 48 h of growth under different treatment solutions.

**Supplementary Fig. 22 |** Violin plot of shoot length distribution for amaranth seeds after 48 h of growth under different treatment solutions.

**Supplementary Fig. 23 |** **Detection of BrO_3_^−^ formation in different processes by ion chromatograms.** (a) BrO_3_^−^ standard sample detected by IC. (b) Ion chromatograms of BrO_3_^−^ in the UV_254_-Br, UV_254_-PB, UV_222_-Br, UV_222_-PB systems at different time points.

**Supplementary Fig. 24 |** **Effect of pH on SDZ degradation kinetics in the UV_222_-Br system.** Degradation curves and pseudo-first-order kinetic curves of SDZ in the UV_222_-Br system at (a−b) pH 3, (c−d) pH 6, (e−f) pH 7, (g−h) pH 8, (i−j) pH 9, and (k−l) pH 11. Conditions: [SDZ]_0_ = 20 uM, [Br]_0_ = 0.5 mM, pH = 3.0-11.0. Error bars represent SD of three replicate experiments, and the central value is the mean of the data. Data are presented as mean values +/- SD.

**Supplementary Fig. 25 |** Effect of pH on the existence form of SDZ.

**Supplementary Fig. 26 |** **Molecular structures and frontier orbitals of SDZ species.** (a) Highest occupied molecular orbital (HOMO) and lowest unoccupied molecular orbital (LUMO), (b) molecular structures of SDZ under different existing forms.

**Supplementary Fig. 27 |** **•OH generation in the UV_222_-Br system at different pH.** Under different pH values in the UV_222_-Br process, (a) degradation of benzoic acid (BA), (b) the generated concentration of p-hydroxybenzoic acid (p-HBA), and (c) the cumulative concentration of •OH. Error bars represent SD of three replicate experiments, and the central value is the mean of the data. Data are presented as mean values +/- SD.

**Supplementary Fig. 28 | Effect of pH on SDZ degradation kinetics in the UV_222_-PB system.** Degradation curves and pseudo-first-order kinetic curves of SDZ in the UV_222_-PB system at (a−b) pH 3, (c−d) pH 6, (e−f) pH 7, (g−h) pH 8, (i−j) pH 9, and (k−l) pH 11. Conditions: [SDZ]_0_ = 20 uM, [PAA]_0_ = [Br]_0_ = 0.5 mM, pH = 3.0-11.0. Error bars represent SD of three replicate experiments, and the central value is the mean of the data. Data are presented as mean values +/- SD.

**Supplementary Fig. 29 |** **Effects of inorganic anions on SDZ degradation kinetics in UV_222_-based systems.** Pseudo-first-order kinetic fitting curves of SDZ under different concentrations of (a) Cl^−^, (b) SO_4_^2−^, (c) NO_3_^−^, and (d) CO_3_^2−^ in the UV_222_-PB system. Degradation curves and pseudo-first-order kinetic fitting curves of SDZ under different NO_3_^−^ concentrations in the (e−f) UV_222_, (g−h) UV_222_-Br, and (i−j) UV_222_-PAA systems. Conditions: [SDZ]_0_ = 20 μM, [PAA]_0_ = [Br]_0_ = 0.5 mM, pH = 7.0. Error bars represent SD of three replicate experiments, and the central value is the mean of the data. Data are presented as mean values +/- SD.

**Supplementary Fig. 30 |** **Performance of the UV_222_-PB system in complex water matrices.** (a) Pseudo-first-order kinetic fitting curves for different simulated salinity water bodies in the UV_222_-PB system (TSSW: trace‑salinity simulated water, LSSW: low‑salinity simulated water, MSSW: medium‑salinity simulated water, HSSW: high‑salinity simulated water). Pseudo-first-order kinetic fitting curves of SDZ under different concentrations of (b) Suwannee River III HA (SRHA) and (c) Suwannee River III FA (SRFA) in the UV_222_-PB system. Conditions: [SDZ]_0_ = 20 μM, [PAA]_0_ = [Br]_0_ = 0.5 mM. Error bars represent SD of three replicate experiments, and the central value is the mean of the data. Data are presented as mean values +/- SD.

**Supplementary Fig. 31** **| Degradation of SDZ in real water matrices and under high chloride concentrations.** (a) Pseudo-first-order kinetic curves of SDZ under the UV_222_-PB process in different real water bodies (DW: Deionized Wate, XRW: Xiangjiang River water, LRW: Liuyang River water, DLW: Dongting Lake water, YS: Yellow Sea, ASW: Aquaculture Seawater Wastewater). (b) Pseudo-first-order kinetic curves of SDZ under different processes with UV_222_. (c) Pseudo-first-order kinetic curves, (d) degradation curves, and (e) pseudo-first-order rate constant (*k*_obs_) values for SDZ under different processing conditions in the dark. [SDZ]_0_ = 20 μM, [PAA]_0_ = [Br]_0_ = 0.5 mM, [Cl]_0_ = 0.5 M. Error bars represent SD of three replicate experiments, and the central value is the mean of the data. Data are presented as mean values +/- SD.

**Supplementary Fig. 32 | Kinetic modeling of the UV_222_-PBC system.** (a) Degradation model curves for different systems in the UV_222_-PBC process. (b) Degradation simulation curves of UV_222_-PB at different Cl⁻ concentrations. (c) Quasi-steady concentrations of different reactive species, (d) reaction rate curves, and (e) species concentration curves in the model. (f) Predicted curves and (g) residual concentrations of PAA, H_2_O_2_ and Br^−^.

**Supplementary Fig. 33 |** **Degradation kinetics of multiple organic micropollutants under UV_222_-based processes.** (a, f) sulfadiazine (SDZ), (b, g) sulfamethazine (SMZ), (c, h) ciprofloxacin (CIP), (d, i) carbamazepine (CBZ), and (e, j) tetracycline hydrochloride (TC) degradation curves and pseudo-first-order kinetic fitting curves under different UV_222_ processes. Conditions: [OMPs]_0_ = 20 uM, pH = 7.0, [PAA]_0_ = [Br]_0_ = 0.5 Mm, [Cl]_0_ = 0.5 M. Error bars represent SD of three replicate experiments, and the central value is the mean of the data. Data are presented as mean values +/- SD.

**Supplementary Fig. 34 |** Actual digital images of the continuous-flow apparatus.

**Supplementary Fig. 35 |** Pseudo-first-order kinetic fit curves and pseudo-first-order rate constant (*k*_obs_) values for the UV_222_-PB continuous-flow (UV_222_-PB (CF)) system.

**Supplementary Fig. 36 |** Removing the SDZ from the boundaries of life-cycle assessment systems across different systems.

**Supplementary Fig. 37 |** Comparison of environmental impacts in the life cycle assessment of UV_222_-PAA, UV_222_-Br, UV_222_-PB, and UV_254_-PB systems. TAP, Terrestrial acidification potential; GWP, Global warming potential; FETP, Freshwater ecotoxicity potential; METP, Marine ecotoxicity potential; TETP, Terrestrial ecotoxicity potential; FFP, Fossil fuel potential; FEP, Freshwater eutrophication potential; MEP, Marine eutrophication potential; HTPc, Human toxicity potential (carcinogenic); HTPnc, Human toxicity potential (non-carcinogenic); IRP, Ionising radiation potential; LOP, Agricultural land occupation; SOP, Surplus ore potential; ODP, Ozone depletion potential; PMFP, Particulate matter formation potential; HOFP, Photochemical oxidant formation potential (humans); EOFP, Photochemical oxidant formation potential (ecosystems); and WCP, Water consumption potential.

**Supplementary Fig. 38 |** Comparison of environmental impacts in life cycle assessments of different processes. TAP, Terrestrial acidification potential; GWP, Global warming potential; FETP, Freshwater ecotoxicity potential; METP, Marine ecotoxicity potential; TETP, Terrestrial ecotoxicity potential; FFP, Fossil fuel potential; FEP, Freshwater eutrophication potential; MEP, Marine eutrophication potential; HTPc, Human toxicity potential (carcinogenic); HTPnc, Human toxicity potential (non-carcinogenic); IRP, Ionising radiation potential; LOP, Agricultural land occupation; SOP, Surplus ore potential; ODP, Ozone depletion potential; PMFP, Particulate matter formation potential; HOFP, Photochemical oxidant formation potential (humans); EOFP, Photochemical oxidant formation potential (ecosystems); and WCP, Water consumption potential.

**Supplementary Fig. 39** **|** Comparison of the environmental impacts of UV_222_-PB (CF) in life cycle assessments for 2025 and 2060.

**Supplementary Fig. 40 |** Schematic diagram of irradiation experiments using different light sources.

**Supplementary Fig. 41 |** **Emission spectra of different light sources**. Emission spectra of (a) KrCl* excimer lamp (UV_222_), (b) low-pressure ultraviolet lamp (UV_254_), and (c) medium-pressure ultraviolet lamp (UV_365_).

Supplementary References

1. Bolton, J. R.; Linden, K. G. Standardization of Methods for Fluence (UV Dose) Determination in Bench-Scale UV Experiments. *J. Environ. Eng.* **129**, 209–215 (2003).
2. Chen, L. *et al.* Accurate identification and formation mechanism unraveling of radicals in UV-induced peracetic acid activation system using in-situ electron paramagnetic resonance. *Sci. Bull.* **70**, 1581-1585 (2025).
3. Guo, K. et al. Comparison of the UV/chlorine and UV/H_2_O_2_ processes in the degradation of PPCPs in simulated drinking water and wastewater: Kinetics, radical mechanism and energy requirements. *Water Res.* **147**, 184-194 (2018).
4. Guo, K., Wei, W., Wu, S., Song, W. & Fang, J. Abatement of Structurally Diverse Micropollutants by the UV/Permanganate Process: Roles of Hydroxyl Radicals and Reactive Manganese Species. *ACS EST Water.* **2**, 593-603 (2022).
5. Luo, Y. *et al.* Critical role of dissolved oxygen and iron–copper synergy in dual-metal/char catalyst systems. *Environ. Sci.: Nano* **11**, 2091-2102 (2024).
6. Li, Z. *et al.* Catalytic wastewater dephosphorization for phosphorus recycling to fertilizers. *Appl. Catal. B-Environ. Energy.* **369**, 125154 (2025).
7. Xiao, J. *et al.* Simultaneous spectrophotometric determination of peracetic acid and the coexistent hydrogen peroxide using potassium iodide as the indicator. *Anal. Methods-UK.* **11**, 1930-1938 (2019).
8. Gao, Y. *et al.* Impact of water matrices on micropollutant oxidation by Far-UVC/peracetic acid versus UV_254_/peracetic acid processes: Kinetics modeling and mechanism. *Water Res.* **289**, 124830 (2026).
9. Zhang, T. & Huang, C.-H. Modeling the Kinetics of UV/Peracetic Acid Advanced Oxidation Process. *Environ. Sci. Technol .***54**, 7579-7590 (2020).
10. Chateauneuf, J., Lusztky, J. & Ingold, K. U. Spectroscopic and Kinetic Characteristics of Aroyloxyl Radicals. 1. The 4-Methoxybenzoyloxyl Radical. *J. Am. Chem. Soc.* **110**, 2878-2887 (1988).
11. Kamath, D., Mezyk, S. P. & Minakata, D. Elucidating the Elementary Reaction Pathways and Kinetics of Hydroxyl Radical-Induced Acetone Degradation in Aqueous Phase Advanced Oxidation Processes. *Environ. Sci. Technol.* **52**, 7763-7774 (2018).
12. Liu, T. *et al.* Unexpected Role of Nitrite in Promoting Transformation of Sulfonamide Antibiotics by Peracetic Acid: Reactive Nitrogen Species Contribution and Harmful Disinfection Byproduct Formation Potential. *Environ. Sci. Technol.* **56**, 1300-1309 (2021).
13. Neta, P., Huie, R. E. & Ross, A. B. Rate Constants for Reactions of Peroxyl Radicals in Fluid Solutions. *J. Phys. Chem. Ref. Data* **19**, 413-513 (1990). https://doi.org:10.1063/1.555854
14. Guo, K. *et al.* Roles of Bromine Radicals and Hydroxyl Radicals in the Degradation of Micropollutants by the UV/Bromine Process. *Environ. Sci. Technol.* **54**, 6415-6426 (2020).
15. Liu, T. *et al.* Selective Transformation of Micropollutants in Saline Wastewater by Peracetic Acid: The Overlooked Brominating Agents. *Environ. Sci. Technol.* **57**, 18940-18949 (2023).
16. Buxton, G. V., Greenstock, C. L., Helman, W. P. & Ross, A. B. Critical Review of rate constants for reactions of hydrated electrons, hydrogen atoms and hydroxyl radicals (⋅OH/⋅O^−^ in Aqueous Solution. *J. Phys. Chem. Ref. Data* **17**, 513-886 (1988).
17. Weinstein, J. & Bielski, B. H. J. Kinetics of the Interaction of HO_2_ and O_2_⁻ Radicals with Hydrogen Peroxide. The Haber-Weiss Reaction. *J. Am. Chem. Soc.* **101**, 58-62 (1979).
18. Shah, A. D., Liu, Z.-Q., Salhi, E., Höfer, T. & von Gunten, U. Peracetic Acid Oxidation of Saline Waters in the Absence and Presence of H_2_O_2_: Secondary Oxidant and Disinfection Byproduct Formation. *Environ. Sci. Technol.* **49**, 1698-1705 (2015).
19. Grebel, J. E., Pignatello, J. J. & A., W. Effect of Halide Ions and Carbonates on Organic Contaminant Degradation by Hydroxyl Radical-Based Advanced Oxidation Processes in Saline Waters. *Environ. Sci. Technol.* **44**, 6822-6828 (2010).
20. Wang, A. *et al.* Insights into the effects of bromide at fresh water levels on the radical chemistry in the UV/peroxydisulfate process. *Water Res.* **197**, 117042 (2021).
21. Yang, Y., Shin, J., Jasper, J. T. & Hoffmann, M. R. Multilayer Heterojunction Anodes for Saline Wastewater Treatment: Design Strategies and Reactive Species Generation Mechanisms. *Environ. Sci. Technol.* **50**, 8780-8787 (2016).
22. Zheng, W. *et al.* Trace Br^–^ Inhibits Halogenated Byproduct Formation in Saline Wastewater Electrochemical Treatment. *Environ. Sci. Technol.* **58**, 12212-12224 (2024).
